# An Enantioselective Decarboxylative Glycolate Aldol Reaction

**DOI:** 10.1021/acs.orglett.4c03251

**Published:** 2024-10-10

**Authors:** Md. Ataur Rahman, Mohammad Rehan, Torsten Cellnik, Brij Bhushan Ahuja, Alan R. Healy

**Affiliations:** Chemistry Program, New York University Abu Dhabi (NYUAD), Saadiyat Island, United Arab Emirates

## Abstract

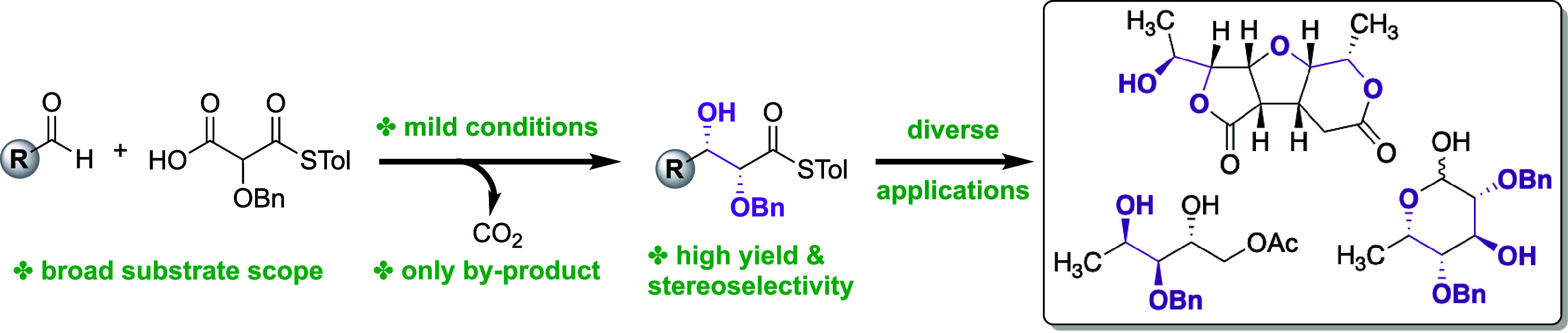

Herein, we report the application of a benzyloxy-functionalized malonic acid half thioester as an activated ester equivalent in a highly enantioselective decarboxylative glycolate aldol reaction. This robust method operates at ambient temperature, tolerates air and moisture, and generates CO_2_ as the only byproduct. The synthetic applicability of the method is demonstrated by the large-scale enantiodivergent synthesis of α-benzyloxy-β-hydroxybutyric acid thioester and its subsequent conversion to diverse polyoxygenated building blocks, deoxy-sugars, and (−)-angiopterlactone B.

Few asymmetric carbon–carbon bond-forming reactions have become as prominent as the aldol condensation in the synthesis of complex molecules.^1^ The asymmetric aldol reaction unites a carbonyl-derived enolate and an acceptor aldehyde to create a new carbon–carbon bond with the concomitant formation of two new stereogenic centers in a reliable and selective fashion. The glycolate aldol reaction, the condensation of an α-alkoxyacetate and an aldehyde, represents an important example of this transformation that allows for the stereocontrolled synthesis of enantiopure 1,2-diols. Vicinal *syn*-diols are ubiquitous units in carbohydrates and polyoxygenated natural products, in particular polyketides (Figure 1A).^2^ While the stereoselective synthesis of *syn*-diols using a chiral auxiliary-based approach with preformed enolates is well established,^3^ the catalytic enantioselective synthesis of these motifs remains challenging. Several catalytic enantioselective α-hydroxyacetyl aldol protocols have been reported using aromatic ketones^4^ or hydroxyacetone derivatives as the enolate partner.^5^ However, these protocols have not found widespread application in synthesis due to the limited synthetic versatility of the products. Examples of the more synthetically useful catalytic, enantioselective glycolate aldol reactions with the carbonyl donor at the carboxylic acid oxidation state remain scarce.^6,7^

**Figure 1:**
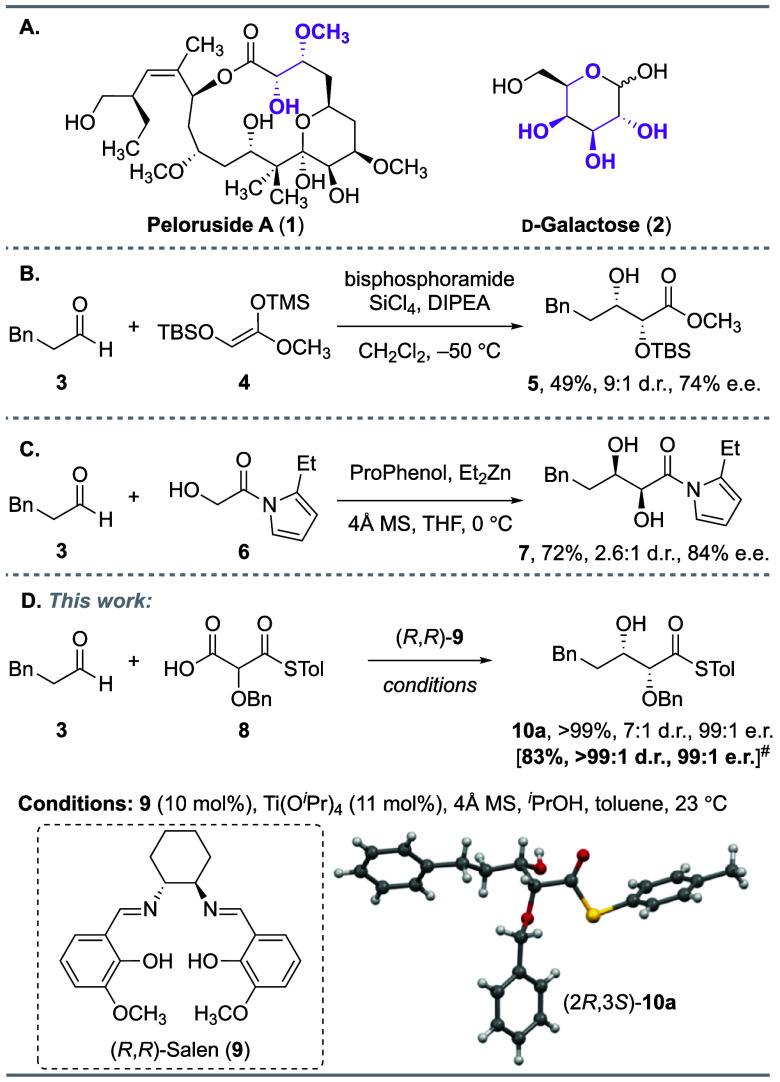
(A) Aliphatic *syn*-1,2-diols are prevalent motifs in natural products (**1**) and carbohydrates (**2**). (B) Chiral bisphosphoramide-catalyzed Mukaiyama glycolate aldol (Denmark). (C) Zinc-ProPhenol-catalyzed glycolate aldol (Trost). (D) This work. A decarboxylative *syn*-glycolate aldol reaction. ^#^After chromatographic purification. Red for oxygen, gray for carbon, yellow for sulfur, and white for hydrogen.

Mukaiyama-type glycolate aldol reactions that can deliver the 1,2-diol product in high selectivity have been reported by the groups of Kobayashi and Denmark.^8^ In the latter case, either *syn*- or *anti*-diol products could be attained by a chiral Lewis base-catalyzed addition of preformed glycolate-derived silyl ketene acetals to aldehydes (Figure 1B). The Trost group has pioneered the use of dinuclear zinc-ProPhenol catalysts for aldol-type transformations, including an aldol reaction using α-hydroxyketones as the carbonyl donor.^4b^ They subsequently reported a ProPhenol-catalyzed *syn*-selective glycolate aldol reaction using *N*-acylpyrrole **6** as an activated ester equivalent (Figure 1C).^9^ However, aliphatic aldehydes have proven to be troublesome substrates in these methods, delivering the corresponding *syn*-diol products (**5** and **7**) in moderate yield and stereoselectivity, thereby limiting the synthetic utility of these approaches. Therefore, a new catalytic glycolate aldol reaction that provides efficient and stereoselective access to synthetically important *syn*-diols is highly desirable.

Decarboxylative carbon–carbon bond-forming reactions using malonic acid half thioesters (MAHTs) as ester enolate surrogates have become increasingly popular in asymmetric synthesis.^10^ The presence of the additional carboxylic acid moiety on MAHT overcomes common challenges associated with using thioesters as templates in asymmetric reactions. The 1,3-dicarbonyl unit can coordinate the catalyst to generate an activated and more rigid thioester enolate nucleophile. Functionalized MAHTs have been successfully used as acetate,^11^ propionate,^12^ α-chloroacetate,^13^ and α-fluoroacetate surrogates^14^ in enantioselective aldol reactions. We previously reported a stereodivergent aldol reaction using alkyl-substituted MAHTs in the presence of a Ti(IV)-salen catalyst.^12b^ The desirable features of the method include high yield and selectivity, easy setup, scalability, and high atom economy. Herein, we report that this mild method tolerates α-alkoxy-functionalized MAHTs, thereby enabling the challenging synthesis of *syn*-1,2-diols through a catalytic glycolate aldol.

We began our studies by investigating the condensation of α-benzyloxy-MAHT **8** and hydrocinnamaldehyde **3** using our previously reported conditions (Figure 1D). The desired *syn*-diol product **10a** was obtained in quantitative yield with high diastereo- and enantioselectivity (7:1 dr, 99:1 er).^15^ Chromatographic purification provided **10a** in 83% yield as a single stereoisomer (>99:1 dr, 99:1 er). To assess the robustness of this transformation, we carried out sensitivity screening using a modified version of the protocol described previously by the Glorious group.^16^ The sensitivity of the reaction to 2-fold changes in temperature, concentration, or catalyst loading or the presence of water (1 equiv) and oxygen (open to air) was systematically examined (Table S1). The results demonstrate that the method is remarkably robust, as variation of the parameters mentioned above, or the presence of air or water, had little impact on the yield or stereoselectivity of the reaction.

We next explored the scope of the transformation (Figure 2). A range of branched and linear alkyl aldehydes provided the desired diol products in high yield and selectivity, including previously challenging α-branched substrates (**b** and **e**).^8d^ Alkyl aldehydes containing common functional groups, including chloride (**f**), alkyne (**g**), alkene (**h**), and a sensitive acetal (**m**), were all tolerated. Alkynyl (**k**) and phenylacetaldehyde (**i**) were suitable substrates, although the more hindered diphenylacetaldehyde (**j**) provided the desired product with only moderate enantioselectivity. The aldol reaction with methyl 5-oxopentanoate provided the aldol product in addition to a small quantity of lactone **10l** (Scheme S2). Exposure of the product mixture to *p*-toluenesulfonic acid (*p*TsOH) catalyzed the quantitative conversion of the aldol product to lactone **10l**. Importantly, α-alkoxy and β-amino aldehydes were suitable substrates, providing access to important precursors (**n** and **o**, respectively) for the *de novo* synthesis of monosaccharides and iminosugars.^17^

**Figure 2:**
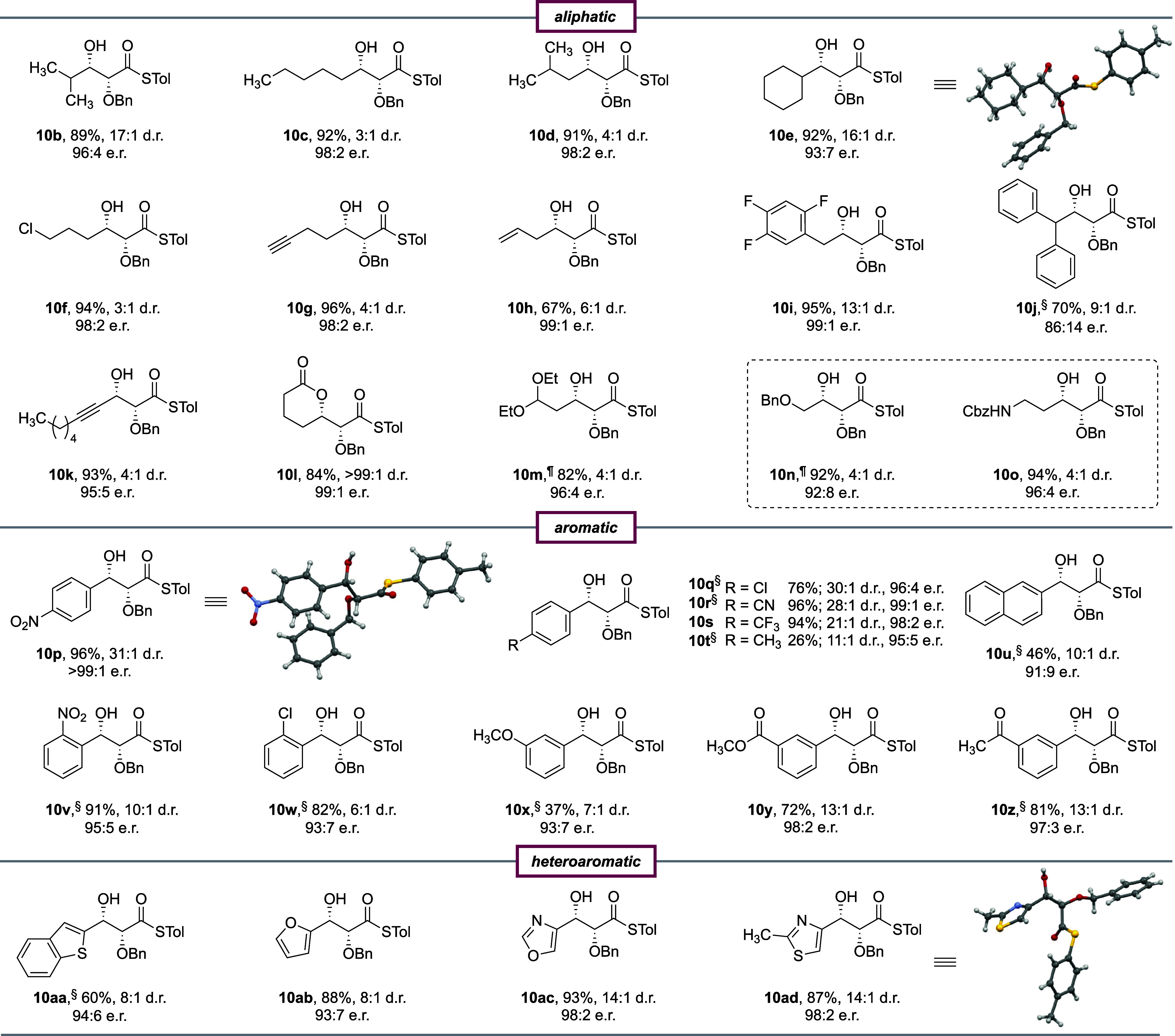
Aldehyde substrate scope for the *syn*-selective glycolate aldol reaction. Reactions were performed on a 0.3 mmol scale with respect to the aldehyde in toluene (0.1 M) for 24–48 h unless otherwise stated. Isolated yields after chromatographic purification are reported. ^§^Reaction performed for 48 h in toluene (0.4 M). ^¶^Reaction performed using 15 mol % Ti(O^*i*^Pr)_4_. The dr and er values were determined by supercritical fluid chromatography (SFC) with a chiral stationary phase.

The *syn*-selective glycolate aldol reaction also proved to be general for a range of structurally and electronically diverse aromatic aldehydes. Aromatic aldehydes with *meta*, *ortho*, and *para* substitutions gave the aldol products in excellent yields and high selectivity. A broad range of substituents were tolerated, including halides (**q** and **w**), trifluoromethyl (**s**), nitro (**p** and **v**), cyano (**r**), and reactive functionalities such as esters (**y**) and ketones (**z**). While high selectivity was observed across all substituents, the use of electron rich aromatic substrates such as 2-naphthaldehyde (**u**), or 3-methoxybenzaldehyde (**x**) resulted in a sluggish reaction, providing the diol product in moderate yield. Finally, aldehydes containing prevalent heteroaromatic scaffolds, such as benzothiophene (**aa**), furan (**ab**), oxazole (**ac**), and thiazole (**ad**), were converted into the corresponding diol products in high yield and selectivity. The expected absolute and relative configurations of aliphatic diol **10e**, aromatic diol **10p**, and heteroaromatic diol **10ad** were confirmed by X-ray crystal analysis.

The utility of this method was further demonstrated through the synthesis of *syn*-(2*S*,3*R*)-dihydroxybutyric acid (thio)ester **10ae** and its enantiomer, (2*R*,3*S*)-**10ae**, which are important building blocks for the synthesis of complex carbohydrates and other densely oxygenated natural products (Scheme 1). Prior syntheses of this valuable substrate have typically employed Sharpless dihydroxylation of the corresponding ester.^18^ By employing our decarboxylative glycolate aldol, the multigram synthesis of both enantiomers of *syn*-2,3-dihydroxybutyric acid thioester **10ae** was achieved in a single atom economic transformation. This approach avoids the olefination reaction, removes the need for toxic osmium catalysts, and is facile to run on a large scale. Furthermore, the thioester provides a versatile functional handle that can be directly converted into carboxylic acid derivatives and ketones.

**Scheme 1:**
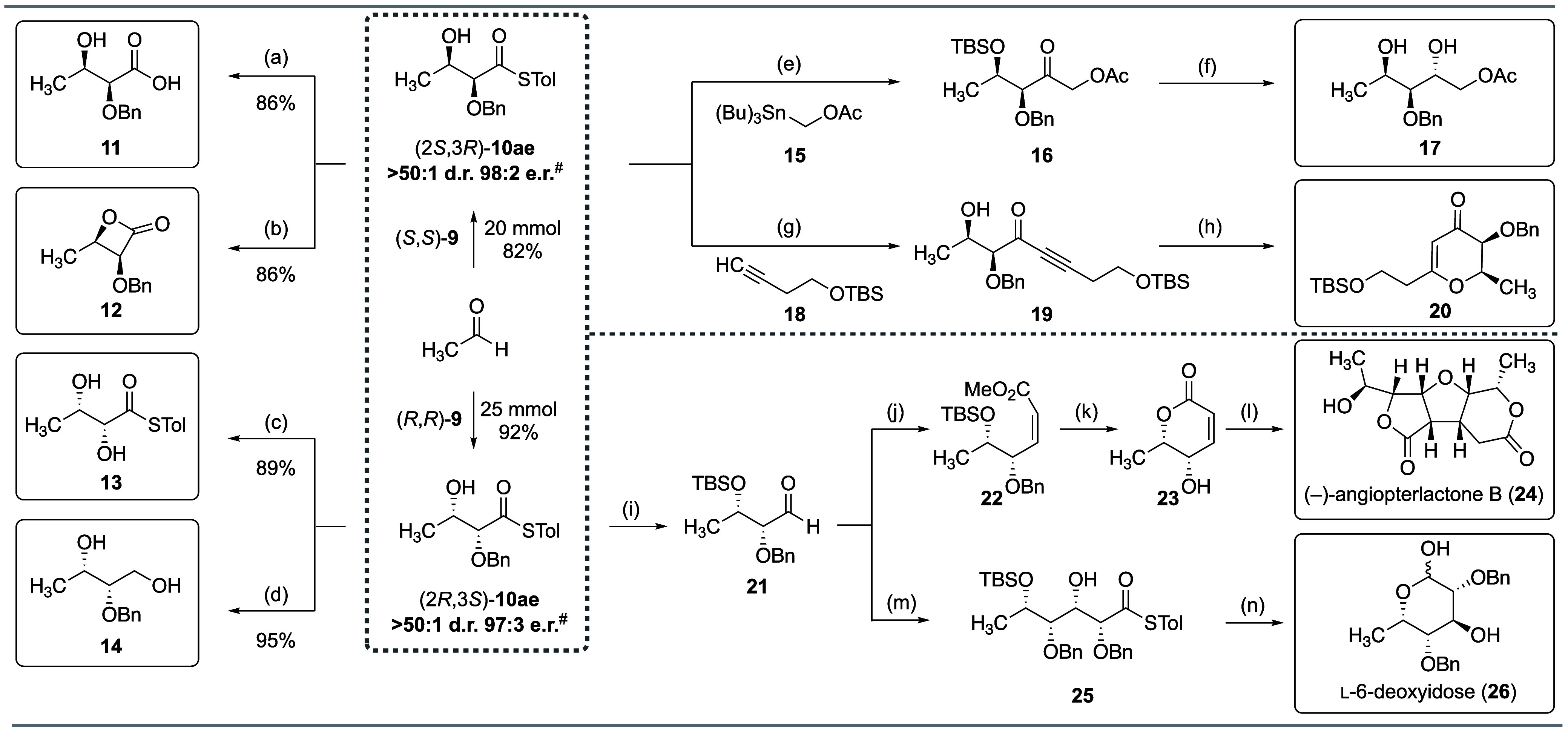
Multigram-Scale Synthesis and Functionalization of Both Enantiomers of Thioester Product **10ae** See the Supporting Information for experimental details. ^#^After chromatographic purification. Conditions: (a) LiOH·H_2_O (1.1 equiv), aqueous H_2_O_2_ (30%, 10 equiv), 5:1 THF/H_2_O, 23 °C, 12 h; (b) AgTFA (1.5 equiv), DIPEA (1.6 equiv), CH_2_Cl_2_, 24 h, 23 °C; (c) TiCl_4_ (1.0 M in CH_2_Cl_2_, 2 equiv), CH_2_Cl_2_, 0 °C, 16 h; (d) LiAlH_4_ (1.0 M in Et_2_O, 2.2 equiv), Et_2_O, 3 h, 0 °C; (e) (i) TBSOTf (1.2 equiv), DIPEA (1.5 equiv), CH_2_Cl_2_, 1 h, 0 °C, 94%; (ii) **15** (6 equiv), CuOAc (2 equiv), DMF, 80 °C, 24 h, 73%; (f) (i) *p*TsOH (20 mol %), 1:1 CH_2_Cl_2_/MeOH, 6 h, 23 °C, 66%; (ii) Me_4_NHB(OAc)_3_ (28.5 equiv), 1:1 CH_3_CN/AcOH, −40 °C, 18 h, 69%; (g) **18** (2 equiv), CuI (2 equiv), TFP (40 mol %), Pd(dppf)Cl_2_ (20 mol %), DIPEA (1.01 equiv), DMF, 50 °C, 48 h, 72%; (h) AuCl (5 mol %), CH_2_Cl_2_, 20 h, 23 °C, 74%; (i) (i) TBSOTf (1.2 equiv), DIPEA (1.5 equiv), CH_2_Cl_2_, 1 h, 0 °C, 99%; (ii) Pd(OAc)_2_ (30 mol %), MgSO_4_ (15 equiv), Et_3_SiH (10 equiv), 2 h, 0 °C, acetone, 86%; (j) (CF_3_CH_2_O)_2_P(O)CH_2_CO_2_CH_3_ (2 equiv), NaH (2 equiv), −78 °C, 2 h, 90%; (k) (i) *p*TsOH (20 mol %), CH_2_Cl_2_, 23 °C, 24 h, 90%; (ii) TiCl_4_ (1 M in CH_2_Cl_2_, 2 equiv), CH_2_Cl_2_, 0 °C, 16 h, 88%; (l) K_2_CO_3_ (20 mol %), DCE, 70 °C, 16 h, 16%; (m) **9** (1.2 equiv), (*R*,*R*)-**7** (10 mol %), Ti(O^*i*^Pr)_4_ (11 mol %), ^*i*^PrOH (1 equiv), toluene, 4 Å molecular sieves, 23 °C, 24 h, 81%; (n) (i) CSA (20 mol %), 1:1 CH_2_Cl_2_/MeOH, 50 °C, 20 h, 85%; (ii) DIBAL-H (1 M in toluene, 2 equiv), CH_2_Cl_2_, −78 °C, 2 h, 83%.

The enantioenriched *syn*-diol thioester products can be readily transformed into synthetically important chiral building blocks (Scheme 1). Hydrolysis of (2*S*,3*R*)-thioester product **10ae** provided differentially protected *syn*-(2*S*,3*R*)-dihydroxybutyric acid **11**. β-Lactones serve as versatile synthetic intermediates in the synthesis of numerous important compound classes, as they undergo a variety of transformations in a stereospecific fashion.^19^ The glycolate aldol products can be readily converted into α-alkoxy-β-lactones (such as **12**) by silver trifluoroacetate (AgTFA)-catalyzed intramolecular lactonization. Unprotected *syn*-(2*R*,3*S*)-dihydroxybutyric acid thioester **13** is accessed by the debenzylation of (2*R*,3*S*)-**10ae** using TiCl_4_ in dichloromethane. Finally, reduction of (2*R*,3*S*)-**10ae** with lithium aluminum hydride (LiAlH_4_) afforded triol **14** in 95% yield. Importantly, all of these transformations were achieved under mild reaction conditions without a loss of optical purity.

The thioester can also serve as a versatile functional handle for metal-catalyzed cross-coupling reactions to generate enantioenriched 2,3-dihydroxyketones (Scheme 1). *O*-Silyl protection of the β-hydroxy group of (2*S*,3*R*)-**10ae** followed by a copper-catalyzed cross-coupling with acetoxymethyl stannane **15** yielded protected d-deoxyxylulose **16**,^20^ a valuable polyoxygenated intermediate containing four orthogonal oxygen atoms that can each be selectively functionalized. *p*TsOH-mediated desilylation of **16** followed by subjection of the corresponding β-hydroxy ketone to tetramethylammonium triacetoxyborohydride [Me_4_NHB(OAc)_3_] selectively reduced it to *anti*-diol **17**.^21^ Thioester (2*S*,3*R*)-**10ae** can also be converted into α,β-acetylenic ketone **19** via a Pd-catalyzed cross-coupling with alkyne **18**.^22^ Subjecting chiral ynone **19** to a gold-catalyzed intramolecular oxy-Michael reaction directly afforded tetrahydropyrone **20**.^23^

*O*-Silyl protection of (2*R*,3*S*)-*syn* aldol product **10ae** and subsequent Pd-mediated Fukuyama reduction gave protected dihydroxy-aldehyde **21**, a valuable intermediate in the synthesis of 6-deoxy-sugars and polyoxygenated natural products (Scheme 1). Olefination of aldehyde **21** using Still–Gennari modified Horner–Wadsworth–Emmons conditions yielded *Z*-olefinic ester **22** in 90% yield.^24^ Treatment of **22** with *p*TsOH promoted desilylation of the *O*-TBS-protected β-hydroxyl group and concomitant *in situ* cyclization to the corresponding lactone. TiCl_4_-mediated debenzylation yielded α,β-unsaturated δ-lactone **23**. The groups of Lawrence^25^ and Bhattacharya^26^ have previously demonstrated that lactone **23** could undergo a biomimetic dimerization under mildly basic conditions to generate (−)-angiopterlactone B (**24**) as a single diastereomer. Indeed, subjecting **23** to K_2_CO_3_ in 1,2-dichloroethane (DCE) provided the desired natural product in 16% yield. Finally, aldehyde **21** can be converted into stereochemically diverse deoxyhexoses via several known protocols.^17a,27^ In this case, subjecting aldehyde **21** to a second (*R*,*R*)-salen-catalyzed decarboxylative glycolate aldol yielded *syn*–*syn* aldol product (2*R*,3*S*,4*S*,5*S*)-**25** in 81% yield. Camphorsulfonic acid (CSA)-mediated deprotection of the *O*-TBS-protected alcohol initiated an intramolecular cyclization to directly yield the corresponding lactone. Partial reduction of the lactone using DIBAL-H afforded an inseparable 3:1 anomeric mixture of 2,4-bis(*O*-benzyl)-l-6-deoxyidose (**26**) in 83% yield.^17a^

In conclusion, we disclose a catalytic decarboxylative glycolate aldol reaction using OBn-MAHT as an activated glycolate surrogate.^28^ The mild and robust method delivers highly enantioenriched aromatic and aliphatic *syn*-diols. The α-benzyloxy-β-hydroxy thioester products serve as versatile building blocks in the stereoselective synthesis of valuable polyoxygenated molecules, as demonstrated by the multigram enantiodivergent synthesis and derivatization of the dihydroxybutyric acid thioester. This method adds to the ever-growing toolbox of enantioselective decarboxylative C–C bond-forming reactions using functionalized MAHTs.

## Data Availability

The data underlying this study are available in the published article and its Supporting Information.

## Supporting Information Available

Schemes, figures, and tables; detailed experimental procedures; and characterization data for all new compounds (PDF)
