# Supplementary material for: An Enantioselective Decarboxylative Glycolate Aldol Reaction
Source: Org Lett. 2024 Oct 10;26(42):9040–5. doi: 10.1021/acs.orglett.4c03251 (PMC11519920; doi:10.1021/acs.orglett.4c03251)
Supplement: Supplementary file 1 — ol4c03251_si_001.pdf [file ol4c03251_si_001.pdf]

Supporting Information

An Enantioselective Decarboxylative Glycolate Aldol Reaction

Md. Ataur Rahman, Mohammad Rehan, Torsten Cellnik, Brij Bhushan Ahuja, Alan R. Healy*

Chemistry Program, New York University Abu Dhabi (NYUAD), Saadiyat Island, United Arab Emirates (UAE)

Email: alan.healy@nyu.edu

Table of Contents

Reaction Sensitivity Assessment 2
Figure S1. Development of an anti-glycolate aldol. 3
Experimental Procedures 4
General Information 4
General methods 5
Synthetic Procedures (MAHT Synthesis): 6
Synthetic Procedures (glycolate aldol): 8
Large Scale reactions: 23
Synthetic Procedures (transformations): 24
Catalog of SFC Spectra 33
Catalog of Nuclear Magnetic Resonance Spectra 66
Catalog of X-ray data 178
Bibliography 183

Reaction Sensitivity Assessment

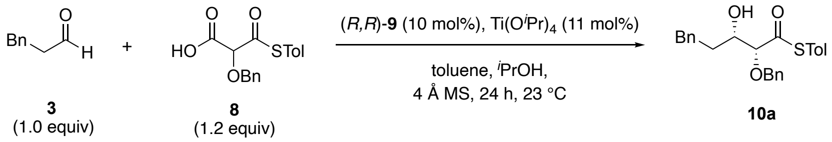

Entry	Modification	Deviation from standard condition	Yield [%]	Deviation/%	*d.e.* [%]	Deviation/%	*e.e.* [%]	Deviation/%
**1**	standard reaction	-	99	-	64	-	96	-
**2**	Low T	10 °C	80	-19%	62	-2%	96	0%
**3**	High T	40 °C	100	+1%	66	+2%	91	-5%
**4**	Low c	0.05 M	80	-19%	44	-20%	83	-13%
**5**	High c	0.20 M	100	+1%	62	-2%	97	+1%
**6**	Water	1 equiv of water	100	+1%	60	-4%	97	+1%
**7**	Open to air	Open to air	100	+1%	64	0%	97	+1%
**8**	Low cat.	**9** (5 mol%) and Ti(O^{i}Pr)$_4$ (5.5 mol%)	99	0%	58	-6%	94	-2%
**9**	High cat.	**9** (20 mol%) and Ti(O^{i}Pr)$_4$ (22 mol%)	99	0%	56	-8%	95	-1%

Table S1. Sensitivity assessment of reaction yield, diastereoselectivity and enantioselectivity of the glycolate aldol reaction. Yield and diastereoselectivity were determined by crude NMR utilizing ethylene carbonate as internal standard. Enantioselectivity was determined by supercritical fluid chromatography (SFC) with a chiral stationary phase. Lowering the reaction temperature (entry 2) and reducing the concentration of the aldehyde (entry 4) resulted in decreased conversion to the product. The lower concentration also led to significantly poorer stereoselectivity. Increasing the reaction temperature (entry 3) led to a slight decrease in enantioselectivity. No significant changes were observed with higher concentration (entry 5), the addition of water (entry 6), or conducting the reaction in an open-air environment (entry 7). Both low and high catalyst loadings (entries 8 and 9) exhibited slight variations in the degree of diastereoselectivity (*d.e.*).

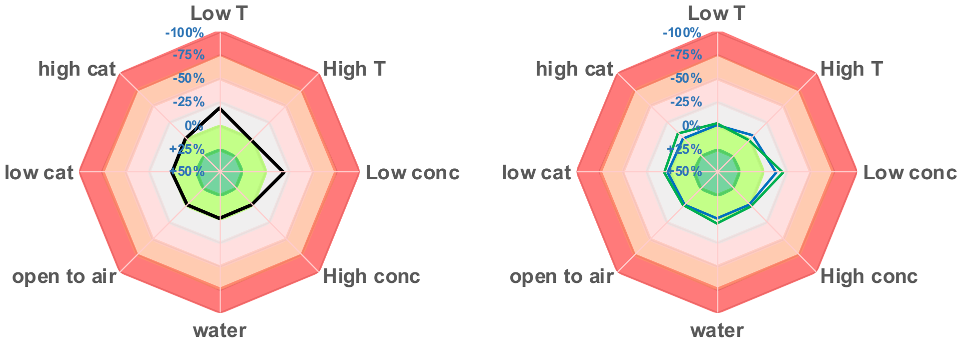

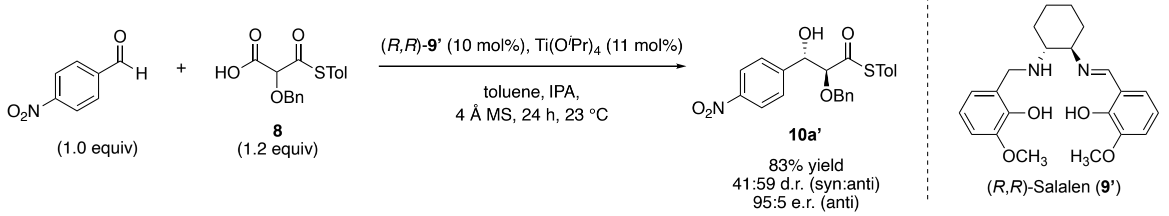

Figure S1. Development of an *anti*-glycolate aldol.

To investigate the stereodivergent glycolate aldol reaction, we employed the Salalen ligand (*S*,*S*)-**9a** under previously optimized conditions using 4-nitrobenzaldehyde as the aldehyde substrate.[1] Initial results provided the desired *anti*-aldol **10a**' in high yield and enantioselectivity; but with low diasteroselectivity (~1.5:1 *anti*-selectivity). A subsequent screening of a small library of salalen ligands, additives, and other modifications to the reaction conditions did not significantly improve the diastereoselectivity. A more extensive exploration of the *anti*-glycolate aldol reaction and mechanistic studies are on-going.

Experimental Procedures

General Information

General Experimental Procedures. All reactions were performed in a single-neck, flame-dried, round-bottomed flask fitted with rubber septa under a positive pressure of argon unless otherwise noted. Reactions that required heating were carried out in temperature-controlled heating blocks. Air- and moisture-sensitive liquids were transferred via syringe or stainless-steel cannula. Organic solutions were concentrated by rotary evaporation at 30–32 °C. Flash-column chromatography was performed employing silica gel (60 Å, 40–63 µm particle size) purchased from SiliCycle (Quebec City, CA). Analytical thin-layered chromatography (TLC) was performed using glass plates pre-coated with silica gel (0.25 mm, 60 Å pore size) impregnated with a fluorescent indicator (254 nm). TLC plates were visualized by exposure to ultraviolet light (UV) or by staining with potassium permanganate ($KMnO_4$) and subsequent heating.

Materials. Commercial solvents and reagents were used as received with the following exceptions. Acetonitrile, toluene, dichloromethane, *N,N*-dimethylformamide and tetrahydrofuran were purified and dried *via* PureSolv-system (inert®). Cyclohexanecarboxaldehyde, hydrocinnamaldehyde were distilled *in vacuo* prior to use. The ligands **9**[1] and **S1**[1], substrate **S2**[2], **15**[3] and **18**[1] and the aldehydes for the synthesis *of* **10h**[4], **10i**[4], **10m**[5] and**10o**[6] were synthesized according to literature. Molecular sieves were activated in an oven at 115 °C overnight. The titanium (IV) isopropoxide solution in toluene (0.50 M) was freshly prepared prior to use.

Instrumentation. Proton nuclear magnetic resonance spectra (^{1}H NMR) were recorded at 500 MHz at 24 °C. Chemical shifts are expressed in parts per million (ppm, δ scale) downfield from tetramethylsilane and are referenced using residual undeuterated solvent ($CDCl_3$, δ 7.26; CD_3OD, δ 3.31). Data are represented as follows: chemical shift, multiplicity (s = singlet, d = doublet, t = triplet, q = quartet, m = multiplet and/or multiple resonances, br = broad, app = apparent), coupling constant in Hertz, integration, and assignment. Proton-decoupled carbon nuclear magnetic resonance spectra (^{13}C NMR) were recorded at 126 MHz at 24 °C. Chemical shifts are expressed in parts per million (ppm, δ scale) downfield from tetramethylsilane and are referenced to the carbon resonances of the solvent ($CDCl_3$, δ 77.16, CD_3OD, δ 49.00). Signals of protons and carbons were assigned, as far as possible, by using [^{13}C] DEPT (Distortionless Enhancement by Polarization Transfer) and the following two-dimensional NMR spectroscopy techniques: [^{1}H, ^{1}H] COSY (Correlation Spectroscopy), [^{1}H, ^{13}C] HSQC (Heteronuclear Single Quantum Coherence) and long range [^{1}H, ^{13}C] HMBC (Heteronuclear Multiple Bond Connectivity). Fluorine nuclear magnetic resonance spectra (^{19}F NMR) were recorded at 470 MHz at 24 °C. Analytical liquid chromatography/mass spectrometry (SFC (Supercritical Fluid Chromatography)/MS) was performed on Agilent SFC/MS instrument (1260 Infinity II) equipped with a InfinityLab Poroshell 120 Hilic column (2.7 µm particle size, 4.6 × 150 mm), electrospray (ESI) mass spectrometry detector, and photodiode array detector. Samples were eluted with methanol (containing 0.1% formic acid and 0.1% ammonium hydroxide)-carbon dioxide (10% methanol–carbon dioxide for 2 min → linear gradient to 30% methanol–carbon dioxide over 2 min → 30% methanol–carbon dioxide for 2 min) at a flow rate of 2 mL/min. High-resolution mass spectrometry (HRMS) was obtained on an UPLC/HRMS instrument (Agilent 1290 Infinity II) equipped with a Q-TOF (UHD Accurate-Mass) and photodiode array detector. Samples were eluted over a guard column with 50% acetonitrile–water containing 0.1% formic acid for 1 min, at a flow rate of 300 µL/min. Chiral SFC was measured on an Agilent 1260 instrument with an IA-3 CHIRALPAK column (4.6 mm × 100 mm, 3 µm), IB-3 CHIRALPAK column (4.6 mm × 100mm, 3 µm), IC-3 CHIRALPAK column (4.6 mm × 100mm, 3 µm), IG-3 CHIRALPAK column (4.6 mm × 100mm, 3 µm), OD-H CHIRALPAK column (4.6 mm × 100mm, 5 µm) and OJ-H CHIRALPAK column (4.6 mm × 100mm, 5 µm). Optical rotations were measured on a polarimeter Anton Paar (MCP 5100) at 20 °C at a wavelength of λ 589 nm in a 1 mL quartz cell (0.5 dm length). The concentration is given in g/100 mL. The X-ray crystal structures were measured on a Bruker D8 Apex Duo system with PHOTON II detector.

General methods

General method for racemic aldol reaction:

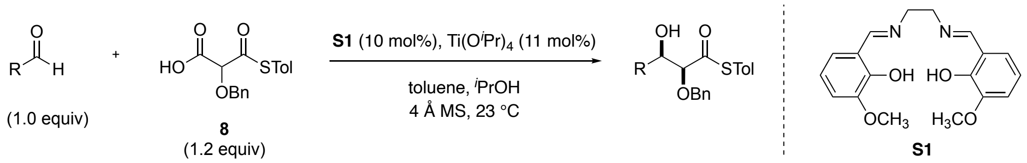

To a suspension of the achiral salen **S1** (0.03 mmol, 10 mol%) and activated molecular sieves (4 Å, 200 mg) in toluene (0.10 M) was added 2-propanol (0.30 mmol, 1.00 equiv) followed by a solution of titanium (IV) isopropoxide (0.50 M in toluene; 0.03 mmol, 11 mol%). The catalyst mixture was stirred for 1 h at 23 °C. Malonic acid half thioester **8** (0.36 mmol, 1.20 equiv) was added to the catalyst mixture in one portion. The resulting red solution was stirred for 15 min at 23 °C followed by addition of the aldehyde (0.30 mmol, 1 equiv). The reaction mixture was stirred at 23 °C until consumption of the aldehyde was observed. The solution gradually turned to a yellow color over the course of the reaction. The product mixture was filtered through celite and rinsed with ethyl acetate. The filtrate was concentrated and the residue was purified by column chromatography.

General method for the aldol reaction:

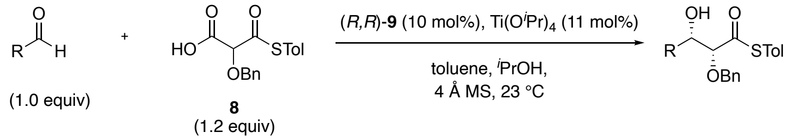

To a suspension of (*R,R*)-**9** (0.03 mmol, 10 mol%) and activated molecular sieves (4 Å, 200 mg) in toluene (0.10 M) was added 2-propanol (0.30 mmol, 1.00 equiv) followed by a solution of titanium (IV) isopropoxide (0.50 M in toluene; 0.03 mmol, 11 mol%). The catalyst mixture was stirred for 1 h at 23 °C. Malonic acid half thioester **8** (0.36 mmol, 1.20 equiv) was added to the catalyst mixture in one portion. The resulting red solution was stirred for 15 min at 23 °C followed by addition of the aldehyde (0.30 mmol, 1 equiv). The reaction mixture was stirred at 23 °C until consumption of the aldehyde was observed. The solution gradually turned to a yellow color over the course of the reaction. The product mixture was filtered through a celite and rinsed with ethyl acetate. The filtrate was concentrated and the residue was purified by column chromatography. **For deviations from the general procedure (for example catalyst loading, reaction concentration, time or scale) see the corresponding entries.*

Synthetic Procedures (MAHT Synthesis):

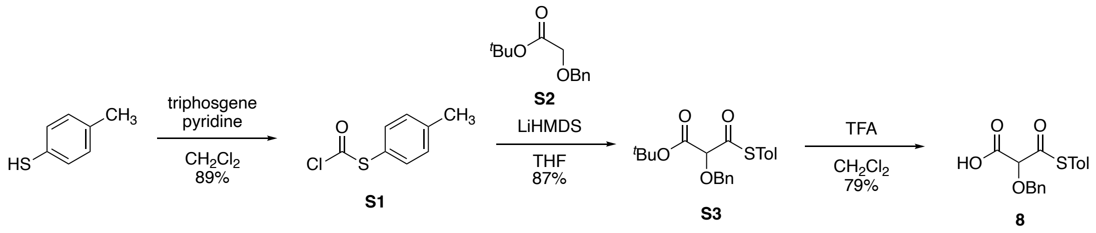

Scheme S1. *Synthesis of MAHT* ***8****.*

Synthesis of S-(p-tolyl) carbonochloridothioate **S1**

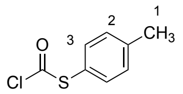

To a solution of 4-methylbenzenethiol (40.0 g, 322 mmol, 1 equiv) in dichloromethane (0.50 M, 645 mL) was added triphosgene (47.8 g, 161 mmol, 0.50 equiv) at 0 °C. After 5 min a solution of pyridine (25.9 mL, 322 mmol, 1.00 equiv) in dichloromethane (5.00 M, 50.0 ml) was slowly added at 0 °C. The reaction mixture was stirred for 1 h at 0 °C. The product mixture was diluted with ice-cold water. The organic layer was separated and the aqueous layer was washed with dichloromethane (3 ×). The combined organic layers were washed with cold water (2 ×), dried over sodium sulfate, filtered, and the filtrate was concentrated. The residue was purified by vacuum distillation to yield **S1** as a colorless oil (53.5 g, 89%).

^{1}H NMR (500 MHz, $CDCl_3$) δ 7.46 – 7.40 (m, 2H, H_2), 7.30 – 7.25 (m, 2H, H_3), 2.41 (s, 3H, H_1).

^{13}C NMR (126 MHz, $CDCl_3$) δ 165.5 (C), 141.6 (C), 134.1 (2 × CH), 130.6 (2 × CH), 123.8 (C), 21.5 (CH_3).

R_f = 0.36 (10% ethyl acetate-hexane; UV).

Synthesis of tert-butyl 2-(benzyloxy)-3-oxo-3-(p-tolylthio)propanoate **S3**

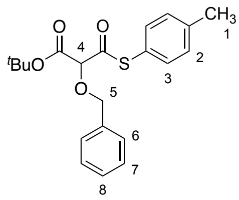

To a solution of lithium bis(trimethylsilyl)amide (LiHMDS, 45.0 mL, 45.0 mmol, 1.0 M in tetrahydrofuran, 2.00 equiv) in tetrahydrofuran (0.19 M, 120 mL) was added dropwise a solution of *tert*-butyl 2-(benzyloxy)acetate **S2** (5.00 g, 22.5 mmol, 1 equiv) in tetrahydrofuran (1.5 M, 15.0 mL) at -78 °C. The reaction mixture was stirred for 1 h at –78 °C, followed by dropwise addition of a solution of **S1** (4.62 g, 24.7 mmol, 1.10 equiv) in tetrahydrofuran (1.5 M, 15.0 mL). The product mixture was stirred for 30 min at –78 °C. The reaction mixture was quenched at –78 °C by the addition of saturated aqueous ammonium chloride solution and ethyl acetate. The organic layer was separated and the aqueous layer was extracted with ethyl acetate (2 ×). The combined organic layers were dried over sodium sulfate, filtered and the filtrate was concentrated. The residue was purified by flash column chromatography (eluting with 5% ethyl acetate-hexane initially, grading to 15% ethyl acetate-hexane). The product **S3** was obtained as a yellow oil (7.27 g, 87%).

^{1}H NMR (500 MHz, $CDCl_3$) δ 7.48 – 7.44 (m, 2H, H_7), 7.42 – 7.33 (m, 3H, $H_{6,8}$), 7.31 – 7.27 (m, 2H, H_2), 7.25 – 7.21 (m, 2H, H_3), 4.81 (d, J = 11.8 Hz, 1H, H_5), 4.77 (d, J = 11.8 Hz, 1H, H_5), 4.52 (s, 1H, H_4), 2.38 (s, 3H, H_1), 1.48 (s, 9H, H_9).

^{13}C NMR (126 MHz, $CDCl_3$) δ 194.7 (C), 165.1 (C), 140.0 (C), 136.2 (C), 134.8 (2 × CH), 130.3 (2 × CH), 128.7 (2 × CH), 128.5 (CH), 128.4 (2 × CH), 123.3 (C), 84.0 (CH), 83.5 (C), 73.3 (CH_2), 28.0 (3 × CH_3), 21.5 (CH_3).

HRMS-CI (m/z): $[M + Na]^+$ calcd for $C_{21}H_{24}O_4SNa$, 395.1288; found, 395.1280.

R_f = 0.46 (20% ethyl acetate-hexane; UV).

Synthesis of 2-(benzyloxy)-3-oxo-3-(p-tolylthio)propanoic acid **8**

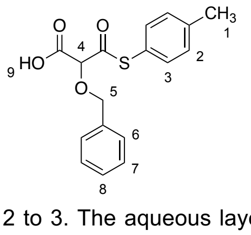

To a solution of **S3** (7.27 g, 19.5 mmol, 1 equiv) in dichloromethane (0.20 M, 225 mL) was added trifluoroacetic acid (30.0 mL, 390 mmol, 20.0 equiv) at 0 °C. The reaction mixture was stirred for 30 min at 0 °C, followed by 16 h at 23 °C. The product mixture was concentrated and diluted with diethyl ether. The organic layer was washed with a saturated aqueous sodium bicarbonate solution (6 ×). The combined aqueous layers were acidified with aqueous hydrogen chloride solution (12 N) at 0 °C until the pH reached 2 to 3. The aqueous layer was extracted with dichloromethane (4 ×). The combined organic layers were dried over sodium sulfate,

filtered, and the filtrate was concentrated. The residue was purified by column chromatography (eluting with 5% ethyl acetate-hexane initially, grading to 40% ethyl acetate-hexane) to yield **9** as a white solid (4.90 g, 79%).

^{1}H NMR (500 MHz, $CDCl_3$) δ 9.51 (brs, 1H, H_9), 7.49 – 7.45 (m, 2H, H_7), 7.44 – 7.35 (m, 3H, $H_{6,8}$), 7.33 – 7.29 (m, 2H, H_2), 7.26 – 7.22 (m, 2H, H_3), 4.86 (d, J = 11.7 Hz, 1H, H_5), 4.83 (d, J = 11.7 Hz, 1H, H_5), 4.69 (s, 1H, H_4), 2.39 (s, 3H, H_1).

^{13}C NMR (126 MHz, $CDCl_3$) δ 194.6 (C), 169.9 (C), 140.3 (C), 135.5 (C), 134.7 (2 × CH), 130.3 (2 × CH), 128.9 (2 × CH), 128.8 (CH), 128.6 (2 × CH), 122.5 (C), 82.7 (CH), 73.9 (CH_2), 21.5 (CH_3).

HRMS-CI (m/z): $[M + Na]^+$ calcd for $C_{17}H_{16}O_4SNa$, 339.0662; found, 339.0658.

R_f = 0.38 (50% ethyl acetate-hexane; UV).

Synthetic Procedures (glycolate aldol):

Synthesis of S-(p-tolyl) (2R,3S)-2-(benzyloxy)-3-hydroxy-5-phenylpentanethioate **10a**

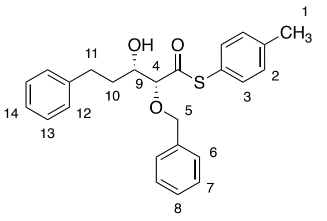

Following the general method, (*R*,*S*)-**10a** was prepared from hydrocinnamaldehyde **3** (99 μL, 0.75 mmol, 1 equiv) and MAHT **8** (283 mg, 0.89 mmol, 1.20 equiv). The reaction mixture was stirred for 24 h. The residue was purified by column chromatography (eluting with 5% ethyl acetate–hexane initially, grading to 30% ethyl acetate–hexane). The product was obtained as a colorless oil (300 mg, 99%) as a mixture of diastereomers (d.r. (*syn:anti*) = 7:1), which were separated by column chromatography (eluting with 5% ethyl acetate–hexane initially, grading to 30% ethyl acetate–hexane). The pure *syn* product was obtained as a colorless oil (252 mg, 83%, d.r. (*syn:anti*) > 99:1).

syn diastereomer (pure):

^{1}H NMR (500 MHz, $CDCl_3$) δ 7.46 – 7.33 (m, 5H, $H_{6\text{-}8}$), 7.29 – 7.14 (m, 9H, $H_{2,3,12\text{-}14}$), 4.93 (d, *J* = 11.1 Hz, 1H, H_5), 4.55 (d, *J* = 11.1 Hz, 1H, H_5), 3.98 (d, *J* = 4.6 Hz, 1H, H_4), 3.88 (ddd, *J* = 8.8, 4.6, 4.6 Hz, 1H, H_9), 2.82 (ddd, *J* = 13.9, 8.8, 5.9 Hz, 1H, H_{11}), 2.63 (ddd, *J* = 13.9, 7.9, 7.9 Hz, 1H, H_{11}), 2.41 (brs, 1H, OH), 2.38 (s, 3H, H_1), 1.89 – 1.80 (m, 2H, H_{10}).

^{13}C NMR (126 MHz, $CDCl_3$) δ 201.2 (C), 141.6 (C), 139.9 (C), 136.6 (C), 134.7 (2 × CH), 130.2 (2 × CH), 128.8 (2 × CH), 128.63 (2 × CH), 128.62 (2 × CH), 128.57 (CH), 128.5 (2 × CH), 126.0 (CH), 123.5 (C), 87.2 (CH), 74.5 (CH_2), 72.1 (CH), 34.6 (CH_2), 31.7 (CH_2), 21.5 (CH_3).

mixture of diastereomers: SFC (IC-3, CO_2/CH_3OH = 90:10, 2.0 mL/min, 298 K, 254 nm): t_R(*anti*, major) = 4.8 min, t_R(*anti*, minor) = 6.0 min, t_R(*syn*, major) = 7.2 min, t_R(*syn*, minor) = 7.8 min, d.r. (*syn:anti*) = 7:1, e.r.(*syn*) = 99:1 (97% *ee*), e.r.(*anti*) = 87:13 (74% *ee*).

syn product (pure): SFC (IC-3, CO_2/CH_3OH = 90:10, 2.0 mL/min, 298 K, 254 nm): t_R(*anti*, major) = 4.8 min, t_R(*anti*, minor) = 6.0 min, t_R(*syn*, major) = 7.2 min, t_R(*syn*, minor) = 7.8 min, d.r. (*syn:anti*) > 99:1, e.r.(*syn*) = 99:1 (97% *ee*).

HRMS-CI (m/z): $[M + Na]^+$ calcd for $C_{25}H_{26}O_3SNa$, 429.1495; found, 429.1500.

R_f = 0.55 (20% ethyl acetate-hexane; UV).

$[\alpha]_D^{25}$ = +100.8 (c 0.5, $CHCl_3$)

The absolute stereochemistry for (2*R*,3*S*)-**10a** was confirmed by x-ray crystallography (see *Catalog of X-ray data*). The crystal was grown by a slow evaporation of dichloromethane.

Synthesis of S-(p-tolyl) (2R,3S)-2-(benzyloxy)-3-hydroxy-4-methylpentanethioate **10b**

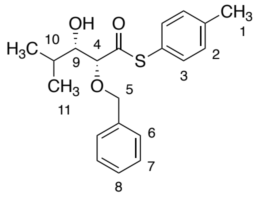

Following the general method, (*R*,*S*)-**10b** was prepared from isobutyraldehyde (27 μL, 0.30 mmol, 1 equiv) and MAHT **8** (114 mg, 0.36 mmol, 1.20 equiv). The reaction mixture was stirred for 48 h. The residue was purified by column chromatography (eluting with 5% ethyl acetate–hexane initially, grading to 30% ethyl acetate–hexane). The product was obtained as a colorless oil (92 mg, 89%).

Major diastereomer:

^{1}H NMR (500 MHz, $CDCl_3$) δ 7.50 – 7.35 (m, 5H, $H_{6\text{-}8}$), 7.33 (d, *J* = 8.1 Hz, 2H, H_2), 7.25 (d, *J* = 8.1 Hz, 2H, H_3), 4.99 (d, *J* = 11.0 Hz, 1H, H_5), 4.57 (d, *J* = 11.0 Hz, 1H, H_5), 4.13 (d, *J* = 3.7 Hz, 1H, H_4), 3.59 (dd, *J* = 6.8, 3.7 Hz, 1H, H_9), 2.40 (s, 3H, H_1), 2.18 (brs, 1H, OH), 1.90 – 1.79 (m, 1H, H_{10}), 1.00 (d, *J* = 6.7 Hz, 3H, H_{11}), 0.86 (d, *J* = 6.8 Hz, 3H, H_{11}).

^{13}C NMR (126 MHz, $CDCl_3$) δ 201.3 (C), 139.8 (C), 136.7 (C), 134.7 (2 × CH), 130.2 (2 × CH), 128.73 (2 × CH), 128.69 (2 × CH), 128.5 (CH), 123.6 (C), 85.5 (CH), 78.1 (CH), 74.3 (CH_2), 30.7 (CH), 21.5 (CH_3), 19.5 (CH_3), 17.9 (CH_3).

Minor diastereomer (detectable non-overlapping resonances):

^{1}H NMR (500 MHz, $CDCl_3$) δ 4.07 (d, *J* = 5.9 Hz, 1H, H_4), 3.70 (dd, *J* = 5.7, 5.7 Hz, 1H, H_9), 0.92 (d, *J* = 6.7 Hz, 3H, H_{11}).

^{13}C NMR (126 MHz, $CDCl_3$) δ 85.8 (CH), 29.3 (CH), 19.7 (CH_3), 16.7 (CH_3).

SFC (OJ-H, CO_2/CH_3OH = 80:20, 2.0 mL/min, 298 K, 254 nm): t_R(*syn*, minor) = 2.9 min, t_R(*syn*, major) = 3.3 min, t_R(*anti*, major) = 4.9 min, t_R(*anti*, minor) = 5.4 min, d.r. (*syn:anti*) = 17:1, e.r.(*syn*) = 96:4 (93% *ee*), e.r.(*anti*) = 89:11 (77% *ee*).

HRMS-CI (m/z): $[M + Na]^+$ calcd for $C_{20}H_{24}O_3SNa$, 367.1338; found, 367.1322.

R_f = 0.52 (20% ethyl acetate-hexane; UV).

Synthesis of S-(p-tolyl) (2R,3S)-2-(benzyloxy)-3-hydroxyoctanethioate **10c**

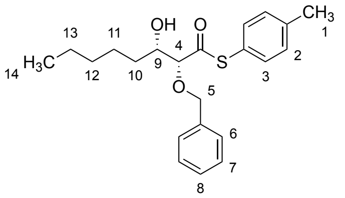

Following the general method, (*R*,*S*)-**10c** was prepared from hexanal (37 μL, 0.30 mmol, 1 equiv) and MAHT **8** (114 mg, 0.36 mmol, 1.20 equiv). The reaction mixture was stirred for 24 h. The residue was purified by column chromatography (eluting with 5% ethyl acetate–hexane initially, grading to 30% ethyl acetate–hexane). The product was obtained as a white solid (103 mg, 92%).

Major diastereomer:

^{1}H NMR (500 MHz, $CDCl_3$) δ 7.39 – 7.24 (m, 5H, H_{6-8}), 7.22 – 7.19 (m, 2H, H_2), 7.16 – 7.13 (m, 2H, H_3), 4.86 (d, *J* = 11.2 Hz, 1H, H_5), 4.47 (d, *J* = 11.2 Hz, 1H, H_5), 3.87 (d, *J* = 4.5 Hz, 1H, H_4), 3.77 (ddd, *J* = 7.8, 4.6, 4.5 Hz, 1H, H_9), 2.29 (s, 3H, H_1), 2.13 (brs, 1H, OH), 1.47 – 1.32 (m, 3H, $H_{10,11}$), 1.25 – 1.10 (m, 5H, H_{11-13}), 0.78 (t, *J* = 7.0 Hz, 3H, H_{14}).

^{13}C NMR (126 MHz, $CDCl_3$) δ 201.3 (C), 139.9 (C), 136.6 (C), 134.7 (2 × CH), 130.2 (2 × CH), 128.7 (2 × CH), 128.6 (2 × CH), 128.4 (CH), 123.6 (C), 87.2 (CH), 74.4 (CH_2), 72.9 (CH), 33.0 (CH_2), 31.7 (CH_2), 25.2 (CH_2), 22.6 (CH_2), 21.5 (CH_3), 14.1 (CH_3).

Minor diastereomer (detectable non-overlapping resonances):

^{1}H NMR (500 MHz, $CDCl_3$) δ 4.51 (d, *J* = 11.3 Hz, 1H, H_5), 3.96 (d, *J* = 4.8 Hz, 1H, H_4), 3.82 (ddd, *J* = 8.8, 4.8, 3.6 Hz, 1H, H_9).

^{13}C NMR (126 MHz, $CDCl_3$) δ 201.1 (C), 136.9 (C), 134.7 (2 × CH), 128.5 (2 × CH), 128.4 (CH), 123.6 (C), 87.7 (CH), 74.7 (CH_2), 73.0 (CH), 32.1 (CH_2), 31.8 (CH_2), 25.3 (CH_2), 22.7 (CH_2).

SFC (IC-3, CO_2/CH_3OH = 80:20, 2.0 mL/min, 298 K, 254 nm): t_R(*anti*, major) = 1.6 min, t_R(*anti*, minor) = 1.8 min, t_R(*syn*, major) = 2.1 min, t_R(*syn*, minor) = 2.4 min, d.r. (*syn:anti*) = 3:1, e.r.(*syn*) = 98:2 (95% *ee*), e.r.(*anti*) = 83:17 (66% *ee*).

HRMS-CI (m/z): $[M + H]^+$ calcd for $C_{22}H_{29}O_3S$, 373.1832; found, 373.1815.

R_f = 0.42 (10% ethyl acetate-hexane; UV).

Synthesis of S-(p-tolyl) (2R,3S)-2-(benzyloxy)-3-hydroxy-5-methylhexanethioate **10d**

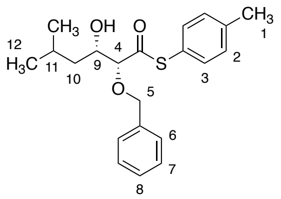

Following the general method, (*R*,*S*)-**10d** was prepared from isovaleraldehyde (33 μL, 0.30 mmol, 1 equiv) and MAHT **8** (114 mg, 0.36 mmol, 1.20 equiv). The reaction mixture was stirred for 48 h. The residue was purified by column chromatography (eluting with 5% ethyl acetate–hexane initially, grading to 30% ethyl acetate–hexane). The product was obtained as a colorless oil (98 mg, 91%).

Major diastereomer:

^{1}H NMR (500 MHz, $CDCl_3$) δ 7.39 – 7.23 (m, 5H, H_{6-8}), 7.21 (d, *J* = 8.1 Hz, 2H, H_2), 7.15 (d, *J* = 8.1 Hz, 2H, H_3), 4.86 (d, *J* = 11.1 Hz, 1H, H_5), 4.48 (d, *J* = 11.1 Hz, 1H, H_5), 3.99 – 3.85 (m, 1H, H_9), 3.83 (d, *J* = 4.4 Hz, 1H, H_4), 2.29 (s, 3H, H_1), 2.13 (brs, 1H, OH), 1.72 – 1.60 (m, 1H, H_{11}), 1.38 (ddd, *J* = 14.2, 9.7, 5.2 Hz, 1H, H_{10}), 1.18 (ddd, *J* = 14.2, 9.0, 3.6 Hz, 1H, H_{10}), 0.82 (d, *J* = 6.7 Hz, 3H, H_{12}), 0.78 (d, *J* = 6.6 Hz, 3H, H_{12}).

^{13}C NMR (126 MHz, $CDCl_3$) δ 201.3 (C), 139.8 (C), 136.6 (C), 134.7 (2 × CH), 130.2 (2 × CH), 128.7 (2 × CH), 128.6 (2 × CH), 128.5 (CH), 123.5 (C), 87.6 (CH), 74.4 (CH_2), 71.1 (CH), 41.8 (CH_2), 24.5 (CH), 23.5 (CH_3), 21.8 (CH_3), 21.4 (CH_3).

Minor diastereomer (detectable non-overlapping resonances):

^{1}H NMR (500 MHz, $CDCl_3$) δ 4.52 (d, *J* = 11.2 Hz, 1H, H_5), 1.26 (ddd, *J* = 14.2, 9.5, 3.1 Hz, 1H, H_{10}).

^{13}C NMR (126 MHz, $CDCl_3$) δ 200.9 (C), 136.9 (C), 128.42 (CH), 128.38 (2 × CH), 123.6 (C), 88.0 (CH), 74.7 (CH_2), 71.2 (CH), 40.9 (CH_2), 24.4 (CH), 23.7 (CH_3), 21.6 (CH_3).

SFC (IA-3, CO_2/CH_3OH = 90:10, 2.0 mL/min, 298 K, 254 nm): t_R(*syn*, major) = 2.5 min, t_R(*anti*, major) = 2.8 min, t_R(*syn*, minor) = 3.3 min, t_R(*anti*, minor) = 4.1 min, d.r. (*syn:anti*) = 4:1, e.r.(*syn*) = 98:2 (96% *ee*), e.r.(*anti*) = 86:14 (71% *ee*).

HRMS-CI (m/z): $[M + H]^+$ calcd for $C_{21}H_{27}O_3S$, 359.1675; found, 359.1655.

R_f = 0.70 (20% ethyl acetate-hexane; UV).

Synthesis of S-(p-tolyl) (2R,3S)-2-(benzyloxy)-3-cyclohexyl-3-hydroxypropanethioate **10e**

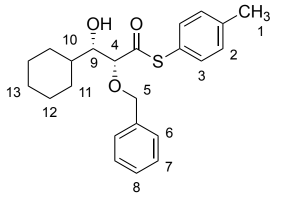

Following the general method, (*R*,*S*)-**10e** was prepared from cyclohexanecarboxaldehyde (36 µL, 0.30 mmol, 1 equiv) and MAHT **8** (114 mg, 0.36 mmol, 1.20 equiv). The reaction mixture was stirred for 24 h. The residue was purified by column chromatography (eluting with 5% ethyl acetate–hexane initially, grading to 50% ethyl acetate–hexane). The product was obtained as a colorless oil (107 mg, 92%).

Major diastereomer:

^{1}H NMR (500 MHz, $CDCl_3$) δ 7.37 – 7.33 (m, 2H, H_6), 7.34 – 7.28 (m, 2H, H_7), 7.28 – 7.24 (m, 1H, H_8), 7.24 – 7.19 (m, 2H, H_2), 7.17 – 7.12 (m, 2H, H_3), 4.89 (d, *J* = 11.2 Hz, 1H, H_5), 4.45 (d, *J* = 11.2 Hz, 1H, H_5), 4.04 (d, *J* = 3.4 Hz, 1H, H_4), 3.49 (dd, *J* = 7.3, 3.4 Hz, 1H, H_9), 2.29 (s, 3H, H_1), 2.02 (brs, 1H, OH), 1.89 – 1.81 (m, 1H, $H_{11/12/13}$), 1.68 – 1.62 (m, 1H, $H_{11/12/13}$), 1.60 – 1.50 (m, 2H, $H_{11/12/13}$), 1.45 – 1.35 (m, 1H, H_{10}), 1.30 – 1.21 (m, 1H, $H_{11/12/13}$), 1.14 – 0.85 (m, 5H, $H_{11/12/13}$).

^{13}C NMR (126 MHz, $CDCl_3$) δ 201.6 (C), 139.8 (C), 136.7 (C), 134.8 (2 × CH), 130.2 (2 × CH), 128.72 (2 × CH), 128.71 (2 × CH), 128.5 (CH), 123.7 (C), 84.7 (CH), 77.4 (CH), 74.3 (CH_2), 40.2 (CH), 29.5 (CH_2), 28.3 (CH_2), 26.3 (CH_2), 26.1 (CH_2), 26.0 (CH_2), 21.5 (CH_3).

Minor diastereomer (detectable non-overlapping resonances):

^{1}H NMR (500 MHz, $CDCl_3$) δ 4.86 (d, *J* = 11.3 Hz, 1H, H_5), 4.50 (d, *J* = 11.3 Hz, 1H, H_5), 4.00 (d, *J* = 5.7 Hz, 1H, H_4), 3.60 (dd, *J* = 5.7, 5.5 Hz, 1H, H_9).

^{13}C NMR (126 MHz, $CDCl_3$) δ 85.2 (CH), 40.2 (CH), 29.8 (CH_2), 27.2 (CH_2), 26.4 (CH_2).

SFC (IC-3, CO_2/CH_3OH = 80:20, 2.0 mL/min, 298 K, 254 nm): t_R(*anti*, major) = 2.1 min, t_R(*anti*, minor) = 2.6 min, t_R(*syn*, major) = 3.0 min, t_R(*syn*, minor) = 3.6 min, d.r. (*syn:anti*) = 16:1, e.r.(*syn*) = 93:7 (85% *ee*), e.r.(*anti*) = 88:12 (76% *ee*).

HRMS-CI (m/z): $[M + H]^+$ calcd for $C_{23}H_{29}O_3S$, 385.1832; found, 385.1811.

R_f = 0.70 (20% ethyl acetate-hexane; UV).

The absolute stereochemistry for (2*R*,3*S*)-**10e** was confirmed by x-ray crystallography (see *Catalog of X-ray data*). The crystal was grown by a slow evaporation of dichloromethane.

Synthesis of S-(p-tolyl) (2R,3S)-2-(benzyloxy)-6-chloro-3-hydroxyhexanethioate **10f**

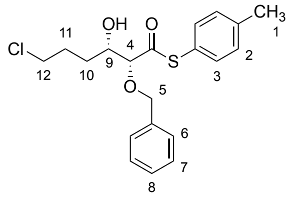

Following the general method, (*R*,*S*)-**10f** was prepared from 4-chlorobutanal (31 µL, 0.30 mmol, 1 equiv) and MAHT **8** (114 mg, 0.36 mmol, 1.20 equiv). The reaction mixture was stirred for 24 h. The residue was purified by column chromatography (eluting with 5% ethyl acetate–hexane initially, grading to 30% ethyl acetate–hexane). The product was obtained as a colorless oil (107 mg, 94%).

Major diastereomer:

^{1}H NMR (500 MHz, $CDCl_3$) δ 7.39 – 7.23 (m, 5H, H_{6-8}), 7.20 (d, *J* = 8.0 Hz, 2H, H_2), 7.14 (d, *J* = 8.0 Hz, 2H, H_3), 4.86 (d, *J* = 11.1 Hz, 1H, H_5), 4.47 (d, *J* = 11.1 Hz, 1H, H_5), 3.85 (d, *J* = 4.5 Hz, 1H, H_4), 3.78 (ddd, *J* = 8.6, 4.5, 4.1 Hz, 1H, H_9), 3.47 – 3.35 (m, 2H, H_{12}), 2.28 (s, 3H, H_1), 1.91 – 1.80 (m, 1H, H_{11}), 1.76 – 1.44 (m, 3H, $H_{10,11}$).

^{13}C NMR (126 MHz, $CDCl_3$) δ 201.1 (C), 140.0 (C), 136.5 (C), 134.7 (2 × CH), 130.3 (2 × CH), 128.8 (2 × CH), 128.7 (2 × CH), 128.4 (CH), 123.3 (C), 87.1 (CH), 74.5 (CH_2), 72.1 (CH), 44.9 (CH_2), 30.2 (CH_2), 28.7 (CH_2), 21.5 (CH_3).

Minor diastereomer (detectable non-overlapping resonances):

^{1}H NMR (500 MHz, $CDCl_3$) δ 4.50 (d, *J* = 11.3 Hz, 1H, H_5), 3.94 (d, *J* = 5.0 Hz, 1H, H_4).

^{13}C NMR (126 MHz, $CDCl_3$) δ 201.2 (C), 136.7 (C), 128.6 (2 × CH), 128.5 (CH), 123.3 (C), 87.4 (CH), 74.8 (CH_2), 72.2 (CH), 45.0 (CH_2), 29.4 (CH_2), 28.8 (CH_2).

SFC (IC-3, CO_2/CH_3OH = 90:10, 2.0 mL/min, 298 K, 254 nm): t_R(*anti*, major) = 3.1 min, t_R(*anti*, minor) = 3.6 min, t_R(*syn*, minor) = 5.1 min, t_R(*syn*, major) = 5.9 min, d.r. (*syn:anti*) = 3:1, e.r.(*syn*) = 98:2 (95% *ee*), e.r.(*anti*) = 86:14 (71% *ee*).

HRMS-CI (m/z): $[M + Na]^+$ calcd for $C_{20}H_{23}ClO_3SNa$, 401.0949; found, 401.0930.

R_f = 0.50 (20% ethyl acetate-hexane; UV).

Synthesis of S-(p-tolyl) (2R,3S)-2-(benzyloxy)-3-hydroxyhept-6-ynethioate **10g**

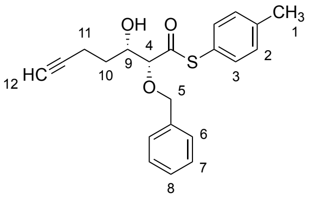

Following the general method, (*R*,*S*)-**10g** was prepared from pent-4-ynal (27 μL, 0.30 mmol, 1 equiv) and MAHT **8** (114 mg, 0.36 mmol, 1.20 equiv). The reaction mixture was stirred for 24 h. The residue was purified by column chromatography (eluting with 5% ethyl acetate–hexane initially, grading to 30% ethyl acetate–hexane). The product was obtained as a white solid (102 mg, 96%).

Major diastereomer:

^{1}H NMR (500 MHz, $CDCl_3$) δ 7.46 – 7.30 (m, 5H, H_{6-8}), 7.27 (d, *J* = 8.0 Hz, 2H, H_2), 7.21 (d, *J* = 8.0 Hz, 2H, H_3), 4.92 (d, *J* = 11.2 Hz, 1H, H_5), 4.55 (d, *J* = 11.2 Hz, 1H, H_5), 4.00 (ddd, *J* = 7.8, 5.0, 4.4 Hz, 1H, H_9), 3.95 (d, *J* = 4.4 Hz, 1H, H_4), 2.35 (s, 3H, H_1), 2.33 – 2.24 (m, 2H, H_{11}), 1.92 (t, *J* = 2.7 Hz, 1H, H_{12}), 1.74 – 1.64 (m, 2H, H_{10}).

^{13}C NMR (126 MHz, $CDCl_3$) δ 201.1 (C), 139.9 (C), 136.5 (C), 134.7 (2 × CH), 130.2 (2 × CH), 128.7 (2 × CH), 128.6 (2 × CH), 128.3 (CH), 123.3 (C), 86.9 (CH), 83.6 (C), 74.4 (CH_2), 71.4 (CH), 69.0 (CH), 31.7 (CH_2), 21.4 (CH_3), 14.8 (CH_2).

Minor diastereomer (detectable non-overlapping resonances):

^{1}H NMR (500 MHz, $CDCl_3$) δ 4.59 (d, *J* = 11.3 Hz, 1H, H_5).

^{13}C NMR (126 MHz, $CDCl_3$) δ 200.8 (C), 136.7 (C), 128.7 (2 × CH), 128.4 (2 × CH), 128.3 (CH), 123.4 (C), 87.3 (CH), 83.7 (C), 74.6 (CH_2), 71.5 (CH_2), 30.7 (CH_2).

SFC (IA-3, CO_2/CH_3OH = 94:6, 2.0 mL/min, 298 K, 254 nm): t_R(*syn*, major) = 6.2 min, t_R(*anti*, minor) = 6.9 min, t_R(*anti*, major) = 9.4 min, t_R(*syn*, minor) = 11.6 min, d.r. (*syn:anti*) = 4:1, e.r.(*syn*) = 98:2 (96% *ee*), e.r.(*anti*) = 84:16 (68% *ee*).

HRMS-CI (m/z): $[M + Na]^+$ calcd for $C_{21}H_{22}O_3SNa$, 377.1182; found, 377.1166.

R_f = 0.30 (20% ethyl acetate-hexane; UV).

Synthesis of S-(p-tolyl) (2R,3S)-2-(benzyloxy)-3-hydroxyhex-5-enethioate **10h**

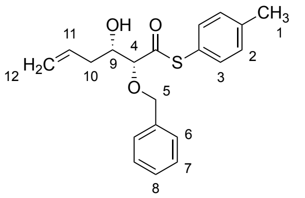

Following the general method, (*R*,*S*)-**10h** was prepared from but-3-enal (21 mg, 0.30 mmol, 1 equiv) and MAHT **8** (114 mg, 0.36 mmol, 1.20 equiv). The reaction mixture was stirred for 24 h. The residue was purified by column chromatography (eluting with 5% ethyl acetate–hexane initially, grading to 30% ethyl acetate–hexane). The product was obtained as a colorless oil (69 mg, 67%).

Major diastereomer:

^{1}H NMR (500 MHz, $CDCl_3$) δ 7.49 – 7.35 (m, 5H, H_{6-8}), 7.33 – 7.28 (m, 2H, H_2), 7.27 – 7.23 (m, 2H, H_3), 5.85 – 5.72 (m, 1H, H_{11}), 5.12 – 5.05 (m, 2H, H_{12}), 4.98 (d, *J* = 11.1 Hz, 1H, H_5), 4.58 (d, *J* = 11.1 Hz, 1H, H_5), 4.02 (d, *J* = 4.1 Hz, 1H, H_4), 3.97 (ddd, *J* = 7.4, 5.6, 4.1 Hz, 1H, H_9), 2.39 (s, 3H, H_1), 2.37 – 2.30 (m, 2H, H_{10}), 1.98 (brs, 1H, OH).

^{13}C NMR (126 MHz, $CDCl_3$) δ 201.2 (C), 140.0 (C), 136.6 (C), 134.8 (2 × CH), 133.9 (CH), 130.3 (2 × CH), 128.8 (2 × CH), 128.7 (2 × CH), 128.6 (CH), 123.6 (C), 118.4 (CH_2), 86.3 (CH), 74.6 (CH_2), 72.4 (CH), 37.8 (CH_2), 21.5 (CH_3).

Minor diastereomer (detectable non-overlapping resonances):

^{1}H NMR (500 MHz, $CDCl_3$) δ 5.19 – 5.12 (m, 2H, H_{12}), 4.64 (d, *J* = 11.2 Hz, 1H, H_5), 4.09 (d, *J* = 5.1 Hz, 1H, H_4).

^{13}C NMR (126 MHz, $CDCl_3$) δ 201.1 (C), 136.9 (C), 134.1 (2 × CH), 128.8 (2 × CH), 128.5 (CH), 128.4 (2 × CH), 118.7 (CH_2), 87.0 (CH), 74.7 (CH_2), 72.2 (CH), 36.8 (CH_2).

SFC (IC-3, CO_2/CH_3OH = 90:10, 2.0 mL/min, 298 K, 254 nm): t_R(*anti*, major) = 2.8 min, t_R(*anti*, minor) = 3.3 min, t_R(*syn*, major) = 4.0 min, t_R(*syn*, minor) = 4.6 min, d.r. (*syn:anti*) = 6:1, e.r.(*syn*) = 99:1 (99% *ee*), e.r.(*anti*) = 85:15 (70% *ee*).

HRMS-CI (m/z): $[M + Na]^+$ calcd for $C_{20}H_{22}O_3SNa$, 365.1182; found, 365.1168.

R_f = 0.33 (20% ethyl acetate-hexane; UV).

Synthesis of S-(p-tolyl) (2R,3S)-2-(benzyloxy)-3-hydroxy-4-(2,4,5-trifluorophenyl)butanethioate **10i**

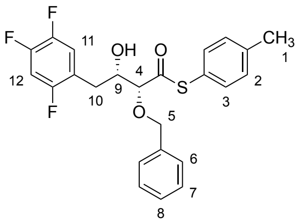

Following the general method, (*R*,*S*)-**10i** was prepared from 2-(2,4,5-trifluorophenyl)acetaldehyde (40 µL, 0.30 mmol, 1 equiv) and MAHT **8** (114 mg, 0.36 mmol, 1.20 equiv). The reaction mixture was stirred for 24 h. The residue was purified by column chromatography (eluting with 5% ethyl acetate–hexane initially, grading to 30% ethyl acetate–hexane). The product was obtained as a white solid (128 mg, 95%).

Major diastereomer:

^{1}H NMR (500 MHz, $CDCl_3$) δ 7.53 – 7.36 (m, 5H, $H_{6\text{-}8}$), 7.32 (d, *J* = 8.1 Hz, 2H, H_2), 7.26 (d, *J* = 8.1 Hz, 2H, H_3), 6.98 (ddd, *J* = 10.6, 8.8, 6.8 Hz, 1H, H_{12}), 6.89 (ddd, *J* = 10.1, 9.2, 6.6 Hz, 1H, H_{11}), 5.01 (d, *J* = 11.1 Hz, 1H, H_5), 4.60 (d, *J* = 11.1 Hz, 1H, H_5), 4.10 (ddd, *J* = 8.7, 4.2, 4.2 Hz, 1H, H_9), 4.02 (d, *J* = 4.2 Hz, 1H, H_4), 2.89 (dd, *J* = 14.2, 4.2 Hz, 1H, H_{10}), 2.73 (dd, *J* = 14.2, 8.7, 1H, H_{10}), 2.40 (s, 3H, H_1), 2.37 (brs, 1H, OH).

^{13}C NMR (126 MHz, $CDCl_3$) δ 201.2 (C), 156.2 (ddd, *J* = 244.5, 9.4, 2.8 Hz, CF), 150.2 – 147.7 (m, CF), 146.6 (ddd, *J* = 244.1, 12.4, 3.6 Hz, CF), 140.1 (C), 136.4 (C), 134.7 (2 × CH), 130.3 (2 × CH), 128.84 (2 × CH), 128.75 (CH), 128.7 (2 × CH), 123.3 (C), 121.2 (dt, *J* = 17.9, 5.0 Hz, C), 119.2 (dd, *J* = 19.1, 5.9 Hz, CH), 105.4 (dd, *J* = 28.6, 20.6 Hz, CH), 86.1 (CH), 74.6 (CH_2), 72.4 (CH), 32.1 (CH_2), 21.5 (CH_3).

^{19}F NMR (470 MHz, $CDCl_3$) δ -118.92 – -119.29 (m, 1F), -135.75 – -136.53 (m, 1F), -142.80 – -143.21 (m, 1F).

Minor diastereomer (detectable non-overlapping resonances):

^{1}H NMR (500 MHz, $CDCl_3$) δ 4.64 (d, *J* = 11.2 Hz, 1H, H_5), 4.16 (ddd, *J* = 9.0, 4.4, 4.4 Hz, 1H, H_9), 2.97 (dd, *J* = 14.5, 4.4 Hz, 1H, H_{10}), 2.81 (dd, *J* = 14.5, 9.0 Hz, 1H, H_{10}).

^{13}C NMR (126 MHz, $CDCl_3$) δ 200.9 (C), 136.6 (C), 128.8 (2 × CH), 128.6 (CH), 128.5 (2 × CH), 123.3 (C), 86.7 (CH), 74.8 (CH_2), 72.5 (CH).

SFC (IC-3, CO_2/CH_3OH = 94:6, 2.0 mL/min, 298 K, 254 nm): t_R(*anti*, major) = 4.4 min, t_R(*anti*, minor) = 5.2 min, t_R(*syn*, major) = 5.6 min, t_R(*syn*, minor) = 6.1 min, d.r. (*syn:anti*) = 13:1, e.r.(*syn*) = 99:1 (98% *ee*), e.r.(*anti*) = 96:4 (92% *ee*).

HRMS-CI (m/z): $[M + Na]^+$ calcd for $C_{24}H_{21}F_3O_3SNa$, 469.1056; found, 469.1056.

R_f = 0.53 (20% ethyl acetate-hexane; UV).

Synthesis of S-(p-tolyl) (2R,3S)-2-(benzyloxy)-3-hydroxy-4,4-diphenylbutanethioate **10j**

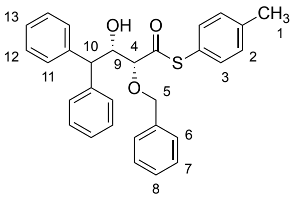

Following the general method, (*R*,*S*)-**10j** was prepared from diphenylacetaldehyde (53 µL, 0.30 mmol, 1 equiv) and MAHT **8** (114 mg, 0.36 mmol, 1.20 equiv) in toluene (0.40 M, 0.75 mL). The reaction mixture was stirred for 48 h. The residue was purified by column chromatography (eluting with 5% ethyl acetate–hexane initially, grading to 30% ethyl acetate–hexane). The product was obtained as a colorless oil (98 mg, 70%).

Major diastereomer:

^{1}H NMR (500 MHz, $CDCl_3$) δ 7.50 – 7.11 (m, 19H, $H_{2,3,6\text{-}8,11\text{-}13}$), 4.83 (d, *J* = 10.7 Hz, 1H, H_5), 4.78 (dd, *J* = 9.9, 2.3 Hz, 1H, H_9), 4.27 – 4.18 (m, 2H, $H_{5,10}$), 3.96 (d, *J* = 2.3 Hz, 1H, H_4), 2.35 (s, 3H, H_1), 2.07 (brs, 1H, OH).

^{13}C NMR (126 MHz, $CDCl_3$) δ 201.5 (C), 141.5 (C), 141.1 (C), 139.8 (C), 136.8 (C), 134.8 (2 × CH), 130.2 (2 × CH), 129.1 (2 × CH), 128.8 (2 × CH), 128.7 (4 × CH), 128.6 (2 × CH), 128.5 (CH), 128.3 (2 × CH), 127.1 (CH), 126.9 (CH), 123.7 (C), 84.8 (CH), 75.3 (CH_2), 74.9 (CH), 54.9 (CH), 21.4 (CH_3).

Minor diastereomer (detectable non-overlapping resonances):

^{1}H NMR (500 MHz, $CDCl_3$) δ 4.67 (dd, *J* = 7.9, 4.5 Hz, 1H, H_9), 4.43 (d, *J* = 7.9 Hz, 1H, H_{10}), 4.38 (d, *J* = 11.3 Hz, 1H, H_5), 3.93 (d, *J* = 4.5 Hz, 1H, H_4).

^{13}C NMR (126 MHz, $CDCl_3$) δ 201.2 (C), 141.5 (C), 140.4 (C), 134.9 (2 × CH), 129.2 (2 × CH), 128.8 (2 × CH), 128.7 (2 × CH), 128.5 (CH), 128.4 (2 × CH), 127.0 (CH), 126.7 (CH), 123.9 (C), 85.1 (CH), 75.7 (CH_2), 74.1 (CH), 52.7 (CH).

SFC (IA-3, CO_2/CH_3OH = 80:20, 2.0 mL/min, 298 K, 254 nm): t_R(*syn*, minor) = 3.2 min, t_R(*syn*, major) = 3.6 min, t_R(*anti*, major) = 4.3 min, t_R(*anti*, minor) = 5.5 min, d.r. (*syn:anti*) = 9:1, e.r.(*syn*) = 86:14 (73% *ee*), e.r.(*anti*) = 77:23 (54% *ee*).

HRMS-CI (m/z): $[M + Na]^+$ calcd for $C_{30}H_{28}O_3SNa$, 491.1651; found, 491.1654.

R_f = 0.18 (10% ethyl acetate-hexane; UV).

Synthesis of S-(p-tolyl) (2R,3S)-2-(benzyloxy)-3-hydroxydec-4-ynethioate **10k**

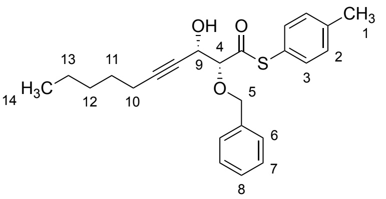

Following the general method, (*R*,*S*)-**10k** was prepared from 2-octynal (43 µL, 0.30 mmol, 1 equiv) and MAHT **8** (114 mg, 0.36 mmol, 1.20 equiv). The reaction mixture was stirred for 24 h. The residue was purified by column chromatography (eluting with 5% ethyl acetate–hexane initially, grading to 30% ethyl acetate–hexane). The product was obtained as a colorless oil (111 mg, 93%).

Major diastereomer:

^{1}H NMR (500 MHz, $CDCl_3$) δ 7.42 – 7.24 (m, 5H, $H_{6\text{-}8}$), 7.19 (d, *J* = 8.2 Hz, 2H, H_2), 7.15 – 7.11 (m, 2H, H_3), 4.88 (d, *J* = 11.3 Hz, 1H, H_5), 4.72 (d, *J* = 11.3 Hz, 1H, H_5), 4.61 – 4.56 (m, 1H, H_9), 4.04 (d, *J* = 3.5 Hz, 1H, H_4), 2.44 (brs, 1H, OH), 2.28 (s, 3H, H_1), 2.17 – 2.04 (m, 2H, H_{10}), 1.46 – 1.39 (m, 2H, H_{11}), 1.33 – 1.12 (m, 4H, $H_{12,13}$), 0.85 – 0.73 (m, 3H, H_{14}).

^{13}C NMR (126 MHz, $CDCl_3$) δ 200.0 (C), 139.9 (C), 136.6 (C), 134.7 (2 × CH), 130.2 (2 × CH), 128.73 (CH), 128.69 (2 × CH), 128.6 (2 × CH), 123.6 (C), 87.6 (C), 86.5 (CH), 77.5 (C), 75.1 (CH_2), 63.8 (CH), 31.1 (CH_2), 28.3 (CH_2), 22.3 (CH_2), 21.5 (CH_3), 18.8 (CH_2), 14.1 (CH_3).

Minor diastereomer (detectable non-overlapping resonances):

^{1}H NMR (500 MHz, $CDCl_3$) δ 4.93 (d, *J* = 11.4 Hz, 1H, H_5), 4.66 (d, *J* = 11.4 Hz, 1H, H_5), 4.11 (d, *J* = 4.7 Hz, 1H, H_4).

^{13}C NMR (126 MHz, $CDCl_3$) δ 199.7 (C), 136.8 (C), 128.5 (2 × CH), 128.4 (2 × CH), 123.5 (C), 88.3 (C), 86.7 (CH), 76.4 (C), 75.0 (CH_2), 64.1 (CH), 31.1 (CH_2), 18.9 (CH_2).

SFC (OD-H, CO_2/CH_3OH = 94:6, 2.0 mL/min, 298 K, 254 nm): t_R(*syn*, major) = 7.1 min, t_R(*syn*, minor) = 7.9 min, t_R(*anti*, minor) = 8.7 min, t_R(*anti*, major) = 10.7 min, d.r. (*syn:anti*) = 4:1, e.r.(*syn*) = 95:5 (91% *ee*), e.r.(*anti*) = 69:31 (37% *ee*).

HRMS-CI (m/z): $[M + Na]^+$ calcd for $C_{24}H_{28}O_3SNa$, 419.1651; found, 419.1631.

R_f = 0.51 (20% ethyl acetate-hexane; UV).

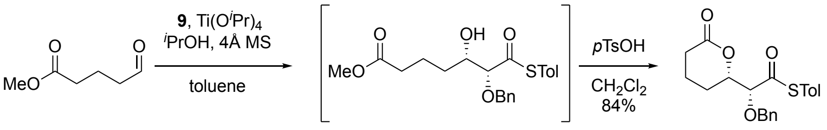

10l

Scheme S2. *Synthesis of acetate* ***10l***.

Synthesis of S-(p-tolyl) (R)-2-(benzyloxy)-2-((S)-6-oxotetrahydro-2H-pyran-2-yl)ethanethioate **10l**

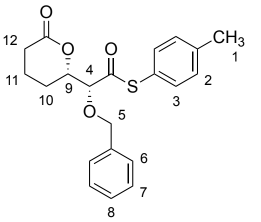

Following the general method, (*R*,*S*)-**10l** was prepared from methyl 5-oxopentanoate (78 µL, 0.60 mmol, 1 equiv) and MAHT **8** (228 mg, 0.72 mmol, 1.20 equiv). The reaction mixture was stirred for 24 h. The product mixture was filtered through celite and rinsed with dichloromethane. The filtrate was concentrated and the residue was redissolved in dichloromethane (0.05 M, 12.0 mL). *p*-Toluenesulfonic acid (23 mg, 0.12 mmol, 0.20 equiv) was added and the reaction mixture was stirred for 60 h at 40 °C. The product mixture was cooled down to 23 °C and diluted with ice-cold water and dichloromethane. The organic layer was separated and the aqueous layer was extracted with dichloromethane. The combined organic layers were dried over sodium sulfate, filtered, and the filtrate was concentrated. The residue was purified by column chromatography (eluting with 5% ethyl acetate–hexane initially, grading to 40% ethyl acetate–hexane). The product was obtained as a white solid a single diastereomer (186 mg, 84%).

^{1}H NMR (500 MHz, $CDCl_3$) δ 7.49 – 7.34 (m, 5H, $H_{2,6\text{-}7}$), 7.32 (d, *J* = 8.1 Hz, 2H, H_2), 7.24 (d, *J* = 7.9 Hz, 2H, H_3), 5.01 (d, *J* = 11.4 Hz, 1H, H_5), 4.70 – 4.60 (m, 2H, $H_{5,9}$), 4.06 (d, *J* = 3.7 Hz, 1H, H_4), 2.61 – 2.50 (m, 1H, H_{12}), 2.48 – 2.40 (m, 1H, H_{12}), 2.38 (s, 3H, H_1), 1.96 – 1.88 (m, 1H, H_{11}), 1.81 – 1.72 (m, 3H, $H_{10,11}$).

^{13}C NMR (126 MHz, $CDCl_3$) δ 199.8 (C), 170.3 (C), 139.9 (C), 136.4 (C), 134.7 (2 × CH), 130.2 (2 × CH), 128.74 (2 × CH), 128.69 (2 × CH), 128.5 (CH), 123.4 (C), 85.4 (CH), 80.0 (CH), 74.9 (CH_2), 29.7 (CH_2), 23.9 (CH_2), 21.5 (CH_3), 18.3 (CH_2).

SFC (IC-3, CO_2/CH_3OH = 80:20, 2.0 mL/min, 298 K, 254 nm): t_R(*syn*, major) = 4.9 min, t_R(*syn*, minor) = 5.7 min, e.r. = 99:1 (97% *ee*).

HRMS-CI (m/z): $[M + Na]^+$ calcd for $C_{21}H_{22}O_4SNa$, 393.1131; found, 393.1110.

R_f = 0.15 (20% ethyl acetate-hexane; UV).

$[\alpha]_D^{25}$ = +110.39 (c 0.5, $CHCl_3$)

Synthesis of S-(p-tolyl) (2R,3S)-2-(benzyloxy)-5,5-diethoxy-3-hydroxypentanethioate **10m**

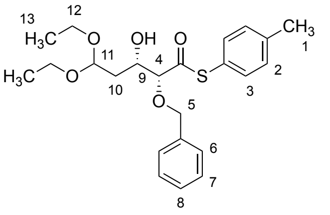

Following the general method, (*R*,*S*)-**10m** was prepared from 3,3-diethoxypropanal (47 mg, 0.30 mmol, 1 equiv), MAHT **8** (114 mg, 0.36 mmol, 1.20 equiv) and titanium (IV) isopropoxide (0.50 M in toluene, 90 µL, 0.05 mmol, 15 mol%). The reaction mixture was stirred for 24 h. The residue was purified by column chromatography (eluting with 5% ethyl acetate–hexane initially, grading to 30% ethyl acetate–hexane). The product was obtained as a colorless oil (103 mg, 82%).

Major diastereomer:

^{1}H NMR (500 MHz, $CDCl_3$) δ 7.47 – 7.27 (m, 7H, $H_{2,6\text{-}8}$), 7.24 – 7.19 (m, 2H, H_3), 4.94 (d, *J* = 11.3 Hz, 1H, H_5), 4.67 (dd, *J* = 6.5, 4.7 Hz, 1H, H_{11}), 4.57 (d, *J* = 11.3 Hz, 1H, H_5), 4.15 – 4.05 (m, 1H, H_9), 3.96 (d, *J* = 3.9 Hz, 1H, H_4), 3.67 – 3.57 (m, 2H, H_{12}), 3.51 – 3.41 (m, 2H, H_{12}), 2.35 (s, 3H, H_1), 1.91 – 1.83 (m, 1H, H_{10}), 1.75 (ddd, *J* = 14.2, 6.5, 3.0 Hz, 1H, H_{10}), 1.32 – 0.99 (m, 6H, H_{13}).

^{13}C NMR (126 MHz, $CDCl_3$) δ 201.0 (C), 139.8 (C), 136.7 (C), 134.7 (2 × CH), 130.2 (2 × CH), 128.7 (2 × CH), 128.6 (2 × CH), 128.4 (CH), 123.7 (C), 101.2 (CH), 87.0 (CH), 74.5 (CH_2), 69.9 (CH), 62.02 (CH_2), 61.97 (CH_2), 37.1 (CH_2), 21.4 (CH_2), 15.41 (CH_3), 15.38 (CH_3).

Minor diastereomer (detectable non-overlapping resonances):

^{1}H NMR (500 MHz, $CDCl_3$) δ 4.90 (d, *J* = 11.1 Hz, 1H, H_5), 4.72 (appt, *J* = 5.6 Hz, 1H, H_{11}).

^{13}C NMR (126 MHz, $CDCl_3$) δ 200.3 (C), 137.1 (C), 134.7 (2 × CH), 128.6 (2 × CH), 128.3 (2 × CH), 128.2 (2 × CH), 123.8 (CH), 101.8 (CH), 87.6 (CH), 74.6 (CH_2), 70.3 (CH), 62.5 (CH_2), 61.7 (CH_2), 35.5 (CH_2).

SFC (IG-3, CO_2/CH_3OH = 90:10, 2.0 mL/min, 298 K, 254 nm): t_R(*syn*, major) = 6.4 min, t_R(*anti*, major) = 7.0 min, t_R(*syn*, minor) = 8.9 min, t_R(*anti*, minor) = 18.0 min, d.r. (*syn:anti*) = 4:1, e.r.(*syn*) = 96:4 (92% *ee*), e.r.(*anti*) = 78:22 (57% *ee*).

HRMS-CI (m/z): $[M + Na]^+$ calcd for $C_{23}H_{30}O_5SNa$, 441.1706; found, 441.1700.

R_f = 0.35 (20% ethyl acetate-hexane; UV).

Synthesis of S-(p-tolyl) (2R,3S)-2,4-bis(benzyloxy)-3-hydroxybutanethioate **10n**

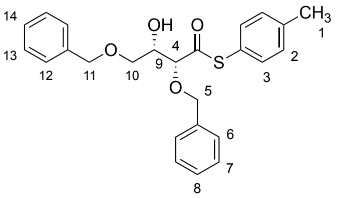

Following the general method, (*R*,*S*)-**10n** was prepared from benzyloxyacetaldehyde (42 µL, 0.30 mmol, 1 equiv), MAHT **8** (114 mg, 0.36 mmol, 1.20 equiv) and titanium (IV) isopropoxide (0.50 M in toluene, 90 µL, 0.05 mmol, 15 mol%). The reaction mixture was stirred for 24 h. The residue was purified by column chromatography (eluting with 5% ethyl acetate–hexane initially, grading to 40% ethyl acetate–hexane). The product was obtained as a colorless oil (116 mg, 92%).

Major diastereomer:

^{1}H NMR (500 MHz, $CDCl_3$) δ 7.49 – 7.27 (m, 12H, $H_{2,6\text{-}8,12\text{-}14}$), 7.26 – 7.21 (m, 2H, H_3), 4.97 (d, *J* = 11.0 Hz, 1H, H_5), 4.61 – 4.44 (m, 3H, $H_{5,11}$), 4.27 (d, *J* = 3.7 Hz, 1H, H_4), 4.20 – 4.15 (m, 1H, H_9), 3.61 (dd, *J* = 9.6, 5.4 Hz, 1H, H_{10}), 3.55 (dd, *J* = 9.6, 6.1 Hz, 1H, H_{10}), 2.44 (brs, 1H, OH), 2.40 (s, 3H, H_1).

^{13}C NMR (126 MHz, $CDCl_3$) δ 200.8 (C), 139.8 (C), 137.9 (C), 136.7 (C), 134.7 (2 × CH), 130.1 (2 × CH), 128.64 (2 × CH), 128.63 (2 × CH), 128.50 (2 × CH), 128.46 (CH), 127.92 (2 × CH), 127.85 (CH), 123.7 (C), 84.3 (CH), 74.8 (CH_2), 73.5 (CH_2), 71.6 (CH), 70.0 (CH_2), 21.4 (CH_3).

Minor diastereomer (detectable non-overlapping resonances):

^{1}H NMR (500 MHz, $CDCl_3$) δ 4.92 (d, *J* = 11.1 Hz, 1H, H_5), 4.64 (d, *J* = 11.1 Hz, 1H, H_5), 4.25 (d, *J* = 5.8 Hz, 1H, H_4), 3.71 – 3.69 (m, 2H, H_{10}).

^{13}C NMR (126 MHz, $CDCl_3$) δ 200.7 (C), 139.8 (C), 137.9 (C), 136.9 (C), 134.7 (2 × CH), 128.4 (2 × CH), 128.3 (2 × CH), 127.9 (CH), 123.6 (2 × CH), 84.9 (CH), 74.4 (CH_2), 73.5 (CH_2), 72.0 (CH), 69.9 (CH_2).

SFC (OJ-H, CO_2/CH_3OH = 80:20, 2.0 mL/min, 298 K, 254 nm): t_R(*syn*, minor) = 9.4 min, t_R(*anti*, major) = 11.2 min, t_R(*syn*, major) = 11.9 min, t_R(*anti*, minor) = 23.4 min, d.r. (*syn:anti*) = 4:1, e.r.(*syn*) = 92:8 (84% *ee*), e.r.(*anti*) = 88:12 (76% *ee*).

HRMS-CI (m/z): $[M + Na]^+$ calcd for $C_{25}H_{26}O_4SNa$, 445.1444; found, 445.1406.

R_f = 0.31 (20% ethyl acetate-hexane; UV).

Synthesis of S-(p-tolyl) (2R,3S)-2-(benzyloxy)-5-(((benzyloxy)carbonyl)amino)-3-hydroxypentanethioate **10o**

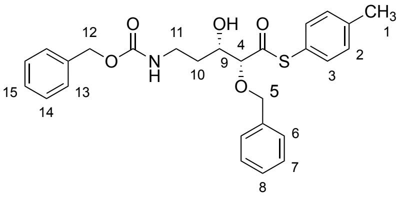

Following the general method, (*R*,*S*)-**10o** was prepared from benzyl (3-oxopropyl)carbamate (62 mg, 0.30 mmol, 1 equiv) and MAHT **8** (114 mg, 0.36 mmol, 1.20 equiv). The reaction mixture was stirred for 24 h. The residue was purified by column chromatography (eluting with 5% ethyl acetate–hexane initially, grading to 40% ethyl acetate–hexane). The product was obtained as a colorless oil (136 mg, 94%).

Major diastereomer:

^{1}H NMR (500 MHz, $CDCl_3$) δ 7.47 – 7.28 (m, 12H, $H_{2,6\text{-}8,13\text{-}15}$), 7.24 (d, *J* = 8.2 Hz, 2H, H_3), 5.21 – 5.14 (m, 1H, H_9), 5.08 (s, 2H, H_{12}), 4.96 (d, *J* = 11.0 Hz, 1H, H_5), 4.57 (d, *J* = 11.0 Hz, 1H, H_5), 3.99 – 3.90 (m, 1H, NH), 3.53 – 3.38 (m, 1H, H_{11}), 3.33 – 3.17 (m, 1H, H_{11}), 2.39 (s, 3H, H_1), 1.74 – 1.65 (m, 2H, H_{10}).

^{13}C NMR (126 MHz, $CDCl_3$) δ 201.0 (C), 156.8 (C), 139.9 (C), 136.6 (C), 136.5 (C), 134.7 (2 × CH), 130.2 (2 × CH), 128.7 (2 × CH), 128.63 (2 × CH), 128.58 (2 × CH), 128.55 (CH), 128.4 (CH), 128.2 (CH), 128.1 (CH), 123.4 (C), 87.2 (CH), 74.5 (CH_2), 71.1 (CH), 66.8 (CH_2), 38.1 (CH_2), 32.8 (CH_2), 21.4 (CH_3).

Minor diastereomer (detectable non-overlapping resonances):

^{1}H NMR (500 MHz, $CDCl_3$) δ 4.65 (d, *J* = 11.2 Hz, 1H, H_5).

^{13}C NMR (126 MHz, $CDCl_3$) δ 200.9 (C), 157.1 (C), 139.9 (C), 136.6 (C), 130.2 (2 × CH), 128.7 (2 × CH), 87.4 (CH_2), 74.7 (CH), 70.9 (CH_2), 32.1 (CH_2).

SFC (IC-3, CO_2/CH_3OH = 80:20, 2.0 mL/min, 298 K, 254 nm): t_R(*anti*, major) = 4.3 min, t_R(*anti*, major) = 5.2 min, t_R(*anti*, minor) = 6.9 min (*syn*, minor), t_R(*syn*, major) = 7.7 min, d.r. (*syn:anti*) = 4:1, e.r.(*syn*) = 96:4 (92% *ee*), e.r.(*anti*) = 83:17 (67% *ee*).

HRMS-CI (m/z): $[M + H]^+$ calcd for $C_{27}H_{30}NO_5S$, 480.1839; found, 480.1826.

R_f = 0.23 (30% ethyl acetate-hexane; UV).

Synthesis of S-(p-tolyl) (2R,3S)-2-(benzyloxy)-3-hydroxy-3-(4-nitrophenyl)propanethioate **10p**

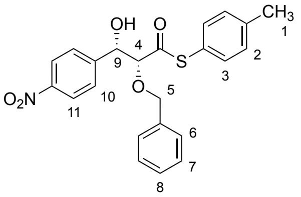

Following the general method, (*R*,*S*)-**10p** was prepared from 4-nitrobenzaldehyde (45 mg, 0.30 mmol, 1 equiv) and MAHT **8** (114 mg, 0.36 mmol, 1.20 equiv). The reaction mixture was stirred for 24 h. The residue was purified by column chromatography (eluting with 5% ethyl acetate–hexane initially, grading to 30% ethyl acetate–hexane). The product was obtained as a yellow solid (122 mg, 96%).

Major diastereomer:

^{1}H NMR (500 MHz, $CDCl_3$) δ 8.15 (d, *J* = 8.7 Hz, 2H, H_{11}), 7.48 (d, *J* = 8.7 Hz, 2H, H_{10}), 7.34 – 7.20 (m, 9H, $H_{2,3,6\text{-}8}$), 5.14 (d, *J* = 3.8 Hz, 1H, H_9), 4.86 (d, *J* = 11.3 Hz, 1H, H_5), 4.43 (d, *J* = 11.3 Hz, 1H, H_5), 4.20 (d, *J* = 3.8 Hz, 1H, H_4), 3.11 (brs, 1 H, OH), 2.39 (s, 3H, H_1).

^{13}C NMR (126 MHz, $CDCl_3$) δ 200.2 (C), 147.7 (C), 146.9 (C), 140.3 (C), 135.7 (C), 134.6 (2 × CH), 130.4 (2 × CH), 128.7 (4 × CH), 127.4 (3 × CH), 123.5 (2 × CH), 122.9 (C), 87.2 (CH), 74.8 (CH_2), 74.0 (CH), 21.5 (CH_3).

Minor diastereomer (detectable non-overlapping resonances):

^{1}H NMR (500 MHz, $CDCl_3$) δ 7.53 (d, *J* = 8.7 Hz, 1H, H_{10}), 5.06 (d, *J* = 6.4 Hz, 1H, H_9), 4.81 (d, *J* = 11.1 Hz, 1H, H_5), 4.37 (d, *J* = 11.1 Hz, 1H, H_5), 4.16 (d, *J* = 6.4 Hz, 1H, H_4).

^{13}C NMR (126 MHz, $CDCl_3$) δ 134.6 (2 × CH), 128.3 (2 × CH), 123.4 (2 × CH), 86.8 (CH), 75.1 (CH_2), 74.1 (CH).

SFC (IC-3, CO_2/CH_3OH = 80:20, 2.0 mL/min, 298 K, 254 nm): t_R(*anti*, major) = 2.6 min, t_R(*anti*, minor) = 3.1 min, t_R(*syn*, major) = 3.7 min, t_R(*syn*, minor) = 4.1 min, d.r. (*syn:anti*) = 31:1, e.r.(*syn*) = 99:1 (99% *ee*), e.r.(*anti*) = 89:11 (78% *ee*).

HRMS-CI (m/z): $[M + Na]^+$ calcd for $C_{23}H_{21}NO_5SNa$, 446.1033; found, 446.1010.

R_f = 0.30 (20% ethyl acetate-hexane; UV).

The absolute stereochemistry for (2*R*,3*S*)-**10p** was confirmed by x-ray crystallography (see *Catalog of X-ray data*). The crystal was grown by a slow evaporation of dichloromethane.

Synthesis of S-(p-tolyl) (2R,3S)-2-(benzyloxy)-3-(4-chlorophenyl)-3-hydroxypropanethioate **10q**

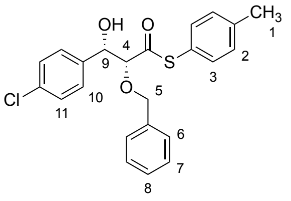

Following the general method, (*R*,*S*)-**10q** was prepared from 4-chlorobenzaldehyde (42 mg, 0.30 mmol, 1 equiv) and MAHT **8** (114 mg, 0.36 mmol, 1.20 equiv) in toluene (0.40 M, 0.75 mL). The reaction mixture was stirred for 48 h. The residue was purified by column chromatography (eluting with 5% ethyl acetate–hexane initially, grading to 30% ethyl acetate–hexane). The product was obtained as a white solid (94 mg, 76%).

Major diastereomer:

^{1}H NMR (500 MHz, $CDCl_3$) δ 7.33 – 7.20 (m, 13H, $H_{2,3,6\text{-}8,10,11}$), 5.01 (d, *J* = 4.0 Hz, 1H, H_9), 4.80 (d, *J* = 11.2 Hz, 1H, H_5), 4.40 (d, *J* = 11.2 Hz, 1H, H_5), 4.12 (d, *J* = 4.0 Hz, 1H, H_4), 2.67 (brs, 1H, OH), 2.37 (s, 3H, H_1).

^{13}C NMR (126 MHz, $CDCl_3$) δ 200.1 (C), 140.0 (C), 138.0 (C), 136.1 (C), 134.6 (2 × CH), 133.9 (C), 130.3 (2 × CH), 128.7 (2 × CH), 128.61 (2 × CH), 128.56 (2 × CH), 128.5 (CH), 127.9 (2 × CH), 123.3 (C), 87.9 (CH), 74.7 (CH_2), 74.2 (CH), 21.5 (CH_3).

Minor diastereomer (detectable non-overlapping resonances):

^{1}H NMR (500 MHz, $CDCl_3$) δ 4.90 (d, *J* = 6.6 Hz, 1H, H_9), 4.73 (d, *J* = 11.1 Hz, 1H, H_5), 4.32 (d, *J* = 11.1 Hz, 1H, H_5), 4.10 (d, *J* = 6.6 Hz, 1H, H_4).

^{13}C NMR (126 MHz, $CDCl_3$) δ 202.0 (C), 140.1 (C), 137.7 (C), 136.3 (C), 134.7 (2 × CH), 134.1 (C), 128.8 (2 × CH), 128.5 (2 × CH), 128.44 (2 × CH), 128.42 (CH), 123.2 (C), 87.1 (CH), 74.9 (CH_2), 74.4 (CH).

SFC (IC-3, CO_2/CH_3OH = 94:6, 2.0 mL/min, 298 K, 254 nm): t_R(*anti*, major) = 9.6 min, t_R(*anti*, minor) = 12.6 min, t_R(*syn*, major) = 13.9 min, t_R(*syn*, minor) = 14.6 min, d.r. (*syn:anti*) = 30:1, e.r.(*syn*) = 96:4 (92% *ee*), e.r.(*anti*) = 87:13 (74% *ee*).

HRMS-CI (m/z): $[M + Na]^+$ calcd for $C_{23}H_{21}ClO_3SNa$, 435.0792; found, 435.0792.

R_f = 0.43 (20% ethyl acetate-hexane; UV).

Synthesis of S-(p-tolyl) (2R,3S)-2-(benzyloxy)-3-(4-cyanophenyl)-3-hydroxypropanethioate **10r**

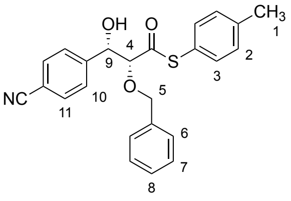

Following the general method, (*R*,*S*)-**10r** was prepared from 4-formylbenzonitrile (39 mg, 0.30 mmol, 1 equiv) and MAHT **8** (114 mg, 0.36 mmol, 1.20 equiv) in toluene (0.40 M, 0.75 mL). The reaction mixture was stirred for 24 h. The residue was purified by column chromatography (eluting with 5% ethyl acetate–hexane initially, grading to 30% ethyl acetate–hexane). The product was obtained as a white solid (116 mg, 96%).

Major diastereomer:

^{1}H NMR (500 MHz, $CDCl_3$) δ 7.58 (d, *J* = 8.3 Hz, 2H, H_{11}), 7.43 (d, *J* = 8.3 Hz, 2H, H_{10}), 7.37 – 7.18 (m, 9H, $H_{2,3,6\text{-}8}$), 5.09 (d, *J* = 3.8 Hz, 1H, H_9), 4.84 (d, *J* = 11.3 Hz, 1H, H_5), 4.40 (d, *J* = 11.3 Hz, 1H, H_5), 4.17 (d, *J* = 3.8 Hz, 1H, H_4), 3.61 (brs, 1H, OH), 2.39 (s, 3H, H_1).

^{13}C NMR (126 MHz, $CDCl_3$) δ 200.2 (C), 145.0 (C), 140.2 (C), 135.7 (C), 134.5 (2 × CH), 132.1 (2 × CH), 130.3 (2 × CH), 128.7 (2 × CH), 128.6 (3 × CH), 127.2 (2 × CH), 123.0 (C), 118.8 (C), 111.7 (C), 87.3 (CH), 74.7 (CH_2), 74.1 (CH), 21.5 (CH_3).

Minor diastereomer (detectable non-overlapping resonances):

^{1}H NMR (500 MHz, $CDCl_3$) δ 7.47 (d, *J* = 8.3 Hz, 2H, H_{10}), 5.00 (d, *J* = 6.4 Hz, 1H, H_9), 4.79 (d, *J* = 11.1 Hz, 1H, H_5), 4.35 (d, *J* = 11.1 Hz, 1H, H_5), 4.14 (d, *J* = 6.4 Hz, 1H, H_4).

^{13}C NMR (126 MHz, $CDCl_3$) δ 144.4 (C), 140.2 (C), 136.0 (C), 134.6 (2 × CH), 132.0 (2 × CH), 128.5 (2 × CH), 128.2 (2 × CH), 86.8 (CH), 75.0 (CH_2), 74.3 (CH).

SFC (IC-3, CO_2/CH_3OH = 80:20, 2.0 mL/min, 298 K, 254 nm): t_R(*anti*, major) = 2.5 min, t_R(*anti*, minor) = 3.0 min, t_R(*syn*, major) = 3.6 min, t_R(*syn*, minor) = 3.9 min, d.r. (*syn:anti*) = 28:1, e.r.(*syn*) = 99:1 (99% *ee*), e.r.(*anti*) = 87:13 (73% *ee*).

HRMS-CI (m/z): $[M + Na]^+$ calcd for $C_{24}H_{21}NO_3SNa$, 426.1134; found, 426.1136.

R_f = 0.28 (20% ethyl acetate-hexane; UV).

The absolute stereochemistry for (2*R*,3*S*)-**10r** was confirmed by x-ray crystallography (see *Catalog of X-ray data*). The crystal was grown by a slow evaporation of dichloromethane.

Synthesis of S-(p-tolyl) (2R,3S)-2-(benzyloxy)-3-hydroxy-3-(4-(trifluoromethyl)phenyl)propanethioate **10s**

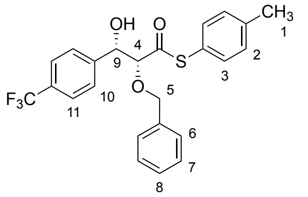

Following the general method, (*R*,*S*)-**10s** was prepared from 4-(trifluoromethyl)benzaldehyde (52 mg, 0.30 mmol, 1 equiv) and MAHT **8** (114 mg, 0.36 mmol, 1.20 equiv). The reaction mixture was stirred for 24 h. The residue was purified by column chromatography (eluting with 5% ethyl acetate–hexane initially, grading to 30% ethyl acetate–hexane). The product was obtained as a white solid (126 mg, 94%).

Major diastereomer:

^{1}H NMR (500 MHz, $CDCl_3$) δ 7.57 (d, *J* = 8.1 Hz, 2H, H_{11}), 7.44 (d, *J* = 8.1 Hz, 2H, H_{10}), 7.32 – 7.17 (m, 9H, $H_{2,3,6\text{-}8}$), 5.11 (d, *J* = 3.7 Hz, 1H, H_9), 4.85 (d, *J* = 11.3 Hz, 1H, H_5), 4.41 (d, *J* = 11.3 Hz, 1H, H_5), 4.18 (d, *J* = 3.7 Hz, 1H, H_4), 2.39 (s, 3H, H_1).

^{13}C NMR (126 MHz, $CDCl_3$) δ 200.3 (C), 143.7 (C), 140.2 (C), 135.9 (C), 134.6 (2 × CH), 130.3 (2 × CH), 128.68 (2 × CH), 128.65 (2 × CH), 128.6 (CH), 126.8 (2 × CH), 125.33 (q, *J* = 3.6 Hz, 2 × CH), 125.27 (C), 123.2 (C), 87.5 (CH), 74.7 (CH_2), 74.3 (CH), 21.5 (CH_3).

^{19}F NMR (470 MHz, $CDCl_3$) δ -62.47 (s, 3F).

Minor diastereomer (detectable non-overlapping resonances):

^{1}H NMR (500 MHz, $CDCl_3$) δ 5.00 (d, *J* = 6.7 Hz, 1H, H_9), 4.78 (d, *J* = 11.1 Hz, 1H, H_5), 4.31 (d, *J* = 11.1 Hz, 1H, H_5), 4.14 (d, *J* = 6.7 Hz, 1H, H_4).

^{13}C NMR (126 MHz, $CDCl_3$) δ 134.7 (2 × CH), 130.1 (2 × CH), 128.5 (2 × CH), 123.2 (C), 87.0 (CH), 75.0 (CH_2), 74.5 (CH).

SFC (IC-3, CO_2/CH_3OH = 97:3, 2.0 mL/min, 298 K, 254 nm): t_R(*anti*, major) = 7.2 min, t_R(*anti*, minor) = 9.8 min, t_R(*syn*, major) = 10.9 min, t_R(*syn*, minor) = 12.3 min, d.r. (*syn:anti*) = 21:1, e.r.(*syn*) = 98:2 (95% *ee*), e.r.(*anti*) = 83:17 (66% *ee*).

HRMS-CI (m/z): $[M + Na]^+$ calcd for $C_{24}H_{21}F_3O_3SNa$, 469.1056; found, 469.1058.

R_f = 0.28 (20% ethyl acetate-hexane; UV).

Synthesis of S-(p-tolyl) (2R,3S)-2-(benzyloxy)-3-hydroxy-3-(p-tolyl)propanethioate **10t**

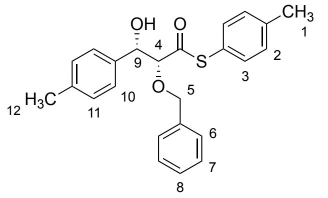

Following the general method, (*R*,*S*)-**10t** was prepared from *p*-tolualdehyde (35 µL, 0.30 mmol, 1 equiv) and MAHT **8** (114 mg, 0.36 mmol, 1.20 equiv) in toluene (0.40 M, 0.75 mL). The reaction mixture was stirred for 48 h. The residue was purified by column chromatography (eluting with 5% ethyl acetate–hexane initially, grading to 30% ethyl acetate–hexane). The product was obtained as a white solid (30 mg, 26%).

Major diastereomer:

^{1}H NMR (500 MHz, $CDCl_3$) δ 7.37 – 7.21 (m, 11H, $H_{2,6\text{-}8,10,11}$), 7.16 (d, *J* = 7.8 Hz, 2H, H_3), 5.04 (d, *J* = 4.2 Hz, 1H, H_9), 4.78 (d, *J* = 11.1 Hz, 1H, H_5), 4.43 (d, *J* = 11.1 Hz, 1H, H_5), 4.18 (d, *J* = 4.2 Hz, 1H, H_4), 2.38 (s, 3H, $H_{1/12}$), 2.37 (s, 3H, $H_{1/12}$).

^{13}C NMR (126 MHz, $CDCl_3$) δ 200.0 (C), 139.9 (C), 137.9 (C), 136.5 (C), 136.4 (C), 134.7 (2 × CH), 130.2 (2 × CH), 129.2 (2 × CH), 128.62 (2 × CH), 128.60 (2 × CH), 128.4 (CH), 126.5 (2 × CH), 123.6 (C), 88.6 (CH), 74.8 (CH), 74.6 (CH_2), 21.5 (CH_3), 21.3 (CH_3).

Minor diastereomer (detectable non-overlapping resonances):

^{1}H NMR (500 MHz, $CDCl_3$) δ 4.93 (d, *J* = 6.7 Hz, 1H, H_9), 4.70 (d, *J* = 11.1 Hz, 1H, H_5), 4.33 (d, *J* = 11.1 Hz, 1H, H_5).

^{13}C NMR (126 MHz, $CDCl_3$) δ 202.1 (C), 139.9 (C), 138.1 (C), 136.7 (C), 136.3 (C), 134.7 (2 × CH), 129.1 (2 × CH), 128.6 (2 × CH), 128.5 (2 × CH), 128.3 (CH), 127.4 (2 × CH), 87.5 (CH), 75.0 (CH), 74.8 (CH_2), 21.4 (CH_3).

SFC (IC-3, CO_2/CH_3OH = 90:10, 2.0 mL/min, 298 K, 254 nm): t_R(*anti*, major) = 7.0 min, t_R(*syn*, major) = 8.5 min, t_R(*anti*, minor) = 9.4 min, t_R(*syn*, minor) = 10.5 min, d.r. (*syn:anti*) = 11:1, e.r.(*syn*) = 95:5 (89% *ee*), e.r.(*anti*) = 76:24 (53% *ee*).

HRMS-CI (m/z): $[M + Na]^+$ calcd for $C_{24}H_{24}O_3SNa$, 415.1338; found, 415.1342.

R_f = 0.43 (20% ethyl acetate-hexane; UV).

Synthesis of S-(p-tolyl) (2R,3S)-2-(benzyloxy)-3-hydroxy-3-(naphthalen-2-yl)propanethioate **10u**

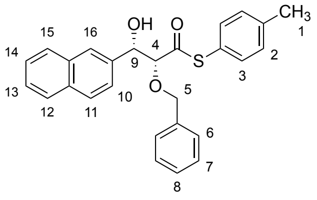

Following the general method, (*R*,*S*)-**10u** was prepared from 2-naphthaldehyde (47 mg, 0.30 mmol, 1 equiv) and MAHT **8** (114 mg, 0.36 mmol, 1.20 equiv) in toluene (0.40 M, 0.75 mL). The reaction mixture was stirred for 48 h. The residue was purified by column chromatography (eluting with 5% ethyl acetate–hexane initially, grading to 30% ethyl acetate–hexane). The product was obtained as a white solid (59 mg, 46%).

Major diastereomer:

^{1}H NMR (500 MHz, $CDCl_3$) δ 7.93 – 7.83 (m, 4H, $H_{10\text{-}16}$), 7.58 – 7.49 (m, 3H, $H_{10\text{-}16}$), 7.33 – 7.24 (m, 9H, $H_{2,3,6\text{-}8}$), 5.30 (d, *J* = 3.9 Hz, 1H, H_9), 4.82 (d, *J* = 11.1 Hz, 1H, H_5), 4.43 (d, *J* = 11.1 Hz, 1H, H_5), 4.34 (d, *J* = 3.9 Hz, 1H, H_4), 3.06 (brs, 1H, OH), 2.42 (s, 3H, H_1).

^{13}C NMR (126 MHz, $CDCl_3$) δ 200.2 (C), 139.9 (C), 137.1 (C), 136.2 (C), 134.6 (2 × CH), 133.29 (C), 133.27 (C), 130.2 (2 × CH), 128.6 (2 × CH), 128.5 (2 × CH), 128.4 (CH), 128.23 (CH), 128.18 (CH), 127.8 (CH), 126.3 (CH), 126.2 (CH), 125.7 (CH), 124.3 (CH), 123.5 (C), 88.2 (CH), 75.0 (CH), 74.7 (CH_2), 21.5 (CH_3).

Minor diastereomer (detectable non-overlapping resonances):

^{1}H NMR (500 MHz, $CDCl_3$) 5.17 (d, *J* = 6.8 Hz, 1H, H_9), 4.75 (d, *J* = 11.1 Hz, 1H, H_5), 4.30 (d, *J* = 6.8 Hz, 1H, H_4).

^{13}C NMR (126 MHz, $CDCl_3$) δ 202.2 (C), 140.0 (C), 136.7 (C), 136.4 (C), 134.7 (2 × CH), 133.5 (C), 133.2 (C), 128.5 (2 × CH), 128.28 (CH), 128.26 (CH), 128.1 (CH), 126.7 (CH), 125.1 (CH), 123.4 (C), 87.4 (CH), 75.2 (CH), 74.6 (CH_2).

SFC (IA-3, CO_2/CH_3OH = 85:15, 2.0 mL/min, 298 K, 254 nm): t_R(*syn*, major) = 11.0 min, t_R(*anti*, minor) = 12.7 min, t_R(*anti*, major) = 13.9 min, t_R(*syn*, minor) = 17.7 min, d.r. (*syn:anti*) = 10:1, e.r.(*syn*) = 91:9 (82% *ee*), e.r.(*anti*) = 76:24 (51% *ee*).

HRMS-CI (m/z): $[M + Na]^+$ calcd for $C_{27}H_{24}O_3SNa$, 451.1338; found, 451.1342.

R_f = 0.44 (20% ethyl acetate-hexane; UV).

Synthesis of S-(p-tolyl) (2R,3S)-2-(benzyloxy)-3-hydroxy-3-(2-nitrophenyl)propanethioate **10v**

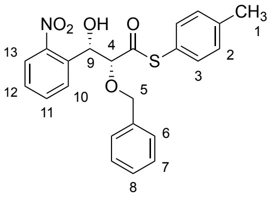

Following the general method, (*R*,*S*)-**10v** was prepared from 2-nitrobenzaldehyde (40 µL, 0.30 mmol, 1 equiv) and MAHT **8** (114 mg, 0.36 mmol, 1.20 equiv) in toluene (0.40 M, 0.75 mL). The reaction mixture was stirred for 48 h. The residue was purified by column chromatography (eluting with 5% ethyl acetate–hexane initially, grading to 30% ethyl acetate–hexane). The product was obtained as a white solid (116 mg, 91%).

Major diastereomer:

^{1}H NMR (500 MHz, $CDCl_3$) δ 7.91 (dd, *J* = 8.3, 1.3 Hz, 1H, H_{13}), 7.81 (dd, *J* = 7.8, 1.4 Hz, 1H, H_{10}), 7.61 (ddd, *J* = 7.8, 7.5, 1.3 Hz, 1H, H_{11}), 7.42 (ddd, *J* = 8.3, 7.5, 1.4 Hz, 1H, H_{12}), 7.31 – 7.18 (m, 7H, $H_{2,6\text{-}8}$), 7.12 (d, *J* = 7.7 Hz, 2H, H_3), 5.79 (d, *J* = 3.1 Hz, 1H, H_9), 4.76 (d, *J* = 11.2 Hz, 1H, H_5), 4.43 (d, *J* = 3.1 Hz, 1H, H_4), 4.27 (d, *J* = 11.2 Hz, 1H, H_5), 2.87 (brs, 1H, OH), 2.35 (s, 3H, H_1).

^{13}C NMR (126 MHz, $CDCl_3$) δ 199.8 (C), 147.6 (C), 140.0 (C), 135.9 (C), 135.6 (C), 134.7 (2 × CH), 133.3 (CH), 130.2 (2 × CH), 130.0 (CH), 128.8 (CH), 128.7 (2 × CH), 128.6 (2 × CH), 128.5 (CH), 124.8 (CH), 123.5 (C), 86.0 (CH), 75.1 (CH_2), 70.3 (CH), 21.5 (CH_3).

Minor diastereomer (detectable non-overlapping resonances):

^{1}H NMR (500 MHz, $CDCl_3$) δ 7.94 (dd, *J* = 8.2, 1.3 Hz, 1H, H_{13}), 7.75 (dd, *J* = 7.9, 1.5 Hz, 1H, H_{10}), 7.55 (ddd, *J* = 7.9, 7.7, 1.3 Hz, 1H, H_{11}), 5.75 (d, *J* = 5.1 Hz, 1H, H_9), 4.80 (d, *J* = 11.3 Hz, 1H, H_5), 4.56 (d, *J* = 11.3 Hz, 1H, H_5), 4.35 (d, *J* = 5.1 Hz, 1H, H_4), 2.34 (s, 3H, H_1).

^{13}C NMR (126 MHz, $CDCl_3$) δ 199.9 (C), 148.2 (C), 136.2 (C), 134.6 (2 × CH), 133.4 (CH), 129.5 (CH), 128.8 (CH), 128.6 (2 × CH), 128.4 (CH), 124.7 (CH), 86.3 (CH), 74.2 (CH_2), 70.6 (CH).

SFC (IA-3, CO_2/CH_3OH = 80:20, 2.0 mL/min, 298 K, 254 nm): t_R(*syn*, major) = 3.6 min, t_R(*anti*, minor) = 4.3 min, t_R(*syn*, major) = 4.8 min, t_R(*anti*, minor) = 5.9 min, d.r. (*syn:anti*) = 10:1, e.r.(*syn*) = 95:5 (91% *ee*), e.r.(*anti*) = 82:18 (64% *ee*).

HRMS-CI (m/z): $[M + Na]^+$ calcd for $C_{23}H_{21}NO_5SNa$, 446.1033; found, 446.1035.

R_f = 0.28 (20% ethyl acetate-hexane; UV).

Synthesis of S-(p-tolyl) (2R,3S)-2-(benzyloxy)-3-(2-chlorophenyl)-3-hydroxypropanethioate **10w**

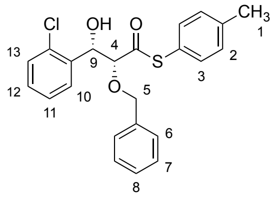

Following the general method, (*R*,*S*)-**10w** was prepared from 2-chlorobenzaldehyde (34 µL, 0.30 mmol, 1 equiv) and MAHT **8** (114 mg, 0.36 mmol, 1.20 equiv) in toluene (0.40 M, 0.75 mL). The reaction mixture was stirred for 48 h. The residue was purified by column chromatography (eluting with 5% ethyl acetate–hexane initially, grading to 30% ethyl acetate–hexane). The product was obtained as a white solid (102 mg, 82%).

Major diastereomer:

^{1}H NMR (500 MHz, $CDCl_3$) δ 7.59 (dd, *J* = 7.7, 1.6 Hz, 1H, H_{13}), 7.34 – 7.18 (m, 10H, $H_{2,6\text{-}8,10\text{-}13}$), 7.14 – 7.09 (m, 2H, H_3), 5.52 (d, *J* = 2.8 Hz, 1H, H_9), 4.67 (d, *J* = 11.1 Hz, 1H, H_5), 4.37 (d, *J* = 2.8 Hz, 1H, H_4), 4.22 (d, *J* = 11.1 Hz, 1H, H_5), 2.66 (brs, 1H, OH), 2.36 (s, 3H, H_1).

^{13}C NMR (126 MHz, $CDCl_3$) δ 199.8 (C), 139.8 (C), 137.4 (C), 136.0 (C), 134.7 (2 × CH), 131.7 (C), 130.2 (2 × CH), 129.4 (CH), 129.1 (CH), 128.7 (CH), 128.6 (2 × CH), 128.5 (2 × CH), 128.4 (CH), 126.9 (CH), 123.7 (C), 85.4 (CH), 75.1 (CH_2), 71.6 (CH), 21.5 (CH_3).

Minor diastereomer (detectable non-overlapping resonances):

^{1}H NMR (500 MHz, $CDCl_3$) δ 7.53 (dd, *J* = 7.5, 2.0 Hz, 1H, H_{13}), 5.45 (d, *J* = 5.0 Hz, 1H, H_9), 4.82 (d, *J* = 11.4 Hz, 1H, H_5), 4.51 (d, *J* = 11.4 Hz, 1H, H_5), 2.34 (s, 3H, H_1).

^{13}C NMR (126 MHz, $CDCl_3$) δ 199.6 (C), 139.8 (C), 136.5 (C), 136.2 (C), 134.6 (2 × CH), 132.6 (C), 129.3 (CH), 129.2 (CH), 128.8 (CH), 128.7 (2 × CH), 128.6 (CH), 128.4 (CH), 127.0 (CH), 123.6 (C), 85.8 (CH), 74.1 (CH), 71.7 (CH), 21.4 (CH_3).

SFC (OD-H, CO_2/CH_3OH = 90:10, 2.0 mL/min, 298 K, 254 nm): t_R(*syn*, minor) = 6.3 min, t_R(*syn*, major) = 7.1 min, t_R(*anti*, major) = 7.7 min, t_R(*anti*, minor) = 8.5 min, d.r. (*syn:anti*) = 6:1, e.r.(*syn*) = 93:7 (86% *ee*), e.r.(*anti*) = 76:24 (52% *ee*).

HRMS-CI (m/z): $[M + Na]^+$ calcd for $C_{23}H_{21}ClO_3SNa$, 435.0792; found, 435.0794.

R_f = 0.28 (20% ethyl acetate-hexane; UV).

Synthesis of S-(p-tolyl) (2R,3S)-2-(benzyloxy)-3-hydroxy-3-(3-methoxyphenyl)propanethioate **10x**

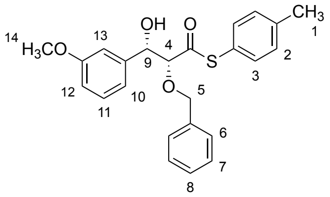

Following the general method, (*R*,*S*)-**10w** was prepared from 3-methoxybenzaldehyde (37 µL, 0.30 mmol, 1 equiv) and MAHT **8** (114 mg, 0.36 mmol, 1.20 equiv) in toluene (0.40 M, 0.75 mL). The reaction mixture was stirred for 48 h. The residue was purified by column chromatography (eluting with 5% ethyl acetate–hexane initially, grading to 30% ethyl acetate–hexane). The product was obtained as a colorless oil (46 mg, 37%).

Major diastereomer:

^{1}H NMR (500 MHz, $CDCl_3$) δ 7.37 – 7.22 (m, 10H, $H_{2,3,6\text{-}8,11}$), 6.98 – 6.92 (m, 2H, $H_{12,13}$), 6.89 – 6.85 (m, 1H, H_{10}), 5.07 (d, *J* = 3.8 Hz, 1H, H_9), 4.78 (d, *J* = 11.1 Hz, 1H, H_5), 4.40 (d, *J* = 11.1 Hz, 1H, H_5), 4.20 (d, *J* = 3.9 Hz, 1H, H_4), 3.77 (s, 3H, H_{14}), 2.57 (brs, 1H, OH), 2.39 (s, 3H, H_1).

^{13}C NMR (126 MHz, $CDCl_3$) δ 200.1 (C), 159.8 (C), 141.3 (C), 139.9 (C), 136.4 (C), 134.7 (2 × CH), 130.2 (2 × CH), 129.5 (CH), 128.60 (2 × CH), 128.59 (2 × CH), 128.4 (CH), 123.6 (C), 118.8 (CH), 114.1 (CH), 111.8 (CH), 88.3 (CH), 74.8 (CH_2), 74.7 (CH), 55.3 (CH_3), 21.5 (CH_3).

Minor diastereomer (detectable non-overlapping resonances):

^{1}H NMR (500 MHz, $CDCl_3$) δ 7.01 (d, *J* = 7.6 Hz, 1H, H_{12}), 6.90 (ddd, *J* = 8.3, 2.7, 1.0 Hz, 1H, H_{10}), 4.93 (d, *J* = 6.8 Hz, 1H, H_9), 4.72 (d, *J* = 11.0 Hz, 1H, H_5), 4.31 (d, *J* = 11.0 Hz, 1H, H_5), 4.16 (d, *J* = 6.8 Hz, 1H, H_4).

^{13}C NMR (126 MHz, $CDCl_3$) δ 202.1 (C), 140.8 (C), 136.6 (C), 134.7 (2 × CH), 130.2 (2 × CH), 129.3 (CH), 128.6 (2 × CH), 128.5 (2 × CH), 128.3 (CH), 123.5 (C), 119.9 (CH), 114.5 (CH), 112.4 (CH), 87.3 (CH), 75.0 (CH_2), 74.8 (CH).

SFC (IC-3, CO_2/CH_3OH = 80:20, 2.0 mL/min, 298 K, 254 nm): t_R(*anti*, major) = 2.9 min, t_R(*anti*, minor) = 3.8 min, t_R(*syn*, major) = 4.1 min, t_R(*syn*, minor) = 6.3 min, d.r. (*syn:anti*) = 7:1, e.r.(*syn*) = 93:7 (86% *ee*), e.r.(*anti*) = 74:26 (47% *ee*).

HRMS-CI (m/z): $[M + Na]^+$ calcd for $C_{24}H_{24}O_4SNa$, 431.1288; found, 431.1290.

R_f = 0.40 (20% ethyl acetate-hexane; UV).

Synthesis of methyl 3-((1S,2R)-2-(benzyloxy)-1-hydroxy-3-oxo-3-(p-tolylthio)propyl)benzoate **10y**

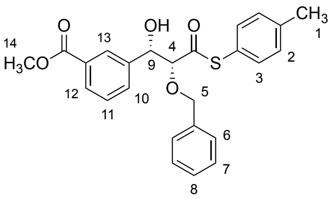

Following the general method, (1*R*,2*S*)-**10y** was prepared from methyl 3-formylbenzoate (49 mg, 0.30 mmol, 1 equiv) and MAHT **8** (114 mg, 0.36 mmol, 1.20 equiv). The reaction mixture was stirred for 48 h. The residue was purified by column chromatography (eluting with 5% ethyl acetate–hexane initially, grading to 40% ethyl acetate–hexane). The product was obtained as a white solid (95 mg, 72%).

Major diastereomer:

^{1}H NMR (500 MHz, CDCl$_3$) δ 8.05 (dd, *J* = 1.5, 1.5 Hz, 1H, H_{13}), 8.00 (ddd, *J* = 7.8, 1.6, 1.5 Hz, 1H, H_{12}), 7.58 (ddd, *J* = 7.8, 1.6, 1.5 Hz, 1H, H_{10}), 7.42 (dd, *J* = 7.8, 7.8 Hz, 1H, H_{11}), 7.34 – 7.21 (m, 9H, $H_{2,3,6\text{-}8}$), 5.13 (d, *J* = 4.0 Hz, 1H, H_9), 4.81 (d, *J* = 11.2 Hz, 1H, H_5), 4.39 (d, *J* = 11.2 Hz, 1H, H_5), 4.20 (d, *J* = 4.0 Hz, 1H, H_4), 3.93 (s, 3H, H_{14}), 2.94 (brs, 1H, OH), 2.39 (s, 3H, H_3).

^{13}C NMR (126 MHz, CDCl$_3$) δ 200.0 (C), 166.9 (C), 140.0 (C), 139.9 (C), 136.0 (C), 134.6 (2 × CH), 131.1 (CH), 130.2 (2 × CH), 129.3 (2 × CH), 128.6 (2 × CH), 128.54 (2 × CH), 128.48 (CH), 128.4 (CH), 127.7 (CH), 123.3 (C), 88.0 (CH), 74.6 (CH$_2$), 74.4 (CH), 52.2 (CH$_3$), 21.4 (CH$_3$).

Minor diastereomer (detectable non-overlapping resonances):

^{1}H NMR (500 MHz, CDCl$_3$) δ 8.10 (s, 1H, H_{13}), 5.03 (d, *J* = 6.6 Hz, 1H, H_9), 4.76 (d, *J* = 11.1 Hz, 1H, H_5), 4.34 (d, *J* = 11.1 Hz, 1H, H_5).

^{13}C NMR (126 MHz, CDCl$_3$) δ 201.8 (C), 167.0 (C), 140.0 (C), 136.3 (C), 134.6 (2 × CH), 132.0 (CH), 130.1 (2 × CH), 129.5 (CH), 128.32 (CH), 128.26 (CH), 123.2 (C), 87.1 (CH), 74.7 (CH$_2$), 74.6 (CH).

SFC (IC-3, CO_2/CH_3OH = 85:15, 2.0 mL/min, 298 K, 254 nm): t_R(*anti*, major) = 5.8 min, t_R(*anti*, minor) = 7.2 min, t_R(*syn*, major) = 9.1 min, t_R(*syn*, minor) = 9.8 min, d.r. (*syn:anti*) = 13:1, e.r.(*syn*) = 98:2 (95% *ee*), e.r.(*anti*) = 85:15 (70% *ee*).

HRMS-CI (m/z): [M + Na]$^+$ calcd for $C_{25}H_{24}O_5SNa$, 459.1237; found, 459.1236.

R_f = 0.32 (20% ethyl acetate-hexane; UV).

Synthesis of S-(p-tolyl) (2R,3S)-3-(3-acetylphenyl)-2-(benzyloxy)-3-hydroxypropanethioate **10z**

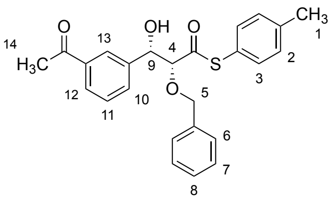

Following the general method, (*R*,*S*)-**10z** was prepared from 3-acetylbenzaldehyde (44 mg, 0.30 mmol, 1 equiv) and MAHT **8** (114 mg, 0.36 mmol, 1.20 equiv) in toluene (0.40 M, 0.75 mL). The reaction mixture was stirred for 48 h. The residue was purified by column chromatography (eluting with 5% ethyl acetate–hexane initially, grading to 40% ethyl acetate–hexane). The product was obtained as a white solid (102 mg, 81%).

Major diastereomer:

^{1}H NMR (500 MHz, CDCl$_3$) δ 7.99 – 7.86 (m, 2H, $H_{12,13}$), 7.58 (d, *J* = 7.7 Hz, 1H, H_{10}), 7.44 (dd, *J* = 7.7, 7.7 Hz, 1H, H_{11}), 7.33 – 7.17 (m, 9H, $H_{2,3,6\text{-}8}$), 5.14 (d, *J* = 4.0 Hz, 1H, H_9), 4.83 (d, *J* = 11.1 Hz, 1H, H_5), 4.38 (d, *J* = 11.1 Hz, 1H, H_5), 4.20 (d, *J* = 4.0 Hz, 1H, H_4), 3.04 (brs, 1H, OH), 2.55 (s, 3H, H_{14}), 2.38 (s, 3H, H_1).

^{13}C NMR (126 MHz, CDCl$_3$) δ 200.1 (C), 198.1 (C), 140.2 (C), 140.0 (C), 137.1 (C), 136.0 (C), 134.6 (2 × CH), 131.3 (CH), 130.2 (2 × CH), 128.7 (CH), 128.58 (2 × CH), 128.56 (2 × CH), 128.4 (CH), 128.0 (CH), 126.6 (CH), 123.2 (C), 88.0 (CH), 74.7 (CH$_2$), 74.4 (CH), 26.8 (CH$_3$), 21.4 (CH$_3$).

Minor diastereomer (detectable non-overlapping resonances):

^{1}H NMR (500 MHz, CDCl$_3$) δ 5.03 (d, *J* = 6.6 Hz, 1H, H_9), 4.77 (d, *J* = 11.1 Hz, 1H, H_5), 4.33 (d, *J* = 11.1 Hz, 1H, H_5), 2.54 (s, 3H, H_{14}).

^{13}C NMR (126 MHz, CDCl$_3$) δ 201.8 (C), 140.0 (C), 139.8 (C), 137.0 (C), 136.3 (C), 134.6 (2 × CH), 132.2 (CH), 128.5 (2 × CH), 128.4 (2 × CH), 128.1 (CH), 127.5 (CH), 123.2 (C), 87.2 (CH), 74.8 (CH$_2$), 74.6 (CH).

SFC (IC-3, CO_2/CH_3OH = 80:20, 2.0 mL/min, 298 K, 254 nm): t_R(*anti*, major) = 3.7 min, t_R(*anti*, minor) = 4.6 min, t_R(*syn*, major) = 5.7 min, t_R(*syn*, minor) = 6.1 min, d.r. (*syn:anti*) = 13:1, e.r.(*syn*) = 97:3 (94% *ee*), e.r.(*anti*) = 87:13 (73% *ee*).

HRMS-CI (m/z): [M + Na]$^+$ calcd for $C_{25}H_{24}O_4SNa$, 443.1288; found, 443.1279.

R_f = 0.34 (30% ethyl acetate-hexane; UV).

Synthesis of S-(p-tolyl) (2R,3R)-3-(benzo[b]thiophen-2-yl)-2-(benzyloxy)-3-hydroxypropanethioate **10aa**

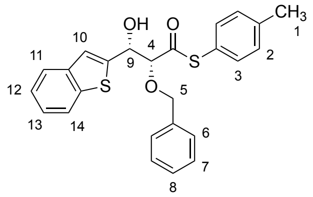

Following the general method, (*R*,*R*)-**10aa** was prepared from benzo[b]thiophene-2-carboxaldehyde (49 mg, 0.30 mmol, 1 equiv) and MAHT **8** (114 mg, 0.36 mmol, 1.20 equiv) in toluene (0.40 M, 0.75 mL). The reaction mixture was stirred for 48 h. The residue was purified by column chromatography (eluting with 5% ethyl acetate–hexane initially, grading to 30% ethyl acetate–hexane). The product was obtained as a light yellow solid (79 mg, 60%).

Major diastereomer:

^{1}H NMR (500 MHz, $CDCl_3$) δ 7.77 (dd, *J* = 7.7, 1.4 Hz, 1H, H_{14}), 7.67 (dd, *J* = 8.2, 1.3 Hz, 1H, H_{11}), 7.35 – 7.24 (m, 7H, $H_{6-8,12,13}$), 7.22 – 7.15 (m, 5H, $H_{2,3,10}$), 5.32 (d, *J* = 3.5 Hz, 1H, H_9), 4.85 (d, *J* = 11.0 Hz, 1H, H_5), 4.57 (d, *J* = 11.0 Hz, 1H, H_5), 4.30 (d, *J* = 3.5 Hz, 1H, H_4), 2.81 (brs, 1H, OH), 2.33 (s, 3H, H_1).

^{13}C NMR (126 MHz, $CDCl_3$) δ 200.2 (C), 144.0 (C), 140.0 (C), 139.8 (C), 139.4 (C), 136.2 (C), 134.7 (2 × CH), 130.2 (2 × CH), 128.7 (2 × CH), 128.6 (2 × CH), 128.5 (2 × CH), 124.4 (CH), 123.8 (CH), 123.3 (C), 122.5 (CH), 121.9 (CH), 87.3 (CH), 75.0 (CH_2), 71.8 (CH), 21.5 (CH_3).

Minor diastereomer (detectable non-overlapping resonances):

^{1}H NMR (500 MHz, $CDCl_3$) δ 5.27 (d, *J* = 6.3 Hz, 1H, H_9), 4.79 (d, *J* = 11.1 Hz, 1H, H_5), 4.47 (d, *J* = 11.1 Hz, 1H, H_5), 4.26 (d, *J* = 6.3 Hz, 1H, H_4).

^{13}C NMR (126 MHz, $CDCl_3$) δ 201.5 (C), 143.1 (C), 139.3 (C), 136.5 (C), 134.7 (2 × CH), 128.6 (2 × CH), 128.4 (CH), 124.5 (CH), 123.8 (CH), 123.3 (C), 122.9 (CH), 122.6 (CH), 87.0 (CH), 75.3 (CH_2), 72.2 (CH).

SFC (IC-3, CO_2/CH_3OH = 70:30, 2.0 mL/min, 298 K, 254 nm): t_R(*syn*, major) = 2.6 min, t_R(*anti*, minor) = 3.4 min, t_R(*anti*, major) = 3.7 min, t_R(*syn*, minor) = 4.1 min, d.r. (*syn:anti*) = 8:1, e.r.(*syn*) = 94:6 (88% *ee*), e.r.(*anti*) = 69:31 (38% *ee*).

HRMS-CI (m/z): $[M + Na]^+$ calcd for $C_{25}H_{22}O_3S_2Na$, 457.0903; found, 457.0884.

R_f = 0.47 (20% ethyl acetate-hexane; UV).

Synthesis of S-(p-tolyl) (2R,3R)-2-(benzyloxy)-3-(furan-2-yl)-3-hydroxypropanethioate **10ab**

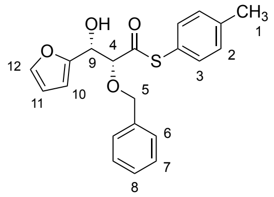

Following the general method, (*R*,*R*)-**10ab** was prepared from furfural (25 µL, 0.30 mmol, 1 equiv) and MAHT **8** (114 mg, 0.36 mmol, 1.20 equiv). The reaction mixture was stirred for 24 h. The residue was purified by column chromatography (eluting with 5% ethyl acetate–hexane initially, grading to 30% ethyl acetate–hexane). The product was obtained as a colorless oil (97 mg, 88%).

Major diastereomer:

^{1}H NMR (500 MHz, $CDCl_3$) δ 7.32 – 7.24 (m, 6H, $H_{6-8,12}$), 7.19 (d, *J* = 8.2 Hz, 2H, H_2), 7.15 (d, *J* = 8.2 Hz, 2H, H_3), 6.23 – 6.18 (m, 2H, $H_{10,11}$), 4.99 (d, *J* = 3.8 Hz, 1H, H_9), 4.73 (d, *J* = 11.1 Hz, 1H, H_5), 4.42 (d, *J* = 11.1 Hz, 1H, H_5), 4.33 (d, *J* = 3.8 Hz, 1H, H_4), 2.67 (brs, 1H, OH), 2.29 (s, 3H, H_1).

^{13}C NMR (126 MHz, $CDCl_3$) δ 199.9 (C), 152.6 (C), 142.3 (CH), 139.9 (C), 136.4 (C), 134.6 (2 × CH), 130.2 (2 × CH), 128.64 (2 × CH), 128.55 (2 × CH), 128.5 (CH), 123.5 (C), 110.6 (CH), 108.0 (CH), 85.6 (CH), 74.7 (CH_2), 69.4 (CH), 21.5 (CH_3).

Minor diastereomer (detectable non-overlapping resonances):

^{1}H NMR (500 MHz, $CDCl_3$) δ 6.35 – 6.30 (m, 2H, $H_{10,11}$), 4.92 (d, *J* = 6.5 Hz, 1H, H_9), 4.31 (d, *J* = 6.5 Hz, 1H, H_4).

^{13}C NMR (126 MHz, $CDCl_3$) δ 201.2 (C), 151.8 (C), 142.6 (CH), 140.0 (C), 136.6 (C), 134.7 (2 × CH), 128.39 (2 × CH), 128.37 (CH), 123.3 (C), 110.6 (CH), 109.1 (CH), 85.7 (CH), 74.9 (CH_2), 69.0 (CH).

SFC (OJ-H, CO_2/CH_3OH = 80:20, 2.0 mL/min, 298 K, 254 nm): t_R(*syn*, minor) = 3.7 min, t_R(*syn*, major) = 4.0 min, t_R(*anti*, major) = 4.4 min, t_R(*anti*, minor) = 7.0 min, d.r. (*syn:anti*) = 8:1, e.r.(*syn*) = 93:7 (86% *ee*), e.r.(*anti*) = 76:24 (52% *ee*).

HRMS-CI (m/z): $[M + Na]^+$ calcd for $C_{21}H_{20}O_4SNa$, 391.0975; found, 391.0961.

R_f = 0.34 (20% ethyl acetate-hexane; UV).

Synthesis of S-(p-tolyl) (2R,3S)-2-(benzyloxy)-3-hydroxy-3-(oxazol-4-yl)propanethioate **10ac**

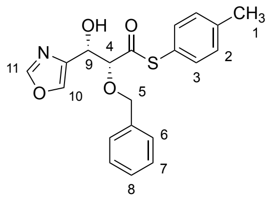

Following the general method, (*R*,*S*)-**10ac** was prepared from 4-oxazolecarboxaldehyde (29 mg, 0.30 mmol, 1 equiv) and MAHT **8** (114 mg, 0.36 mmol, 1.20 equiv). The reaction mixture was stirred for 24 h. The residue was purified by column chromatography (eluting with 5% ethyl acetate–hexane initially, grading to 30% ethyl acetate–hexane). The product was obtained as a light yellow solid (103 mg, 93%).

Major diastereomer:

^{1}H NMR (500 MHz, $CDCl_3$) δ 7.78 (d, *J* = 1.1 Hz, 1H, H_{10}), 7.66 (s, 1H, H_{11}), 7.39 – 7.28 (m, 7H, $H_{2,6\text{-}8}$), 7.23 (d, *J* = 7.9 Hz, 2H, H_3), 5.13 (dd, *J* = 3.1, 1.1 Hz, 1H, H_9), 4.88 (d, *J* = 11.0 Hz, 1H, H_5), 4.54 (d, *J* = 3.1 Hz, 1H, H_4), 4.50 (d, *J* = 11.0 Hz, 1H, H_5), 3.34 (brs, 1H, OH), 2.38 (s, 3H, H_1).

^{13}C NMR (126 MHz, $CDCl_3$) δ 200.0 (C), 151.2 (CH), 140.1 (C), 139.9 (C), 136.8 (CH), 136.4 (C), 134.7 (2 × CH), 130.2 (2 × CH), 128.62 (2 × CH), 128.58 (2 × CH), 128.4 (CH), 123.6 (C), 85.7 (CH), 75.0 (CH_2), 69.2 (CH), 21.4 (CH_3).

Minor diastereomer (detectable non-overlapping resonances):

^{1}H NMR (500 MHz, $CDCl_3$) δ 7.81 (s, 1H, H_{10}), 5.09 (d, *J* = 5.3 Hz, 1H, H_9).

^{13}C NMR (126 MHz, $CDCl_3$) δ 139.9 (C), 136.7 (CH), 128.7 (2 × CH), 86.1 (CH), 74.8 (CH_2), 68.5 (CH).

SFC (OD-H, CO_2/CH_3OH = 80:20, 2.0 mL/min, 298 K, 254 nm): t_R(*syn*, minor) = 2.0 min, t_R(*syn*, major) = 2.2 min, t_R(*anti*, major) = 2.5 min, t_R(*anti*, minor) = 2.8 min, d.r. (*syn:anti*) = 14:1, e.r.(*syn*) = 98:2 (96% *ee*), e.r.(*anti*) = 77:23 (53% *ee*).

HRMS-CI (m/z): $[M + H]^+$ calcd for $C_{20}H_{20}NO_4S$, 370.1108; found, 370.1093.

R_f = 0.37 (20% ethyl acetate-hexane; UV).

Synthesis of S-(p-tolyl) (2R,3S)-2-(benzyloxy)-3-hydroxy-3-(2-methylthiazol-4-yl)propanethioate **10ad**

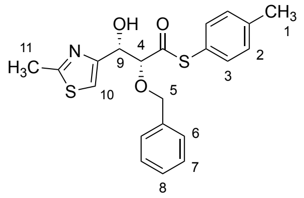

Following the general method, (*R*,*S*)-**10ad** was prepared from 2-methylthiazole-4-carboxaldehyde (38 mg, 0.30 mmol, 1 equiv) and MAHT **8** (114 mg, 0.36 mmol, 1.20 equiv). The reaction mixture was stirred for 24 h. The residue was purified by column chromatography (eluting with 5% ethyl acetate–hexane initially, grading to 50% ethyl acetate–hexane). The product was obtained as a yellow solid (105 mg, 87%).

Major diastereomer:

^{1}H NMR (500 MHz, $CDCl_3$) δ 7.32 – 7.28 (m, 5H, $H_{2,6/7,8}$), 7.25 – 7.20 (m, 4H, $H_{3,6/7}$), 7.14 (d, *J* = 1.1 Hz, 1H, H_{10}), 5.21 (dd, *J* = 3.1, 1.1 Hz, 1H, H_9), 4.83 (d, *J* = 11.2 Hz, 1H, H_5), 4.64 (d, *J* = 3.1 Hz, 1H, H_4), 4.42 (d, *J* = 11.2 Hz, 1H, H_5), 3.29 (brs, 1H, OH), 2.66 (s, 3H, H_{11}), 2.38 (s, 3H, H_1).

^{13}C NMR (126 MHz, $CDCl_3$) δ 200.0 (C), 166.7 (C), 154.8 (C), 139.8 (C), 136.6 (C), 134.7 (2 × CH), 130.2 (2 × CH), 128.6 (2 × CH), 128.5 (2 × CH), 128.3 (CH), 123.8 (C), 115.5 (CH), 86.3 (CH), 74.9 (CH_2), 72.1 (CH), 21.5 (CH_3), 19.0 (CH_3).

Minor diastereomer (detectable non-overlapping resonances):

^{1}H NMR (500 MHz, $CDCl_3$) δ 4.88 (d, *J* = 11.2 Hz, 1H, H_5).

SFC (OJ-H, CO_2/CH_3OH = 80:20, 2.0 mL/min, 298 K, 254 nm): t_R(*syn*, major) = 2.9 min, t_R(*anti*, minor) = 3.3 min, t_R(*anti*, major) = 4.3 min, t_R(*syn*, minor) = 5.1 min, d.r. (*syn:anti*) = 14:1, e.r.(*syn*) = 98:2 (96% *ee*), e.r.(*anti*) = 78:22 (55% *ee*).

HRMS-CI (m/z): $[M + H]^+$ calcd for $C_{21}H_{22}NO_3S2$, 400.1036; found, 400.1017.

R_f = 0.50 (40% ethyl acetate-hexane; UV).

The absolute stereochemistry for (2*R*,3*S*)-**10ad** was confirmed by x-ray crystallography (see *Catalog of X-ray data*). The crystal was grown by a slow evaporation of dichloromethane.

Large Scale reactions:

Synthesis of S-(p-tolyl) (2S,3R)-2-(benzyloxy)-3-hydroxybutanethioate (*S*,*R*)-**10ae**

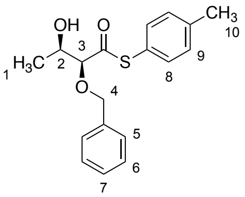

To a suspension of (*S,S*)-**9** (2.00 mmol, 0.77 g, 10 mol%) and activated molecular sieves (4 Å, 13.5 g) in toluene (0.10 M, 200 mL) was added 2-propanol (20.0 mmol, 1.50 mL, 1.00 equiv) followed by a solution of titanium (IV) isopropoxide (0.50 M in toluene; 2.20 mmol, 4.40 mL, 11 mol%). The catalyst mixture was stirred for 1 h at 23 °C. MAHT **8** (8.00 g, 25.3 mmol, 1 equiv) was added to the catalyst mixture in one portion. The resulting red solution was stirred for 15 min at 23 °C followed by addition of acetaldehyde (80.0 mmol, 4.50 mL, 4.00 equiv). The reaction mixture was stirred for 48 h at 23 °C. The product mixture was filtered through a celite and rinsed with ethyl acetate. The filtrate was concentrated and the residue was purified by column chromatography (eluting with 5% ethyl acetate–hexane initially, grading to 30% ethyl acetate–hexane). The product was obtained as a colorless oil (5.16 g, 82%, d.r. (*syn:anti*) = 61:1).

^{1}H NMR (500 MHz, $CDCl_3$) δ 7.44 – 7.29 (m, 5H, H_{5-7}), 7.27 – 7.24 (m, 2H, H_9), 7.22 – 7.18 (m, 2H, H_8), 4.91 (d, *J* = 11.1 Hz, 1H, H_4), 4.54 (d, *J* = 11.1 Hz, 1H, H_4), 4.00 (qd, *J* = 6.4, 5.2 Hz, 1H, H_2), 3.85 (d, *J* = 5.2 Hz, 1H, H_3), 2.34 (s, 3H, H_{10}), 2.26 (brs, 1H, OH), 1.19 (d, *J* = 6.4 Hz, 3H, H_1).

^{13}C NMR (126 MHz, $CDCl_3$) δ 200.9 (C), 139.9 (C), 136.6 (C), 134.7 (2 × CH), 130.3 (2 × CH), 128.7 (2 × CH), 128.60 (2 × CH), 128.56 (CH), 123.4 (C), 88.7 (CH), 74.4 (CH_2), 68.9 (CH), 21.5 (CH_3), 18.7 (CH_3).

SFC (IC-3, CO_2/CH_3OH = 90:10, 2.0 mL/min, 298 K, 254 nm): t_R(*anti*, minor) = 3.1 min, t_R(*anti*, major) = 3.7 min, t_R(*syn*, major) = 4.0 min, t_R(*syn*, minor) = 4.5 min, d.r. (*syn:anti*) = 61:1, e.r.(*syn*) = 98:2 (96% *ee*).

HRMS-CI (m/z): $[M + Na]^+$ calcd for $C_{18}H_{20}O_3SNa$, 339.1025; found, 339.1007.

R_f = 0.35 (20% ethyl acetate-hexane; UV).

$[\alpha]_D^{25}$ = –109.2 (*c* 0.5, $CHCl_3$).

Synthesis of S-(p-tolyl) (2R,3S)-2-(benzyloxy)-3-hydroxybutanethioate (*R*,*S*)-**10ae**

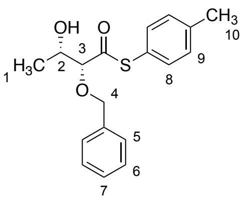

To a suspension of (*R,R*)-**9** (2.53 mmol, 967 mg, 10 mol%) and activated molecular sieves (4 Å, 17.0 g) in toluene (0.10 M, 254 mL) was added 2-propanol (25.3 mmol, 1.90 mL, 1.00 equiv) followed by a solution of titanium (IV) isopropoxide (0.50 M in toluene; 2.78 mmol, 5.60 mL, 11 mol%). The catalyst mixture was stirred for 1 h at 23 °C. MAHT **8** (8.00 g, 25.3 mmol, 1 equiv) was added to the catalyst mixture in one portion. The resulting red solution was stirred for 15 min at 23 °C followed by addition of acetaldehyde (5.70 mL, 101.2 mmol, 4.00 equiv). The reaction mixture was stirred for 48 h at 23 °C. The product mixture was filtered through a celite and rinsed with ethyl acetate. The filtrate was concentrated and the residue was purified by column chromatography (eluting with 5% ethyl acetate–hexane initially, grading to 30% ethyl acetate–hexane). The product was obtained as a colorless oil (7.34 g, 92%, d.r. (*syn:anti*) = 77:1).

^{1}H NMR (500 MHz, $CDCl_3$) δ 7.34 – 7.18 (m, 5H, H_{5-7}), 7.17 – 7.13 (m, 2H, H_9), 7.12 – 7.08 (m, 2H, H_8), 4.81 (d, *J* = 11.1 Hz, 1H, H_4), 4.49 (d, *J* = 11.1 Hz, 1H, H_4), 3.96 – 3.86 (m, 1H, H_2), 3.75 (d, *J* = 5.4 Hz, 1H, H_3), 2.36 (brs, 1H, OH), 2.24 (s, 3H, H_{10}), 1.09 (d, *J* = 6.4 Hz, 3H, H_1).

^{13}C NMR (126 MHz, $CDCl_3$) δ 200.9 (C), 139.9 (C), 136.6 (C), 134.7 (2 × CH), 130.3 (2 × CH), 128.8 (2 × CH), 128.6 (2 × CH), 128.6 (CH), 123.5 (C), 88.7 (CH), 74.5 (CH_2), 68.9 (CH), 21.5 (CH_3), 18.7 (CH_3).

SFC (IC-3, CO_2/CH_3OH = 90:10, 2.0 mL/min, 298 K, 254 nm): t_R(*anti*, major) = 3.1 min, t_R(*anti*, minor) = 3.7 min, t_R(*syn*, minor) = 4.0 min, t_R(*syn*, major) = 4.5 min, d.r. (*syn:anti*) = 77:1, e.r.(*syn*) = 97:3 (94% *ee*).

HRMS-CI (m/z): $[M + Na]^+$ calcd for $C_{18}H_{20}O_3SNa$, 339.1025; found, 339.1007.

R_f = 0.35 (20% ethyl acetate-hexane; UV).

$[\alpha]_D^{25}$ = +116.0 (*c* 0.5, $CHCl_3$).

Synthetic Procedures (transformations):

Synthesis of (2S,3R)-2-(benzyloxy)-3-hydroxybutanoic acid **11**

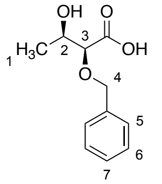

To a solution of (2*S*,3*R*)-**10ae** (90 mg, 0.28 mmol, 1 equiv) in tetrahydrofuran (0.20 M, 1.40 mL) and water (1.00 M, 0.28 mL) was added lithium hydroxide hydrate (14 mg, 0.31 mmol, 1.10 equiv) and hydrogen peroxide (wt.% = 30% in water, 220 µL, 2.84 mmol, 10.0 equiv). The mixture was stirred at 23 °C for 12 h. The product mixture was diluted with saturated aqueous ammonium chloride solution. The organic layer was separated and the aqueous layer was extracted with dichloromethane (2 ×). The organic extracts were discarded and the aqueous layer was acidified to pH 2-3 with aqueous hydrogen chloride solution (1 N). The acidified aqueous layer was extracted with ethyl acetate (2 ×). The combined organic extracts were dried over sodium sulfate, filtered, and the filtrate was concentrated to yield the product **11** as a colorless oil (52 mg, 86%).

^{1}H NMR (500 MHz, $CDCl_3$) δ 7.41 – 7.28 (m, 5H, $H_{5\text{-}7}$), 4.82 (d, *J* = 11.5 Hz, 1H, H_4), 4.49 (d, *J* = 11.5 Hz, 1H, H_4), 4.13 (qd, *J* = 6.4, 3.9 Hz, 1H, H_2), 3.86 (d, *J* = 3.9 Hz, 1H, H_3), 1.27 (d, *J* = 6.4 Hz, 3H, H_1).

^{13}C NMR (126 MHz, $CDCl_3$) δ 175.1 (C), 136.7 (C), 128.7 (2 × CH), 128.5 (2 × CH), 128.5 (CH), 81.3 (CH), 73.3 (CH_2), 68.7 (CH), 19.3 (CH_3).

HRMS-CI (m/z): $[M + Na]^+$ calcd for $C_{11}H_{14}O_4Na$, 233.0784; found, 233.0780.

R_f = 0.28 (70% ethyl acetate-hexane; UV).

$[\alpha]_D^{25}$ = –90.8 (*c* 0.5, $CHCl_3$)

Synthesis of (3S,4R)-3-(benzyloxy)-4-methyloxetan-2-one **12**

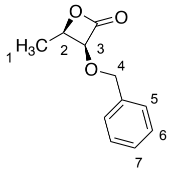

To a solution of (2*S*,3*R*)-**10ae** (115 mg, 0.36 mmol, 1 equiv) in dichloromethane (0.10 M, 3.6 mL) was sequentially added silver trifluoroacetate (AgTFA, 120 mg, 0.55 mmol, 1.50 equiv) and *N,N*-diisopropylethylamine (101 µL, 0.58 mmol, 1.60 equiv). The reaction mixture was stirred for 24 h at 23 °C. The product mixture was filtered through celite eluting with ethyl acetate. The combined organic layers were washed with water, dried over sodium sulfate, filtered, and the filtrate was concentrated. The residue was purified by column chromatography (eluting with 5% ethyl acetate–hexane initially, grading to 40% ethyl acetate–hexane). The product **12** was obtained as a colorless oil (60 mg, 86%).

^{1}H NMR (500 MHz, $CDCl_3$) δ 7.43 – 7.30 (m, 5H, $H_{5\text{-}7}$), 4.94 (d, *J* = 5.6 Hz, 1H, H_3), 4.84 (d, *J* = 11.8 Hz, 1H, H_4), 4.73 (qd, *J* = 6.2, 5.6 Hz, 1H, H_2), 4.66 (d, *J* = 11.8 Hz, 1H, H_4), 1.48 (d, *J* = 6.2 Hz, 3H, H_1).

^{13}C NMR (126 MHz, $CDCl_3$) δ 169.4 (C), 136.4 (C), 128.8 (2 × CH), 128.6 (C), 128.3 (2 × CH), 81.5 (CH), 74.8 (CH), 73.2 (CH_2), 14.8 (CH_3).

HRMS-CI (m/z): $[M + Na]^+$ calcd for $C_{11}H_{12}O_3Na$, 215.0679; found, 215.0675.

R_f = 0.48 (30% ethyl acetate-hexane; UV).

$[\alpha]_D^{25}$ = –74.4 (*c* 0.5, $CHCl_3$).

Synthesis of S-(p-tolyl) (2R,3S)-2,3-dihydroxybutanethioate **13**

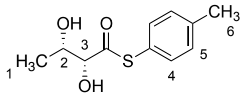

To a solution of (2*R*,3*S*)-**10ae** (100 mg, 0.32 mmol, 1.00 equiv) in dichloromethane (0.05 M, 6.30 mL) was added titanium(IV) chloride (1.00 M in dichloromethane, 632 µL, 0.57 mmol, 2.00 equiv) at 0 °C. The reaction mixture was stirred for 16 h at 0 °C. The product mixture was diluted with saturated aqueous sodium bicarbonate solution and dichloromethane. The organic layer was separated and the aqueous layer was extracted with dichloromethane (2 ×). The combined organic extracts were dried over sodium sulfate, filtered, and the filtrate was concentrated. The residue was purified by column chromatography (eluting with 5% ethyl acetate–hexane initially, grading to 60% ethyl acetate–hexane). The product was obtained as a white solid (64 mg, 89%).

^{1}H NMR (500 MHz, $CDCl_3$) δ 7.32 – 7.28 (m, 2H, H_5), 7.25 – 7.22 (m, 2H, H_4), 4.27 (qd, *J* = 6.5, 2.7 Hz, 1H, H_2), 4.14 (d, *J* = 2.7 Hz, 1H, H_3), 2.38 (s, 3H, H_6), 1.35 (d, *J* = 6.5 Hz, 3H, H_1).

^{13}C NMR (126 MHz, $CDCl_3$) δ 202.0 (C), 140.1 (C), 134.7 (2 × CH), 130.3 (2 × CH), 123.1 (C), 81.1 (CH), 68.9 (CH), 21.5 (CH_3), 19.7 (CH_3).

HRMS-CI (m/z): $[M + Na]^+$ calcd for $C_{11}H_{14}O_3SNa$, 249.0556; found, 249.0548.
R_f = 0.15 (20% ethyl acetate-hexane; UV).
$[\alpha]_D^{25}$ = +94.4 (*c* 0.5, $CHCl_3$).

Synthesis of (2S,3S)-2-(benzyloxy)butane-1,3-diol **14**

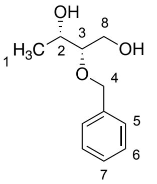

To a solution of (2*R*,3*S*)-**10ae** (115 mg, 0.36 mmol, 1 equiv) in a diethylether (0.05 M, 7.30 mL) was added lithium aluminium hydride ($LiAlH_4$, 1.00 M in Et_2O, 800 µL, 0.80 mmol, 2.20 equiv) at 0 °C. The reaction mixture was stirred for 3 h at 0 °C. The product mixture was diluted with an aqueous sodium hydroxide solution (4 N, 1.00 mL) and ethyl acetate. The crude mixture was filtered through celite eluting with ethyl acetate. The filtrate was concentrated to yield **13** as a colorless oil (68 mg, 95%).

^1H NMR (500 MHz, $CDCl_3$) δ 7.39 – 7.27 (m, 5H, $H_{5\text{-}7}$), 4.72 (d, *J* = 11.5 Hz, 1H, H_4), 4.61 (d, *J* = 11.5 Hz, 1H, H_4), 3.93 (qd, *J* = 6.4, 5.4 Hz, 1H, H_2), 3.85 (dd, *J* = 11.9, 4.1 Hz, 1H, H_8), 3.66 (dd, *J* = 11.9, 4.1 Hz, 1H, H_8), 3.30 (ddd, *J* = 5.4, 4.1, 4.1 Hz, 1H, H_3), 2.50 (brs, 2H, OH), 1.22 (d, *J* = 6.4 Hz, 3H, H_1).
^{13}C NMR (126 MHz, $CDCl_3$) δ 138.0 (C), 128.7 (2 × CH), 128.2 (CH), 128.1 (2 × CH), 83.2 (CH), 73.0 (CH_2), 68.1 (CH), 61.6 (CH_2), 19.2 (CH_3).
HRMS-CI (m/z): $[M + Na]^+$ calcd for $C_{11}H_{16}O_3Na$, 219.0992; found, 219.0984.
R_f = 0.15 (60% ethyl acetate-hexane; UV).
$[\alpha]_D^{25}$ = +28.8 (*c* 0.5, $CHCl_3$).

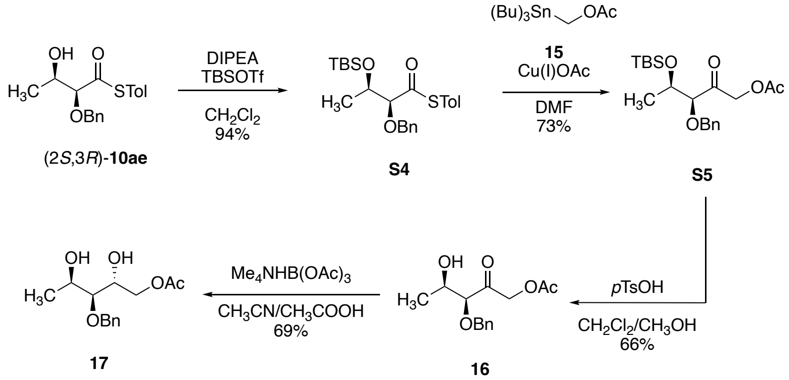

Scheme S3. *Synthesis of acetate* ***17***.

Synthesis of S-(p-tolyl) (2S,3R)-2-(benzyloxy)-3-((tert-butyldimethylsilyl)oxy)butanethioate **S4**

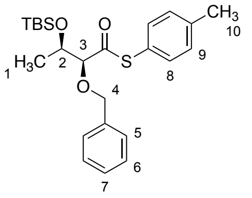

To a solution of (2*S*,3*R*)-**10ae** (295 mg, 0.93 mmol, 1.0 equiv.) in dichloromethane (0.10 M, 10.0 mL) was added *N,N*-diisopropylethylamine (DIPEA, 244 µL, 1.40 mmol, 1.50 equiv) and *t*-butyldimethylsilyl trifluoromethanesulfonate (TBSOTf, 257 µL, 1.12 mmol, 1.20 equiv) at 0 °C. The reaction mixture was stirred for 1 h at 0 °C. The product mixture was diluted with cold water and dichloromethane. The organic layer was separated and the aqueous layer was extracted with dichloromethane (2 ×). The combined organic layers were dried over sodium sulfate, filtered, and the filtrate was concentrated. The product was obtained as a colorless oil (378 mg, 94%).

^1H NMR (500 MHz, $CDCl_3$) δ 7.48 – 7.44 (m, 2H, H_5), 7.41 – 7.31 (m, 3H, $H_{6,7}$), 7.28 (d, *J* = 8.0 Hz, 2H, H_9), 7.23 (d, *J* = 8.0 Hz, 2H, H_8), 4.92 (d, *J* = 11.6 Hz, 1H, H_4), 4.57 (d, *J* = 11.6 Hz, 1H, H_4), 4.15 (qd, *J* = 6.3, 4.8 Hz, 1H, H_2), 3.85 (d, *J* = 4.8 Hz, 1H, H_3), 2.38 (s, 3H, H_{10}), 1.17 (d, *J* = 6.3 Hz, 3H, H_1), 0.89 (s, 9H, TBS), 0.05 (s, 3H, TBS), 0.02 (s, 3H, TBS).
^{13}C NMR (126 MHz, $CDCl_3$) δ 200.7 (C), 139.6 (C), 137.2 (C), 134. 7 (2 × CH), 130.1 (2 × CH), 128.6 (2 × CH), 128.5 (2 × CH), 128.1 (CH), 124.3 (C), 89.4 (CH), 74.2 (CH_2), 69.9 (CH), 26.0 (3 × CH_3), 21.5 (CH_3), 20.1 (CH_3), 18.3 (C), -4.5 (CH_3), -4.6 (CH_3).
HRMS-CI (m/z): $[M + H]^+$ calcd for $C_{24}H_{35}O_3SSi$, 431.2071; found, 431.2045.
R_f = 0.62 (10% ethyl acetate-hexane; UV).
$[\alpha]_D^{25}$ = –110.8 (*c* 0.5, $CHCl_3$).

Synthesis of (3S,4R)-3-(benzyloxy)-4-((tert-butyldimethylsilyl)oxy)-2-oxopentyl acetate **S5**

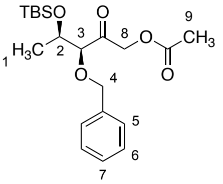

To the solution of **S4** (90 mg, 0.21 mmol, 1 equiv) in *N,N*-dimethylformamide (0.10 M, 2.10 mL) was added (tributylstannyl)methyl acetate **15** (455 mg, 1.25 mmol, 6.00 equiv) and copper(I) acetate (CuOAc, 51 mg, 0.42 mmol, 2.00 equiv). The reaction mixture was stirred for 24 h at 80 °C. The product mixture was diluted with ethyl acetate and filtered through celite eluting with ethyl acetate. The filtrate was concentrated. The residue was purified by column chromatography (eluting with hexane initially, grading to 20% ethyl acetate–hexane). The product was obtained as a colorless oil (58 mg, 73%).

^{1}H NMR (500 MHz, $CDCl_3$) δ 7.40 – 7.27 (m, 5H, $H_{5\text{-}7}$), 5.00 (d, J = 17.5 Hz, 1H, H_8), 4.75 (d, J = 17.5 Hz, 1H, H_8), 4.72 (d, J = 11.8 Hz, 1H, H_4), 4.48 (d, J = 11.8 Hz, 1H, H_4), 4.09 (qd, J = 6.4, 4.4 Hz, 1H, H_2), 3.85 (d, J = 4.4 Hz, 1H, H_3), 2.16 (s, 3H, H_9), 1.14 (d, J = 6.3 Hz, 3H, H_1), 0.87 (s, 9H, TBS), 0.05 (s, 3H, TBS), 0.01 (s, 3H, TBS).

^{13}C NMR (126 MHz, $CDCl_3$) δ 204.5 (C), 170.5 (C), 137.2 (C), 128.6 (2 × CH), 128.3 (CH), 128.2 (2 × CH), 86.8 (CH), 73.5 (CH_2), 69.8 (CH), 68.3 (CH_2), 25.9 (3 × CH_3), 20.6 (CH_3), 19.4 (CH_3), 18.2 (C), -4.6 (CH_3), -4.8 (CH_3).

HRMS-CI (m/z): $[M + H]^+$ calcd for $C_{20}H_{33}O_5Si$, 381.2092; found, 381.2071.

R_f = 0.55 (10% ethyl acetate-hexane; UV).

$[\alpha]_D^{25}$ = –41.2 (*c* 0.5, $CHCl_3$).

Synthesis of (3S,4R)-3-(benzyloxy)-4-hydroxy-2-oxopentyl acetate **16**

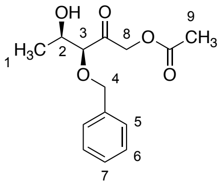

To a solution of **S5** (60 mg, 0.16 mmol, 1 equiv) in dichloromethane (0.10 M, 1.50 mL) and methanol (0.10 M, 1.50 mL) was added *p*-toluenesulfonic acid (*p*TsOH, 6 mg, 0.03 mmol, 20 mol%). The reaction mixture was stirred for 6 h at 23 °C. The product mixture was diluted with water and dichloromethane. The organic layer was separated and the aqueous layer was extracted with dichloromethane (3 ×). The combined organic layers were dried over sodium sulfate, filtered, and the filtrate was concentrated. The residue was purified by column chromatography (eluting with hexane initially, grading to 50% ethyl acetate–hexane). The product was obtained as a colorless oil (28 mg, 66%).

^{1}H NMR (500 MHz, $CDCl_3$) δ 7.43 – 7.28 (m, 5H, $H_{5\text{-}7}$), 4.92 (d, J = 17.7 Hz, 1H, H_3), 4.87 (d, J = 17.7 Hz, 1H, H_4), 4.73 (d, J = 11.5 Hz, 1H, H_8), 4.56 (d, J = 11.5 Hz, 1H, H_8), 4.01 (qd, J = 6.5, 4.1 Hz, 1H, H_2), 3.79 (d, J = 4.1 Hz, 1H, H_3), 2.40 (brs, 1H, OH), 2.16 (s, 3H), 1.23 (d, J = 6.5 Hz, 3H).

^{13}C NMR (126 MHz, $CDCl_3$) δ 205.3 (C), 170.7 (C), 136.6 (C), 128.8 (2 × CH),128.6 (CH), 128.5 (2 × CH), 87.4 (CH), 74.2 (CH_2), 68.6 (CH), 67.8 (CH_2), 20.5 (CH_3), 19.1 (CH_3).

HRMS-CI (m/z): $[M + Na]^+$ calcd for $C_{14}H_{18}NaO_5$, 289.1046; found, 289.1046.

R_f = 0.24 (30 % ethyl acetate-hexane; UV).

$[\alpha]_D^{25}$ = –75.2 (*c* 0.5, $CHCl_3$).

Synthesis of (2R,3S,4R)-3-(benzyloxy)-2,4-dihydroxypentyl acetate **17**

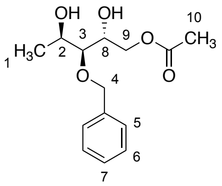

To a solution of tetramethylammonium triacetoxyborohydride ($Me_4NHB(OAc)_3$, 862 mg, 3.28 mmol, 28.5 equiv) in acetonitrile (0.10 M, 1.10 mL) was added acetic acid (0.10 M, 1.10 mL) The reaction mixture was stirred for 30 min and cooled down to –40 °C. A solution of **16** (31 mg, 0.12 mmol, 1 equiv) in acetonitrile (0.20 M, 0.20 mL) was added dropwise. The reaction mixture was stirred for 18 h at –40 °C and warmed up to 23 °C. The reaction mixture was stirred for 2 h at 23 °C. The product mixture was added onto a mixture of ice and saturated aqueous sodium bicarbonate. The product mixture was diluted with aqueous sodium hydroxide (2 N) at 0 °C and extracted with dichloromethane (5 ×). The combined organic layers were dried over sodium sulfate, filtered, and the filtrate was concentrated. The residue was purified by column chromatography (eluting with 20% ethyl acetate–hexane initially, grading to 80% ethyl acetate–hexane). The product **17** was obtained as a colorless oil (21 mg, 69%, mixture of d.r. 92:8). *#

^{1}H NMR (500 MHz, $CDCl_3$) δ 7.39 – 7.27 (m, 5H, $H_{5\text{-}7}$), 4.68 – 4.58 (m, 2H, H_4), 4.33 (dd, J = 11.7, 3.0 Hz, 1H, H_9), 4.17 (dd, J = 11.7, 6.3 Hz, 1H, H_9), 4.06 – 4.03 (m, 2H, $H_{2,8}$), 3.38 (dd, J = 6.7, 3.2 Hz, 1H, H_3), 3.19 (brs, 1H, OH), 2.68 (brs, 1H, OH), 2.08 (s, 3H, H_{10}), 1.29 (d, J = 6.5 Hz, 3H, H_1).

^{13}C NMR (126 MHz, $CDCl_3$) δ 171.7 (C), 137.7 (C), 128.7 (2 × CH), 128.3 (CH), 128.2 (2 × CH), 80.9 (CH), 74.1 (CH_2), 70.4 (CH), 67.4 (CH), 66.3 (CH_2), 21.0 (CH_3), 19.4 (CH_3).
HRMS-CI (m/z): $[M + H]^+$ calcd for $C_{14}H_{20}NaO_5$, 291.1203; found, 291.1197.
R_f = 0.45 (60% ethyl acetate-hexane; UV).
$[\alpha]_D^{25}$ = +26.4 (*c* 0.5, $CHCl_3$).
* The 1,3-*anti* relationship between the two unprotected hydroxyl groups in **17** was confirmed by formation of the acetonide **17a**.
# Although the minor signals in the ^1H NMR spectrum have been tentatively assigned to the minor 1,3-*syn* diastereomer, this assignment cannot be definitively confirmed. The observed diastereomeric ratio (d.r.) aligns with values reported for similar systems.[7]

Synthesis of ((4R,5S,6R)-5-(benzyloxy)-2,2,6-trimethyl-1,3-dioxan-4-yl)methyl acetate **17a**

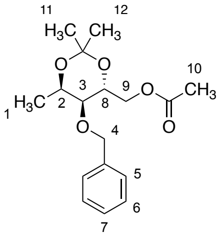

To a solution of **17** (21.8 mg, 0.08 mmol, 1.00 equiv) in dichloromethane (0.10 M, 0.80 mL) was added 2,2-Dimethoxypropane (2,2-DMP, 20 µL, 0.16 mmol, 2.00 equiv), 10-camphorsulfonic acid (CSA, 4 mg, 0.02 mmol, 20 mol%) at 0 °C. The reaction mixture was stirred for 1 h 0 °C. The product mixture was diluted with saturated aqueous sodium bicarbonate solution and dichloromethane, organic layer was separated, aqueous layer was extracted with dichloromethane (2 ×). The combined organic layers were dried over sodium sulfate, filtered and the filtrate was concentrated. The residue was purified by column chromatography (eluting with hexane initially, grading to 40 % ethyl acetate–hexane). The product was obtained as a colorless oil (20 mg, 81%).
^1H NMR (500 MHz, $CDCl_3$) δ 7.41 – 7.26 (m, 5H, H_{5-7}), 4.61 (d, *J* = 11.5 Hz, 1H, H_4), 4.46 (d, *J* = 11.5 Hz, 1H, H_4), 4.13 (dd, *J* = 11.9, 2.9 Hz, 1H, H_3), 4.09 – 4.01 (m, 2H, $H_{2,8}$), 3.88 – 3.81 (m, 1H, H_9), 3.44 (dd, *J* = 7.4, 3.9 Hz, 1H, H_9), 2.05 (s, 3H, H_{10}), 1.39 (s, 3H, $H_{11/12}$), 1.34 (s, 3H, $H_{11/12}$), 1.29 (d, *J* = 6.6 Hz, 3H, H_1).
^{13}C NMR (126 MHz, $CDCl_3$) δ 171.0 (C), 137.9 (C), 128.6 (2 × CH), 128.13 (2 × CH), 128.09 (CH), **101.0 (C)**, 78.5 (CH), 74.2 (CH_2), 71.0 (CH), 67.3 (CH), 64.8 (CH_2), **24.7 (CH_3), 24.2 (CH_3)**, 21.0 (CH_3), 15.3 (CH_3).
R_f = 0.42 (20% ethyl acetate-hexane; UV).
$[\alpha]_D^{25}$ = +32.4 (*c* 0.50, $CHCl_3$).
HRMS-CI (m/z): $[M + H]^+$ calcd for $C_{17}H_{25}O_5$, 309.1697; found, 309.1674.
Analysis of the ^{13}C NMR of this acetonide allowed us to determine the chemical shift of the two methyl groups (δ = 24.2 and 24.7 ppm) and of the ketalic carbon (δ = 101.0 ppm), which are in agreement with a *anti* relationship between the substituents. [8,9]

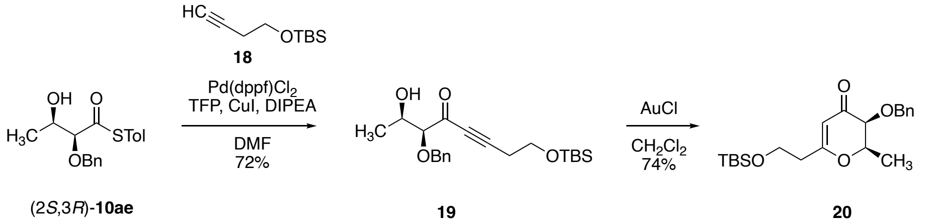

Scheme S4. *Synthesis of dihydropyran* ***20***.

Synthesis of (2R,3S)-3-(benzyloxy)-8-((tert-butyldimethylsilyl)oxy)-2-hydroxyoct-5-yn-4-one **19**

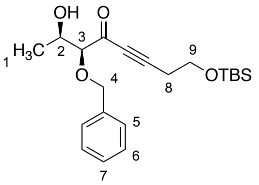

To a solution of (2*S*,3*R*)-**10ae** (100 mg, 0.32 mmol, 1 equiv) in degassed (freeze-pump-thaw, 3 cycles) *N,N*-dimethylformamide (0.44 M, 0.72 mL) under argon were added [1,1′-bis(diphenylphosphino)-ferrocene]dichloropalladium(II) (Pd(dppf)Cl_2, 46 mg, 0.06 mmol, 20 mol%), tri(2-furyl)phosphine (TFP, 29 mg, 0.13 mmol, 40 mol%) and copper(I) iodide (120 mg, 0.63 mmol, 2.00 equiv), followed by *N,N*-diisopropylethylamine (DIPEA, 56 µL, 0.29 mmol, 1.01 eq) and **18** (117 mg, 0.63 mmol, 2.00 equiv). The reaction mixture was stirred for 48 h at 50 °C. After cooling down to 23 °C, celite was added to the solution and the resulting suspension was stirred for 5 min. To the heterogenous mixture was added ethyl acetate and a saturated aqueous sodium chloride solution. The heterogenous mixture was filtered through celite and the celite plug was washed with ethyl acetate. The combined filtrate was dried

over sodium sulfate, filtered, and the filtrate was concentrated. The obtained residue was purified by column chromatography (eluting with hexane initially, grading to 30% ethyl acetate-hexane) to yield **17** as a yellow oil (85 mg, 72%).

^{1}H NMR (500 MHz, $CDCl_3$) δ 7.41 – 7.29 (m, 5H, H_{5-7}), 4.81 (d, *J* = 11.4 Hz, 1H, H_4), 4.45 (d, *J* = 11.4 Hz, 1H, H_4), 4.16 (qd, *J* = 6.4, 5.1 Hz, 1H, H_2), 3.78 (t, *J* = 6.8 Hz, 2H, H_9), 3.74 (d, *J* = 5.1 Hz, 1H, H_3), 2.62 (t, *J* = 6.8 Hz, 2H, H_8), 2.39 (brs, 1H, OH), 1.23 (d, *J* = 6.4 Hz, 3H, H_1), 0.89 (s, 9H, TBS), 0.07 (s, 6H, TBS).

^{13}C NMR (126 MHz, $CDCl_3$) δ 187.7 (C), 137.0 (C), 128.7 (2 × CH), 128.41 (2 × CH), 128.36 (CH), 95.6 (C), 88.8 (C), 80.5 (CH), 73.1 (CH_2), 68.1 (CH), 60.8 (CH_2), 25.9 (3 × CH_3), 23.8 (C), 19.2 (CH_2), 18.4 (CH_3), -5.2 (2 × CH_3).

HRMS-CI (m/z): $[M + NH_4]^+$ calcd for $C_{21}H_{36}NO_4Si$, 394.2408; found, 394.2406.

R_f = 0.33 (20% ethyl acetate-hexane; UV).

$[\alpha]_D^{25}$ = –70.8 (*c* 0.5, $CHCl_3$).

Synthesis of (2R,3S)-3-(benzyloxy)-6-(2-((tert-butyldimethylsilyl)oxy)ethyl)-2-methyl-2,3-dihydro-4H-pyran-4-one **20**

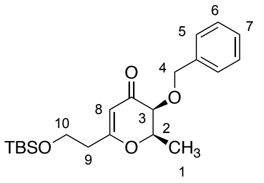

To a solution of **19** (45 mg, 0.12 mmol, 1 equiv) in dichloromethane (0.10 M, 1.20 mL) was added gold(I) chloride (1 mg, 0.01 mmol, 5 mol%) under argon. The reaction mixture was stirred 20 h at 23 °C. The suspension was filtered through silica gel eluting with dichloromethane. The filtrate was concentrated and the residue was purified by column chromatography (eluting with hexane initially, grading to 30% ethyl acetate–hexane). The product was obtained as a colorless oil (33 mg, 74%).

^{1}H NMR (500 MHz, $CDCl_3$) δ 7.38 – 7.26 (m, 5H, H_{5-7}), 5.37 (s, 1H, H_8), 4.77 (d, *J* = 12.0 Hz, 1H, H_4), 4.53 (d, *J* = 12.0 Hz, 1H, H_4), 4.38 (qd, *J* = 6.7, 2.6 Hz, 1H, H_2), 3.87 – 3.76 (m, 2H, H_{10}), 3.47 – 3.42 (m, 1H, H_3), 2.50 – 3.42 (m, 2H, H_9), 1.45 (d, *J* = 6.7 Hz, 3H, H_1), 0.86 (s, 9H, TBS), 0.03 (s, 6H, TBS).

^{13}C NMR (126 MHz, $CDCl_3$) δ 191.0 (C), 175.1 (C), 137.6 (C), 128.5 (2 × CH), 128.4 (2 × CH), 128.0 (CH), 103.3 (CH), 78.0 (CH), 75.9 (CH), 72.0 (CH_2), 59.8 (CH2), 38.6 (CH_2), 26.0 (3 × CH_3), 18.3 (C), 15.2 (CH_3), -5.29 (CH_3), -5.35 (CH_3).

HRMS-CI (m/z): $[M + Na]^+$ calcd for $C_{21}H_{33}O_4Si$, 377.2143; found, 377.2138.

R_f = 0.32 (20% ethyl acetate-hexane; UV).

$[\alpha]_D^{25}$ = +2.4 (*c* 0.5, $CHCl_3$).

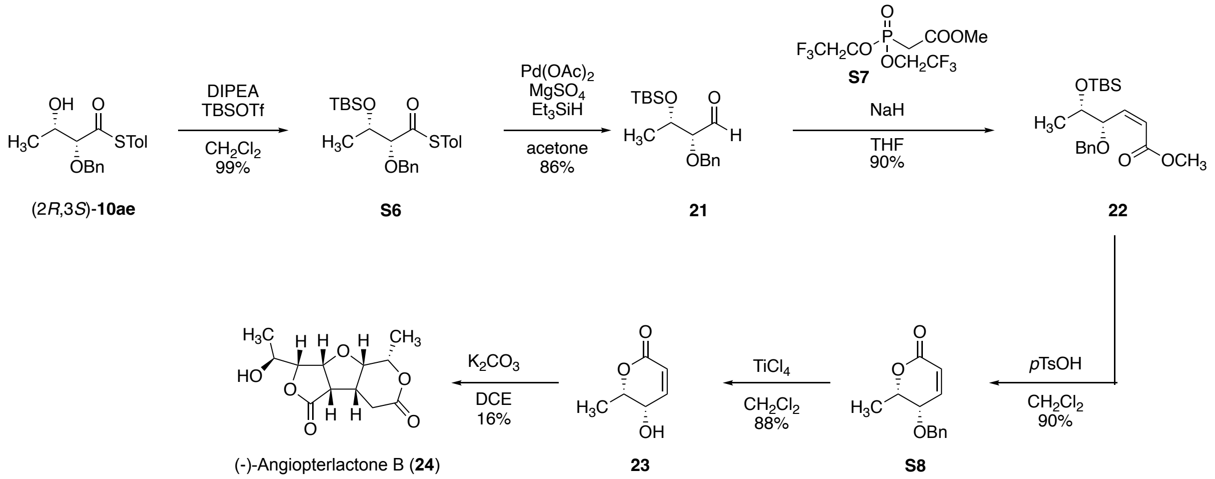

Scheme S5. *Synthesis of (–)-Angiopterlactone B (**24**)*

Synthesis of S-(p-tolyl) (2R,3S)-2-(benzyloxy)-3-((tert-butyldimethylsilyl)oxy)butanethioate **S6**

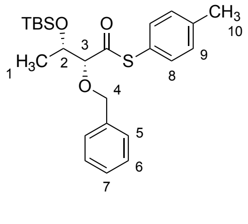

To a solution of (2*R*,3*S*)-**10ae** (650 mg, 2.05 mmol, 1 equiv) in dichloromethane (0.10 M, 20.5 mL) was added *N,N*-diisopropylethylamine (DIPEA, 537 µL, 3.08 mmol, 1.50 equiv) and *t*-butyldimethylsilyl trifluoromethane-sulfonate (TBSOTf, 566 µL, 2.47 mmol, 1.20 equiv) at 0 °C. The reaction mixture was stirred for 1 h at 0 °C. The product mixture was diluted with cold water and dichloromethane. The organic layer was separated and the aqueous layer was extracted with dichloromethane (2 ×). The combined organic

layers were dried over sodium sulfate, filtered, and the filtrate was concentrated. The product was obtained as a colorless oil (873 mg, 99%).

^{1}H NMR (500 MHz, $CDCl_3$) δ 7.48 – 7.43 (m, 2H, H_5), 7.42 – 7.32 (m, 3H, $H_{6,7}$), 7.29 (d, *J* = 8.0 Hz, 2H, H_9), 7.24 (d, *J* = 8.0 Hz, 2H, H_8), 4.92 (d, *J* = 11.6 Hz, 1H, H_4), 4.57 (d, *J* = 11.6 Hz, 1H, H_4), 4.15 (qd, *J* = 6.3, 4.8 Hz, 1H, H_2), 3.85 (d, *J* = 4.8 Hz, 1H, H_3), 2.39 (s, 3H, H_{10}), 1.17 (d, *J* = 6.3 Hz, 3H, H_1), 0.90 (s, 9H, TBS), 0.06 (s, 3H, TBS), 0.03 (s, 3H, TBS).

^{13}C NMR (126 MHz, $CDCl_3$) δ 200.7 (C), 139.6 (C), 137.2 (C), 134. 7 (2 × CH), 130.1 (2 × CH), 128.6 (2 × CH), 128.5 (2 × CH), 128.1 (CH), 124.3 (C), 89.4 (CH), 74.2 (CH_2), 69.9 (CH), 26.0 (3 × CH_3), 21.5 (CH_3), 20.1 (CH_3), 18.3 (C), -4.5 (CH_3), -4.6 (CH_3).

HRMS-CI (m/z): $[M + H]^+$ calcd for $C_{24}H_{35}O_3SSi$, 431.2071; found, 431.2044.

R_f = 0.62 (10% ethyl acetate-hexane; UV).

$[\alpha]_D^{25}$ = +104.8 (*c* 0.5, $CHCl_3$).

Synthesis of (2R,3S)-2-(benzyloxy)-3-((tert-butyldimethylsilyl)oxy)butanal **21**

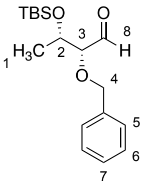

To a solution of **S6** (850 mg, 1.97 mmol, 1 equiv) in acetone (0.03 M, 69.5 mL) was added magnesium sulfate ($MgSO_4$, 3.56 g, 29.6 mmol, 15.0 equiv) and palladium acetate ($Pd(OAc)_2$, 133 mg, 0.59 mmol, 30 mol%) at 0 °C. Triethylsilane (Et_3SiH, 3.10 mL, 19.7 mmol, 10.0 equiv) was added dropwise and stirring was continued for 2 h at 0 °C. The reaction mixture was diluted with methanol and filtered through celite eluting with acetone. The filtrate was concentrated and the residue was purified by column chromatography (eluting with hexane initially, grading to 10% ethyl acetate–hexane). The product was obtained as a colorless oil (522 mg, 86%).

^{1}H NMR (500 MHz, $CDCl_3$) δ 9.77 (d, *J* = 1.5 Hz, 1H, H_8), 7.38 – 7.27 (m, 5H, H_{5-7}), 4.76 (d, *J* = 12.0 Hz, 1H, H_4), 4.54 (d, *J* = 12.0 Hz, 1H, H_4), 4.20 – 4.10 (m, 1H, H_2), 3.73 (dd, *J* = 5.0, 1.5 Hz, 1H, H_3), 1.21 (d, *J* = 6.3 Hz, 3H, H_1), 0.87 (s, 9H, TBS), 0.05 (s, 3H, TBS), 0.03 (s, 3H, TBS).

^{13}C NMR (126 MHz, $CDCl_3$) δ 203.6 (C), 137.5 (C), 128.6 (2 × CH), 128.12 (CH), 128.10 (2 × CH), 86.5 (CH), 72.9 (CH_2), 69.2 (CH), 25.8 (3 × CH_3), 19.6 (CH_3), 18.1 (C), -4.5 (CH_3), -4.9 (CH_3).

HRMS-CI (m/z): $[M + H]^+$ calcd for $C_{17}H_{29}O_3Si$, 309.1880; found, 309.1853.

R_f = 0.38 (10% ethyl acetate-hexane; UV).

$[\alpha]_D^{25}$ = +18.8 (*c* 0.5, $CHCl_3$).

Synthesis of methyl (4S,5S,Z)-4-(benzyloxy)-5-((tert-butyldimethylsilyl)oxy)hex-2-enoate **22**

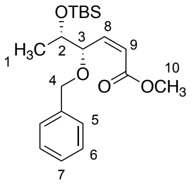

To a solution of methyl 2-(bis(2,2,2-trifluoroethoxy)phosphoryl)acetate **S7** (2.95 g, 9.29 mmol, 2.00 equiv) in tetrahydrofuran (0.08 M, 63 mL) was added sodium hydride (NaH, 60% dispersion in mineral oil, 372 mg, 9.29 mmol, 2.00 equiv) at 0 °C. The reaction mixture was stirred for 30 min at 0 °C and cooled down to −78 ºC, A solution of aldehyde (1.43 g, 4.64 mmol, 1 equiv) in tetrahydrofuran (0.16 M, 30.0 mL) was added to the reaction mixture and stirred for 2 h at −78 °C. The product mixture was diluted with saturated aqueous ammonium chloride solution and ethyl acetate. The organic layer was separated and the aqueous layer extracted with ethyl acetate (2 ×). The combined organic layers were dried over sodium sulfate, filtered, and the filtrate was concentrated. The residue was purified by column chromatography (eluting with hexane initially, grading to 30% ethyl acetate–hexane). The *Z*-olefin product **22** was obtained as a colorless oil (1.52 g, 90%).

^{1}H NMR (500 MHz, $CDCl_3$) δ 7.39 – 7.18 (m, 5H, H_{5-7}), 6.18 (dd, *J* = 11.9, 9.3 Hz, 1H, H_9), 5.98 (dd, *J* = 11.9, 1.1 Hz, 1H, H_8), 4.96 (ddd, *J* = 9.3, 5.1, 1.1 Hz, 1H, H_3), 4.58 (d, *J* = 12.0 Hz, 1H, H_4), 4.46 (d, *J* = 12.0 Hz, 1H, H_4), 3.94 (qd, *J* = 6.4, 5.1 Hz, 1H, H_2), 3.69 (s, 3H, H_{10}), 1.17 (d, *J* = 6.4 Hz, 3H, H_1), 0.87 (s, 9H, TBS), 0.04 (s, 3H, TBS), 0.01 (s, 3H, TBS).

^{13}C NMR (126 MHz, $CDCl_3$) δ 166.4 (C), 147.6 (CH), 138.7 (C), 128.3 (2 × CH), 127.9 (2 × CH), 127.6 (CH), 122.5 (CH), 78.2 (CH), 71.6 (CH_2), 70.7 (CH), 51.4 (CH), 26.0 (3 × CH_3), 19.5 (CH_3), 18.3 (C), -4.59 (CH_3), -4.61 (CH_3).

HRMS-CI (m/z): $[M + H]^+$ calcd for $C_{20}H_{33}O_4Si$, 365.2143; found, 365.2132.

R_f = 0.65 (10% ethyl acetate-hexane; UV).

$[\alpha]_D^{25}$ = −16.4 (*c* 0.5, $CHCl_3$).

Synthesis of (5S,6S)-5-(benzyloxy)-6-methyl-5,6-dihydro-2H-pyran-2-one **S8**

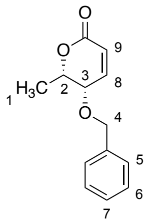

To a solution of **22** (560 mg, 1.54 mmol, 1 equiv) in dichloromethane (0.10 M, 15.4 mL) was added *p*-toluenesulfonic acid (*p*TsOH, 58 mg, 0.31 mmol, 20 mol%). The reaction mixture was stirred for 24 h at 23 °C and diluted with saturated sodium bicarbonate solution and dichloromethane. The organic layer was separated and the aqueous layer was extracted with dichloromethane (2 ×). The combined organic layers were dried over sodium sulfate, filtered, and the filtrate was concentrated. The residue was purified by column chromatography (eluting with hexane initially, grading to 50% ethyl acetate–hexane). The product was obtained as a colorless oil (302 mg, 90%).

^{1}H NMR (500 MHz, $CDCl_3$) δ 7.40 – 7.29 (m, 5H, H_{5-7}), 6.89 (dd, *J* = 9.8, 5.0 Hz, 1H, H_8), 6.13 (d, *J* = 9.8 Hz, 1H, H_9), 4.64 (d, *J* = 11.8, 1H, H_4), 4.59 – 4.56 (m, 2H, $H_{2,4}$), 3.93 (dd, *J* = 5.0, 3.4 Hz, 1H, H_3), 1.49 (d, *J* = 6.7 Hz, 3H, H_1).

^{13}C NMR (126 MHz, $CDCl_3$) δ 163.4 (C), 143.2 (CH), 137.4 (C), 128.7 (2 × CH), 128.3 (CH), 127.9 (2 × CH), 123.5 (CH), 76.6 (CH), 71.6 (CH_2), 68.6 (CH), 15.9 (CH_3).

HRMS-CI (m/z): $[M + H]^+$ calcd for $C_{13}H_{15}O_3$, 219.1016; found, 219.1010.

R_f = 0.4 (30% ethyl acetate-hexane; UV).

$[\alpha]_D^{25}$ = +214.4 (*c* 0.5, $CHCl_3$).

Synthesis of (5S,6S)-5-hydroxy-6-methyl-5,6-dihydro-2H-pyran-2-one **23**

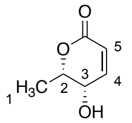

To a solution of **S8** (1.00 g, 4.58 mmol, 1 equiv) in dichloromethane (0.05 M, 91.6 mL) was added titanium(IV) chloride (1.00 M in dichloromethane, 9.20 mL, 9.16 mmol, 2.00 equiv). The reaction mixture was stirred 16 h at 0 °C. The product mixture was diluted with saturated aqueous sodium bicarbonate solution and dichloromethane. The organic layer was separated and the aqueous layer was extracted with dichloromethane (3 ×). The combined organic layers were dried over sodium sulfate, filtered, and the filtrate was concentrated. The residue was purified by column chromatography (eluting with 5% ethyl acetate–hexane initially, grading to 60% ethyl acetate–hexane). The product was obtained as a colorless oil (518 mg, 88%).

^{1}H and ^{13}C NMR data for **23** prepared in this way were in agreement with the literature. The optical rotation was found to be equal to that reported.[10]

^{1}H NMR (500 MHz, $CDCl_3$) δ 7.02 (dd, *J* = 9.7, 5.7 Hz, 1H, H_4), 6.11 (d, *J* = 9.7 Hz, 1H, H_5), 4.54 (qd, *J* = 6.7, 2.7 Hz, 1H, H_2), 4.03 (dd, *J* = 5.7, 2.7 Hz, 1H, H_3), 1.90 (brs, 1H, OH), 1.50 (d, *J* = 6.7 Hz, 3H, H_1).

^{13}C NMR (126 MHz, $CDCl_3$) δ 164.7 (C), 145.1 (CH), 122.3 (CH), 77.5 (CH), 62.8 (CH), 15.8 (CH_3).

HRMS-CI (m/z): $[M + H]^+$ calcd for $C_6H_8O_3$, 129.0546; found, 129.0543.

R_f = 0.28 (70% ethyl acetate-hexane; $KMnO_4$).

$[\alpha]_D^{20}$ = +148.6 (*c* 0.51, H_2O), lit. = +148.0 (*c* 0.51, H_2O).

(–)-Angiopterlactone B (**24**)

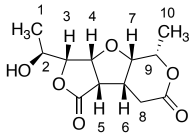

To a solution of **23** (113 mg, 0.88 mmol, 1 equiv) in anhydrous dichloroethane (DCE, 1.50 M, 0.59 mL) was added potassium carbonate (24 mg, 0.18 mmol, 20 mol%). The reaction mixture was stirred for 16 h at 70 °C. The product mixture was diluted with chloroform and filtered through cotton eluting with chloroform. The filtrate was concentrated and the obtained residue was purified by column chromatography (eluting with dichloromethane initially, grading to 8% methanol–dichloromethane). The product was obtained as a white solid (36 mg, 16%).

^{1}H and ^{13}C NMR data for **24** prepared in this way were in agreement with the literature. The optical rotation was found to be equal to that reported.[10]

^{1}H NMR (500 MHz, CD_3OD) δ 4.57 (dd, *J* = 5.3, 3.8 Hz, 1H, H_4), 4.43 (qd, *J* = 6.6, 1.6 Hz, 1H, H_9), 4.21 (dd, *J* = 8.6, 3.8 Hz, 1H, H_3), 4.18 (dd, *J* = 8.7, 1.6 Hz, 1H, H_7), 4.03 (dq, *J* = 8.7, 6.4 Hz, 1H, H_2), 3.53 (dd, *J* = 10.6, 5.3 Hz, 1H, H_5), 3.38 – 3.31 (m, 1H, H_6), 3.12 (dd, *J* = 16.4, 1.0 Hz, 1H, H_8), 2.67 (dd, *J* = 16.4, 9.0 Hz, 1H, H_8), 1.39 (d, *J* = 6.6 Hz, 3H, H_{10}), 1.27 (d, *J* = 6.4 Hz, 3H, H_1).

^{13}C NMR (126 MHz, CD_3OD) δ 176.4 (C), 174.1 (C), 86.6 (CH), 80.3 (CH), 79.8 (CH), 75.2 (CH), 67.4 (CH), 50.2 (CH), 37.8 (CH), 28.8 (CH_2), 18.5 (CH_3), 16.8 (CH_3).

HRMS-CI (m/z): $[M + H]^+$ calcd for $C_{12}H_{17}O_6$, 257.1020; found, 257.1000

R_f = 0.55 (10% methanol-dichloromethane; $KMnO_4$).

$[\alpha]_D^{20}$ = –25.0 (*c* 0.04, ethyl acetate), lit. –25.0 (*c* 0.04, ethyl acetate).

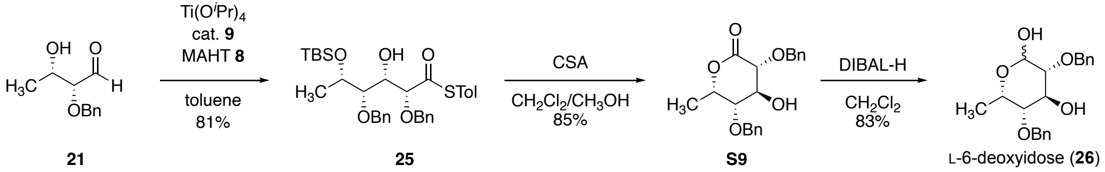

Scheme S6. *Synthesis of L-6-deoxyidose (**26**)*

Synthesis of S-(p-tolyl) (2R,3S,4S,5S)-2,4-bis(benzyloxy)-5-((tert-butyldimethylsilyl)oxy)-3-hydroxyhexanethioate **25**

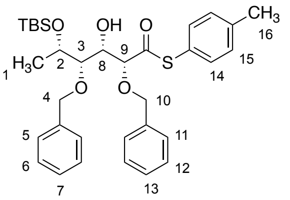

Following the general method, (2*R*,3*S*,4*S*,5*S*)-**25** was prepared from **21** (100 mg, 0.32 mmol, 1 equiv) and MAHT **8** (123 mg, 0.39 mmol, 1.20 equiv). The reaction mixture was stirred for 24 h. The residue was purified by column chromatography (eluting with hexane initially, grading to 20% *tert*-butyl methyl ether–hexane), to yield the product as a colorless oil (152 mg, 81%) as a single diastereomer. *

^{1}H NMR (500 MHz, $CDCl_3$) δ 7.49 – 7.24 (m, 10H, $H_{5-7,11-13}$), 7.23 – 7.15 (m, 4H, $H_{14,15}$), 4.95 (d, *J* = 11.3 Hz, 1H, H_4), 4.70 (d, *J* = 11.5 Hz, 1H, H_{10}), 4.66 (d, *J* = 11.5 Hz, 1H, H_{10}), 4.59 (d, *J* = 11.3 Hz, 1H, H_4), 4.18 (ddd, *J* = 7.6, 5.5, 2.4 Hz, 1H, H_8), 4.14 (d, *J* = 5.5 Hz, 1H, H_9), 3.94 (qd, *J* = 6.3, 5.6 Hz, 1H, H_2), 3.48 (dd, *J* = 5.6, 2.4 Hz, 1H, H_3), 2.95 (d, *J* = 7.6 Hz, 1H, OH), 2.36 (s, 3H, H_{16}), 1.17 (d, *J* = 6.3 Hz, 3H, H_1), 0.84 (s, 9H, TBS), 0.01 (s, 3H, TBS), -0.05 (s, 3H, TBS).

^{13}C NMR (126 MHz, $CDCl_3$) δ 200.8 (C), 139.7 (C), 138.6 (C), 136.8 (C), 134.8 (2 × CH), 130.1 (2 × CH), 128.7 (2 × CH), 128.6 (2 × CH), 128.4 (2 × CH), 127.7 (2 × CH), 127.6 (2 × CH), 123.7 (C), 85.7 (CH), 80.3 (CH), 74.4 (CH_2), 73.5 (CH_2), 70.9 (CH), 68.3 (CH), 26.0 (3 × CH_3), 21.5 (CH), 19.3 (CH), 18.1 (C), -4.4 (CH_3), -4.6 (CH_3).

HRMS-CI (m/z): $[M + H]^+$ calcd for $C_{33}H_{45}O_5SSi$, 581.2751; found, 581.2725.

R_f = 0.22 (20% *tert*-butyl methyl ether-hexane; UV).

$[\alpha]_D^{25}$ = +51.2 (*c* 0.5, $CHCl_3$).

*The stereochemistry of **25** was assigned after analysis of lactone **S8**.

Synthesis of (3R,4S,5S,6S)-3,5-bis(benzyloxy)-4-hydroxy-6-methyltetrahydro-2H-pyran-2-one **S9**

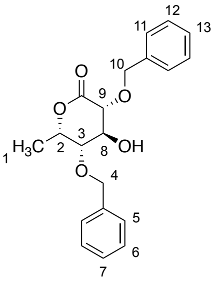

To a solution of **25** (110 mg, 0.19 mmol, 1.0 equiv.) in dichloromethane (0.10 M, 1.90 mL) and methanol (0.10 M, 1.90 mL) was added 10-camphorsulfonic acid (CSA, 9 mg, 0.04 mmol, 20 mol%). The reaction mixture was stirred for 20 h at 50 °C. After cooling down to 23 °C, the product mixture was diluted with a saturated sodium bicarbonate solution and dichloromethane. The organic layer was separated and the aqueous layer was extracted with dichloromethane (2 ×). The combined organic layers were dried over sodium sulfate filtered, and the filtrate was concentrated. The residue was purified by column chromatography (eluting with hexane initially, grading to 50% ethyl acetate–hexane). The product was obtained as a white solid (55 mg, 85%).

^{1}H and ^{13}C NMR data for **S9** prepared in this way were in agreement with the reported enantiomer *(*3*S*,4*R*,5*R*,6*R)*-**S9** from the literature. The optical rotation was found to be equal and opposite to that reported.[11]

^{1}H NMR (500 MHz, $CDCl_3$) δ 7.45 – 7.27 (m, 10H, $H_{5-7,11-13}$), 5.10 (d, *J* = 11.3 Hz, 1H, $H_{4/10}$), 4.75 (d, *J* = 12.0 Hz, 1H, $H_{4/10}$), 4.60 (d, *J* = 11.3 Hz, 1H, $H_{4/10}$), 4.54 (qd, *J* = 6.5, 2.5 Hz, 1H, H_2), 4.49 (d, *J* = 12.0 Hz, 1H, $H_{4/10}$), 4.10 – 4.01 (m, 2H, $H_{8,9}$), 3.54 (dd, *J* = 2.0, 2.0 Hz, 1H, H_3), 2.74 (d, *J* = 2.3 Hz, OH), 1.38 (d, *J* = 6.5 Hz, 3H, H_1).

^{13}C NMR (126 MHz, $CDCl_3$) δ 169.9 (C), 137.2 (C), 137.0 (C), 128.8 (2 × CH), 128.53 (2 × CH), 128.50 (2 × CH), 128.4 (CH), 128.1 (2 × CH), 128.1 (CH), 79.5 (CH), 78.2 (CH), 74.3 (CH), 73.9 (CH), 73.4 (CH_2), 71.3 (CH_2), 16.0 (CH_3).

HRMS-CI (m/z): $[M + H]^+$ calcd for $C_{20}H_{23}O_5$, 343.1540; found, 343.1524

R_f = 0.54 (50% ethyl acetate-hexane; UV).

$[\alpha]_D^{25}$ = +94.8 (*c* 1.5, $CHCl_3$), lit. –95.8 (*c* 1.5, $CHCl_3$).

Synthesis of L-6-deoxyidose (**26**)

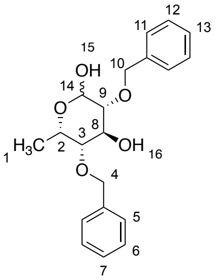

To a solution of **S8** (46 mg, 0.13 mmol, 1 equiv) in dichloromethane (0.08 M, 1.80 mL) was added diisobutylaluminium hydride (DIBAL-H, 1.00 M in toluene, 266 µL, 0.27 mmol, 2.00 equiv) at –78 °C. The reaction mixture was stirred for 2 h at –78 °C. The product mixture was diluted with saturated aqueous ammonium chloride solution and stirring was continued for 20 mins at –78 °C and at 23 °C for another 20 min. The diluted product mixture was filtered through celite eluting with dichloromethane. The organic layer was dried over sodium sulfate, filtered, and the filtrate was concentrated. The residue was purified by column chromatography (eluting with hexane initially, grading to 50% ethyl diethyl ether–hexane). The product was obtained as a white solid (38 mg, 83%, 3:1 mixture of anomers).

^{1}H and ^{13}C NMR data for **26** prepared in this way were in agreement with the reported enantiomer **26** from the literature. The optical rotation was found to be equal and opposite to that reported.[11]

Major anomer:

^{1}H NMR (500 MHz, $CDCl_3$) δ 7.40 – 7.27 (m, 10H, $H_{4\text{-}7,11\text{-}13}$), 5.04 (d, *J* = 5.5 Hz, 1H, H_{14}), 4.84 (d, *J* = 11.7 Hz, 1H, $H_{4/10}$), 4.76 – 4.52 (m, 3H, $H_{4,10}$), 4.28 (qd, *J* = 6.7, 4.5 Hz, 1H, H_2), 3.94 (dd, *J* = 7.8, 7.0 Hz, 1H, H_8), 3.47 (dd, *J* = 7.0, 4.6 Hz, 1H, H_3), 3.30 (dd, *J* = 7.8, 5.5 Hz, 1H, H_9), 2.76 (brs, 1H, H_{16}), 1.27 (d, *J* = 6.7 Hz, 3H, H_1).

^{13}C NMR (126 MHz, $CDCl_3$) δ 138.2 (2 × C), 128.6 (2 × CH), 128.5 (2 × CH), 128.2 (2 × CH), 128.0 (2 × CH), 127.6 (CH), 127.9 (CH), 93.7 (CH), 80.4 (CH), 79.0 (CH), 73.7 (CH_2), 72.8 (CH_2), 70.7 (CH), 67.1 (CH), 14.5 (CH_3).

Minor anomer (detectable non-overlapping resonances):

^{1}H NMR (500 MHz, $CDCl_3$) δ 4.16 (dd, *J* = 4.8, 4.3 Hz, 1H, H_8), 4.05 (qd, *J* = 6.8, 3.0 Hz, 1H, H_2), 3.38 (dd, *J* = 4.3, 3.0 Hz, 1H, H_9), 3.22 (dd, *J* = 4.8, 3.0 Hz, 1H, H_3), 1.33 (d, *J* = 6.8 Hz, 3H, H_1).

^{13}C NMR (126 MHz, $CDCl_3$) δ 138.0 (C), 137.6 (C), 128.7 (2 × CH), 128.6 (2 × CH), 128.5 (2 × CH), 128.32 (CH), 128.30 (2 × CH), 128.1 (CH), 128.0 (2 × CH), 127.9 (2 × CH), 92.2 (CH), 77.7 (CH), 77.5 (CH), 73.3 (CH_2), 73.0 (CH_2), 70.0 (CH), 66.6 (CH), 17.0 (CH_3).

$[\alpha]_D^{25}$ = –3.0 (*c* 0.2, $CHCl_3$), lit. +2.1 (*c* 0.2, $CHCl_3$).

Catalog of SFC Spectra

SFC (IC-3, CO_2/CH_3OH =90:10, 2.0 mL/min, 298 K, 254 nm) for **10a**:

Racemic product:

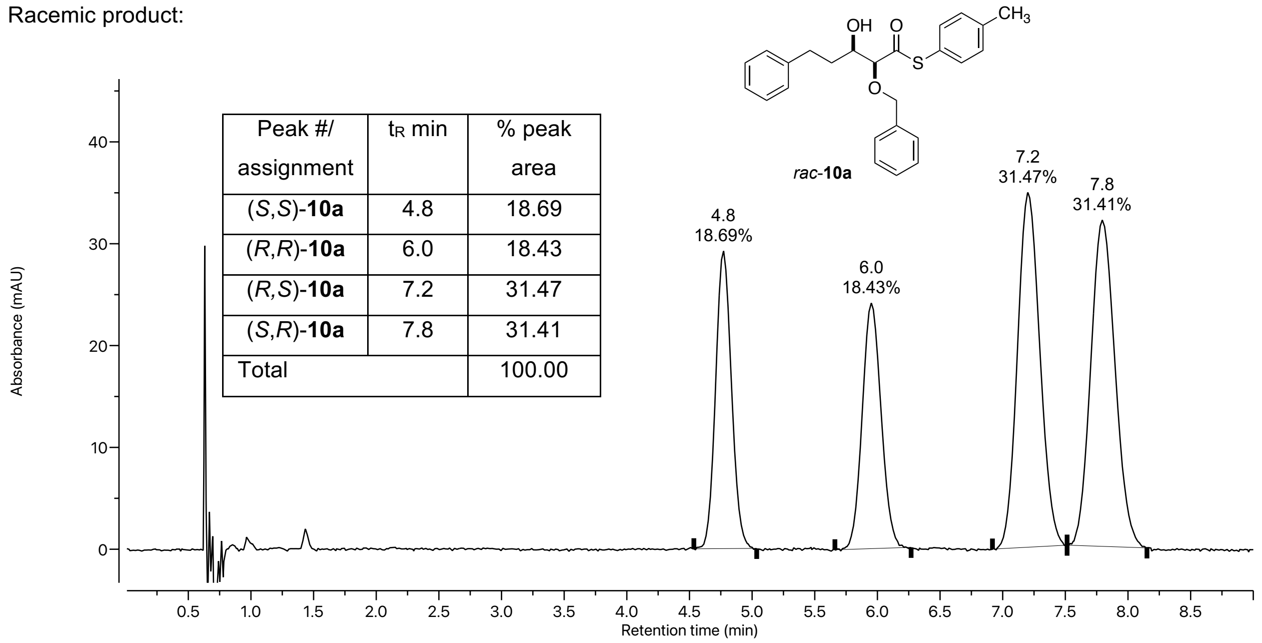

Peak #/ assignment	t_R min	% peak area
(*S*,*S*)-**10a**	4.8	18.69
(*R*,*R*)-**10a**	6.0	18.43
(*R*,*S*)-**10a**	7.2	31.47
(*S*,*R*)-**10a**	7.8	31.41
Total		100.00

syn-product:

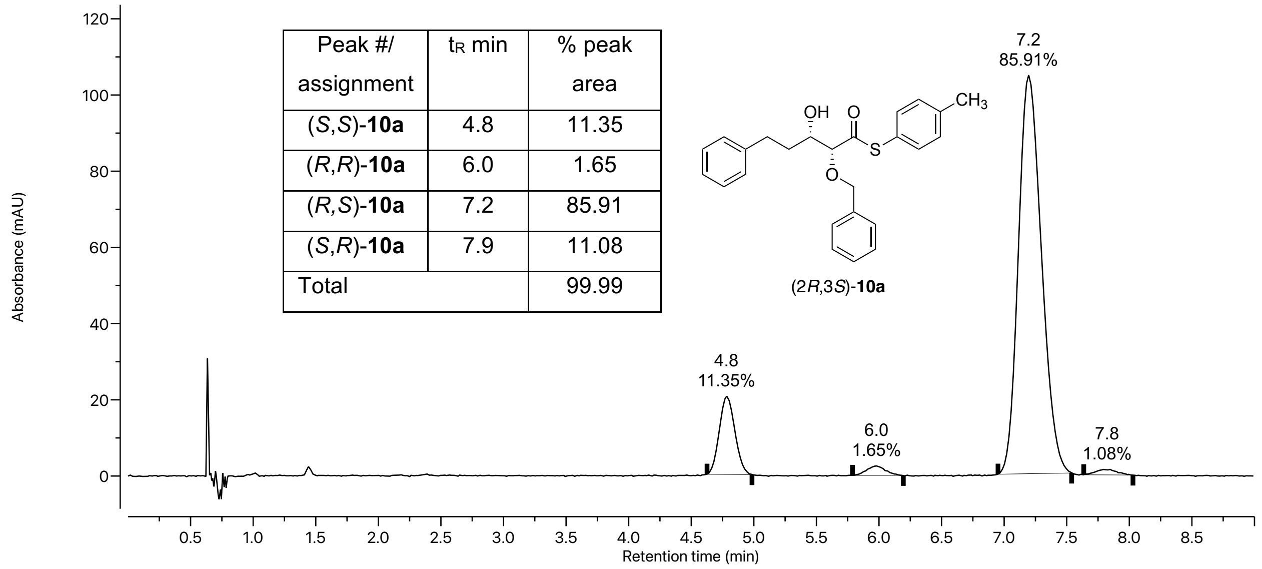

Peak #/ assignment	t_R min	% peak area
(*S*,*S*)-**10a**	4.8	11.35
(*R*,*R*)-**10a**	6.0	1.65
(*R*,*S*)-**10a**	7.2	85.91
(*S*,*R*)-**10a**	7.9	11.08
Total		99.99

syn-product (purified):

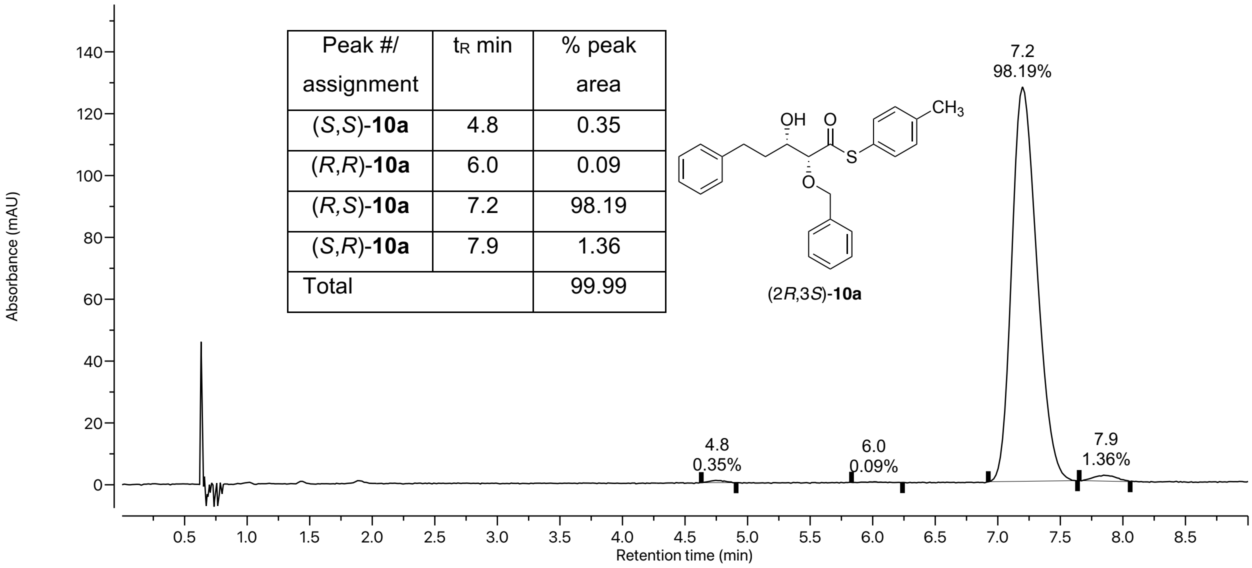

Peak #/ assignment	t_R min	% peak area
(*S*,*S*)-**10a**	4.8	0.35
(*R*,*R*)-**10a**	6.0	0.09
(*R*,*S*)-**10a**	7.2	98.19
(*S*,*R*)-**10a**	7.9	1.36
Total		99.99

SFC (OJ-H, CO_2/CH_3OH =80:20, 2.0 mL/min, 298 K, 254 nm) for **10b**:

Racemic product:

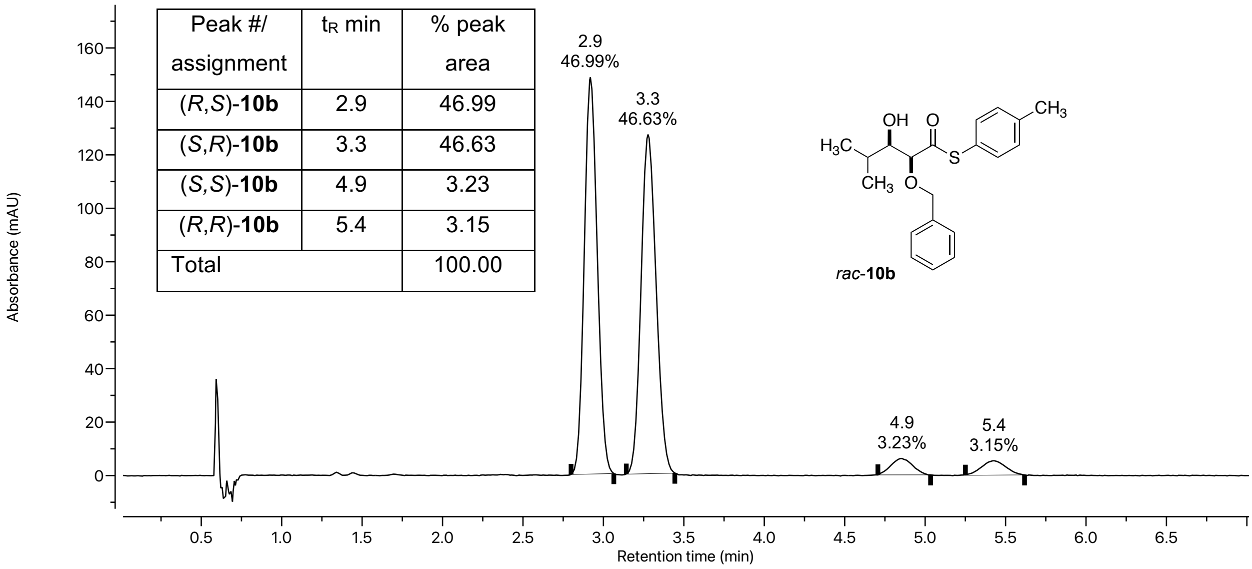

Peak #/ assignment	t_R min	% peak area
(*R*,*S*)-**10b**	2.9	46.99
(*S*,*R*)-**10b**	3.3	46.63
(*S*,*S*)-**10b**	4.9	3.23
(*R*,*R*)-**10b**	5.4	3.15
Total		100.00

syn-product:

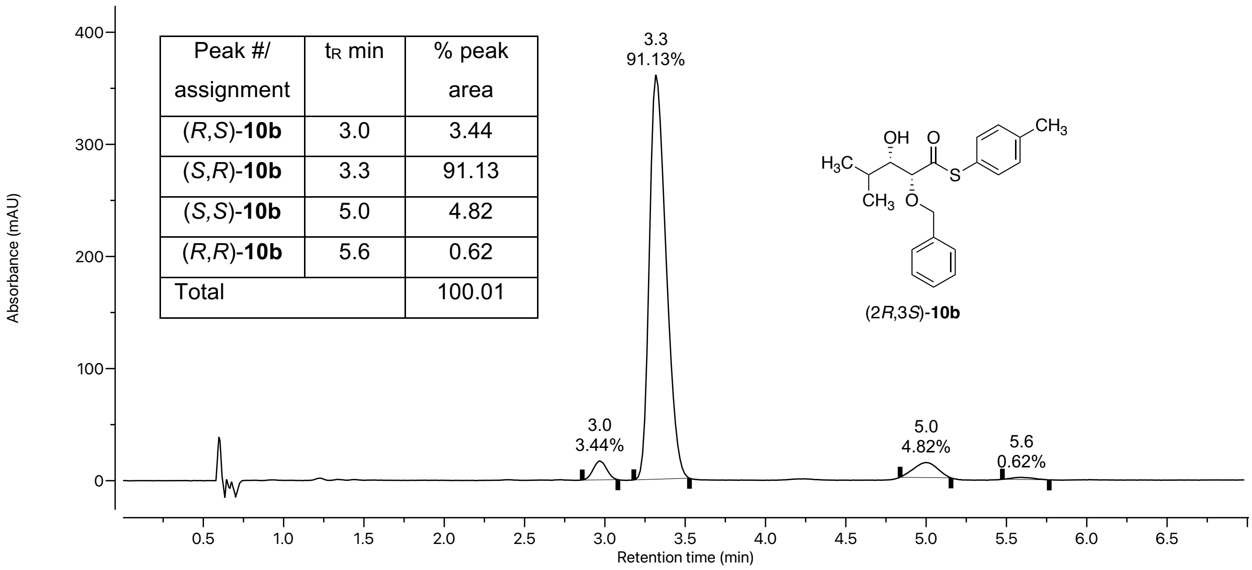

Peak #/ assignment	t_R min	% peak area
(*R*,*S*)-**10b**	3.0	3.44
(*S*,*R*)-**10b**	3.3	91.13
(*S*,*S*)-**10b**	5.0	4.82
(*R*,*R*)-**10b**	5.6	0.62
Total		100.01

SFC (IC-3, CO_2/CH_3OH =80:20, 2.0 mL/min, 298 K, 254 nm) for **10c**:

Racemic product:

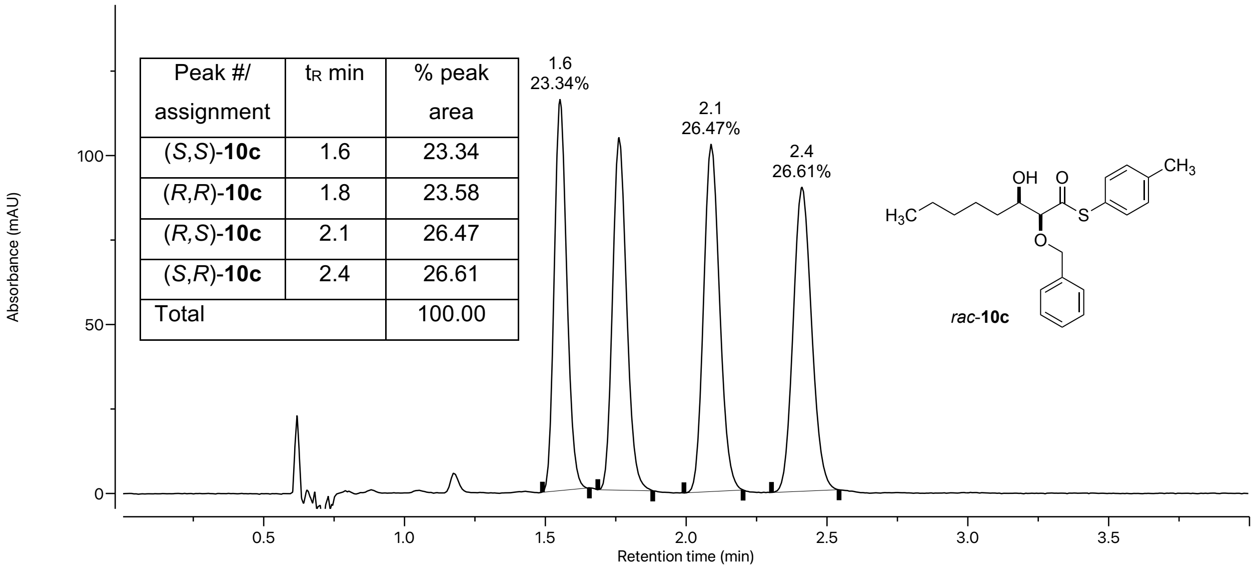

Peak #/ assignment	t_R min	% peak area
(*S*,*S*)-**10c**	1.6	23.34
(*R*,*R*)-**10c**	1.8	23.58
(*R*,*S*)-**10c**	2.1	26.47
(*S*,*R*)-**10c**	2.4	26.61
Total		100.00

syn-product:

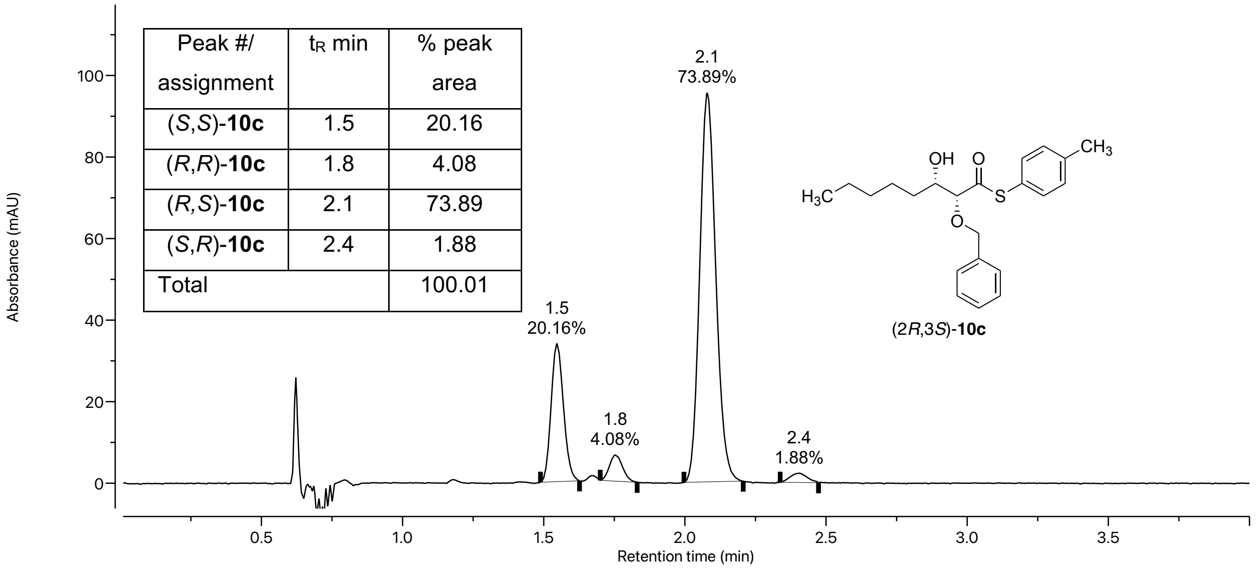

Peak #/ assignment	t_R min	% peak area
(*S*,*S*)-**10c**	1.5	20.16
(*R*,*R*)-**10c**	1.8	4.08
(*R*,*S*)-**10c**	2.1	73.89
(*S*,*R*)-**10c**	2.4	1.88
Total		100.01

SFC (IA-3, CO_2/CH_3OH =90:10, 2.0 mL/min, 298 K, 254 nm) for **10d**:

Racemic product:

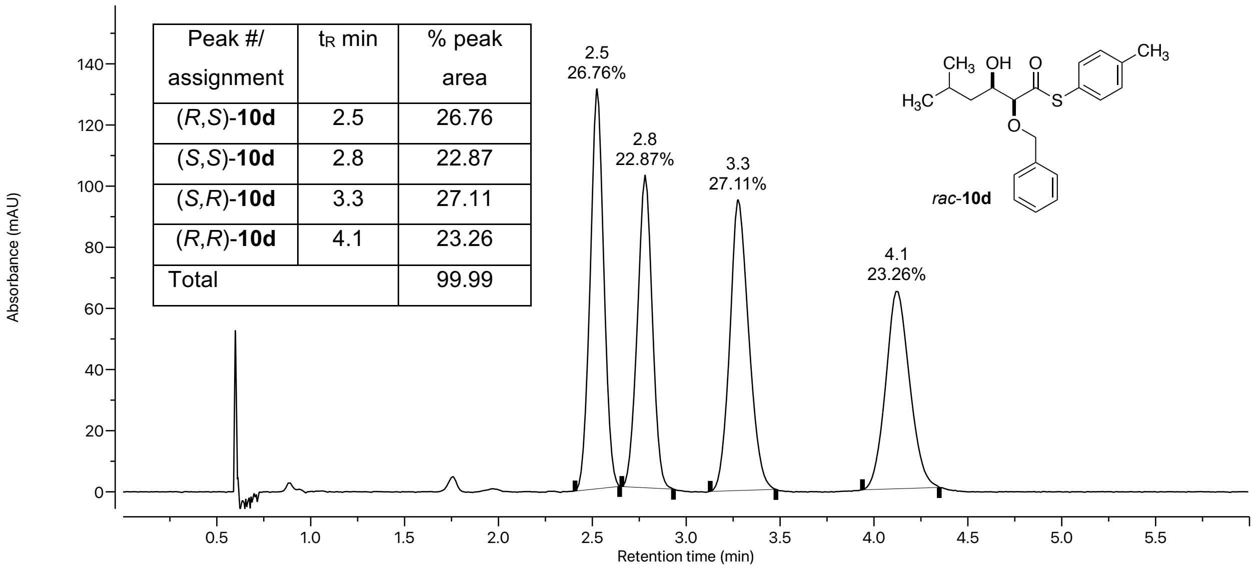

Peak #/ assignment	tR min	% peak area
(*R*,*S*)-**10d**	2.5	26.76
(*S*,*S*)-**10d**	2.8	22.87
(*S*,*R*)-**10d**	3.3	27.11
(*R*,*R*)-**10d**	4.1	23.26
Total		99.99

syn-product:

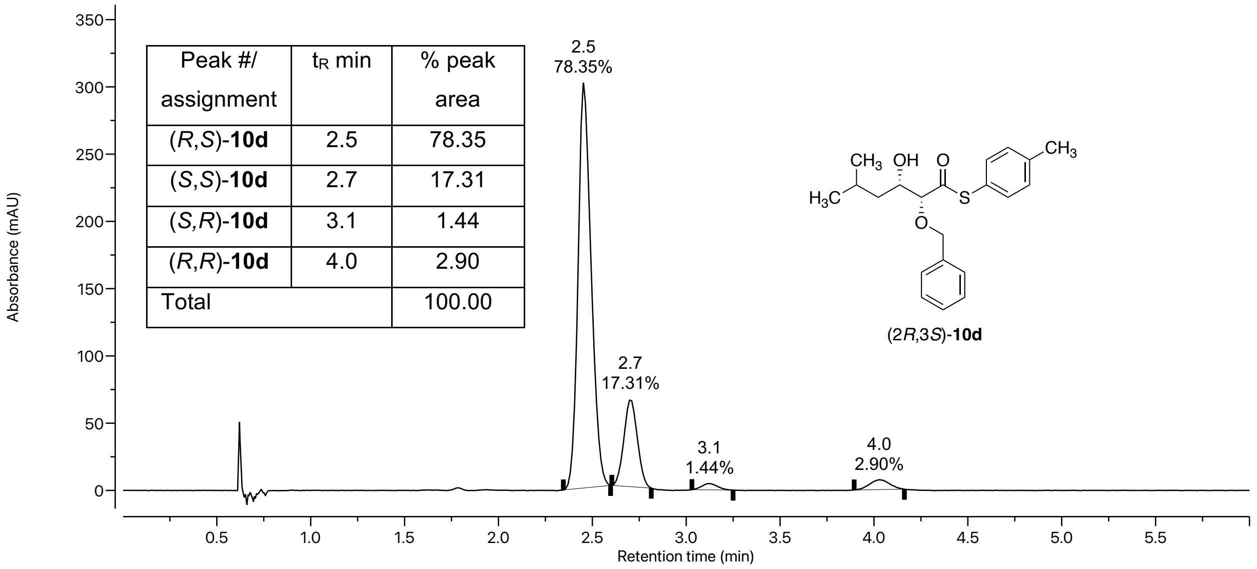

Peak #/ assignment	tR min	% peak area
(*R*,*S*)-**10d**	2.5	78.35
(*S*,*S*)-**10d**	2.7	17.31
(*S*,*R*)-**10d**	3.1	1.44
(*R*,*R*)-**10d**	4.0	2.90
Total		100.00

SFC (IC-3, CO_2/CH_3OH =80:20, 2.0 mL/min, 298 K, 254 nm) for **10e**:

Racemic product:

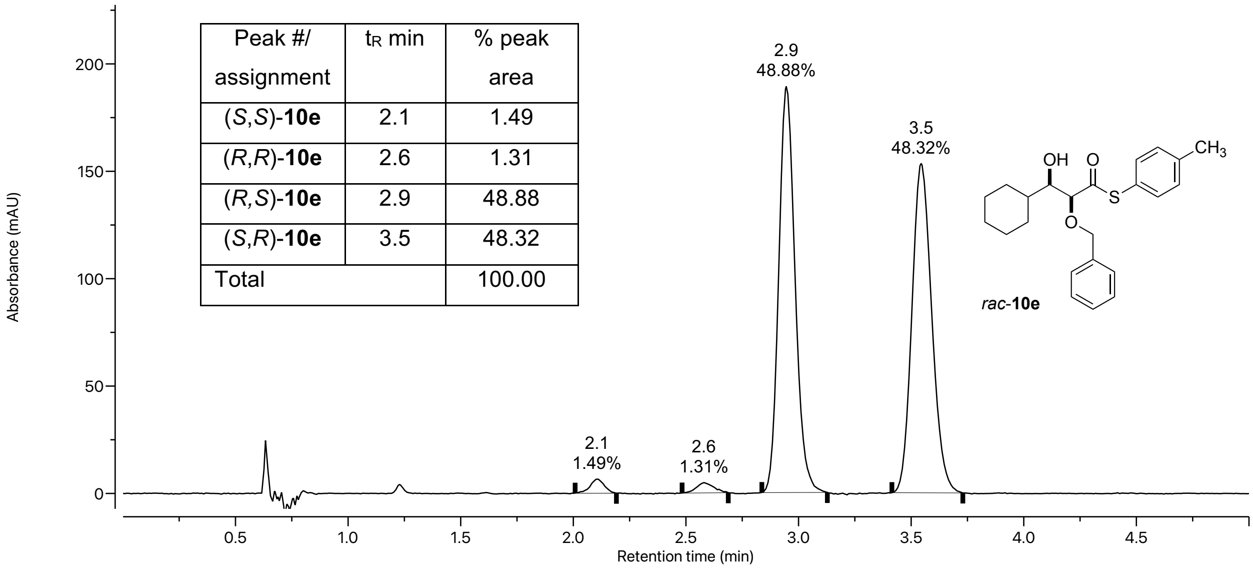

Peak #/ assignment	t_R min	% peak area
(*S*,*S*)-**10e**	2.1	1.49
(*R*,*R*)-**10e**	2.6	1.31
(*R*,*S*)-**10e**	2.9	48.88
(*S*,*R*)-**10e**	3.5	48.32
Total		100.00

syn-product:

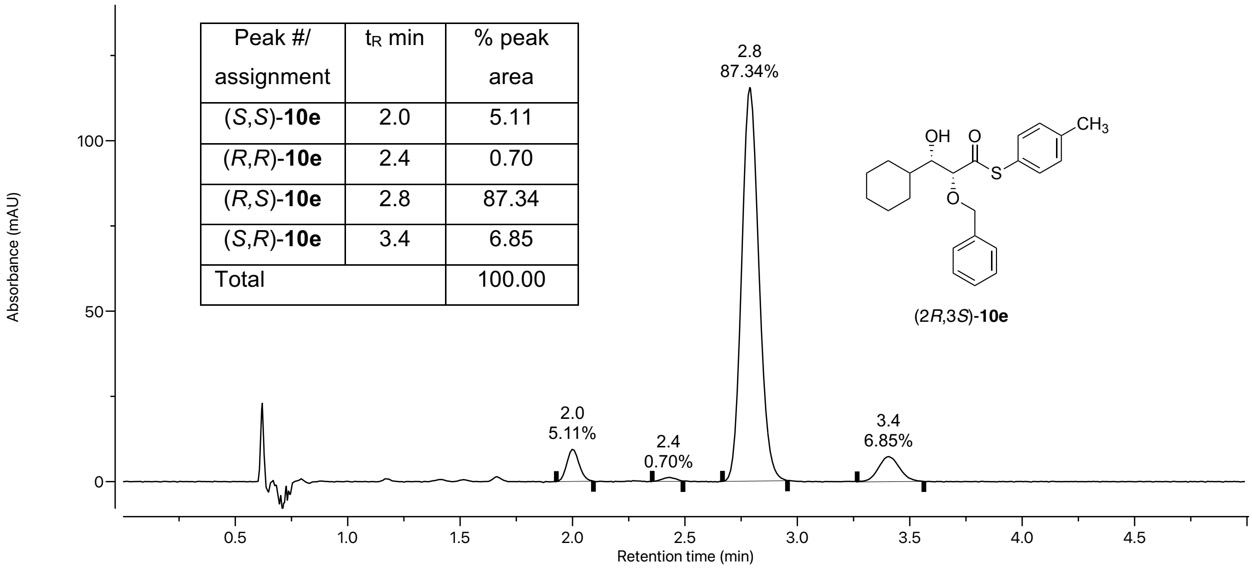

Peak #/ assignment	t_R min	% peak area
(*S*,*S*)-**10e**	2.0	5.11
(*R*,*R*)-**10e**	2.4	0.70
(*R*,*S*)-**10e**	2.8	87.34
(*S*,*R*)-**10e**	3.4	6.85
Total		100.00

SFC (IC-3, CO_2/CH_3OH =90:10, 2.0 mL/min, 298 K, 254 nm) for **10f**:

Racemic product:

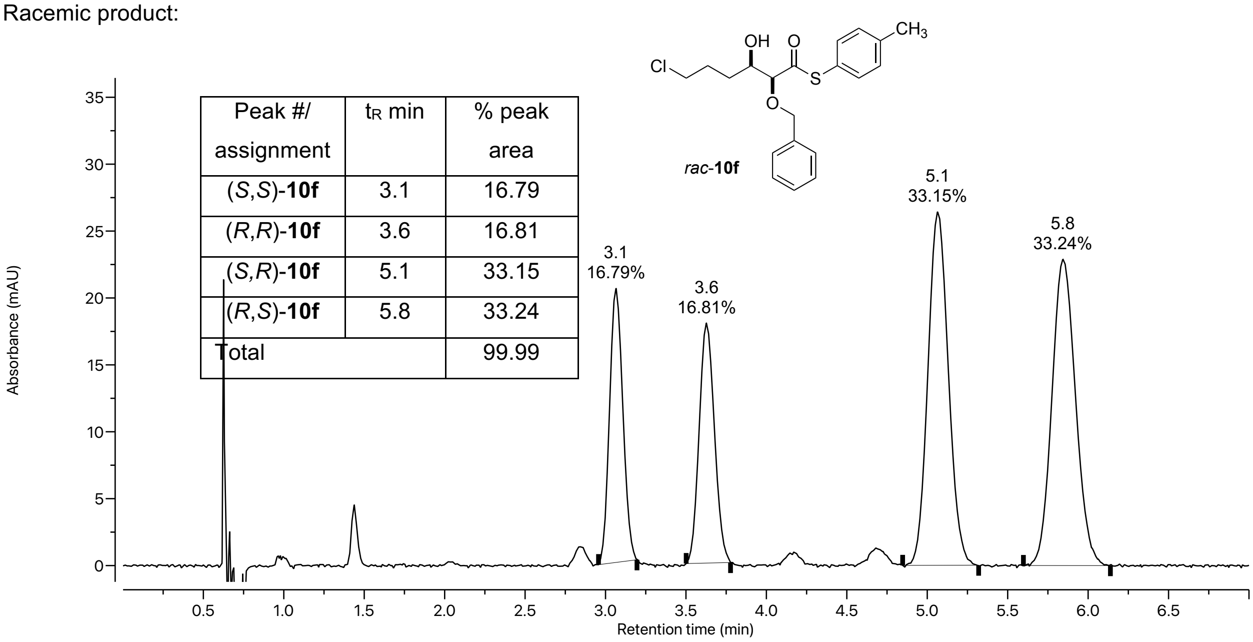

Peak #/ assignment	t_R min	% peak area
(*S*,*S*)-**10f**	3.1	16.79
(*R*,*R*)-**10f**	3.6	16.81
(*S*,*R*)-**10f**	5.1	33.15
(*R*,*S*)-**10f**	5.8	33.24
Total		99.99

syn-product:

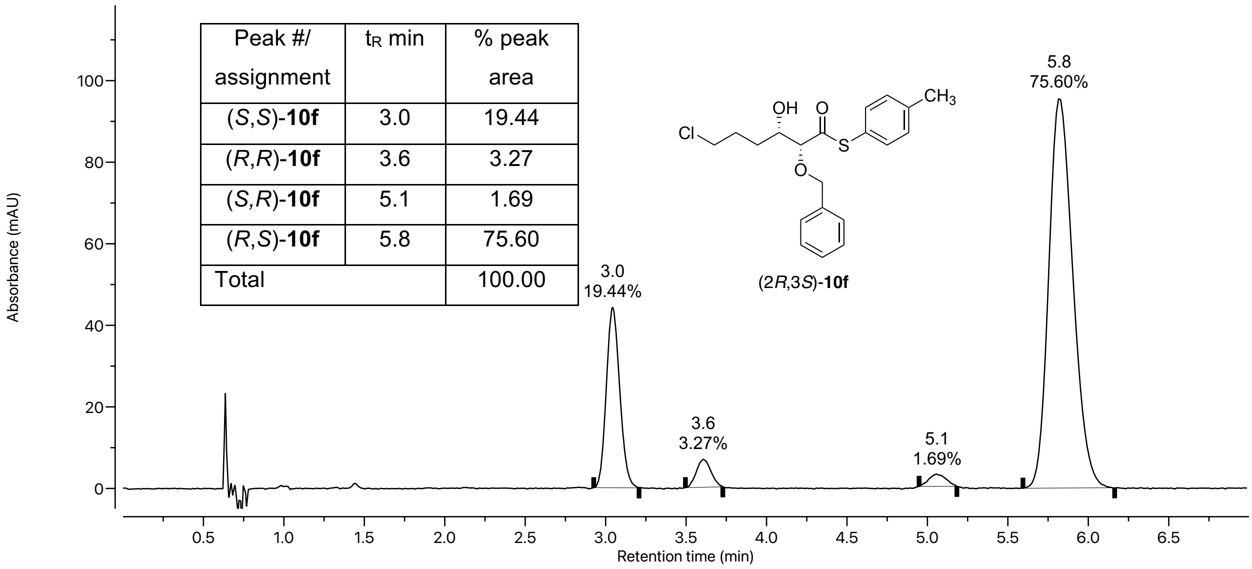

Peak #/ assignment	t_R min	% peak area
(*S*,*S*)-**10f**	3.0	19.44
(*R*,*R*)-**10f**	3.6	3.27
(*S*,*R*)-**10f**	5.1	1.69
(*R*,*S*)-**10f**	5.8	75.60
Total		100.00

SFC (IA-3, CO_2/CH_3OH =94:6, 2.0 mL/min, 298 K, 254 nm) for **10g**:

Racemic product:

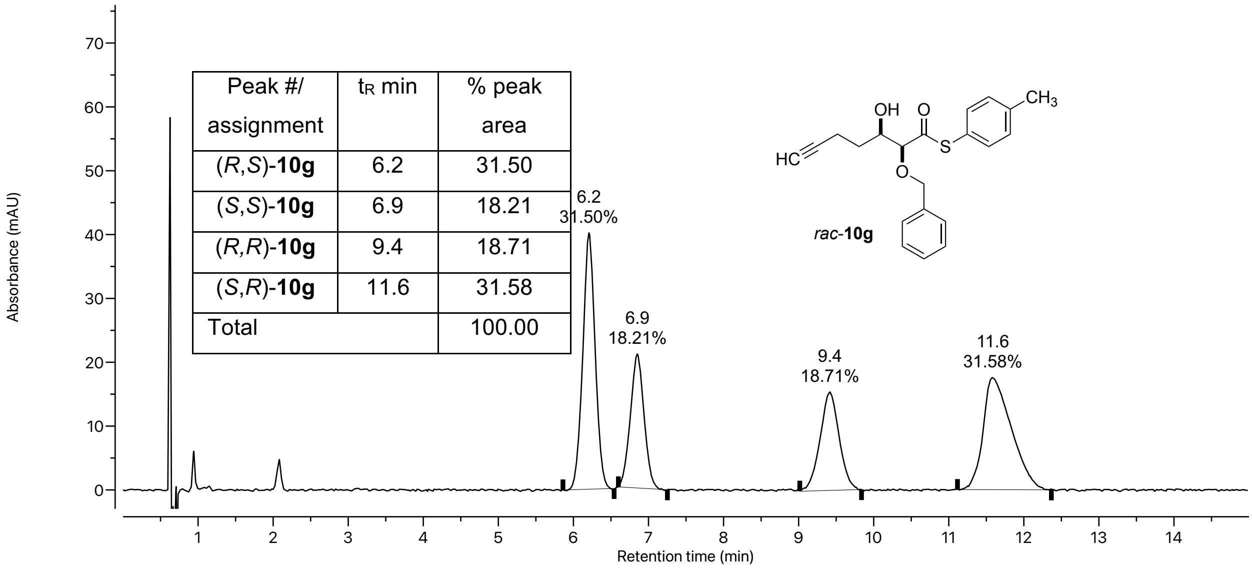

Peak #/ assignment	t_R min	% peak area
(*R*,*S*)-**10g**	6.2	31.50
(*S*,*S*)-**10g**	6.9	18.21
(*R*,*R*)-**10g**	9.4	18.71
(*S*,*R*)-**10g**	11.6	31.58
Total		100.00

syn-product:

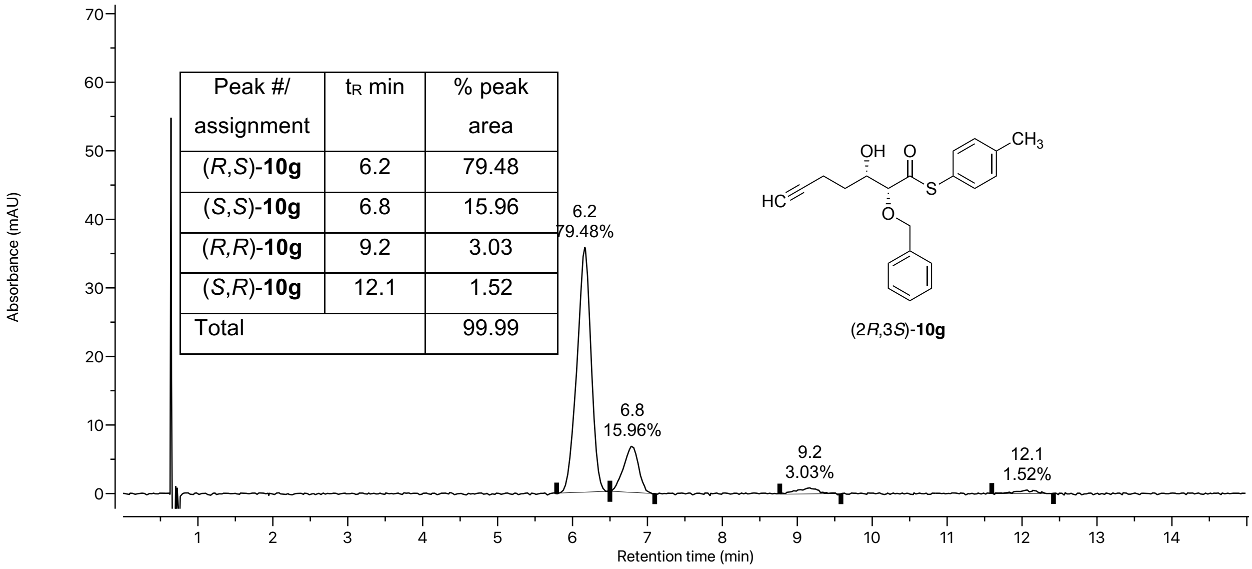

Peak #/ assignment	t_R min	% peak area
(*R*,*S*)-**10g**	6.2	79.48
(*S*,*S*)-**10g**	6.8	15.96
(*R*,*R*)-**10g**	9.2	3.03
(*S*,*R*)-**10g**	12.1	1.52
Total		99.99

SFC (IC-3, CO_2/CH_3OH =90:10, 2.0 mL/min, 298 K, 254 nm) for **10h**:

Racemic product:

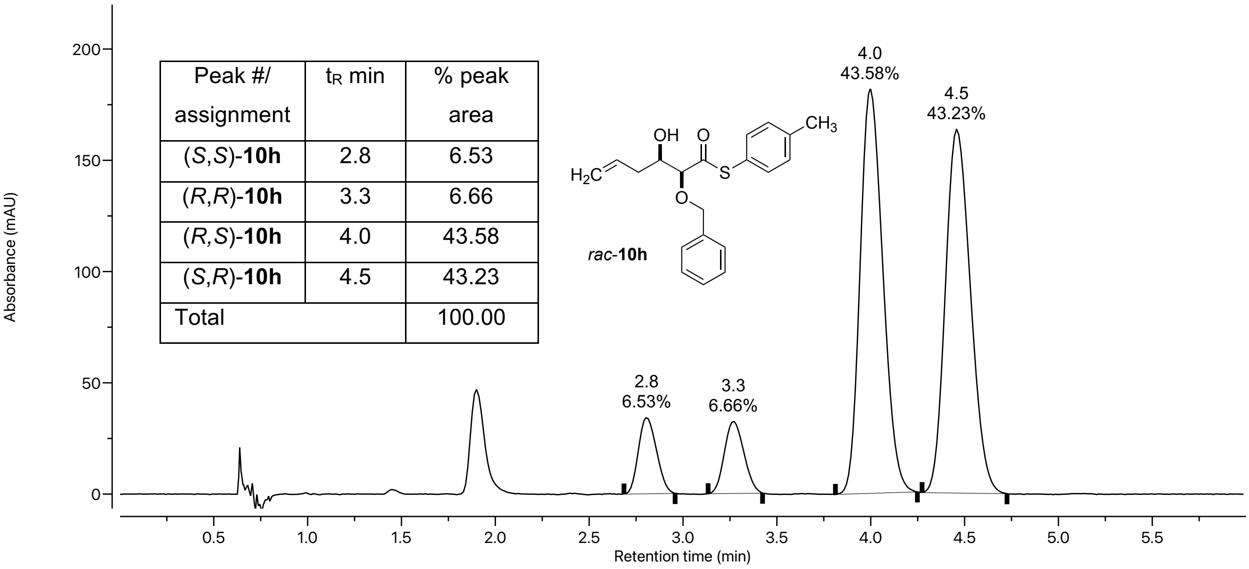

Peak #/ assignment	t_R min	% peak area
(*S*,*S*)-**10h**	2.8	6.53
(*R*,*R*)-**10h**	3.3	6.66
(*R*,*S*)-**10h**	4.0	43.58
(*S*,*R*)-**10h**	4.5	43.23
Total		100.00

syn-product:

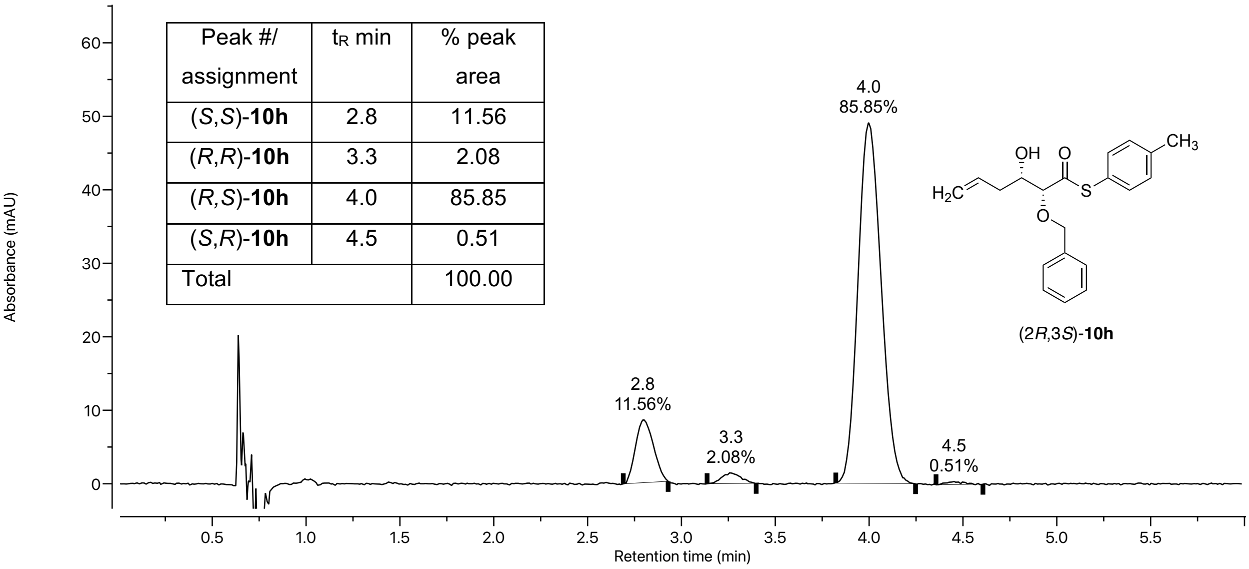

Peak #/ assignment	t_R min	% peak area
(*S*,*S*)-**10h**	2.8	11.56
(*R*,*R*)-**10h**	3.3	2.08
(*R*,*S*)-**10h**	4.0	85.85
(*S*,*R*)-**10h**	4.5	0.51
Total		100.00

SFC (IC-3, CO_2/CH_3OH =94:6, 2.0 mL/min, 298 K, 254 nm) for **10i**:

Racemic product:

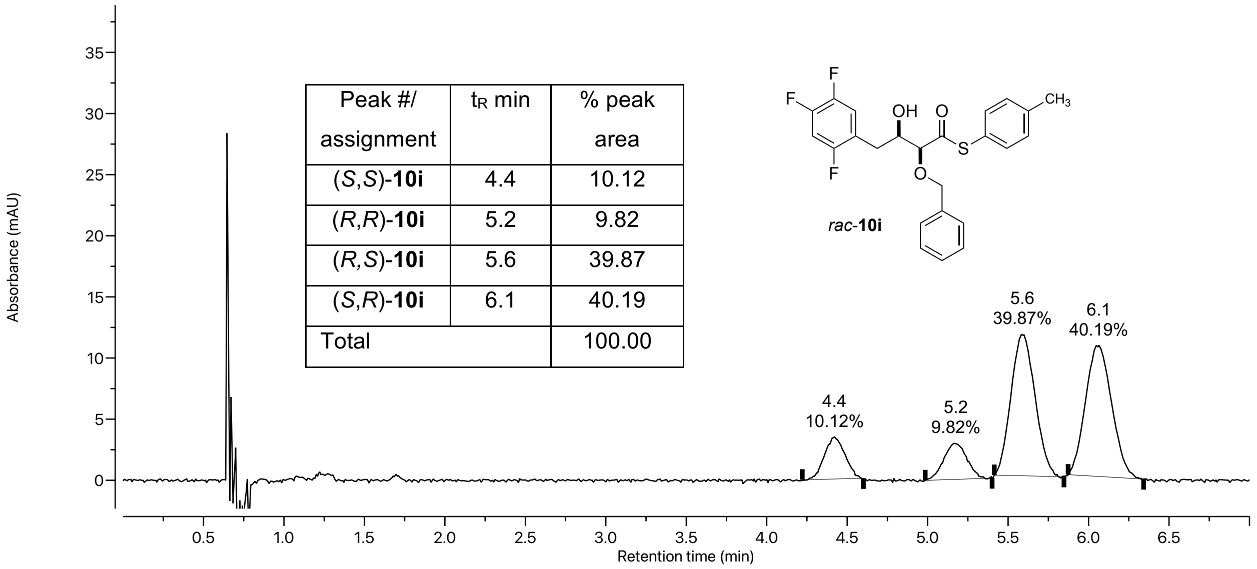

Peak #/ assignment	t_R min	% peak area
(*S*,*S*)-**10i**	4.4	10.12
(*R*,*R*)-**10i**	5.2	9.82
(*R*,*S*)-**10i**	5.6	39.87
(*S*,*R*)-**10i**	6.1	40.19
Total		100.00

syn-product:

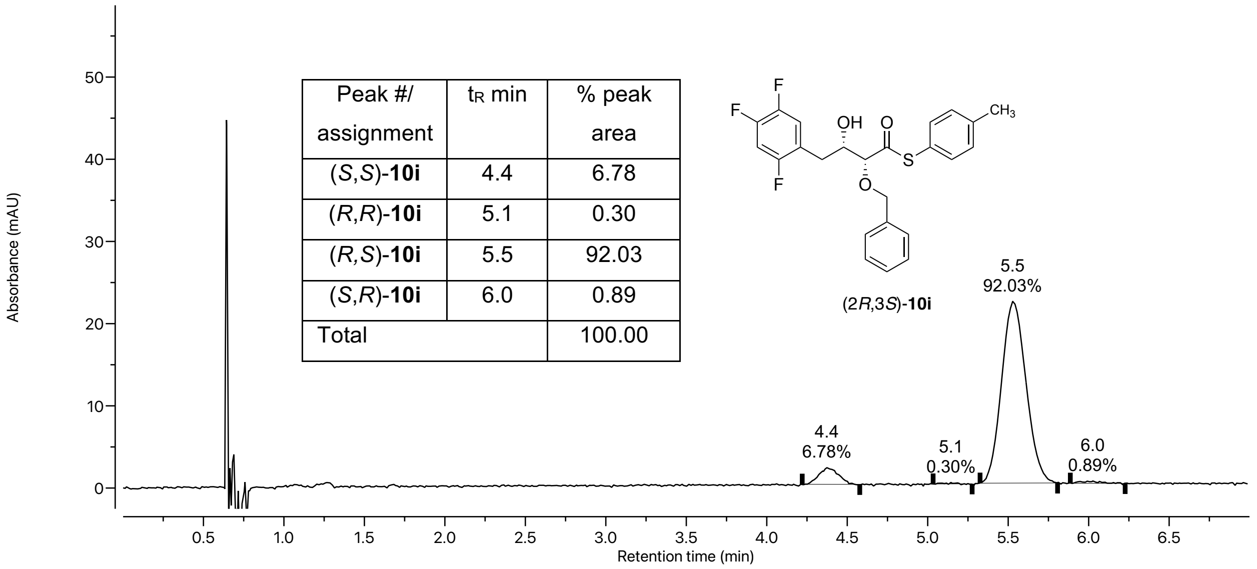

Peak #/ assignment	t_R min	% peak area
(*S*,*S*)-**10i**	4.4	6.78
(*R*,*R*)-**10i**	5.1	0.30
(*R*,*S*)-**10i**	5.5	92.03
(*S*,*R*)-**10i**	6.0	0.89
Total		100.00

SFC (IA-3, CO_2/CH_3OH =80:20, 2.0 mL/min, 298 K, 254 nm) for **10j**:

Racemic product:

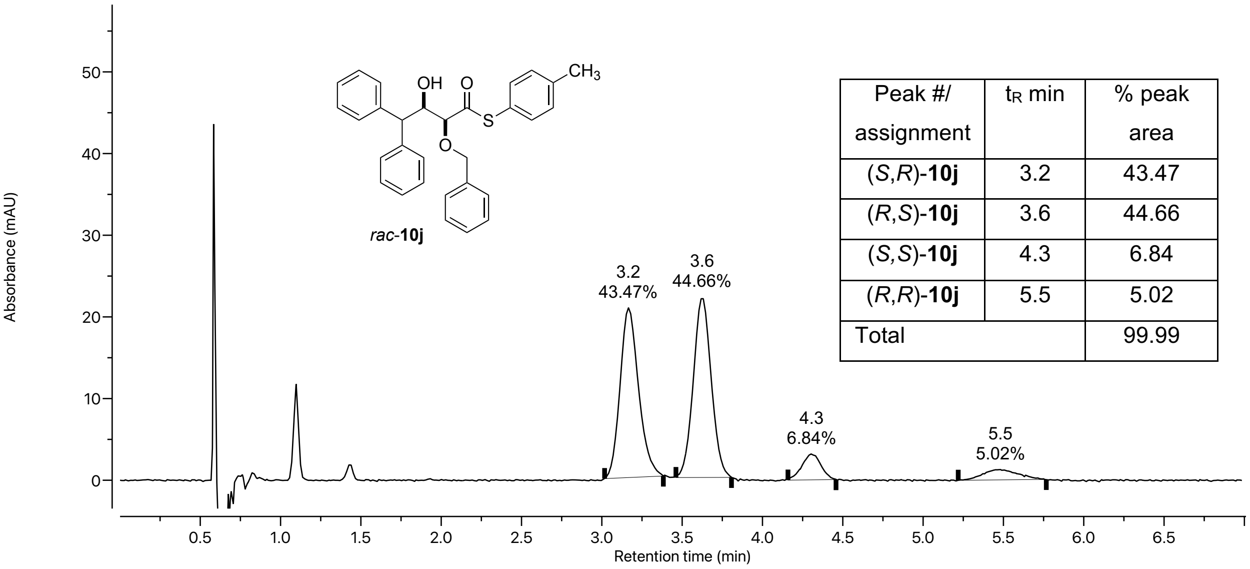

Peak #/ assignment	t_R min	% peak area
(*S*,*R*)-**10j**	3.2	43.47
(*R*,*S*)-**10j**	3.6	44.66
(*S*,*S*)-**10j**	4.3	6.84
(*R*,*R*)-**10j**	5.5	5.02
Total		99.99

syn-product:

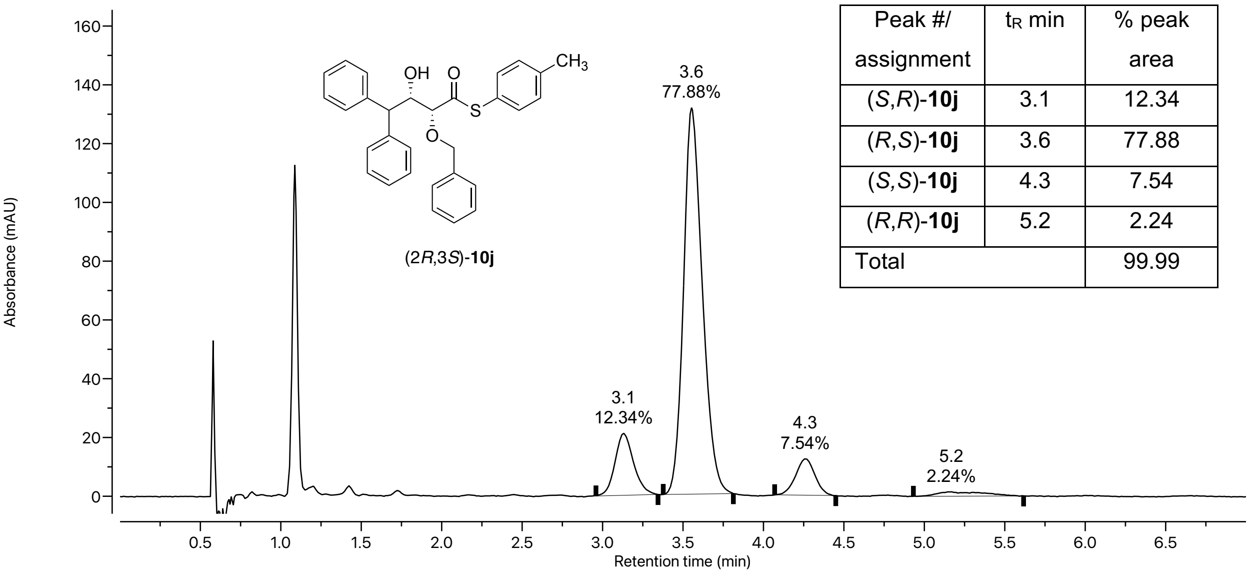

Peak #/ assignment	t_R min	% peak area
(*S*,*R*)-**10j**	3.1	12.34
(*R*,*S*)-**10j**	3.6	77.88
(*S*,*S*)-**10j**	4.3	7.54
(*R*,*R*)-**10j**	5.2	2.24
Total		99.99

SFC (OD-H, CO_2/CH_3OH =94:6, 2.0 mL/min, 298 K, 254 nm) for **10k**:

Racemic product:

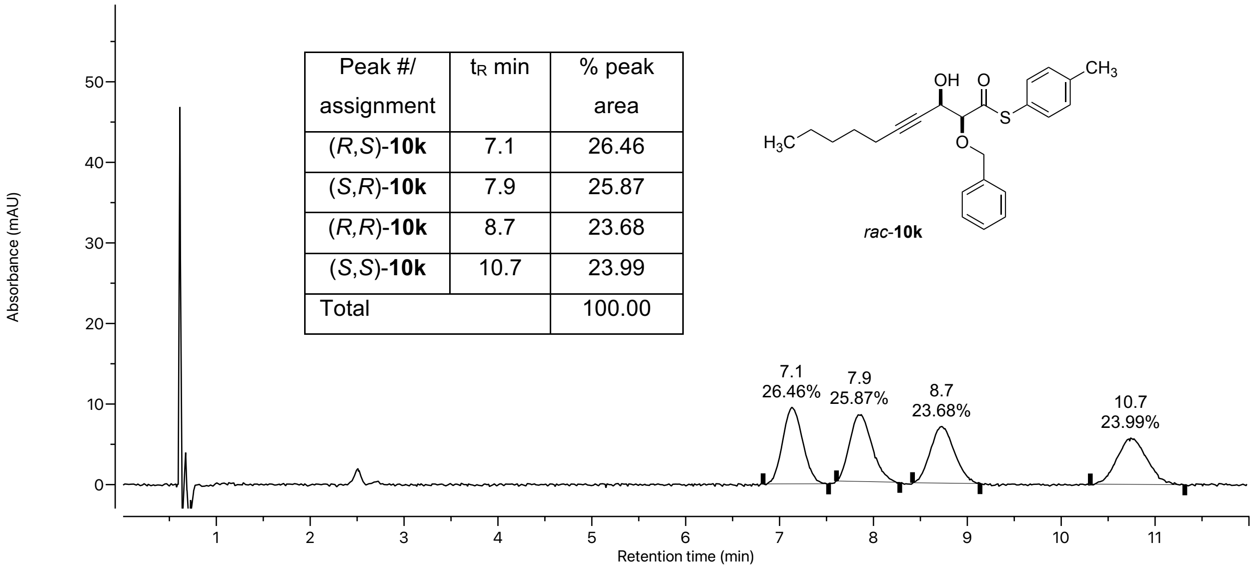

Peak #/ assignment	t_R min	% peak area
(*R*,*S*)-**10k**	7.1	26.46
(*S*,*R*)-**10k**	7.9	25.87
(*R*,*R*)-**10k**	8.7	23.68
(*S*,*S*)-**10k**	10.7	23.99
Total		100.00

syn-product:

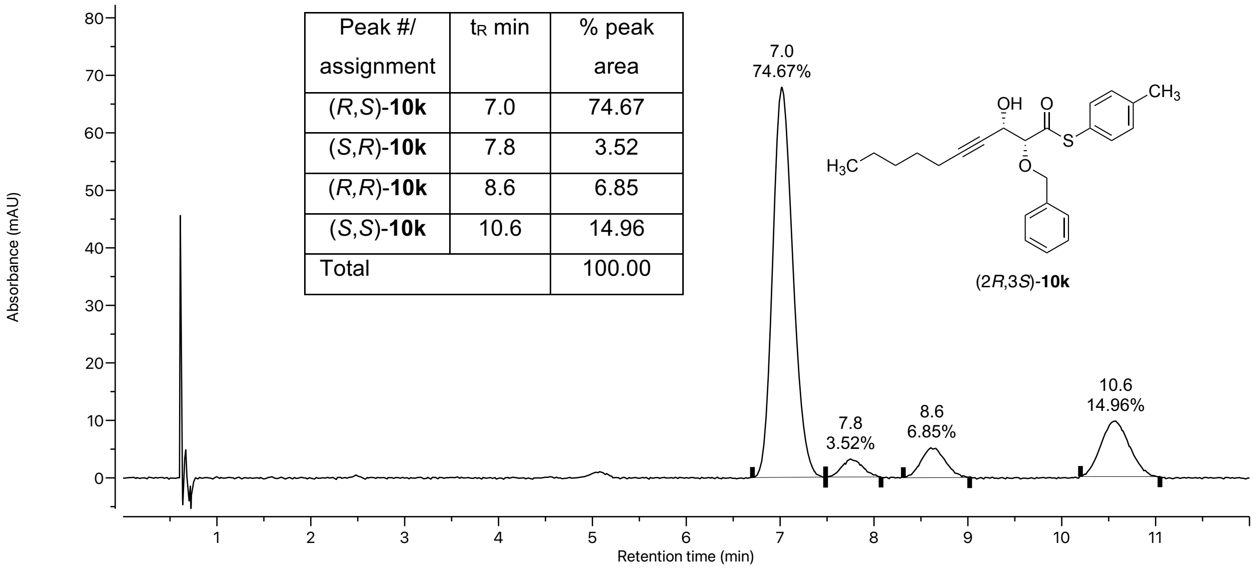

Peak #/ assignment	t_R min	% peak area
(*R*,*S*)-**10k**	7.0	74.67
(*S*,*R*)-**10k**	7.8	3.52
(*R*,*R*)-**10k**	8.6	6.85
(*S*,*S*)-**10k**	10.6	14.96
Total		100.00

SFC (IC-3, CO_2/CH_3OH =80:20, 2.0 mL/min, 298 K, 254 nm) for **10l**:

Racemic product:

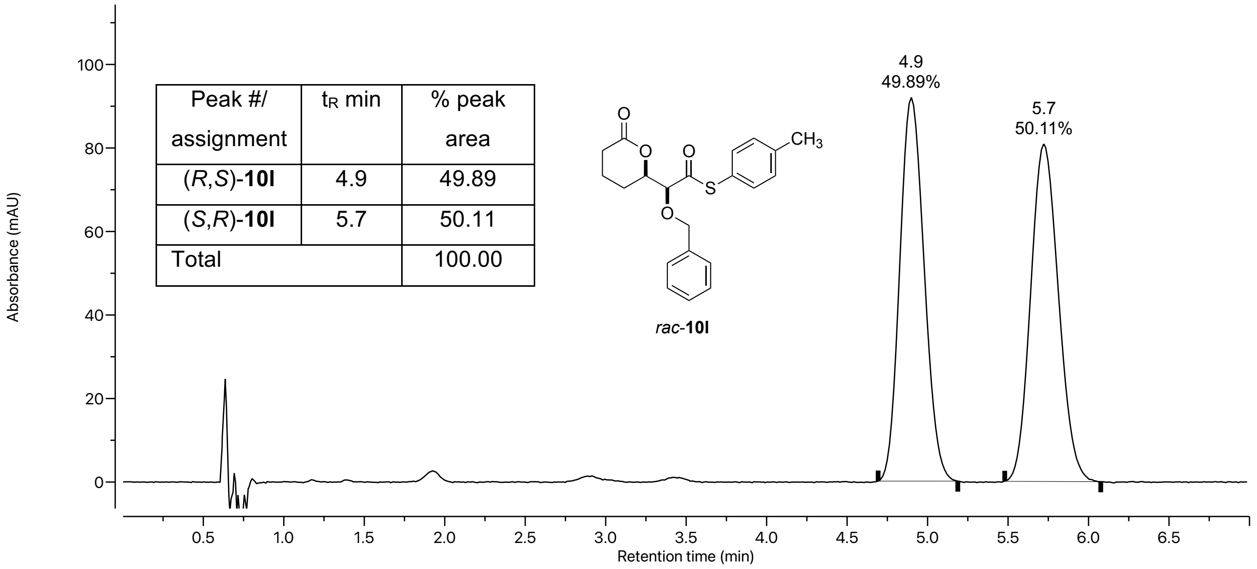

Peak #/ assignment	t_R min	% peak area
(*R*,*S*)-**10l**	4.9	49.89
(*S*,*R*)-**10l**	5.7	50.11
Total		100.00

syn-product:

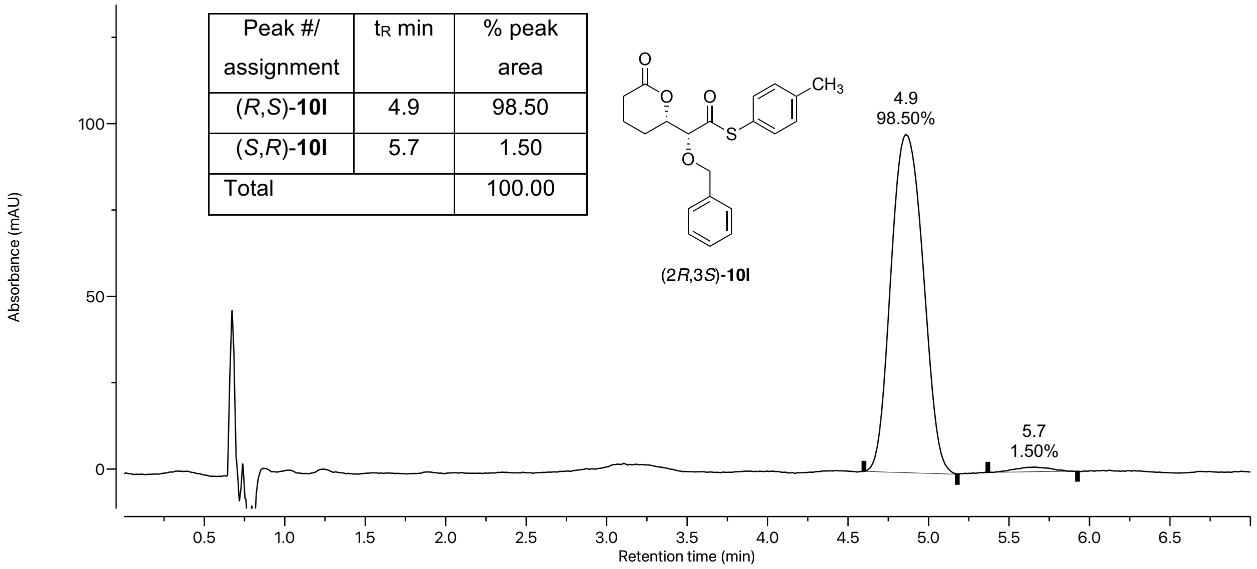

Peak #/ assignment	t_R min	% peak area
(*R*,*S*)-**10l**	4.9	98.50
(*S*,*R*)-**10l**	5.7	1.50
Total		100.00

SFC (IG-3, CO_2/CH_3OH =90:10, 2.0 mL/min, 298 K, 254 nm) for **10m**:

Racemic product:

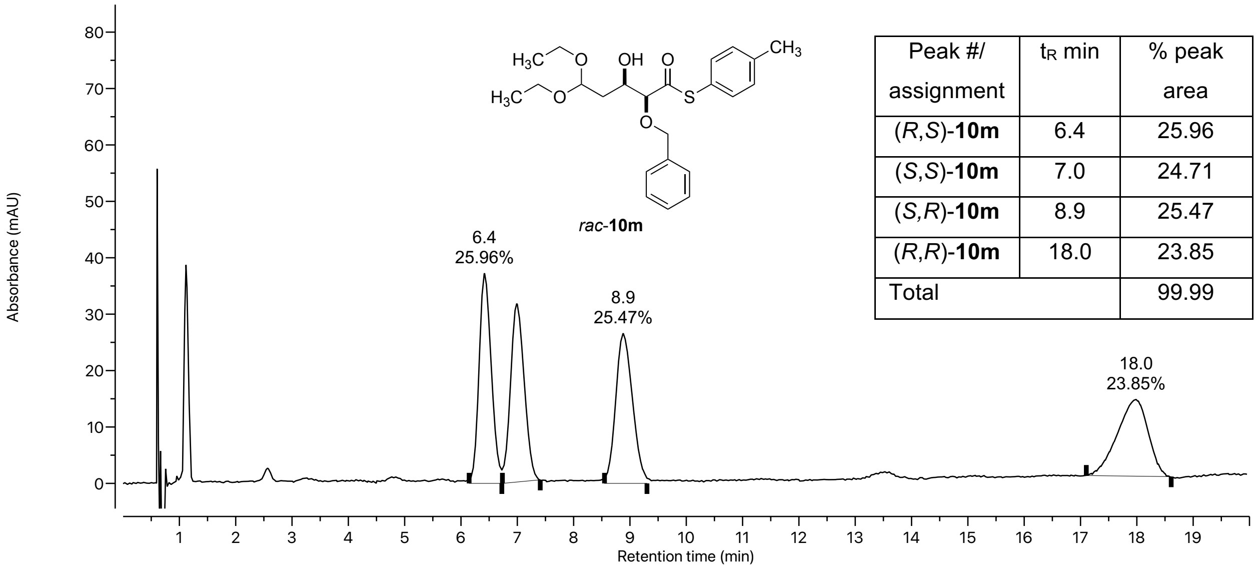

Peak #/ assignment	t_R min	% peak area
(*R*,*S*)-**10m**	6.4	25.96
(*S*,*S*)-**10m**	7.0	24.71
(*S*,*R*)-**10m**	8.9	25.47
(*R*,*R*)-**10m**	18.0	23.85
Total		99.99

syn-product:

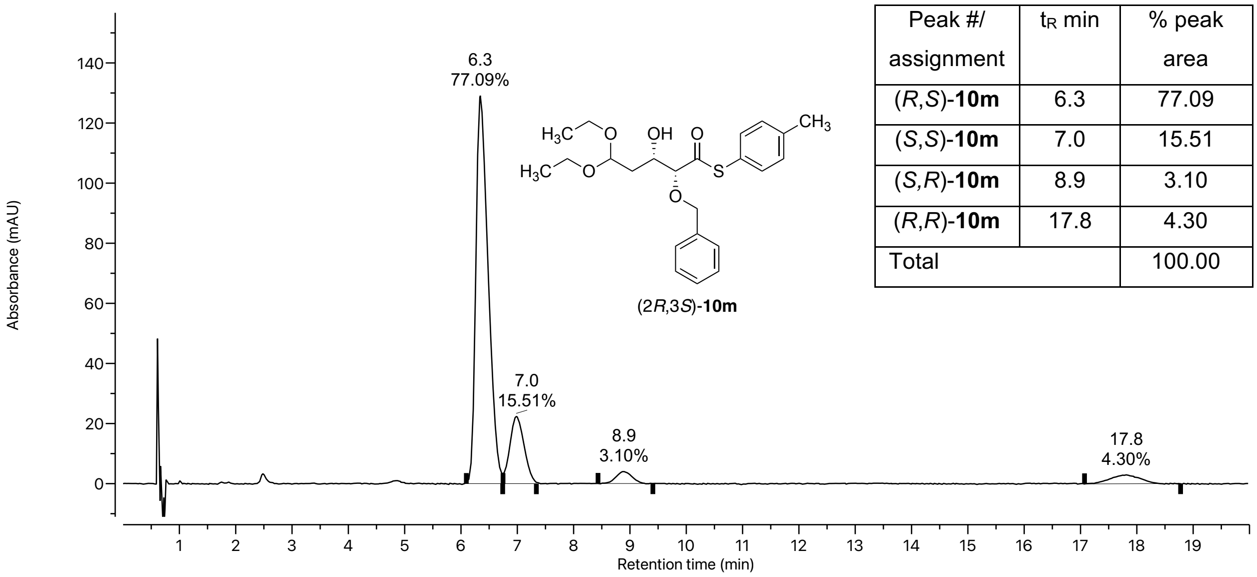

Peak #/ assignment	t_R min	% peak area
(*R*,*S*)-**10m**	6.3	77.09
(*S*,*S*)-**10m**	7.0	15.51
(*S*,*R*)-**10m**	8.9	3.10
(*R*,*R*)-**10m**	17.8	4.30
Total		100.00

SFC (OJ-H, CO_2/CH_3OH =80:20, 2.0 mL/min, 298 K, 254 nm) for **10n**:

Racemic product:

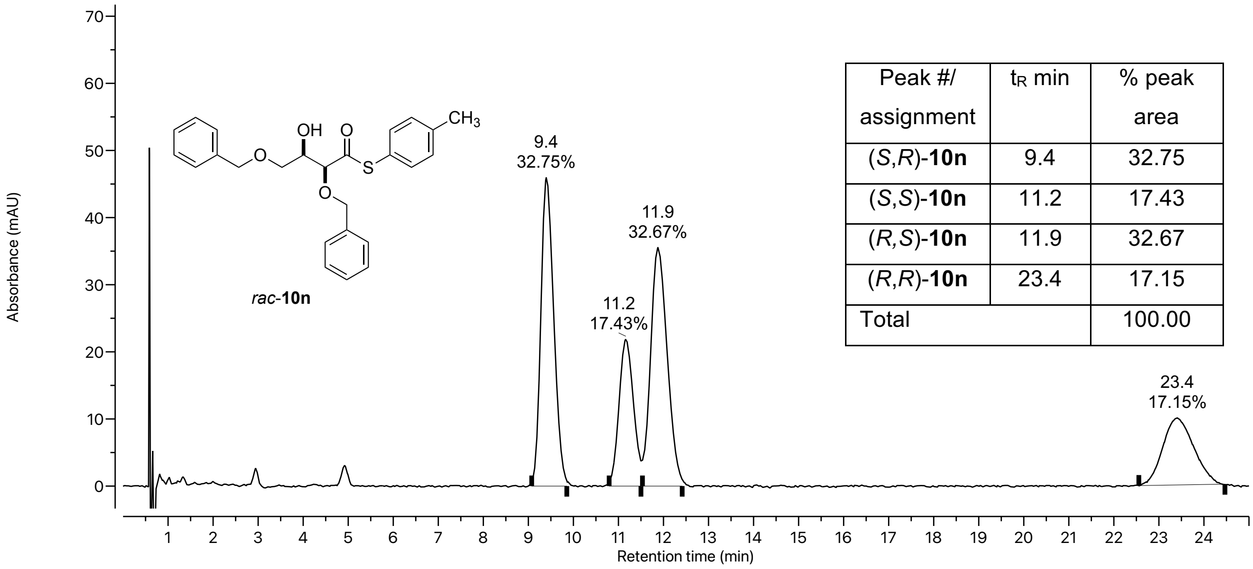

Peak #/ assignment	t_R min	% peak area
(*S*,*R*)-**10n**	9.4	32.75
(*S*,*S*)-**10n**	11.2	17.43
(*R*,*S*)-**10n**	11.9	32.67
(*R*,*R*)-**10n**	23.4	17.15
Total		100.00

syn-product:

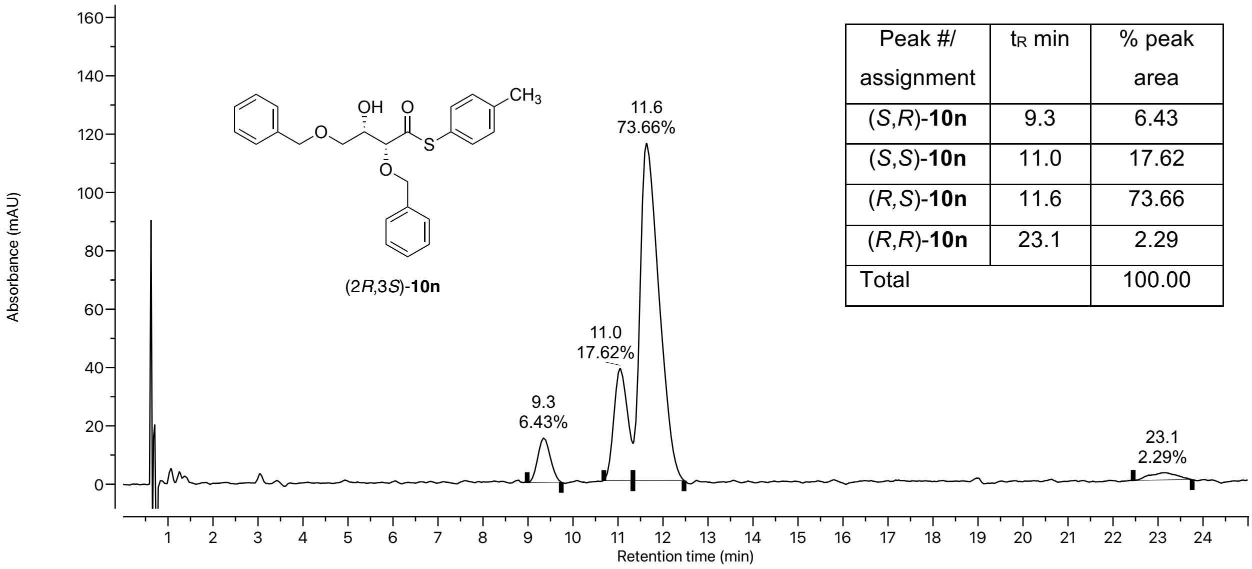

Peak #/ assignment	t_R min	% peak area
(*S*,*R*)-**10n**	9.3	6.43
(*S*,*S*)-**10n**	11.0	17.62
(*R*,*S*)-**10n**	11.6	73.66
(*R*,*R*)-**10n**	23.1	2.29
Total		100.00

SFC (IC-3, CO_2/CH_3OH =80:20, 2.0 mL/min, 298 K, 254 nm) for **10o**:

Racemic product:

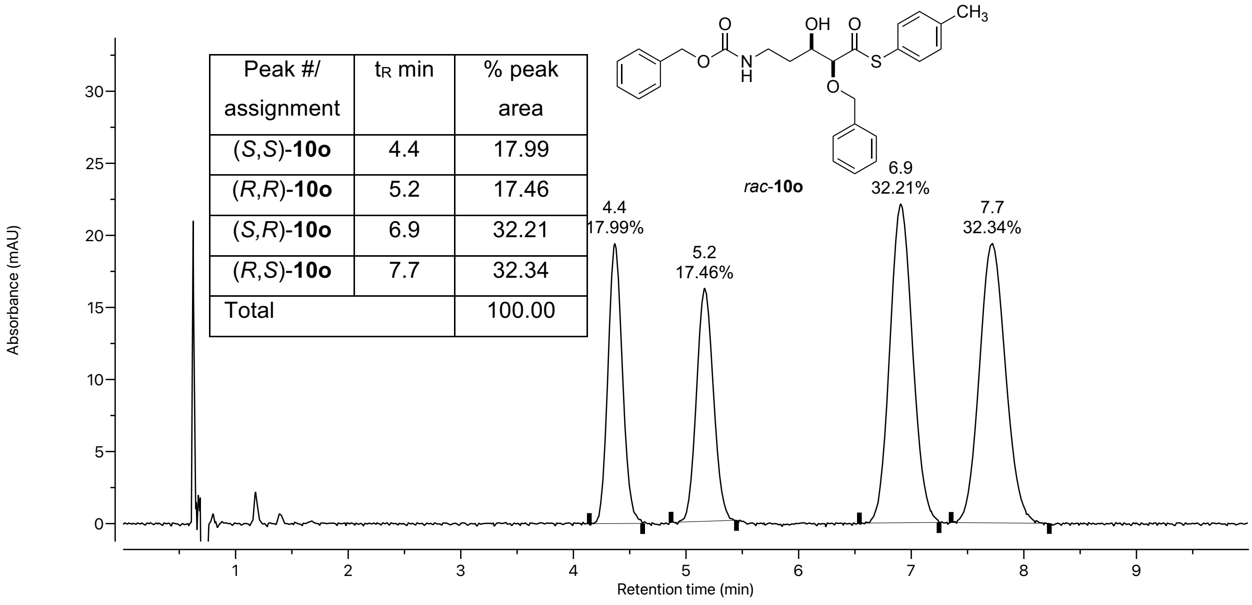

Peak #/ assignment	t_R min	% peak area
(*S*,*S*)-**10o**	4.4	17.99
(*R*,*R*)-**10o**	5.2	17.46
(*S*,*R*)-**10o**	6.9	32.21
(*R*,*S*)-**10o**	7.7	32.34
Total		100.00

syn-product:

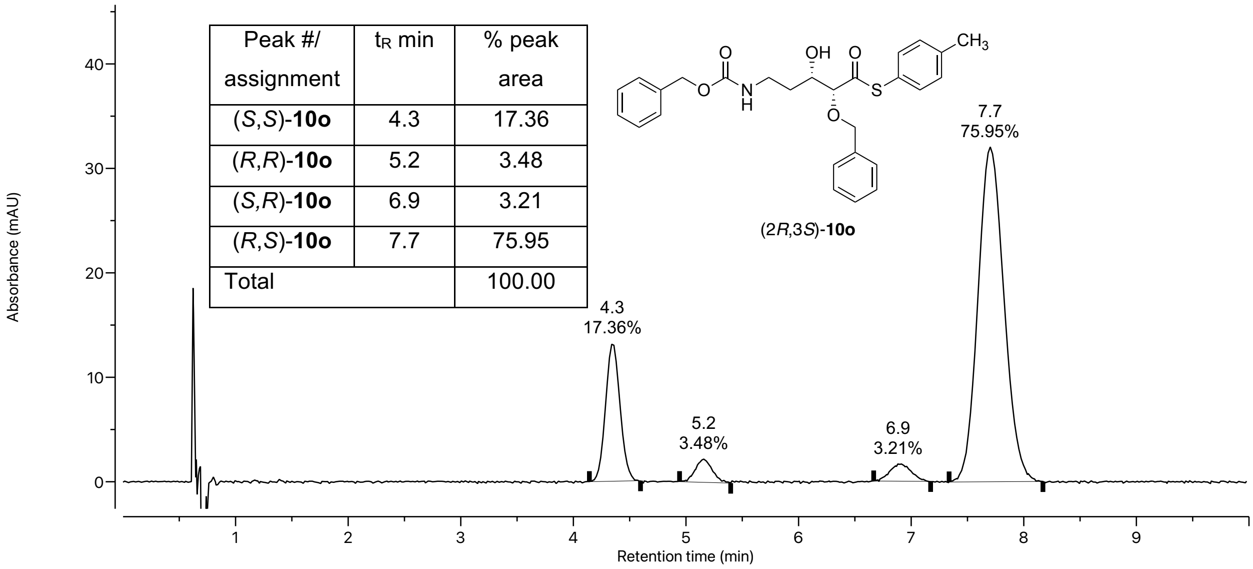

Peak #/ assignment	t_R min	% peak area
(*S*,*S*)-**10o**	4.3	17.36
(*R*,*R*)-**10o**	5.2	3.48
(*S*,*R*)-**10o**	6.9	3.21
(*R*,*S*)-**10o**	7.7	75.95
Total		100.00

SFC (IC-3, CO_2/CH_3OH =80:20, 2.0 mL/min, 298 K, 254 nm) for **10p**:

Racemic product:

Peak #/ assignment	t_R min	% peak area
(*S*,*S*)-**10p**	2.6	10.98
(*R*,*R*)-**10p**	3.1	11.13
(*S*,*R*)-**10p**	3.7	38.95
(*R*,*S*)-**10p**	4.1	38.94
Total		100.00

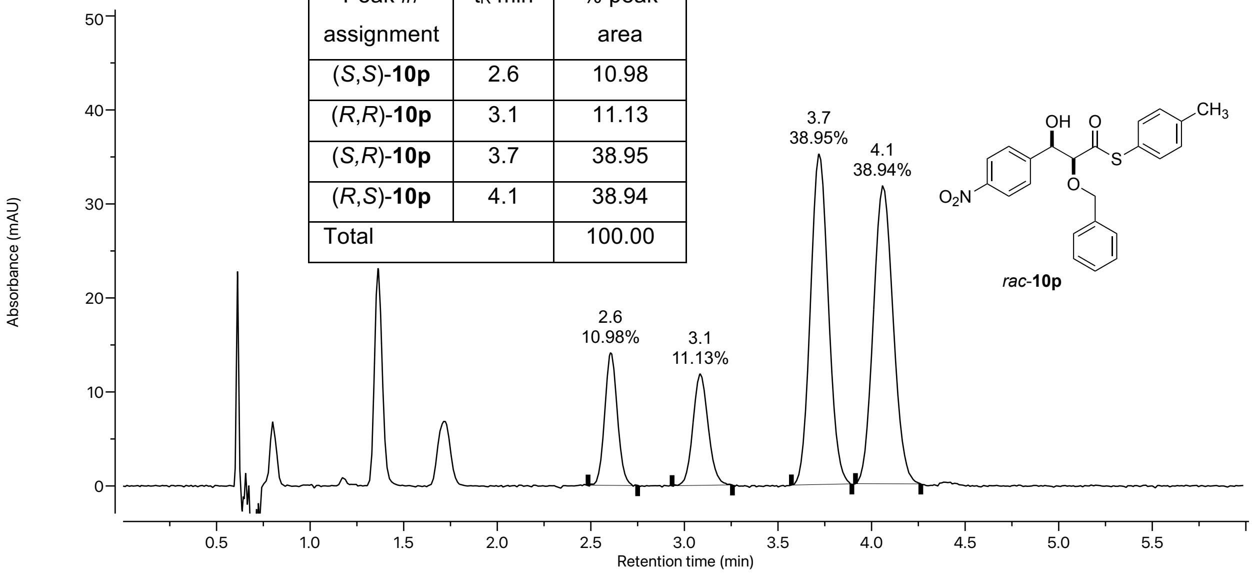

syn-product:

Peak #/ assignment	t_R min	% peak area
(*S*,*S*)-**10p**	2.6	2.81
(*R*,*R*)-**10p**	3.1	0.35
(*S*,*R*)-**10p**	3.7	96.39
(*R*,*S*)-**10p**	4.1	0.45
Total		100.00

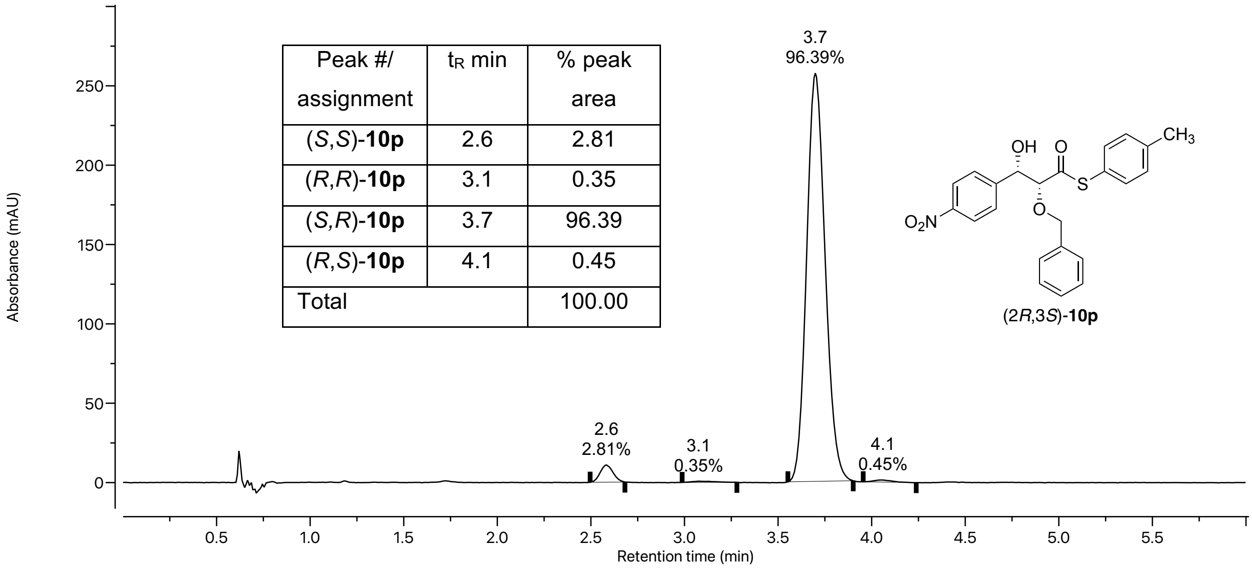

SFC (IC-3, CO_2/CH_3OH =94:6, 2.0 mL/min, 298 K, 254 nm) for **10q**:

Racemic product:

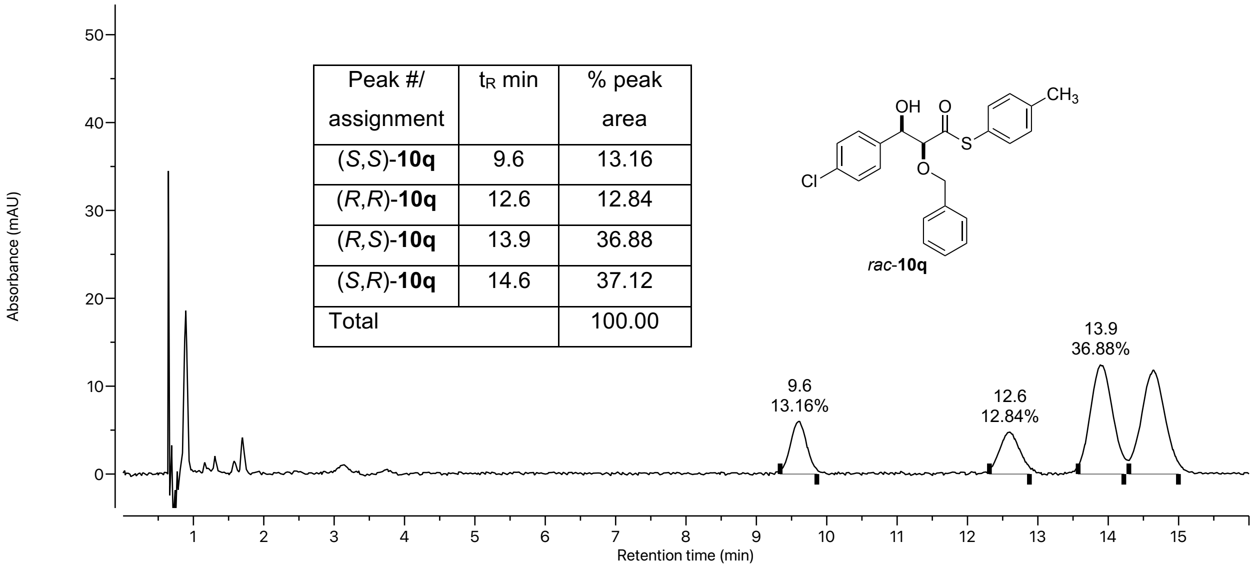

Peak #/ assignment	t_R min	% peak area
(*S*,*S*)-**10q**	9.6	13.16
(*R*,*R*)-**10q**	12.6	12.84
(*R*,*S*)-**10q**	13.9	36.88
(*S*,*R*)-**10q**	14.6	37.12
Total		100.00

syn-product:

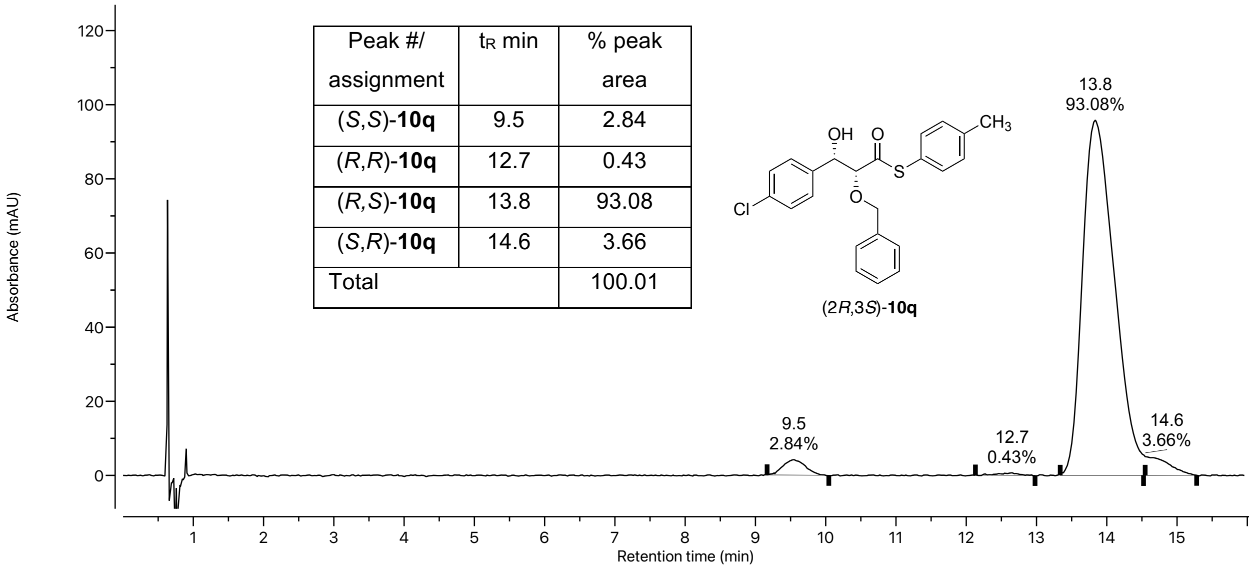

Peak #/ assignment	t_R min	% peak area
(*S*,*S*)-**10q**	9.5	2.84
(*R*,*R*)-**10q**	12.7	0.43
(*R*,*S*)-**10q**	13.8	93.08
(*S*,*R*)-**10q**	14.6	3.66
Total		100.01

SFC (IC-3, CO_2/CH_3OH =80:20, 2.0 mL/min, 298 K, 254 nm) for **10r**:

Racemic product:

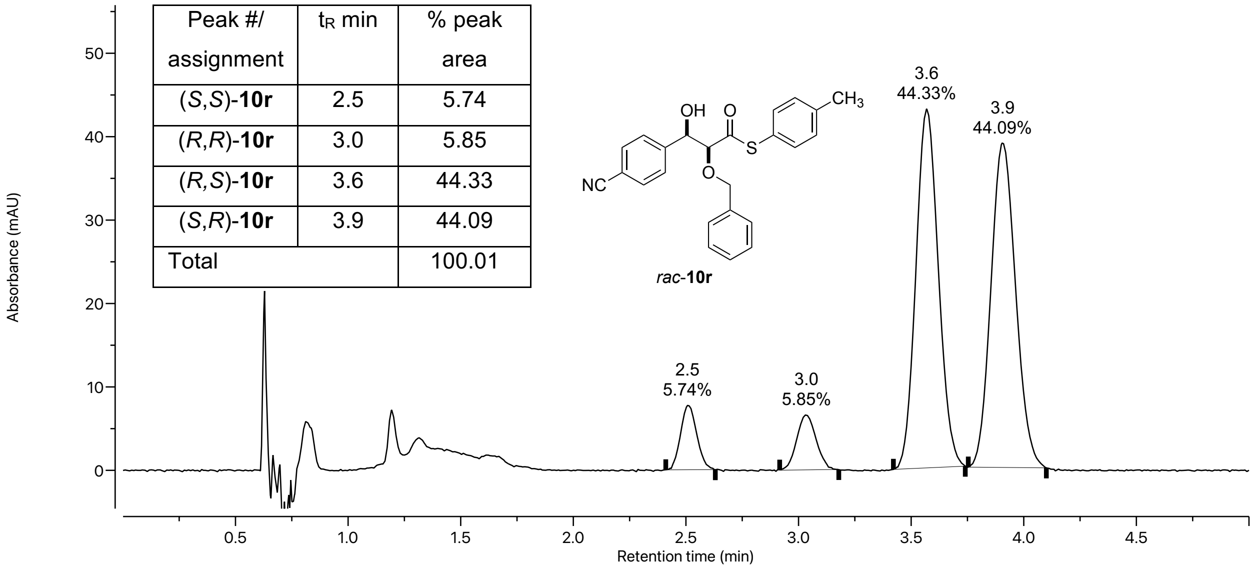

Peak #/ assignment	t_R min	% peak area
(*S*,*S*)-**10r**	2.5	5.74
(*R*,*R*)-**10r**	3.0	5.85
(*R*,*S*)-**10r**	3.6	44.33
(*S*,*R*)-**10r**	3.9	44.09
Total		100.01

syn-product:

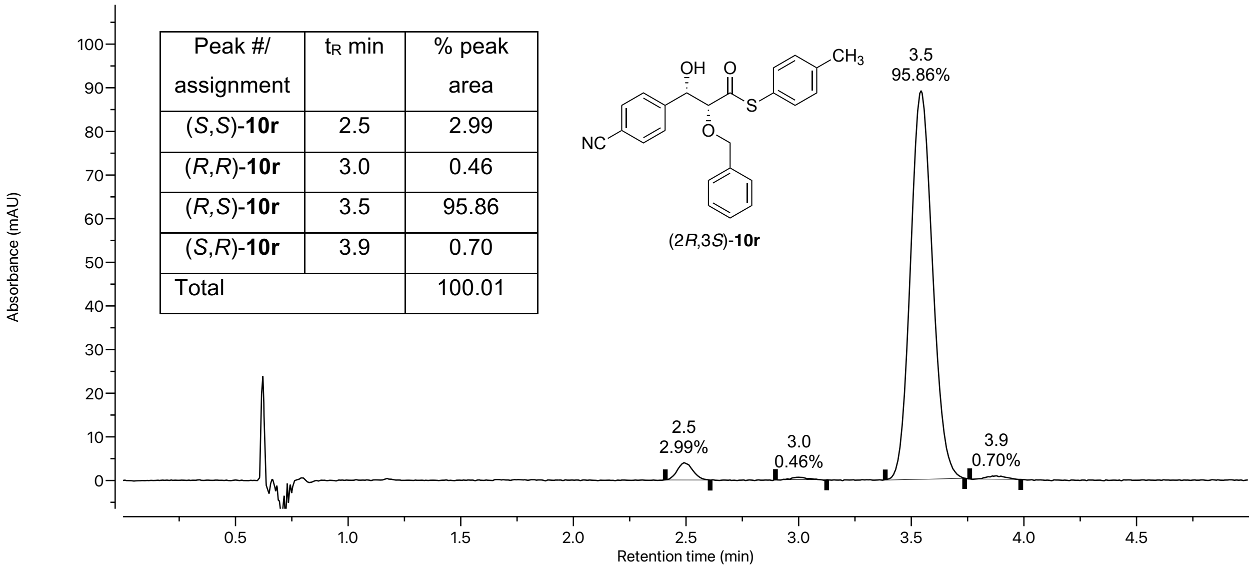

Peak #/ assignment	t_R min	% peak area
(*S*,*S*)-**10r**	2.5	2.99
(*R*,*R*)-**10r**	3.0	0.46
(*R*,*S*)-**10r**	3.5	95.86
(*S*,*R*)-**10r**	3.9	0.70
Total		100.01

SFC (IC-3, CO_2/CH_3OH =97:3, 2.0 mL/min, 298 K, 254 nm) for **10s**:

Racemic product:

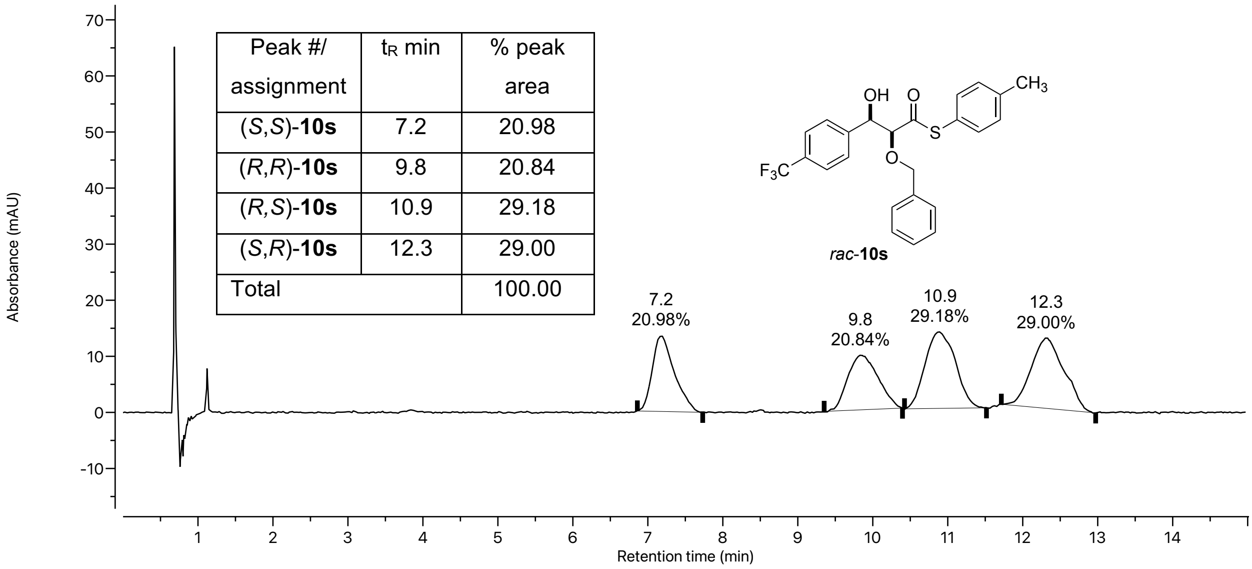

Peak #/ assignment	t_R min	% peak area
(*S*,*S*)-**10s**	7.2	20.98
(*R*,*R*)-**10s**	9.8	20.84
(*R*,*S*)-**10s**	10.9	29.18
(*S*,*R*)-**10s**	12.3	29.00
Total		100.00

syn-product:

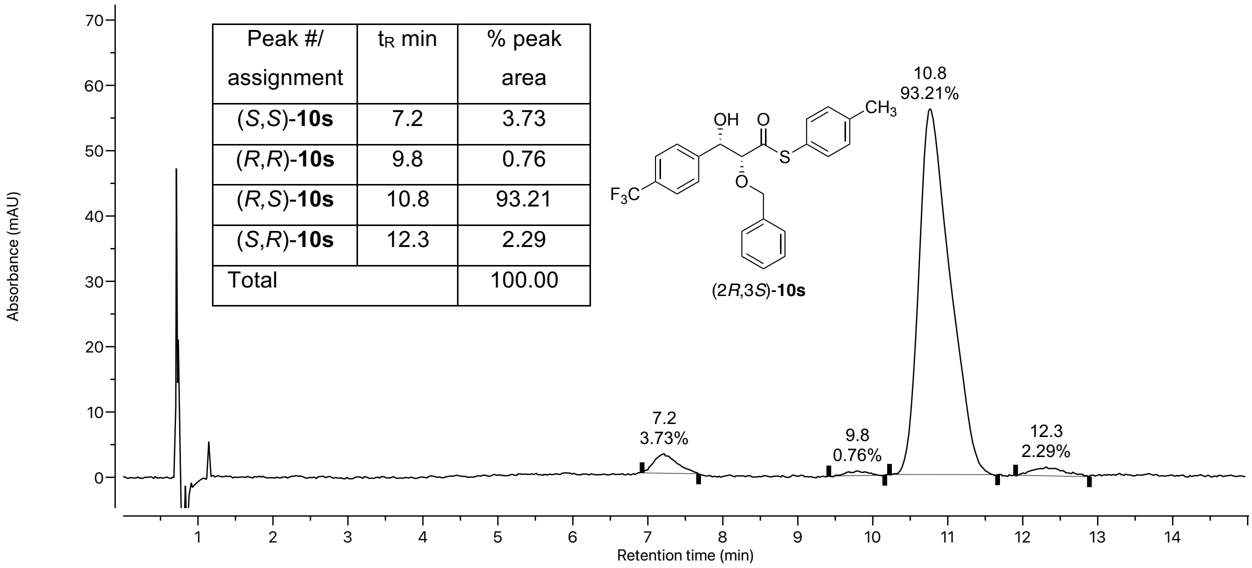

Peak #/ assignment	t_R min	% peak area
(*S*,*S*)-**10s**	7.2	3.73
(*R*,*R*)-**10s**	9.8	0.76
(*R*,*S*)-**10s**	10.8	93.21
(*S*,*R*)-**10s**	12.3	2.29
Total		100.00

SFC (IC-3, CO_2/CH_3OH =90:10, 2.0 mL/min, 298 K, 254 nm) for **10t**:

Racemic product:

Peak #/ assignment	t_R min	% peak area
(*S*,*S*)-**10t**	6.8	16.51
(*R*,*S*)-**10t**	8.4	33.57
(*R*,*R*)-**10t**	9.3	16.59
(*S*,*R*)-**10t**	10.4	33.33
Total		100.00

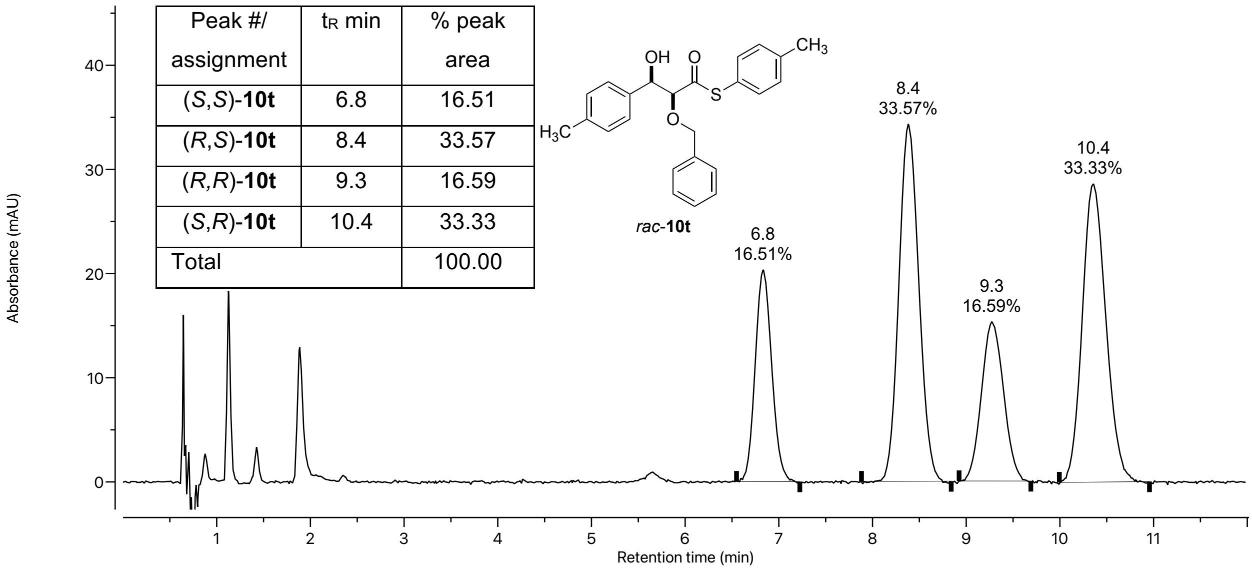

syn-product:

Peak #/ assignment	t_R min	% peak area
(*S*,*S*)-**10t**	7.0	6.46
(*R*,*S*)-**10t**	8.5	86.70
(*R*,*R*)-**10t**	9.4	2.01
(*S*,*R*)-**10t**	10.6	4.84
Total		100.01

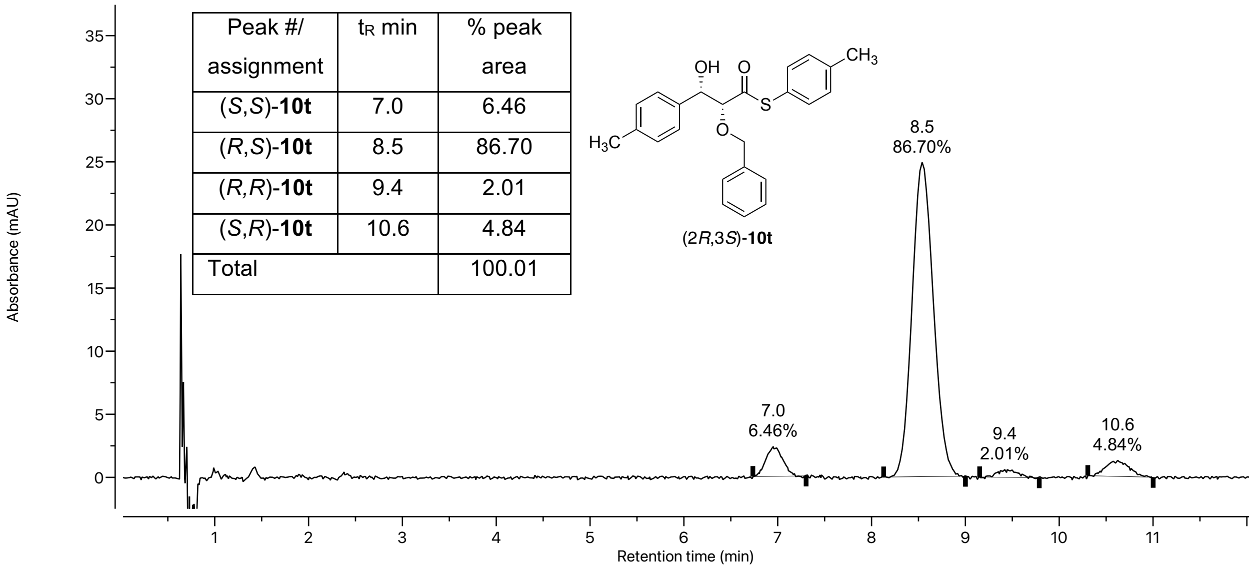

SFC (IA-3, CO_2/CH_3OH =85:15, 2.0 mL/min, 298 K, 254 nm) for **10u**:

Racemic product:

Peak #/ assignment	t_R min	% peak area
(*R*,*S*)-**10u**	11.0	32.72
(*S*,*S*)-**10u**	12.7	17.86
(*R*,*R*)-**10u**	13.9	17.37
(*S*,*R*)-**10u**	17.7	32.05
Total		100.00

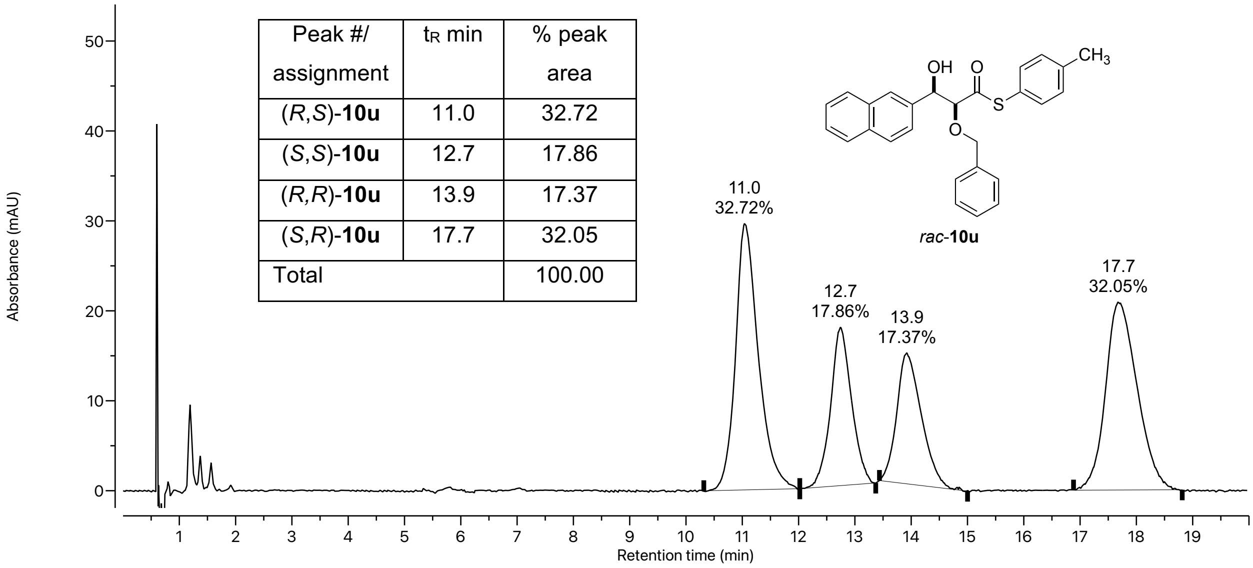

syn-product:

Peak #/ assignment	t_R min	% peak area
(*R*,*S*)-**10u**	10.9	82.95
(*S*,*S*)-**10u**	12.7	6.79
(*R*,*R*)-**10u**	14.0	2.20
(*S*,*R*)-**10u**	17.5	8.06
Total		100.00

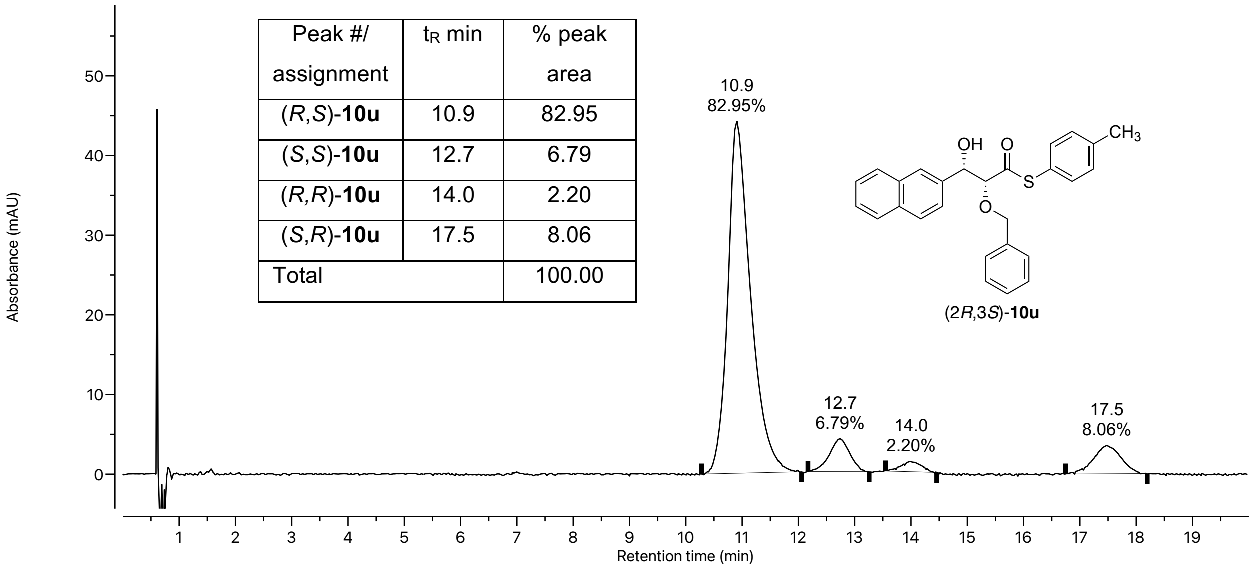

SFC (IA-3, CO_2/CH_3OH =80:20, 2.0 mL/min, 298 K, 254 nm) for **10v**:

Racemic product:

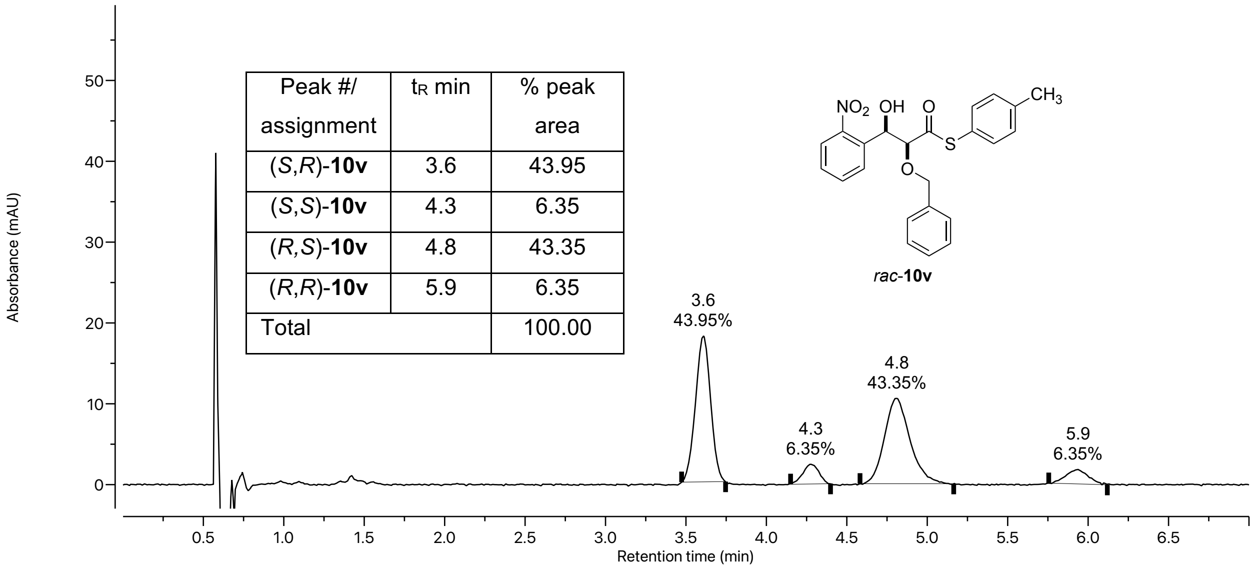

Peak #/ assignment	t_R min	% peak area
(*S*,*R*)-**10v**	3.6	43.95
(*S*,*S*)-**10v**	4.3	6.35
(*R*,*S*)-**10v**	4.8	43.35
(*R*,*R*)-**10v**	5.9	6.35
Total		100.00

syn-product:

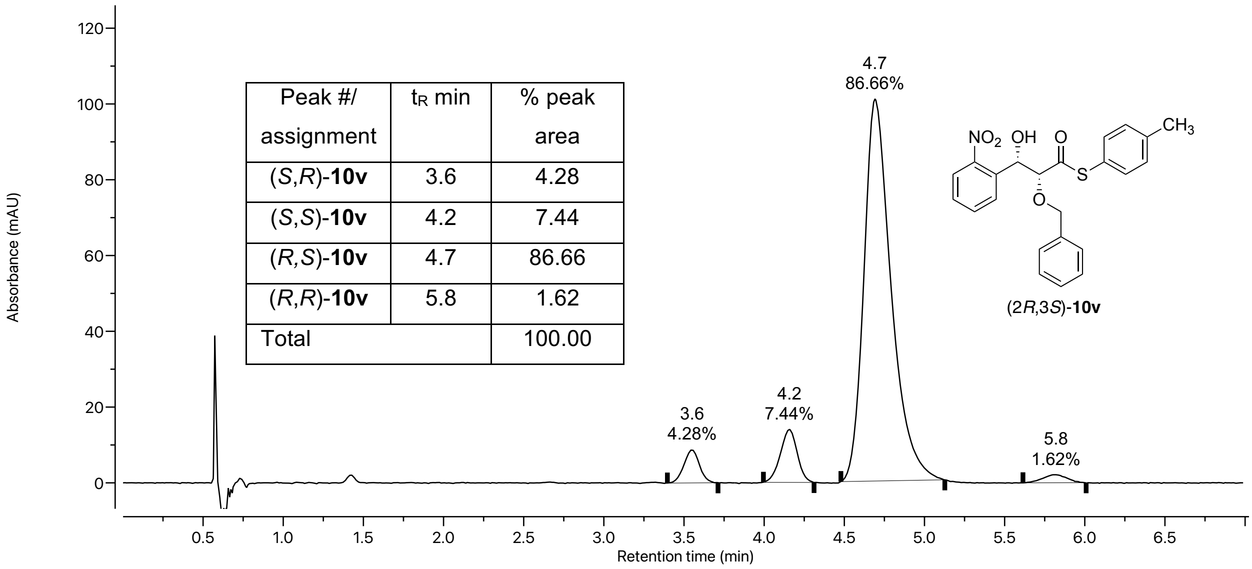

Peak #/ assignment	t_R min	% peak area
(*S*,*R*)-**10v**	3.6	4.28
(*S*,*S*)-**10v**	4.2	7.44
(*R*,*S*)-**10v**	4.7	86.66
(*R*,*R*)-**10v**	5.8	1.62
Total		100.00

SFC (OD-H, CO_2/CH_3OH =90:10, 2.0 mL/min, 298 K, 254 nm) for **10w**:

Racemic product:

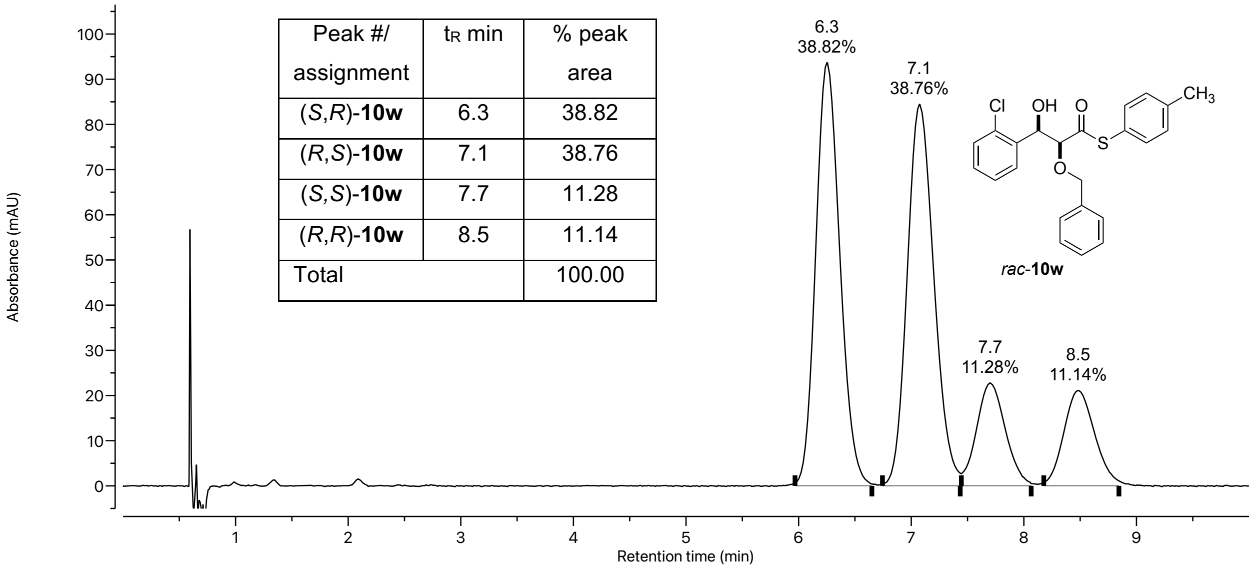

Peak #/ assignment	t_R min	% peak area
(*S*,*R*)-**10w**	6.3	38.82
(*R*,*S*)-**10w**	7.1	38.76
(*S*,*S*)-**10w**	7.7	11.28
(*R*,*R*)-**10w**	8.5	11.14
Total		100.00

syn-product:

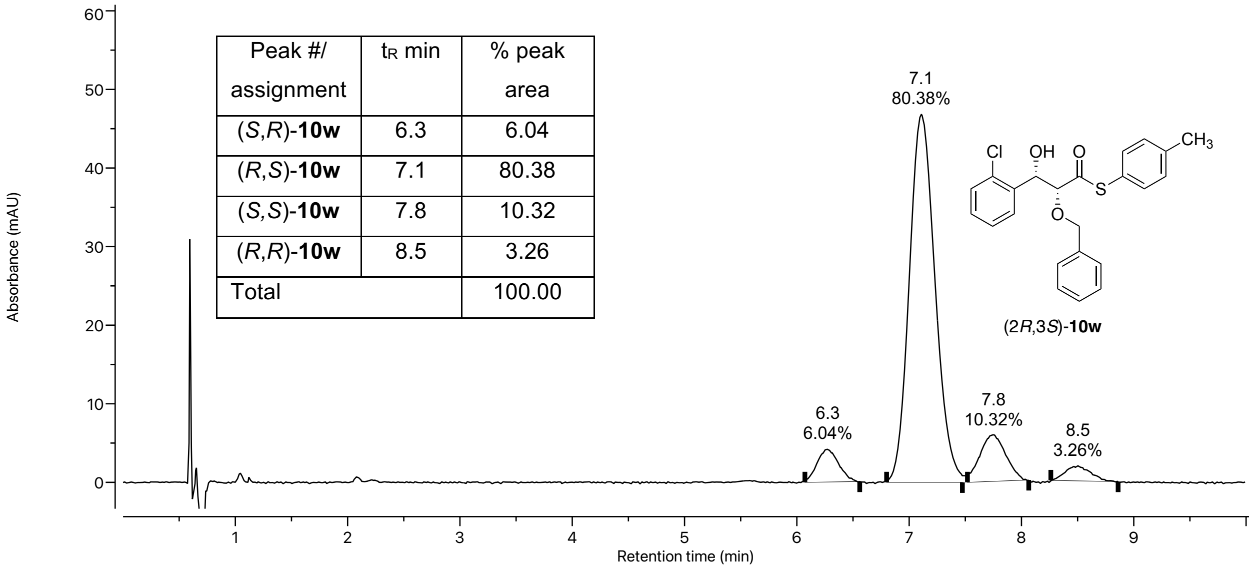

Peak #/ assignment	t_R min	% peak area
(*S*,*R*)-**10w**	6.3	6.04
(*R*,*S*)-**10w**	7.1	80.38
(*S*,*S*)-**10w**	7.8	10.32
(*R*,*R*)-**10w**	8.5	3.26
Total		100.00

SFC (IC-3, CO_2/CH_3OH =80:20, 2.0 mL/min, 298 K, 254 nm) for **10x**:

Racemic product:

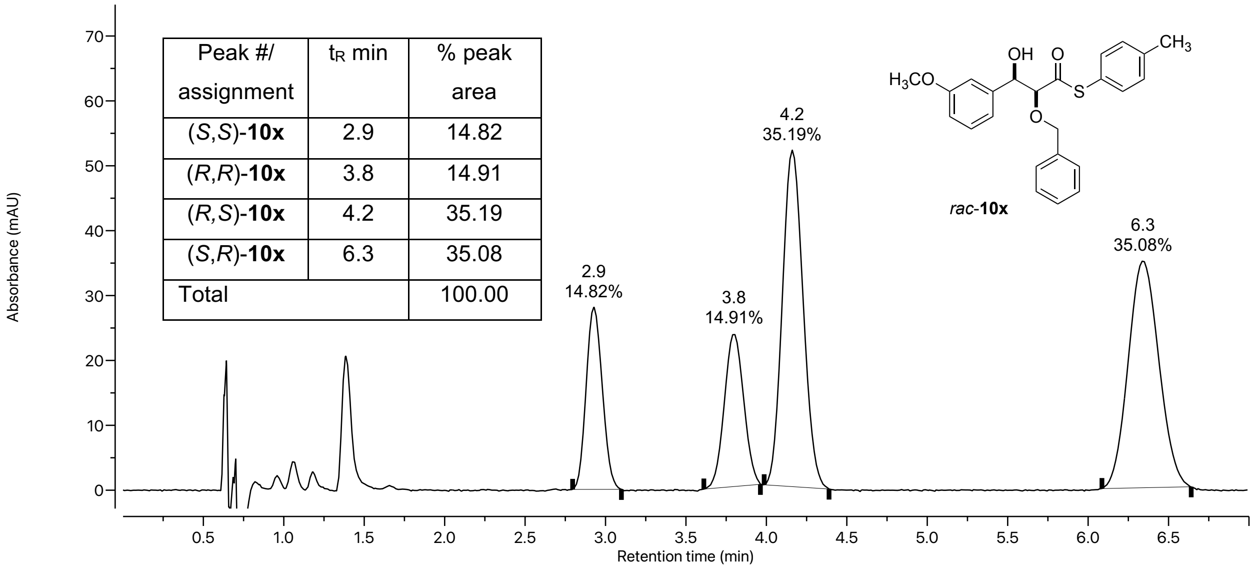

Peak #/ assignment	t_R min	% peak area
(*S*,*S*)-**10x**	2.9	14.82
(*R*,*R*)-**10x**	3.8	14.91
(*R*,*S*)-**10x**	4.2	35.19
(*S*,*R*)-**10x**	6.3	35.08
Total		100.00

syn-product:

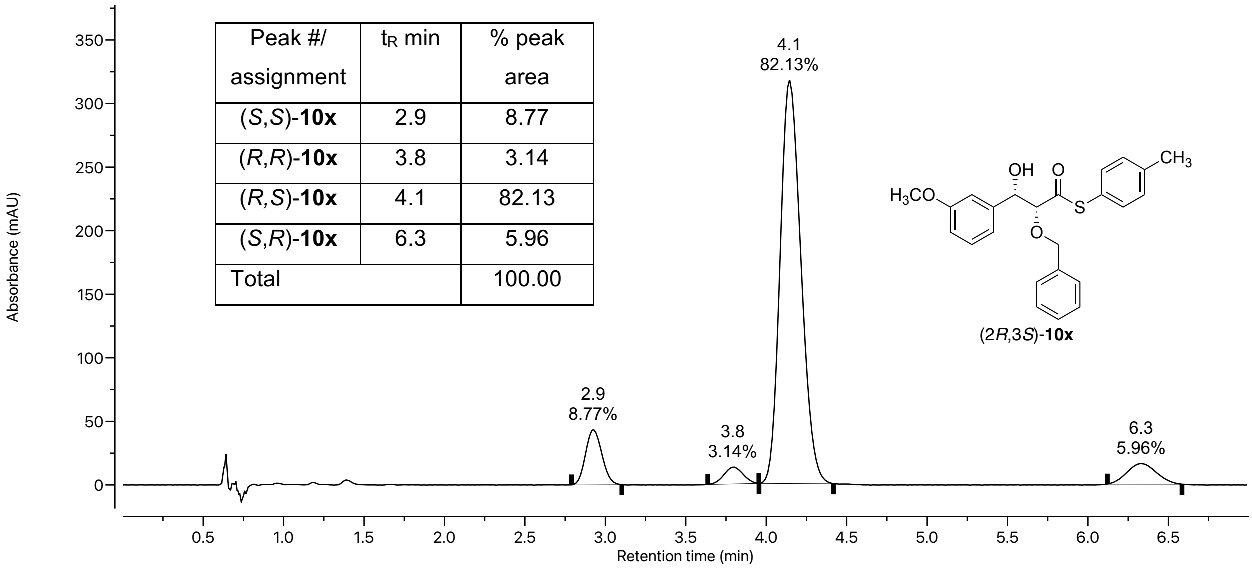

Peak #/ assignment	t_R min	% peak area
(*S*,*S*)-**10x**	2.9	8.77
(*R*,*R*)-**10x**	3.8	3.14
(*R*,*S*)-**10x**	4.1	82.13
(*S*,*R*)-**10x**	6.3	5.96
Total		100.00

SFC (IC-3, CO_2/CH_3OH =85:15, 2.0 mL/min, 298 K, 254 nm) for **10y**:

Racemic product:

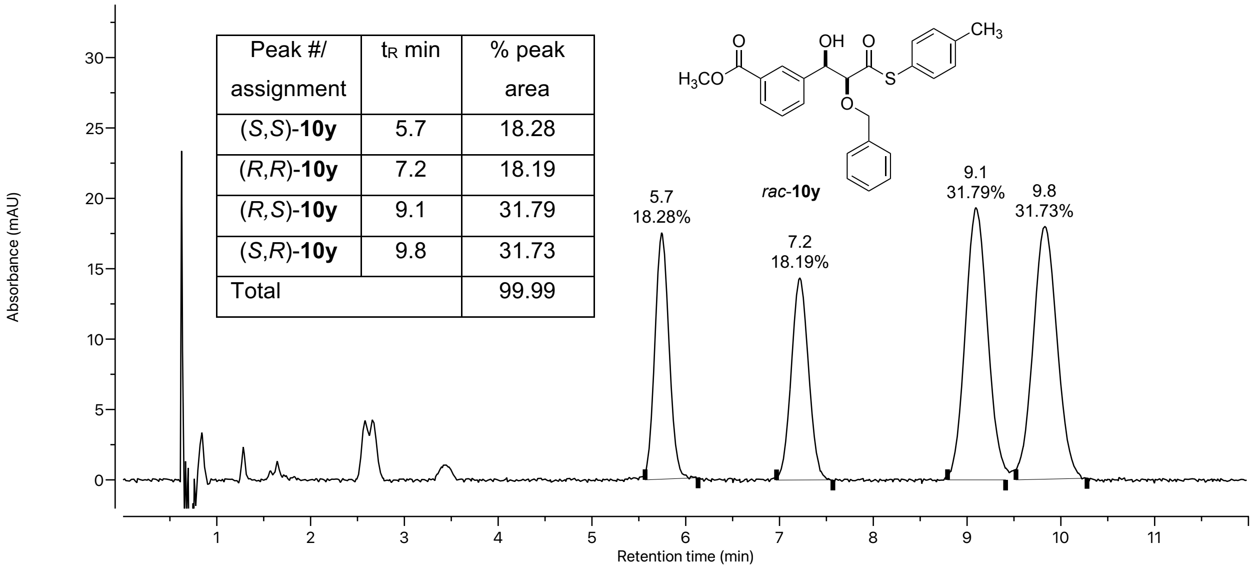

Peak #/ assignment	t_R min	% peak area
(*S*,*S*)-**10y**	5.7	18.28
(*R*,*R*)-**10y**	7.2	18.19
(*R*,*S*)-**10y**	9.1	31.79
(*S*,*R*)-**10y**	9.8	31.73
Total		99.99

syn-product:

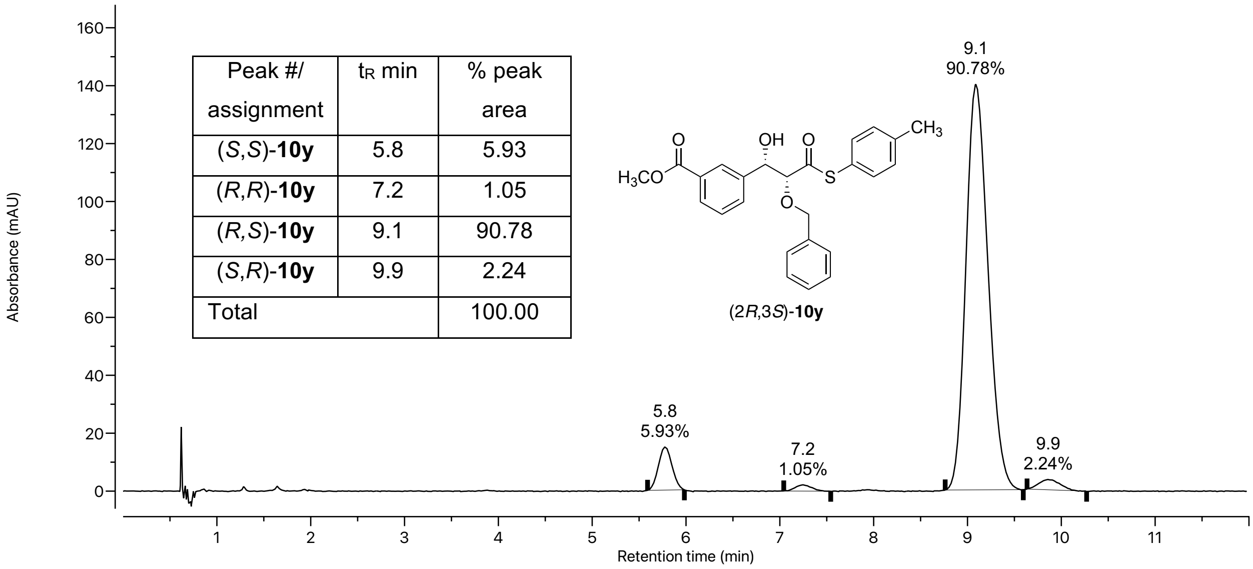

Peak #/ assignment	t_R min	% peak area
(*S*,*S*)-**10y**	5.8	5.93
(*R*,*R*)-**10y**	7.2	1.05
(*R*,*S*)-**10y**	9.1	90.78
(*S*,*R*)-**10y**	9.9	2.24
Total		100.00

SFC (IC-3, CO_2/CH_3OH =80:20, 2.0 mL/min, 298 K, 254 nm) for **10z**:

Racemic product:

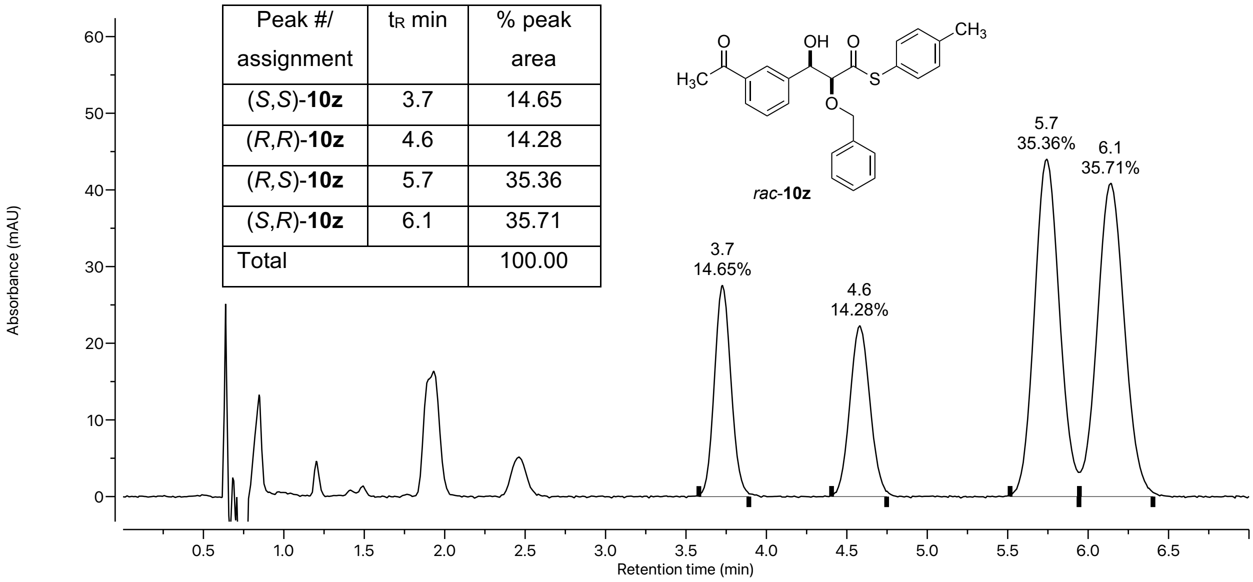

Peak #/ assignment	t_R min	% peak area
(*S*,*S*)-**10z**	3.7	14.65
(*R*,*R*)-**10z**	4.6	14.28
(*R*,*S*)-**10z**	5.7	35.36
(*S*,*R*)-**10z**	6.1	35.71
Total		100.00

syn-product:

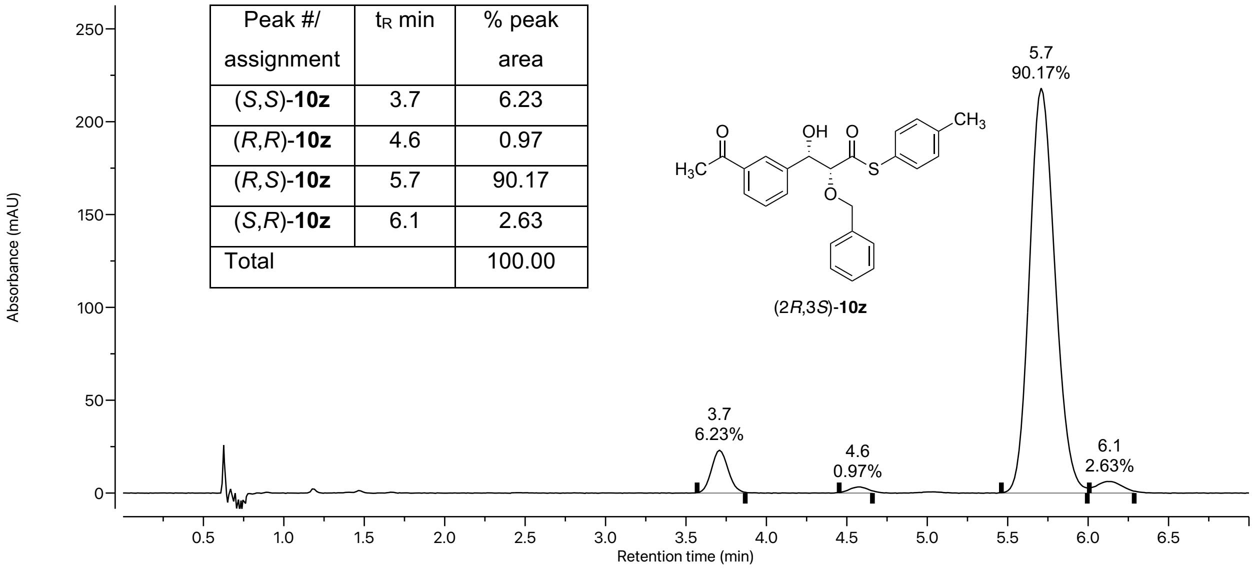

Peak #/ assignment	t_R min	% peak area
(*S*,*S*)-**10z**	3.7	6.23
(*R*,*R*)-**10z**	4.6	0.97
(*R*,*S*)-**10z**	5.7	90.17
(*S*,*R*)-**10z**	6.1	2.63
Total		100.00

SFC (IC-3, CO_2/CH_3OH =80:20, 2.0 mL/min, 298 K, 254 nm) for **10aa**:

Racemic product:

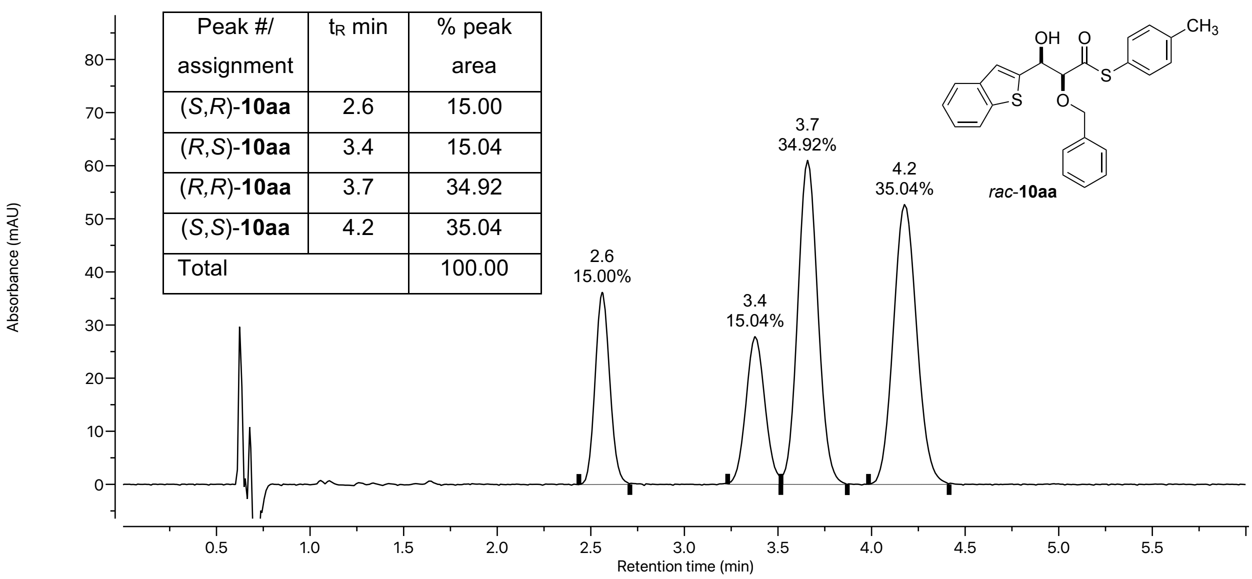

Peak #/ assignment	t_R min	% peak area
(*S*,*R*)-**10aa**	2.6	15.00
(*R*,*S*)-**10aa**	3.4	15.04
(*R*,*R*)-**10aa**	3.7	34.92
(*S*,*S*)-**10aa**	4.2	35.04
Total		100.00

syn-product:

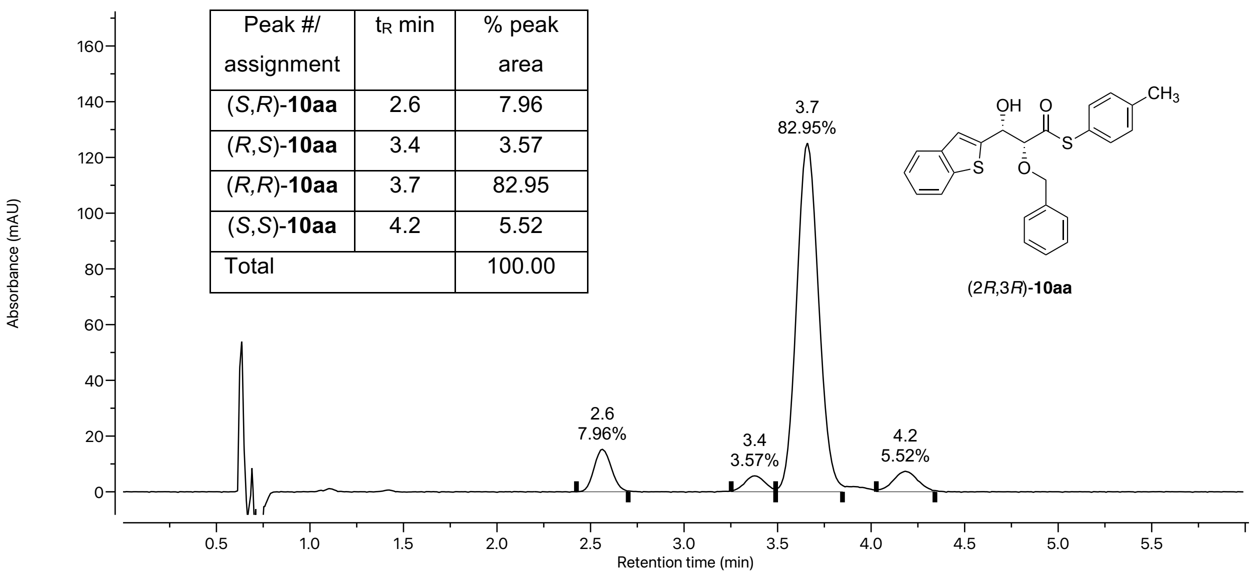

Peak #/ assignment	t_R min	% peak area
(*S*,*R*)-**10aa**	2.6	7.96
(*R*,*S*)-**10aa**	3.4	3.57
(*R*,*R*)-**10aa**	3.7	82.95
(*S*,*S*)-**10aa**	4.2	5.52
Total		100.00

SFC (IC-3, CO_2/CH_3OH =80:20, 2.0 mL/min, 298 K, 254 nm) for **10ab**:

Racemic product:

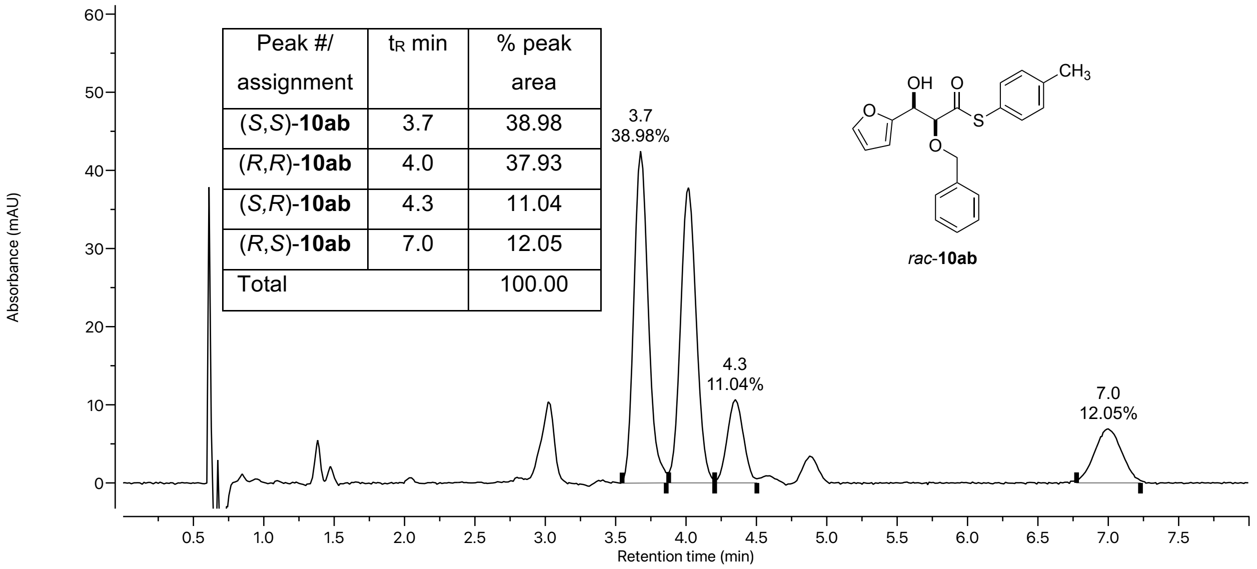

Peak #/ assignment	t_R min	% peak area
(*S*,*S*)-**10ab**	3.7	38.98
(*R*,*R*)-**10ab**	4.0	37.93
(*S*,*R*)-**10ab**	4.3	11.04
(*R*,*S*)-**10ab**	7.0	12.05
Total		100.00

syn-product:

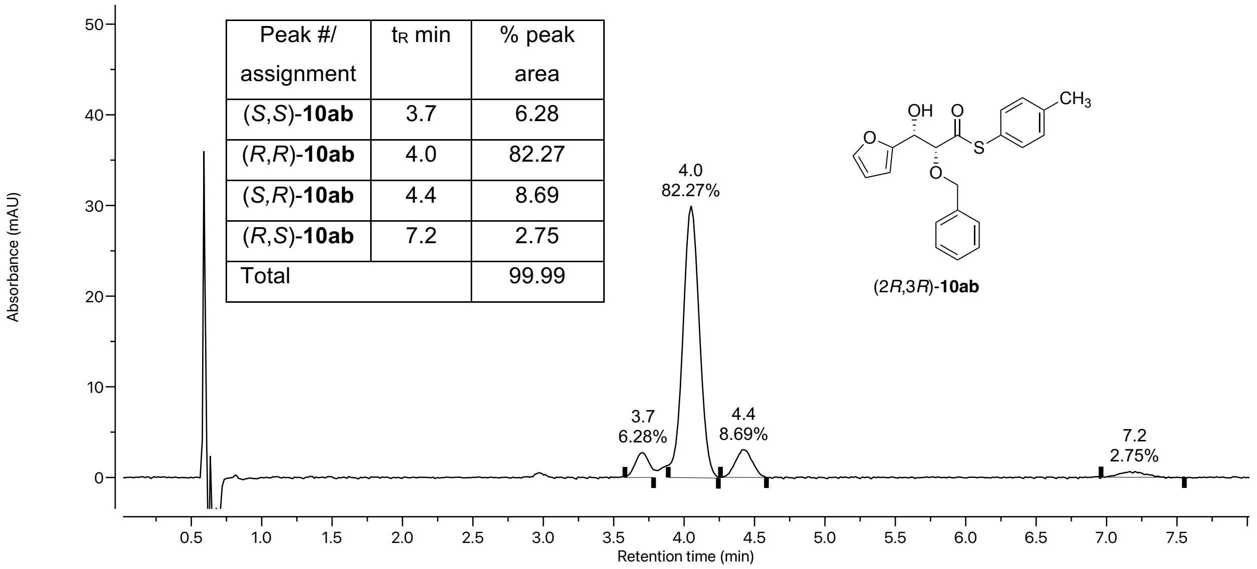

Peak #/ assignment	t_R min	% peak area
(*S*,*S*)-**10ab**	3.7	6.28
(*R*,*R*)-**10ab**	4.0	82.27
(*S*,*R*)-**10ab**	4.4	8.69
(*R*,*S*)-**10ab**	7.2	2.75
Total		99.99

SFC (OD-H, CO_2/CH_3OH =80:20, 2.0 mL/min, 298 K, 254 nm) for **10ac**:

Racemic product:

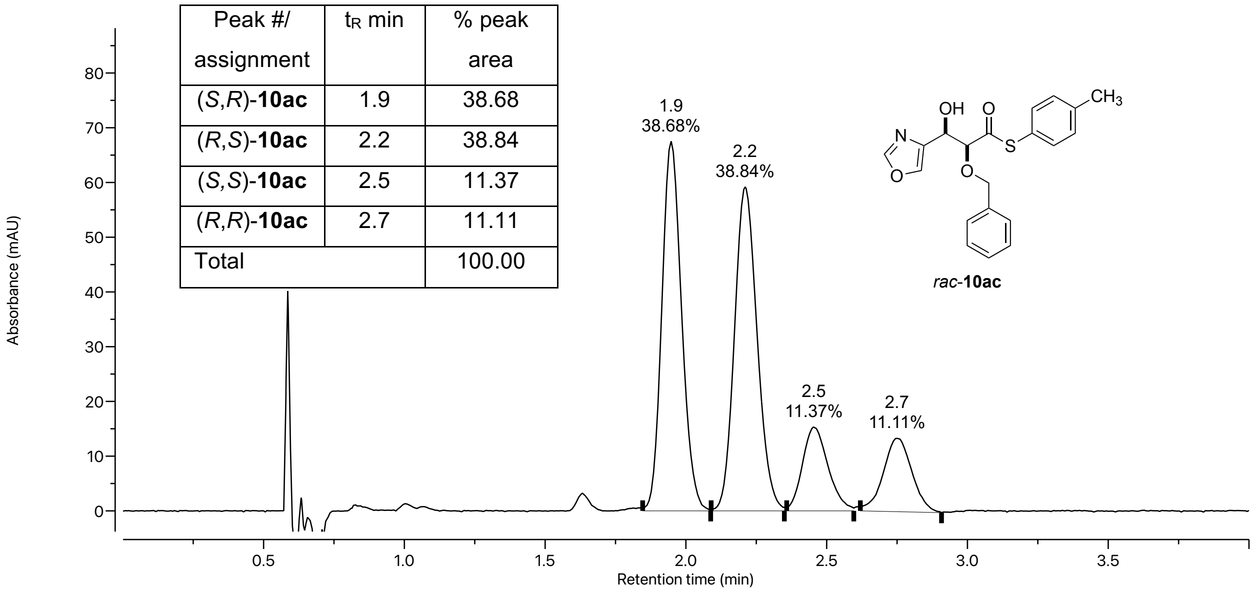

Peak #/ assignment	t_R min	% peak area
(*S*,*R*)-**10ac**	1.9	38.68
(*R*,*S*)-**10ac**	2.2	38.84
(*S*,*S*)-**10ac**	2.5	11.37
(*R*,*R*)-**10ac**	2.7	11.11
Total		100.00

syn-product:

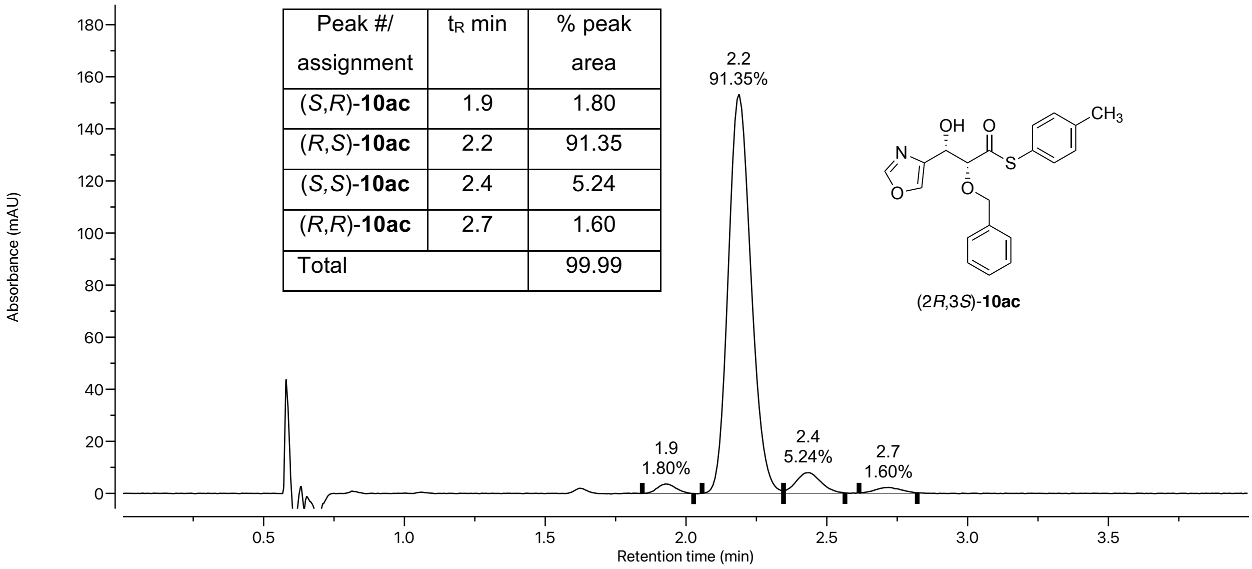

Peak #/ assignment	t_R min	% peak area
(*S*,*R*)-**10ac**	1.9	1.80
(*R*,*S*)-**10ac**	2.2	91.35
(*S*,*S*)-**10ac**	2.4	5.24
(*R*,*R*)-**10ac**	2.7	1.60
Total		99.99

SFC (OJ-H, CO_2/CH_3OH =80:20, 2.0 mL/min, 298 K, 254 nm) for **10ad**:

Racemic product:

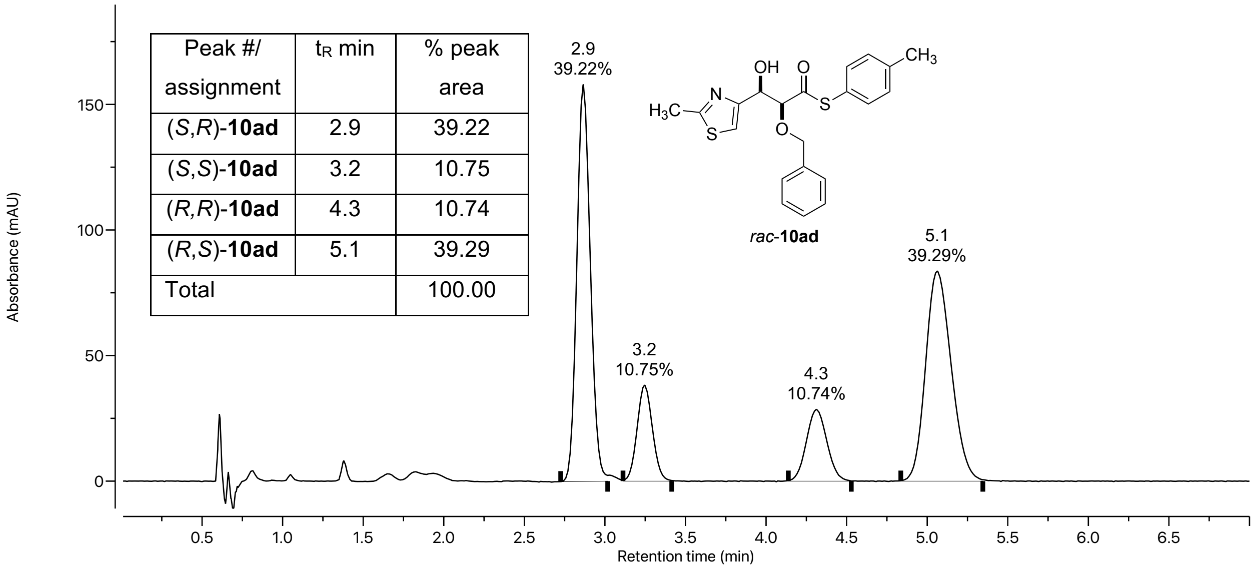

Peak #/ assignment	t_R min	% peak area
(*S*,*R*)-**10ad**	2.9	39.22
(*S*,*S*)-**10ad**	3.2	10.75
(*R*,*R*)-**10ad**	4.3	10.74
(*R*,*S*)-**10ad**	5.1	39.29
Total		100.00

syn-product:

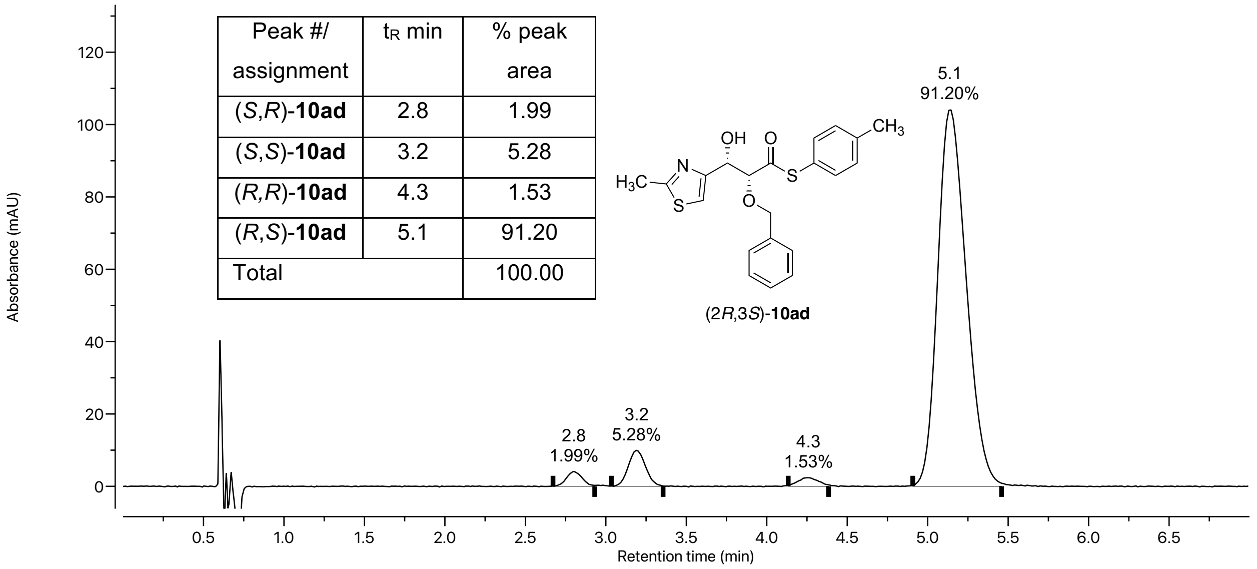

Peak #/ assignment	t_R min	% peak area
(*S*,*R*)-**10ad**	2.8	1.99
(*S*,*S*)-**10ad**	3.2	5.28
(*R*,*R*)-**10ad**	4.3	1.53
(*R*,*S*)-**10ad**	5.1	91.20
Total		100.00

SFC (IC-3, CO_2/CH_3OH =90:10, 2.0 mL/min, 298 K, 254 nm) for **10ae**:

Racemic product:

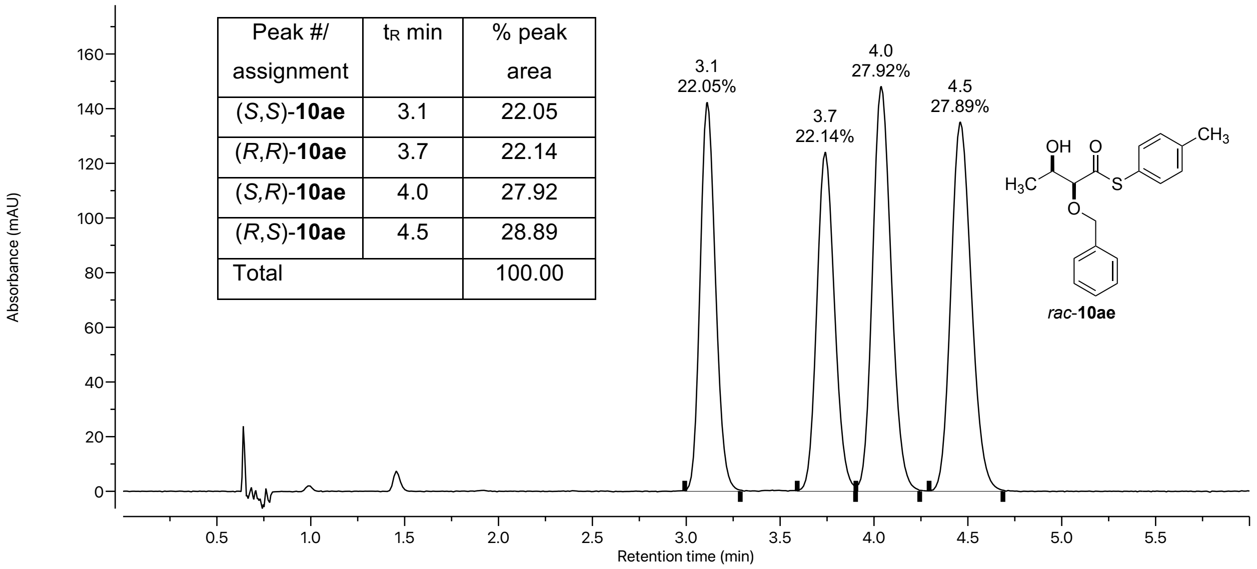

Peak #/ assignment	t_R min	% peak area
(*S*,*S*)-**10ae**	3.1	22.05
(*R*,*R*)-**10ae**	3.7	22.14
(*S*,*R*)-**10ae**	4.0	27.92
(*R*,*S*)-**10ae**	4.5	28.89
Total		100.00

(S,R)-syn-product:

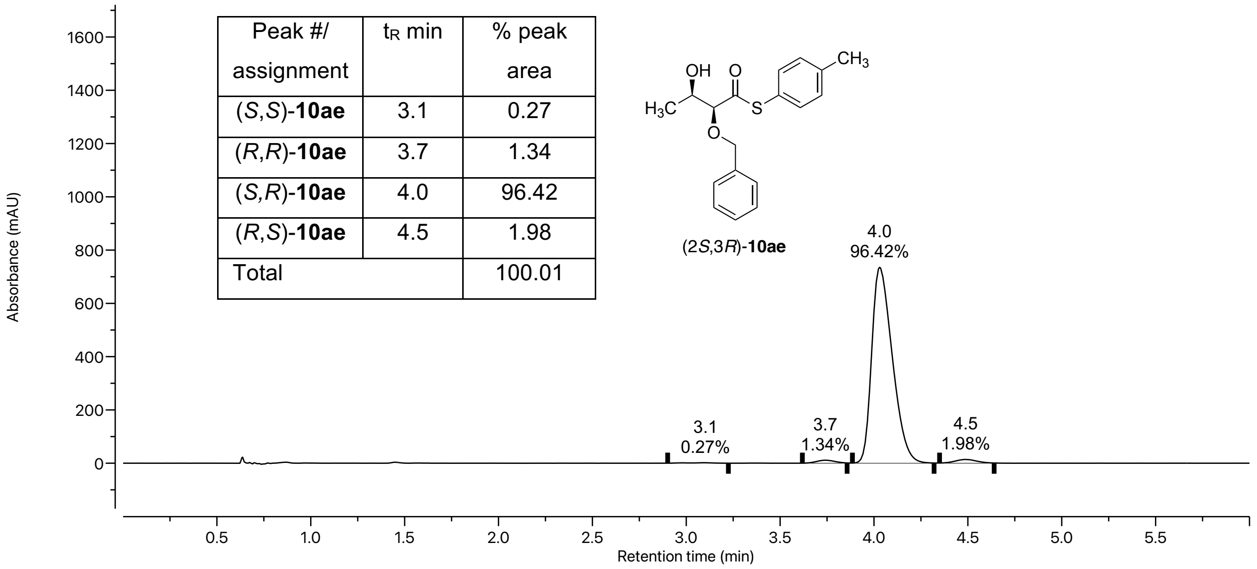

Peak #/ assignment	t_R min	% peak area
(*S*,*S*)-**10ae**	3.1	0.27
(*R*,*R*)-**10ae**	3.7	1.34
(*S*,*R*)-**10ae**	4.0	96.42
(*R*,*S*)-**10ae**	4.5	1.98
Total		100.01

(R,S)-syn-product:

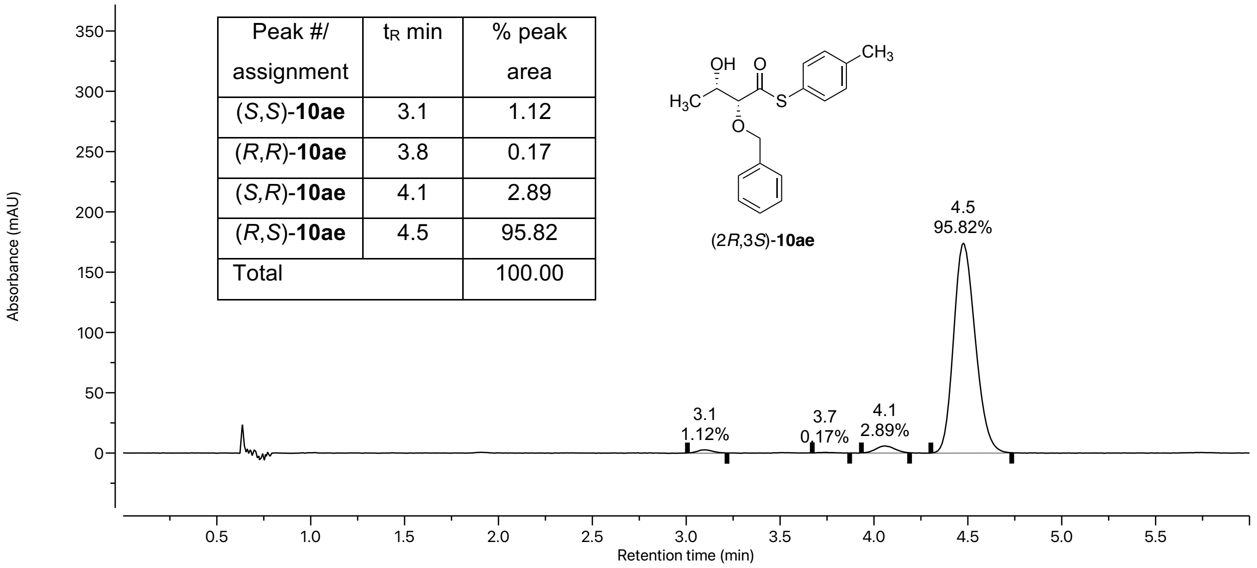

Peak #/ assignment	t_R min	% peak area
(*S*,*S*)-**10ae**	3.1	1.12
(*R*,*R*)-**10ae**	3.8	0.17
(*S*,*R*)-**10ae**	4.1	2.89
(*R*,*S*)-**10ae**	4.5	95.82
Total		100.00

Catalog of Nuclear Magnetic Resonance Spectra

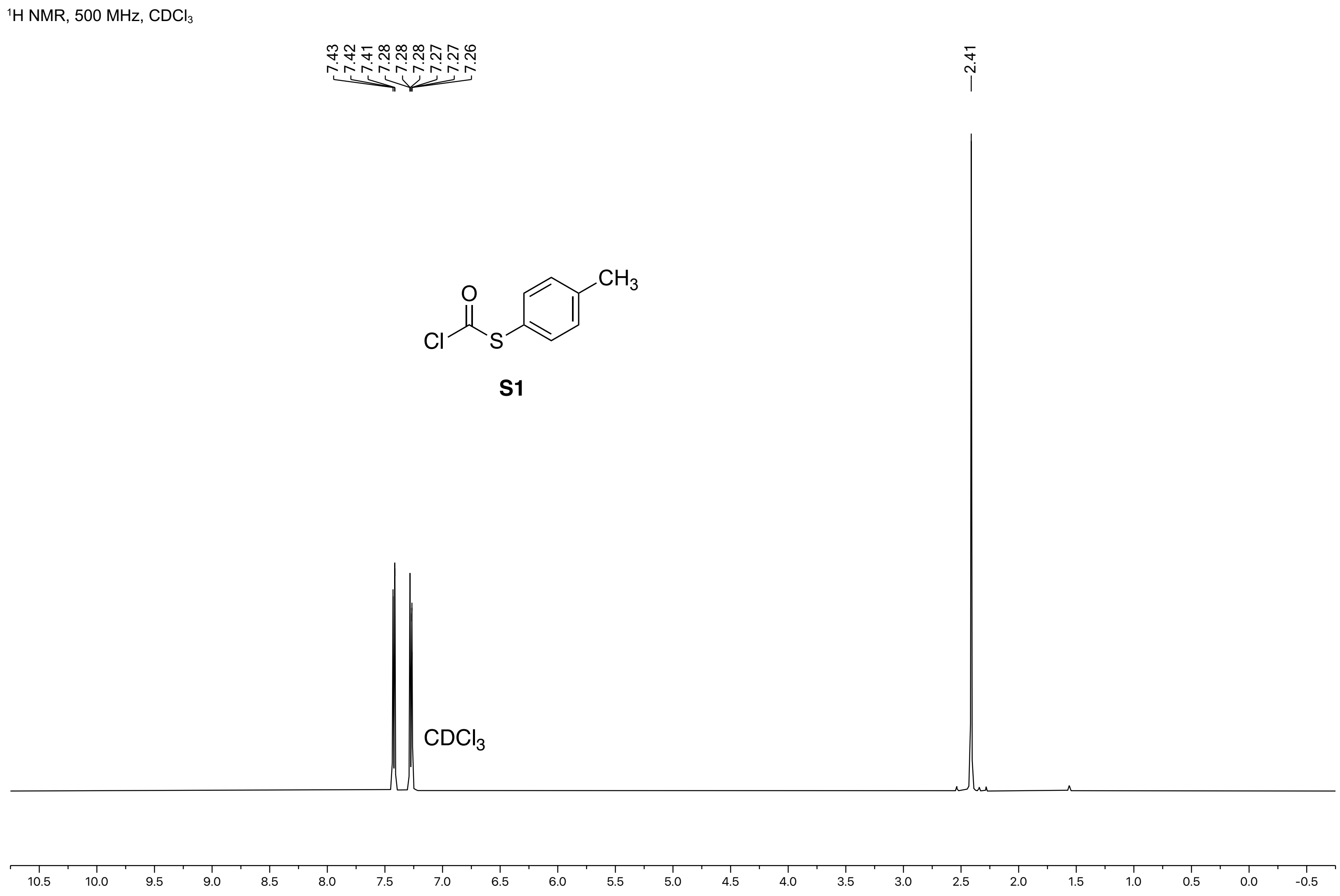

$^{13}C\{^{1}H\}$ NMR, 126 MHz, $CDCl_3$

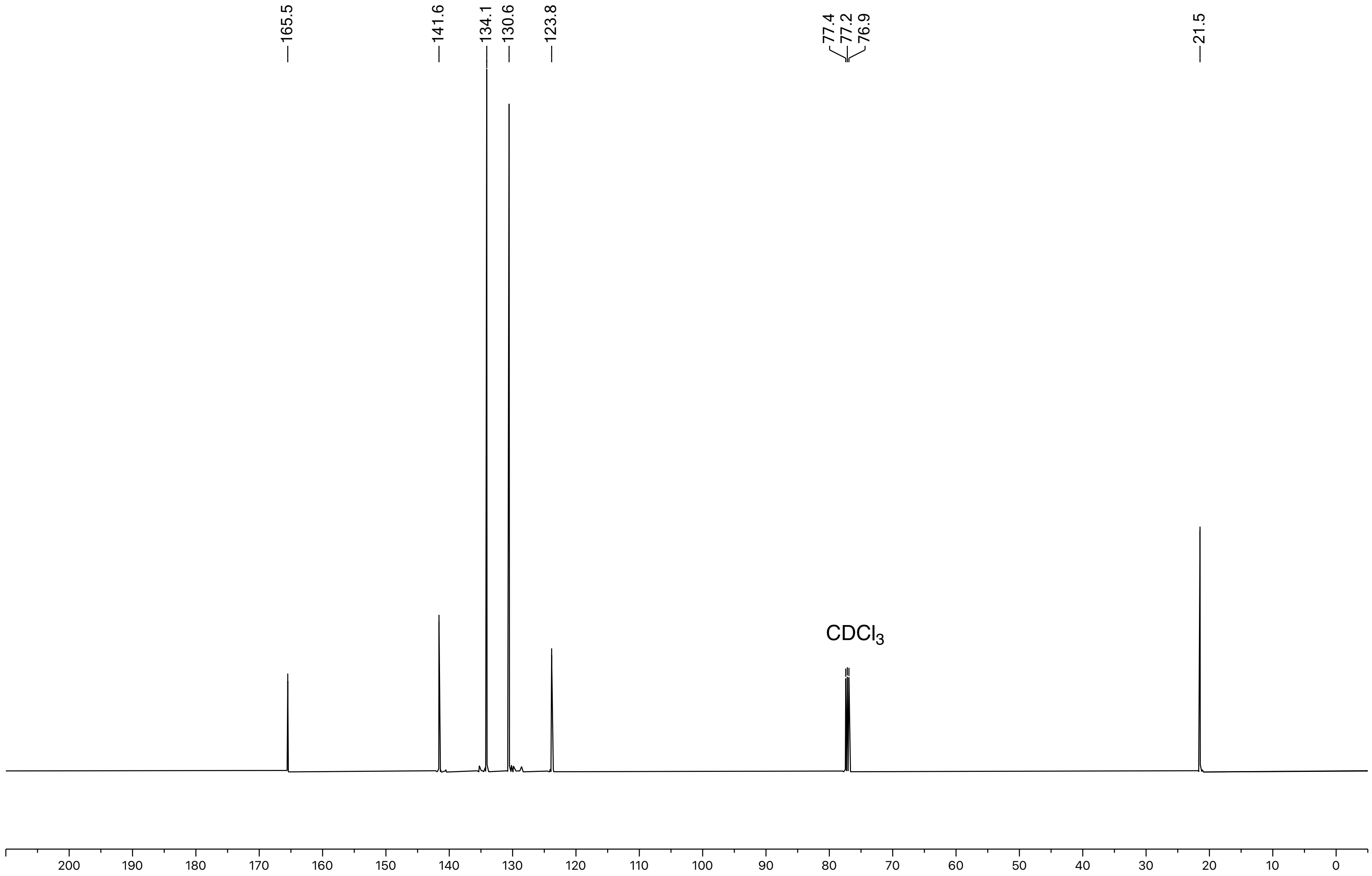

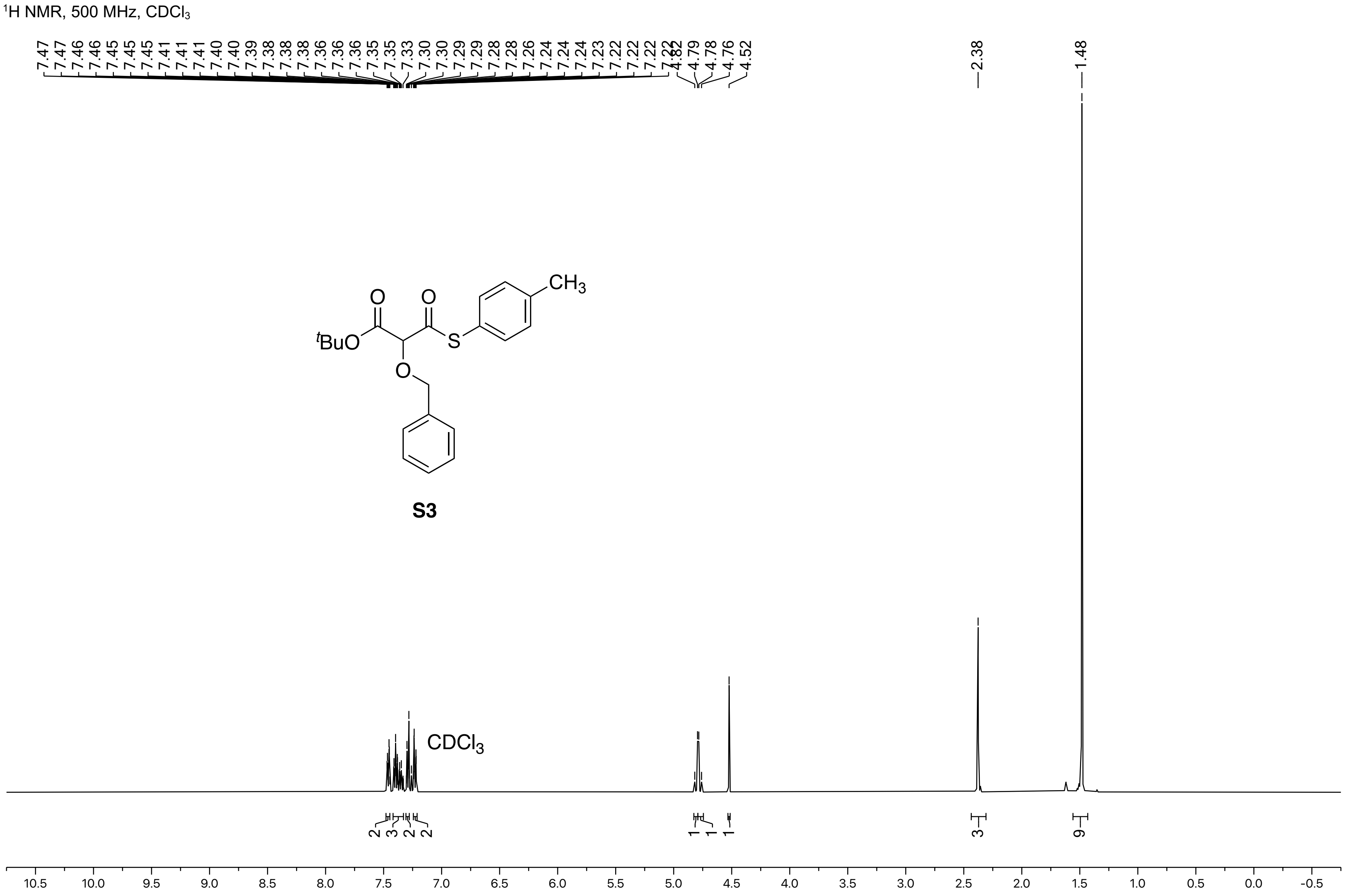

¹H NMR, 500 MHz, CDCl3
S3
CDCl3

$^{13}C\{^{1}H\}$ NMR, 126 MHz, $CDCl_3$

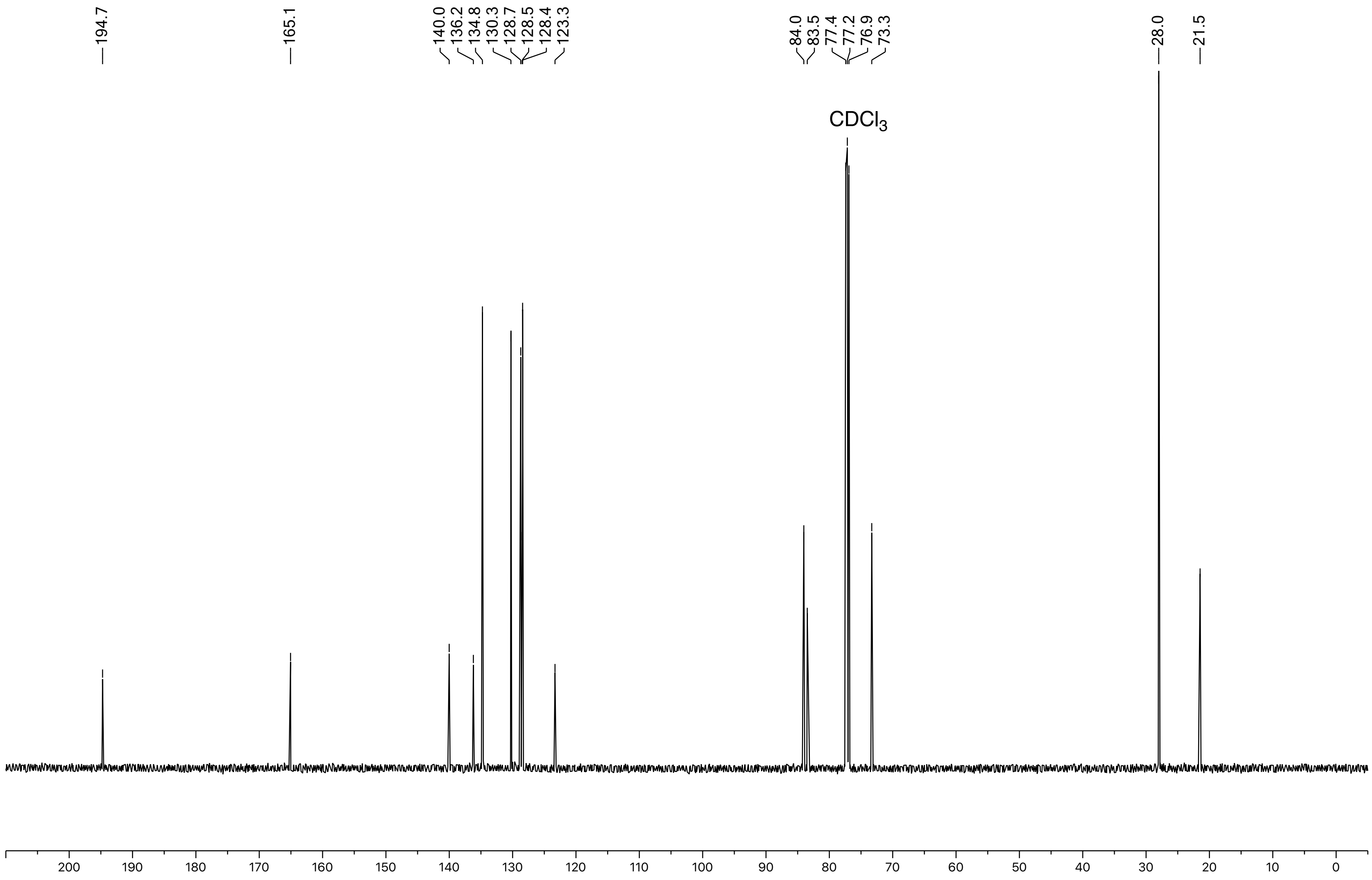

^{1}H NMR, 500 MHz, $CDCl_3$

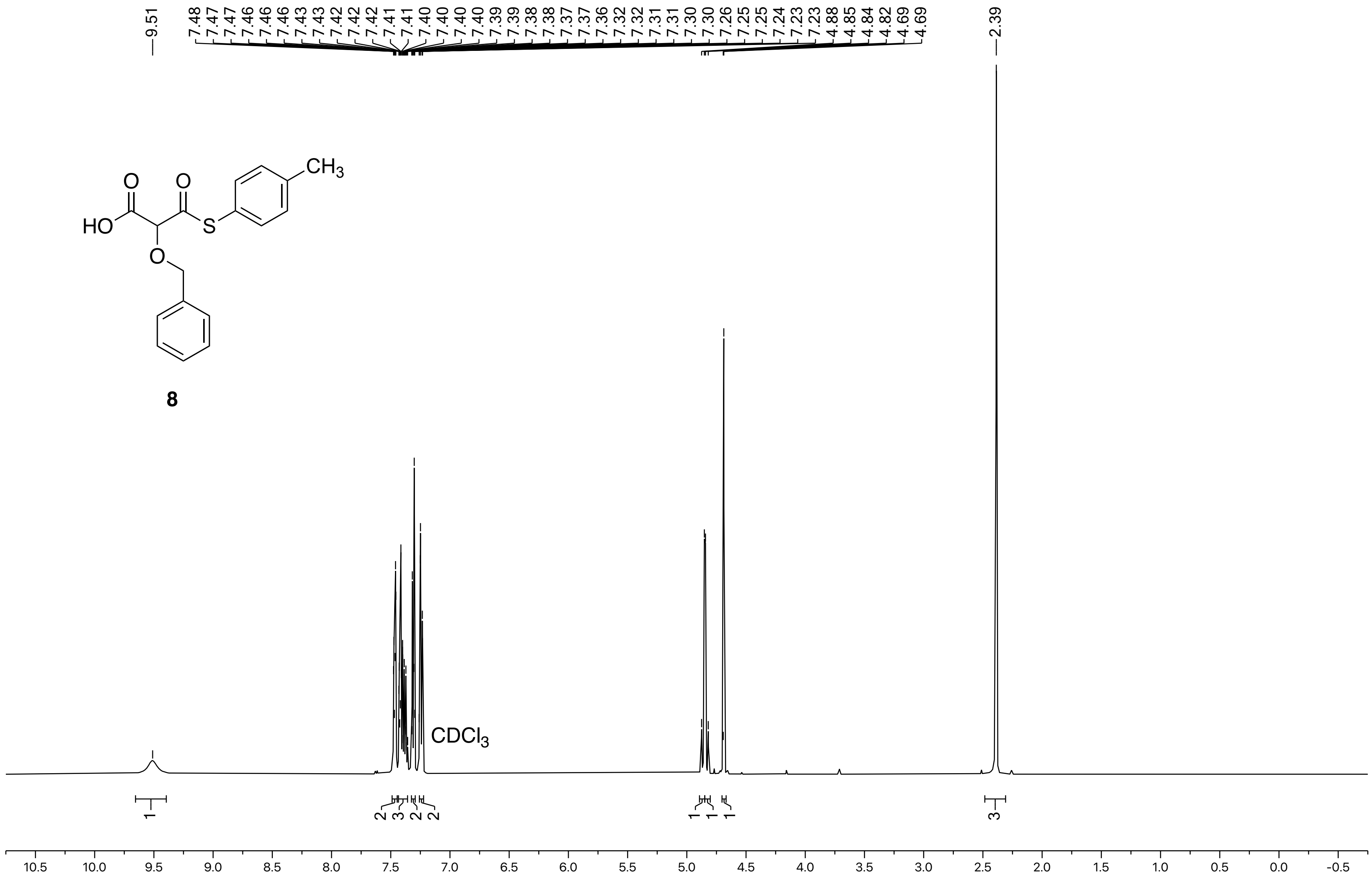

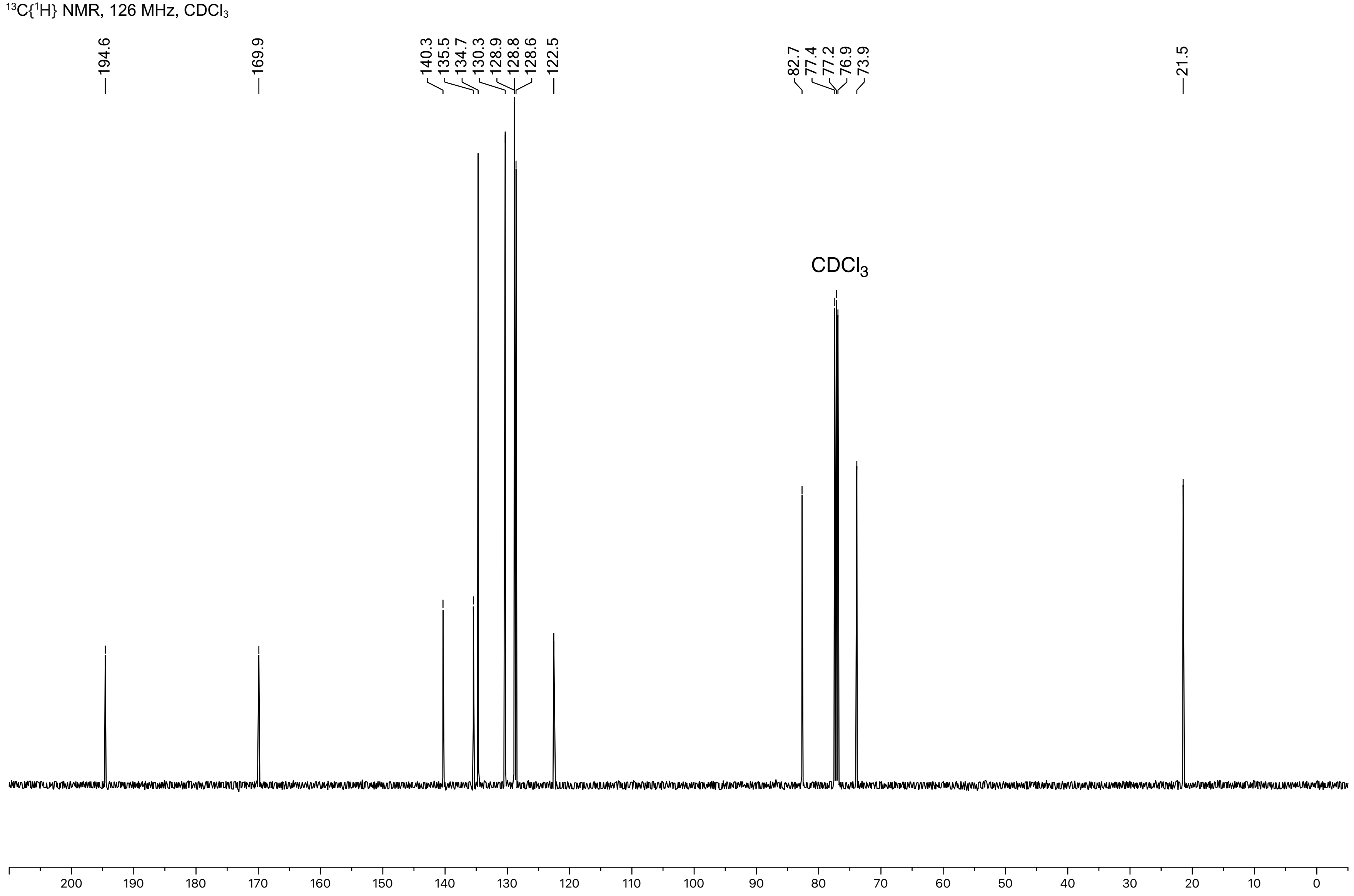

$^{13}C\{^1H\}$ NMR, 126 MHz, $CDCl_3$
194.6
169.9
140.3
135.5
134.7
130.3
128.9
128.8
128.6
122.5
82.7
77.4
77.2
76.9
73.9
21.5
$CDCl_3$
200 190 180 170 160 150 140 130 120 110 100 90 80 70 60 50 40 30 20 10 0

^{1}H NMR, 500 MHz, $CDCl_3$

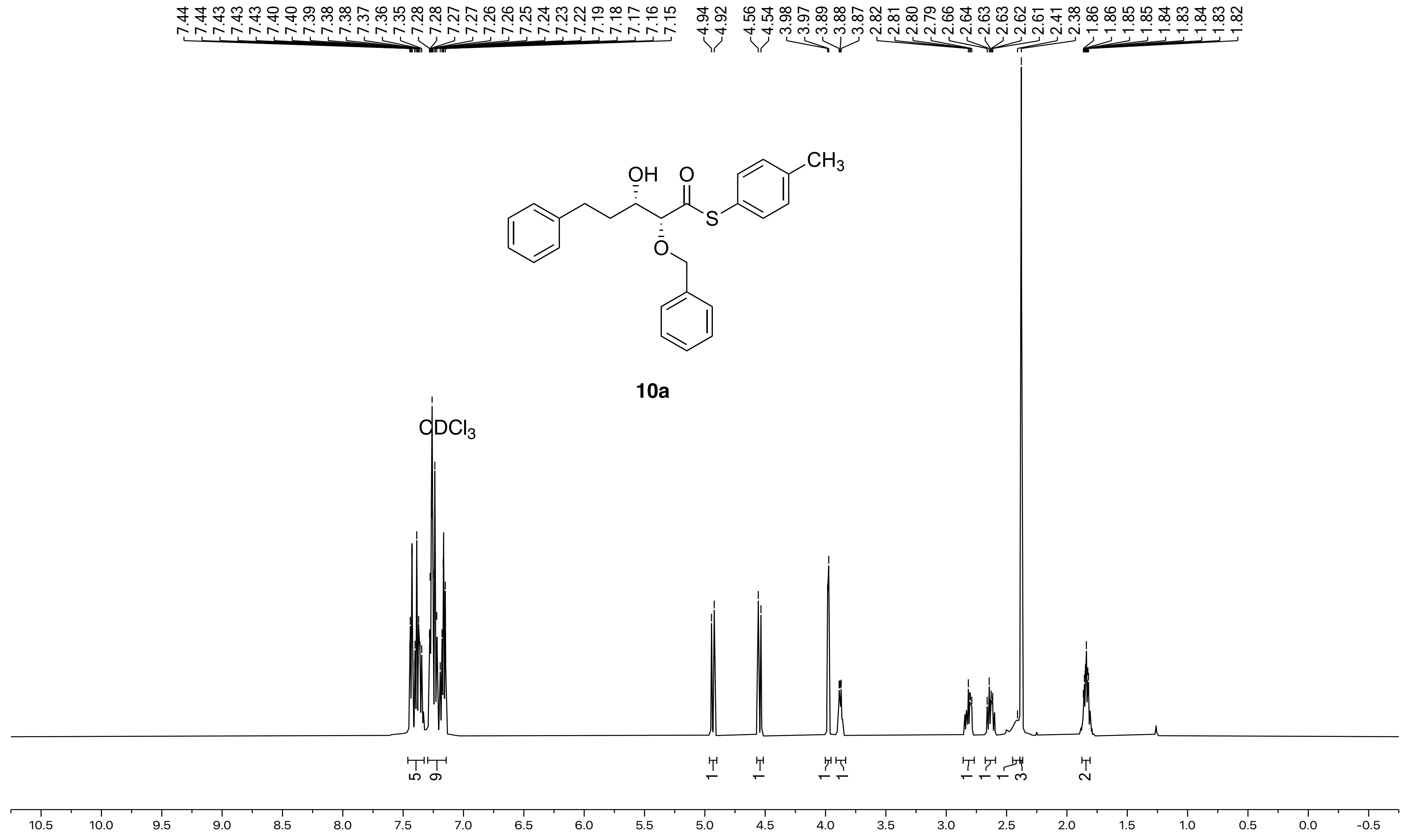

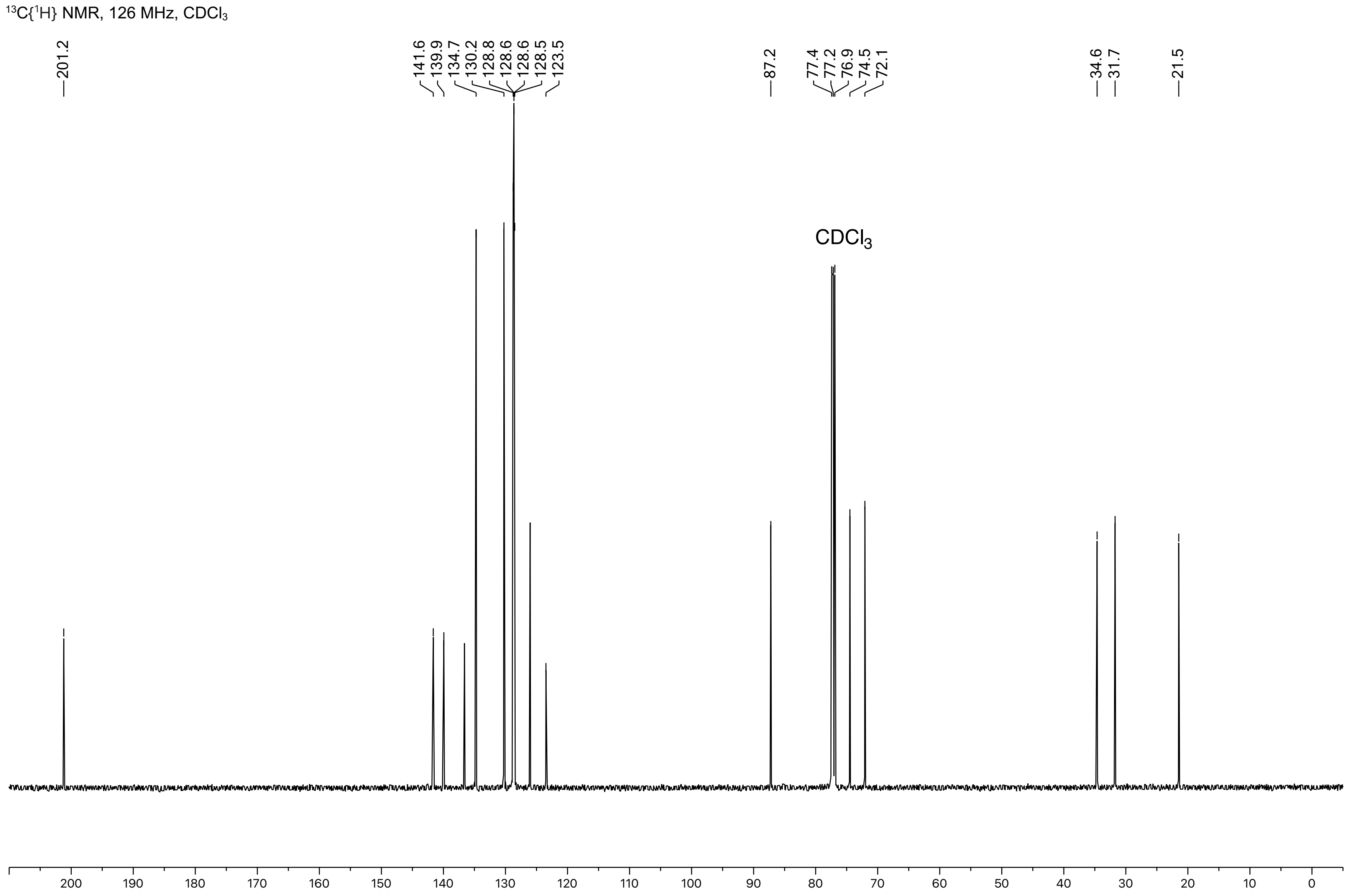
$^{13}C\{^1H\}$ NMR, 126 MHz, $CDCl_3$
201.2
141.6
139.9
134.7
130.2
128.8
128.6
128.6
128.5
123.5
87.2
77.4
77.2
76.9
74.5
72.1
34.6
31.7
21.5
$CDCl_3$
200
190
180
170
160
150
140
130
120
110
100
90
80
70
60
50
40
30
20
10
0

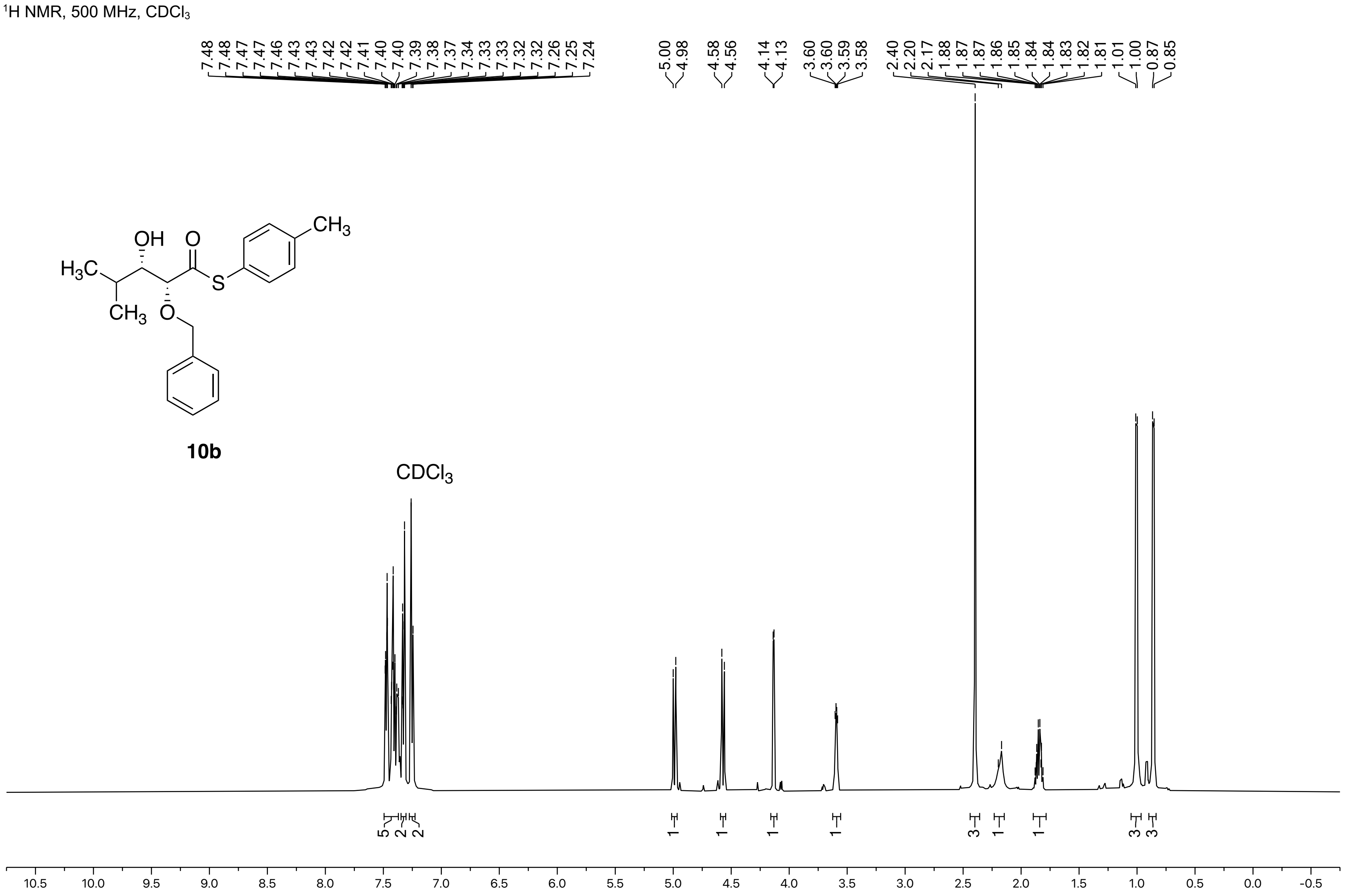

1H NMR, 500 MHz, CDCl3
7.48 7.48 7.47 7.46 7.43 7.43 7.42 7.42 7.41 7.40 7.40 7.39 7.38 7.37 7.34 7.33 7.33 7.32 7.32 7.26 7.25 7.24
5.00 4.98
4.58 4.56
4.14 4.13
3.60 3.60 3.59 3.58
2.40 2.20 2.17 1.88 1.87 1.87 1.86 1.85 1.84 1.84 1.83 1.82 1.81
1.01 1.00 0.87 0.85
OH
O
CH3
H3C
S
CH3
O
10b
CDCl3
5 2 2
1
1
1
1
3 1
1
3 3
10.5 10.0 9.5 9.0 8.5 8.0 7.5 7.0 6.5 6.0 5.5 5.0 4.5 4.0 3.5 3.0 2.5 2.0 1.5 1.0 0.5 0.0 -0.5

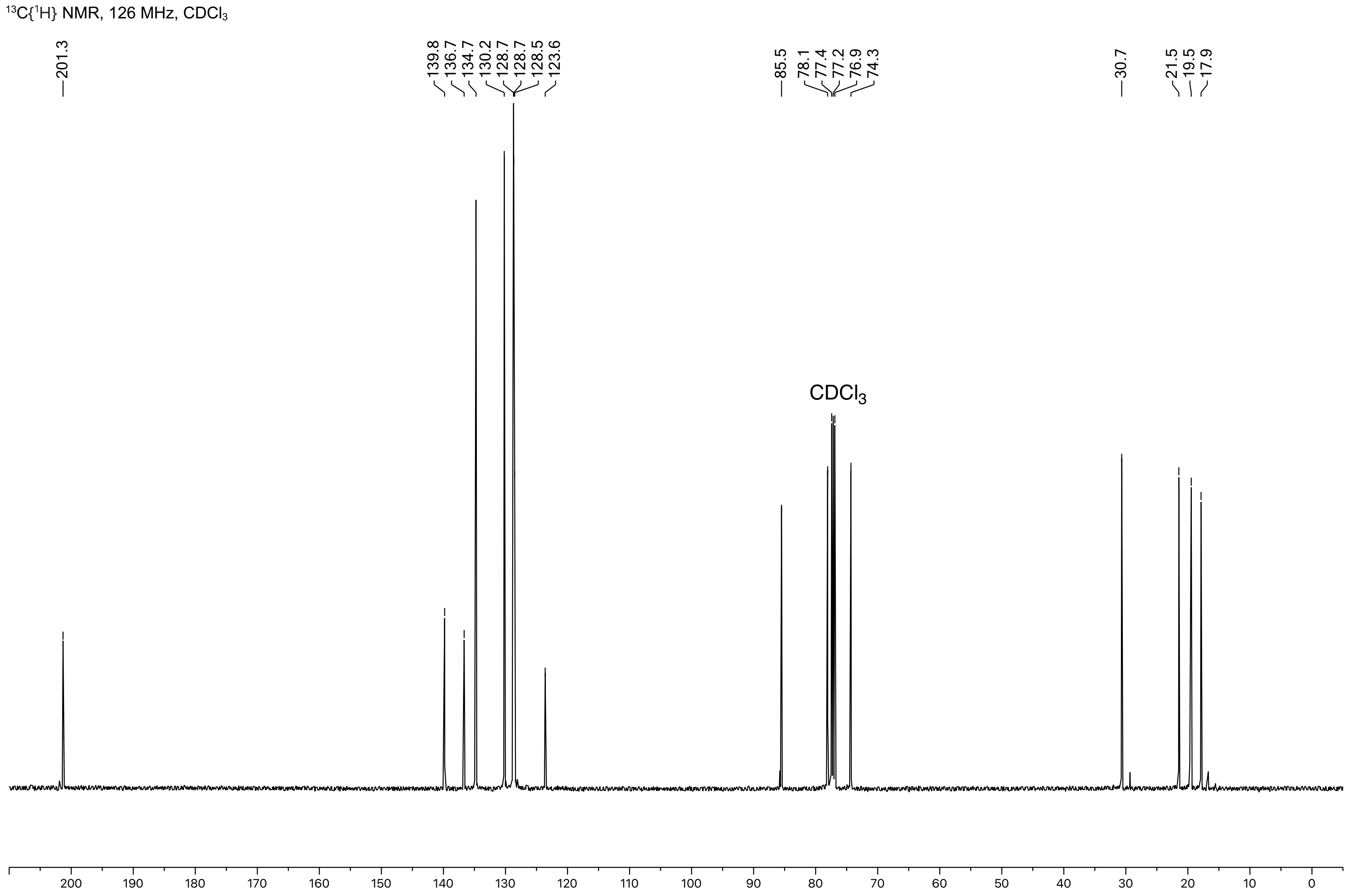

$^{13}C\{^{1}H\}$ NMR, 126 MHz, $CDCl_3$
201.3
139.8
136.7
134.7
130.2
128.7
128.7
128.5
123.6
85.5
78.1
77.4
77.2
76.9
74.3
30.7
21.5
19.5
17.9
$CDCl_3$
200 190 180 170 160 150 140 130 120 110 100 90 80 70 60 50 40 30 20 10 0

^{1}H NMR, 500 MHz, $CDCl_3$

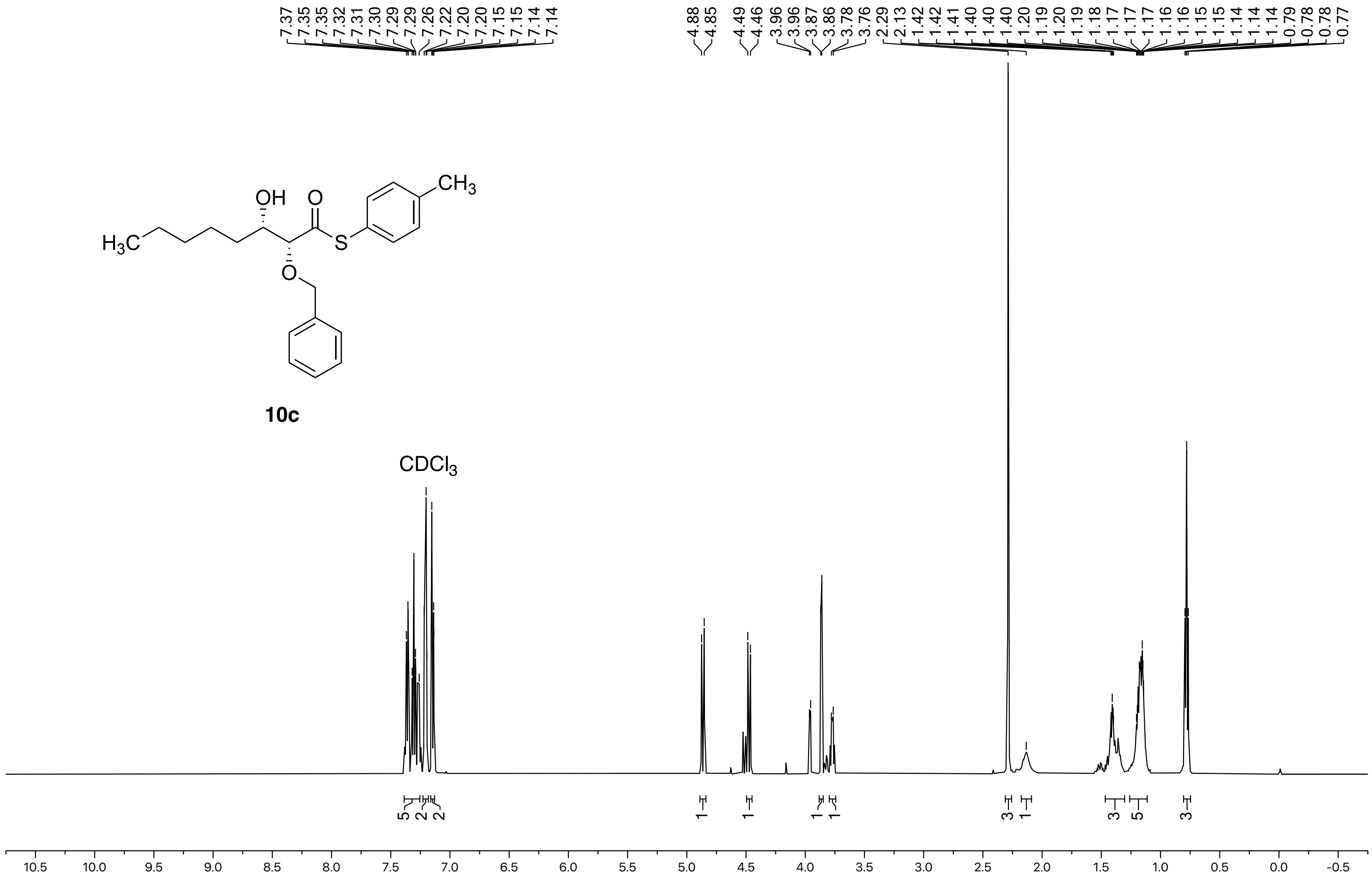

10c

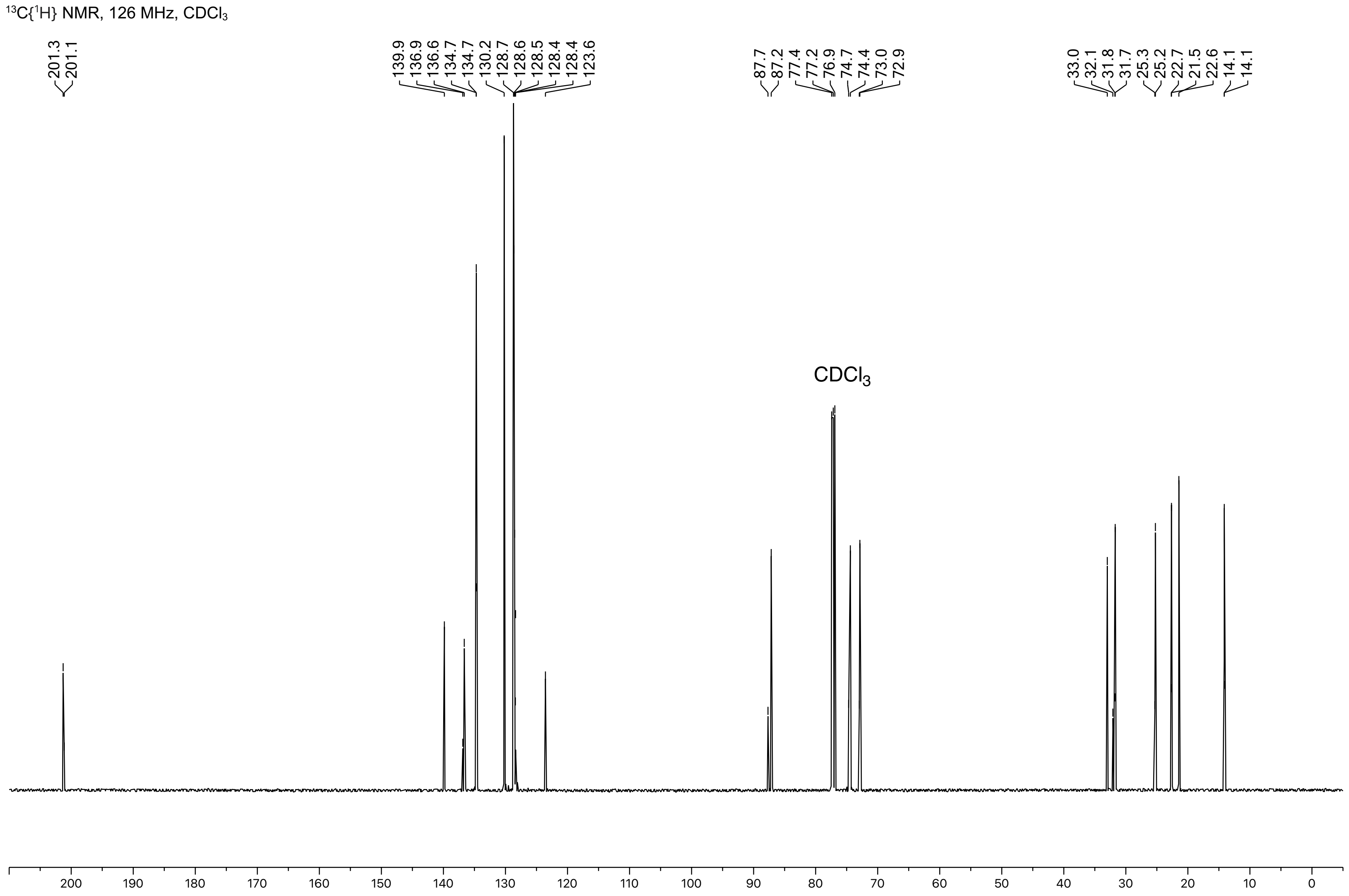

$^{13}C\{^1H\}$ NMR, 126 MHz, $CDCl_3$
201.3
201.1
139.9
136.9
136.6
134.7
134.7
130.2
128.7
128.6
128.5
128.4
128.4
123.6
87.7
87.2
77.4
77.2
76.9
74.7
74.4
73.0
72.9
33.0
32.1
31.8
31.7
25.3
25.2
22.7
21.5
22.6
14.1
14.1
$CDCl_3$
200
190
180
170
160
150
140
130
120
110
100
90
80
70
60
50
40
30
20
10
0

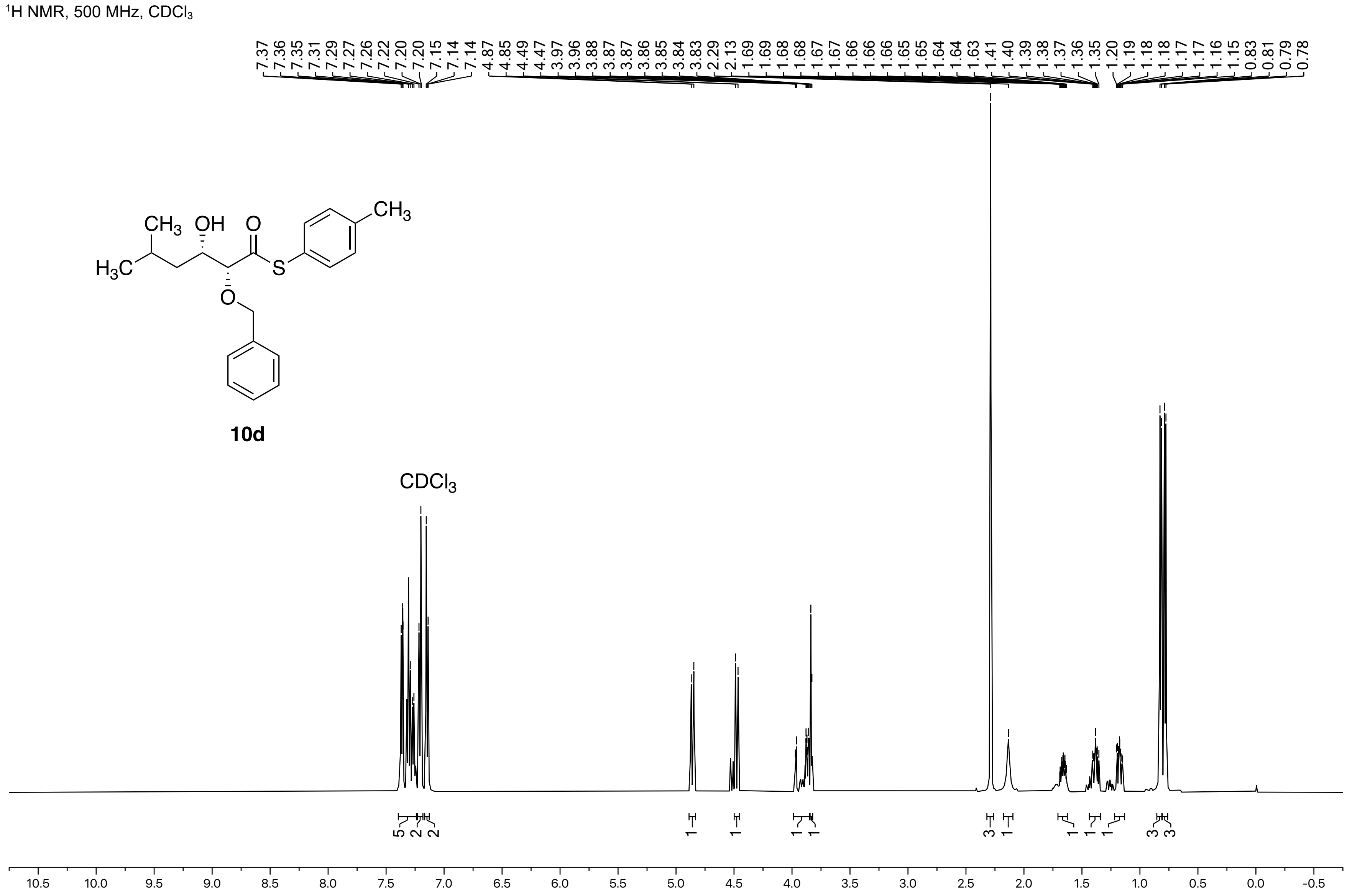

^{1}H NMR, 500 MHz, $CDCl_3$
10d
$CDCl_3$

$^{13}C\{^1H\}$ NMR, 126 MHz, $CDCl_3$

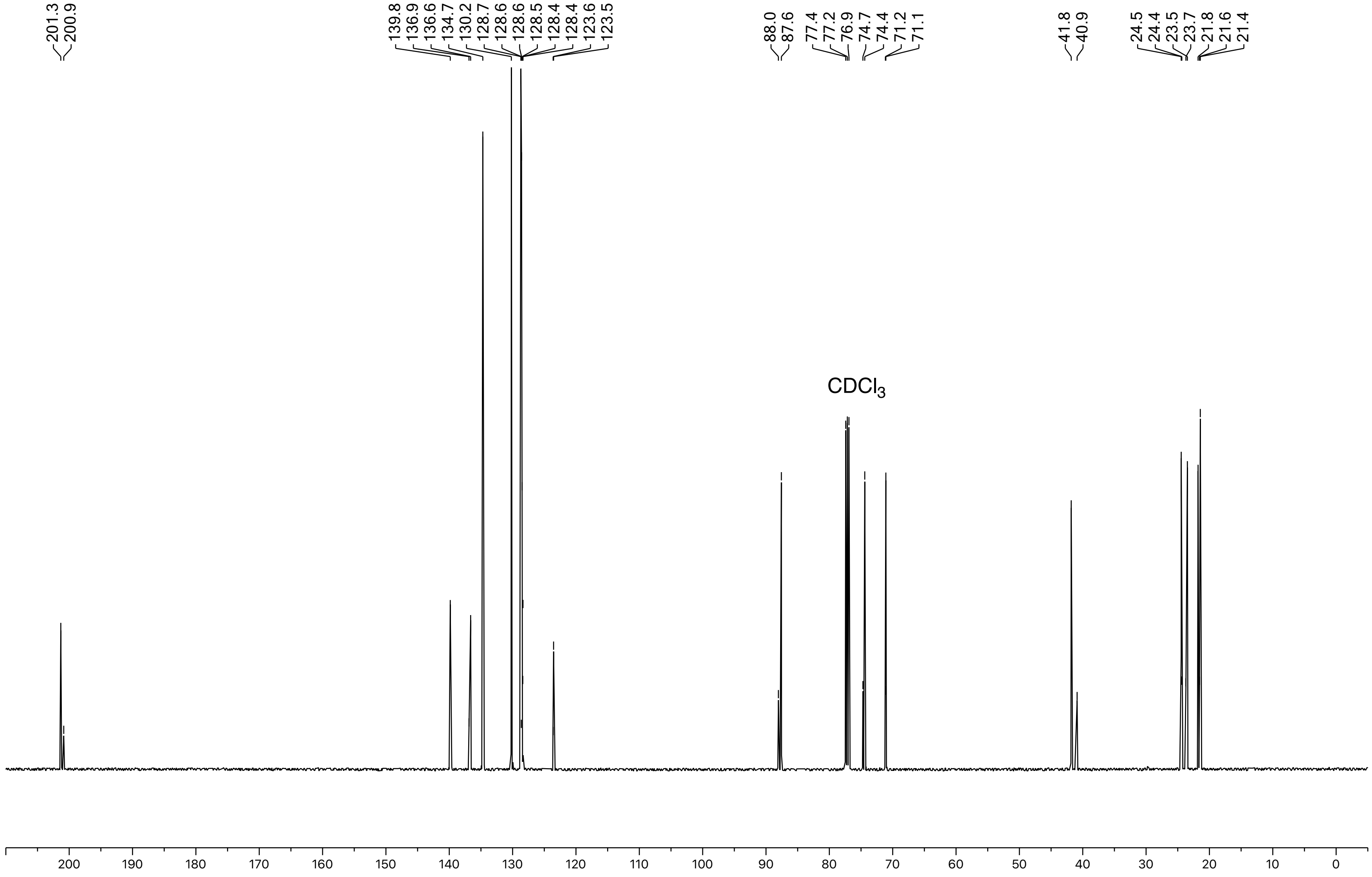

^1H NMR, 500 MHz, $CDCl_3$

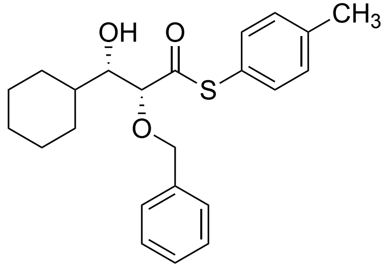

10e

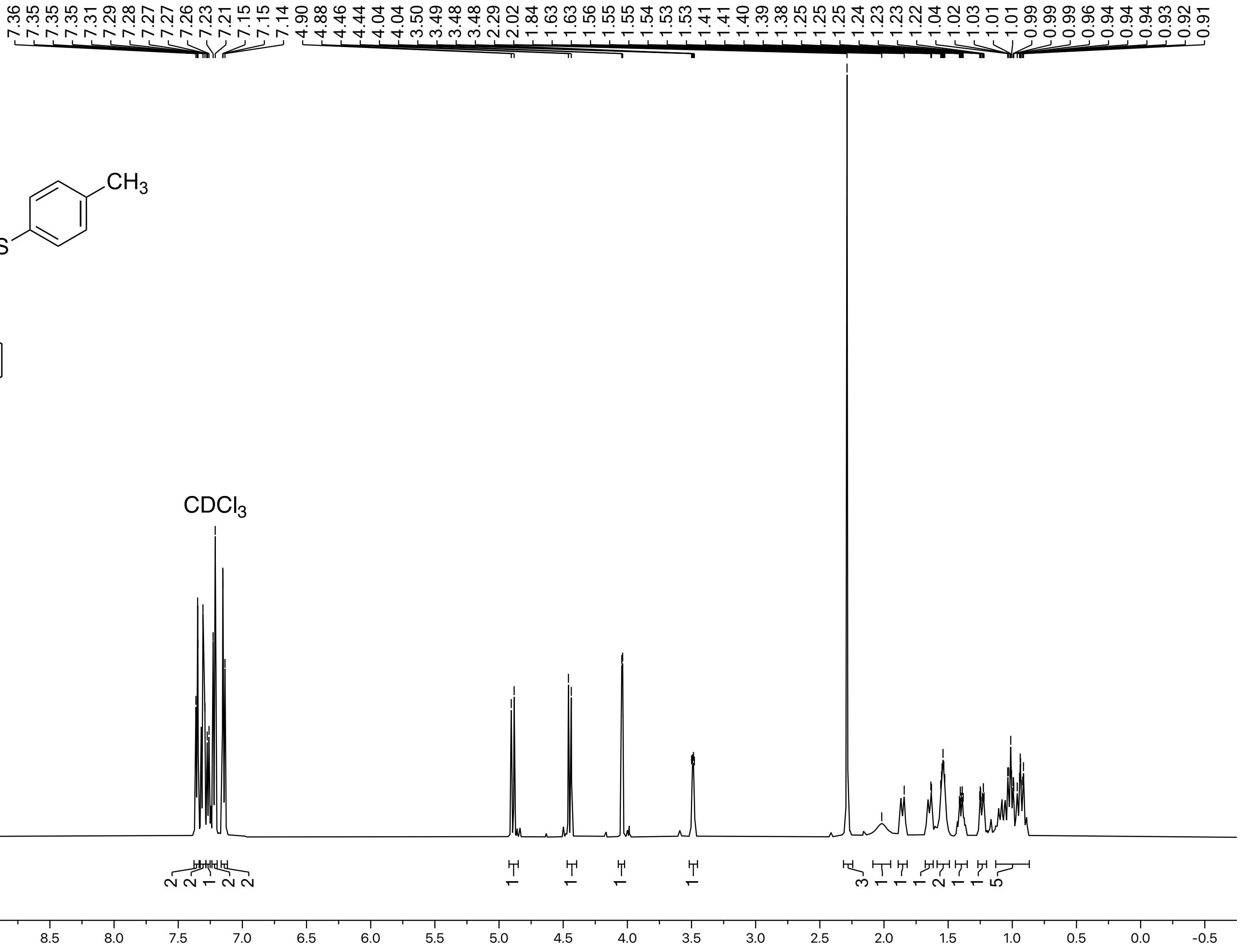

$^{13}C\{^{1}H\}$ NMR, 126 MHz, $CDCl_3$

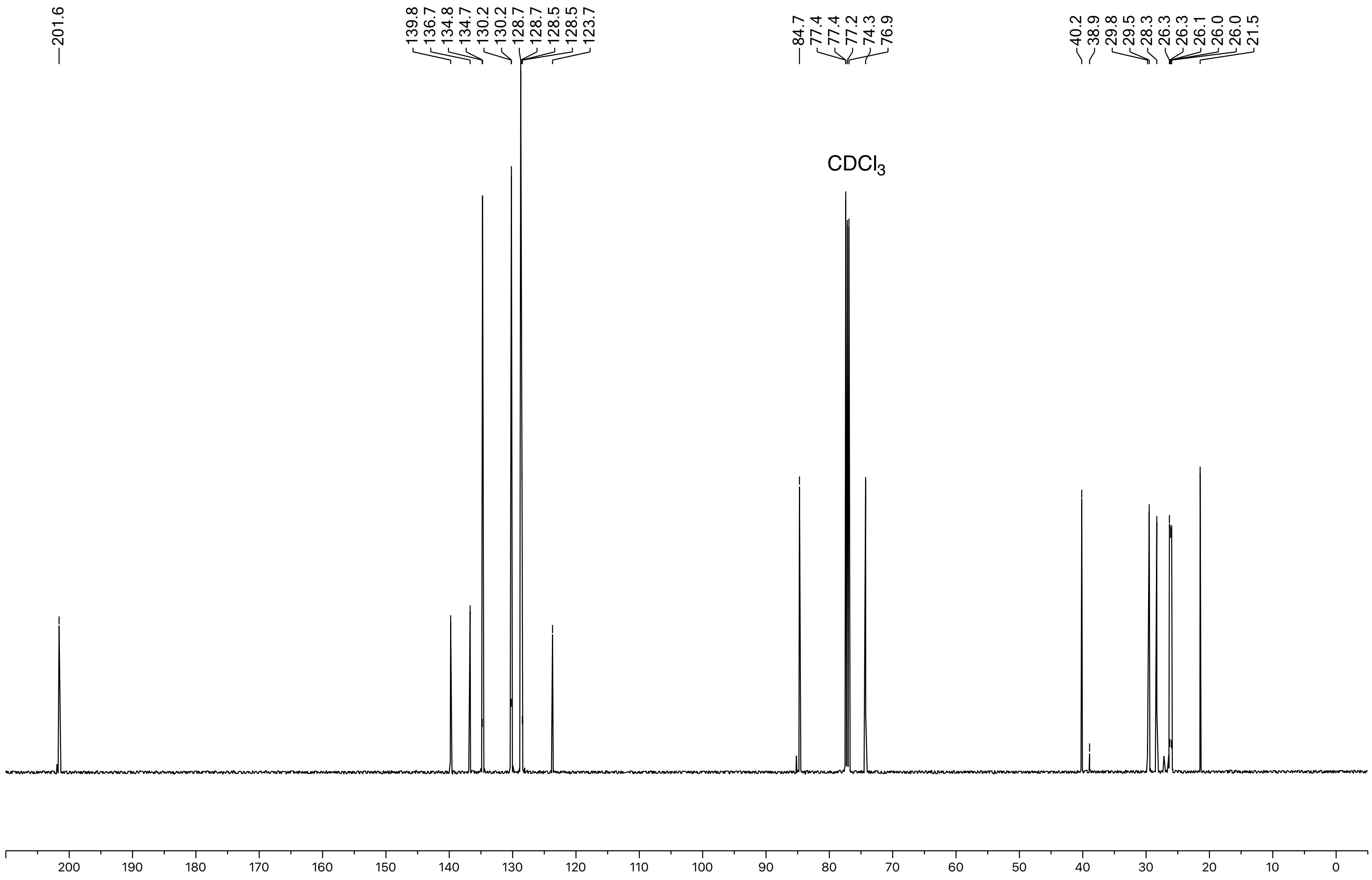

^1H NMR, 500 MHz, $CDCl_3$

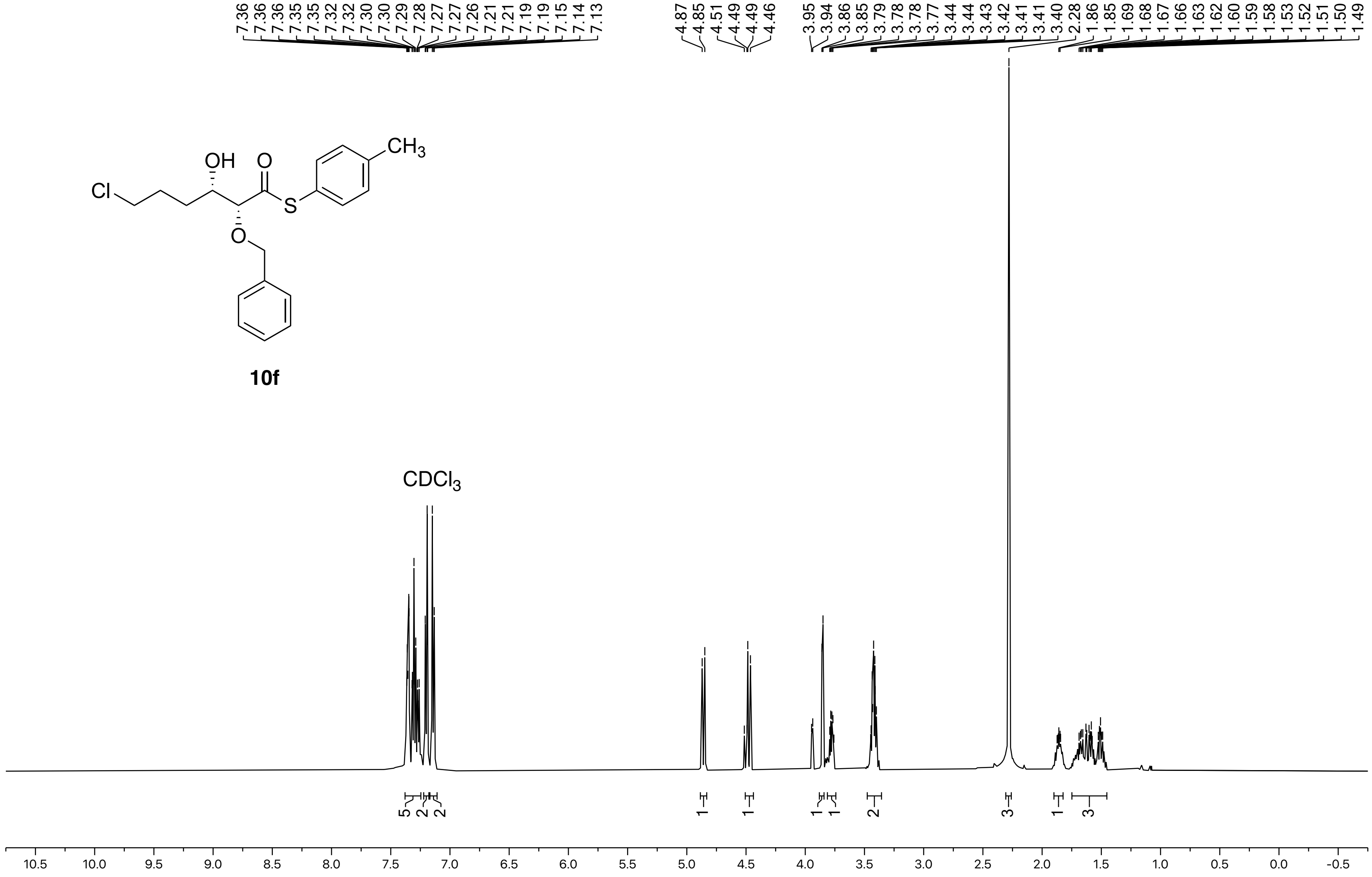

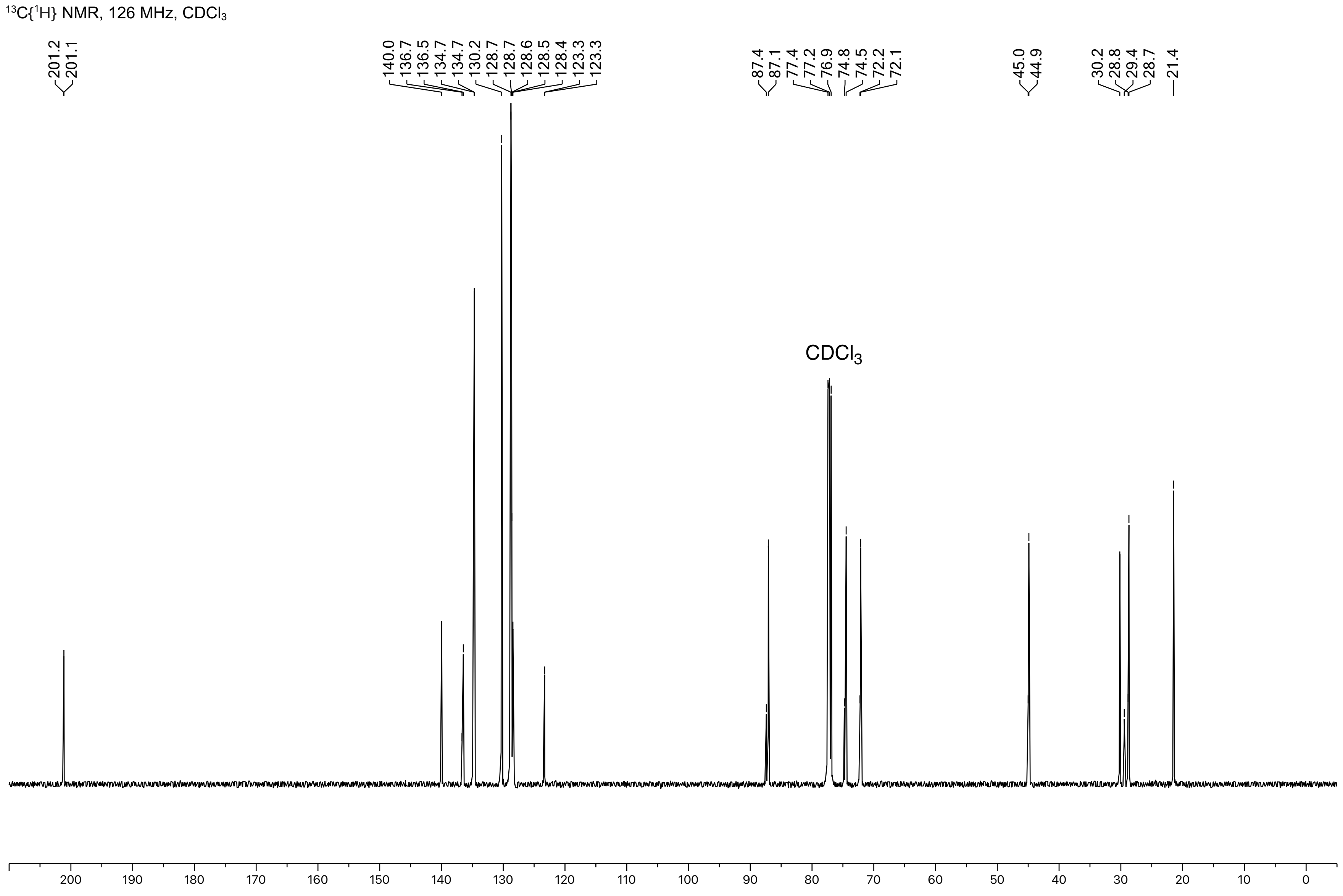
$^{13}C\{^1H\}$ NMR, 126 MHz, $CDCl_3$
201.2
201.1
140.0
136.7
136.5
134.7
134.7
130.2
128.7
128.7
128.6
128.5
128.4
123.3
123.3
87.4
87.1
77.4
77.2
76.9
74.8
74.5
72.2
72.1
45.0
44.9
30.2
28.8
29.4
28.7
21.4
$CDCl_3$
200 190 180 170 160 150 140 130 120 110 100 90 80 70 60 50 40 30 20 10 0

^{1}H NMR, 500 MHz, $CDCl_3$

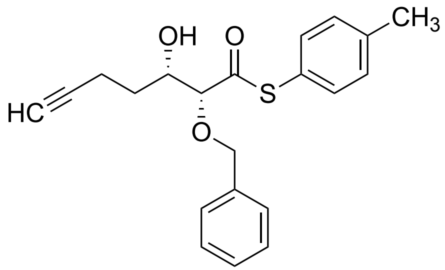

10g

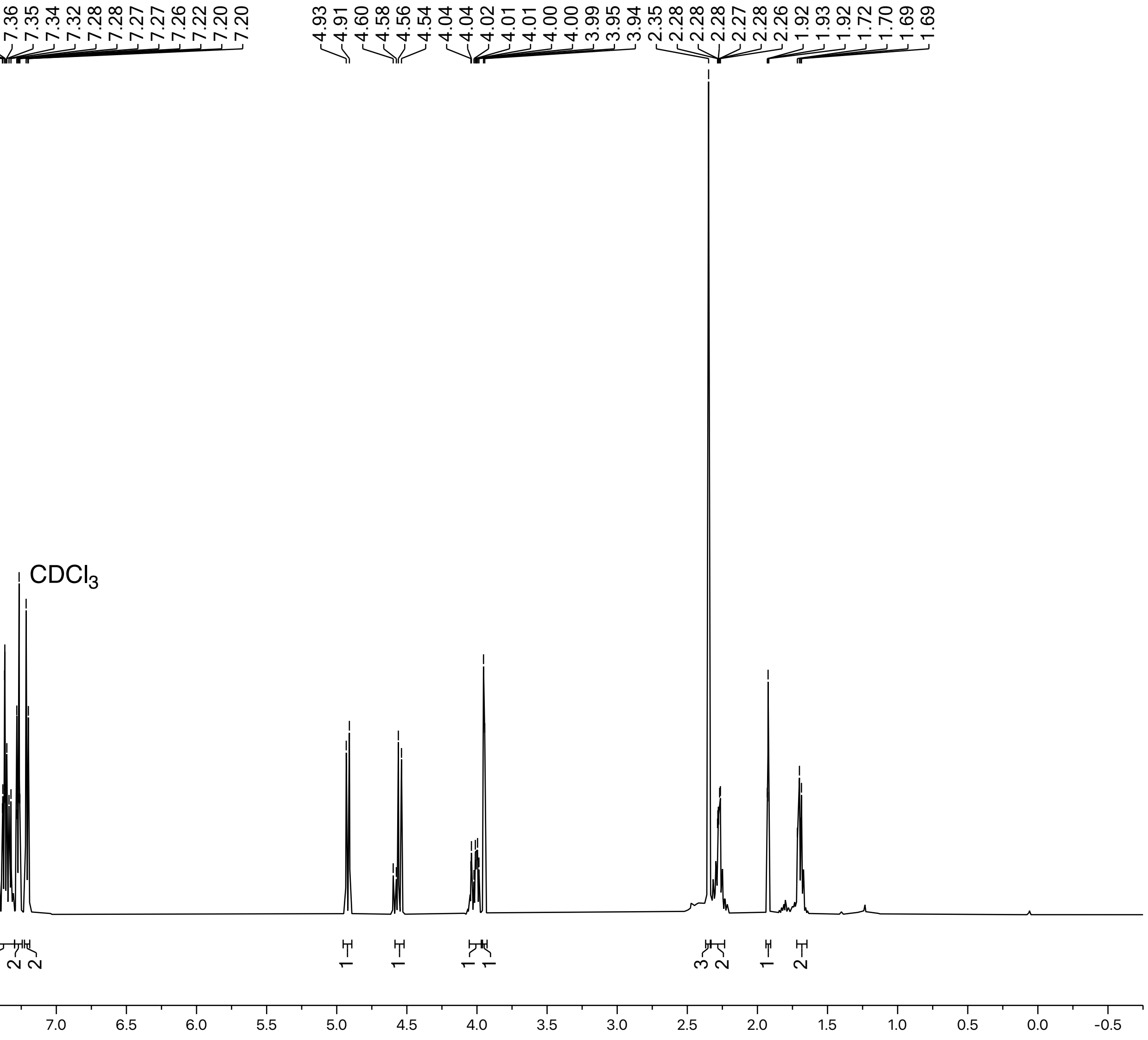

$^{13}C\{^1H\}$ NMR, 126 MHz, $CDCl_3$

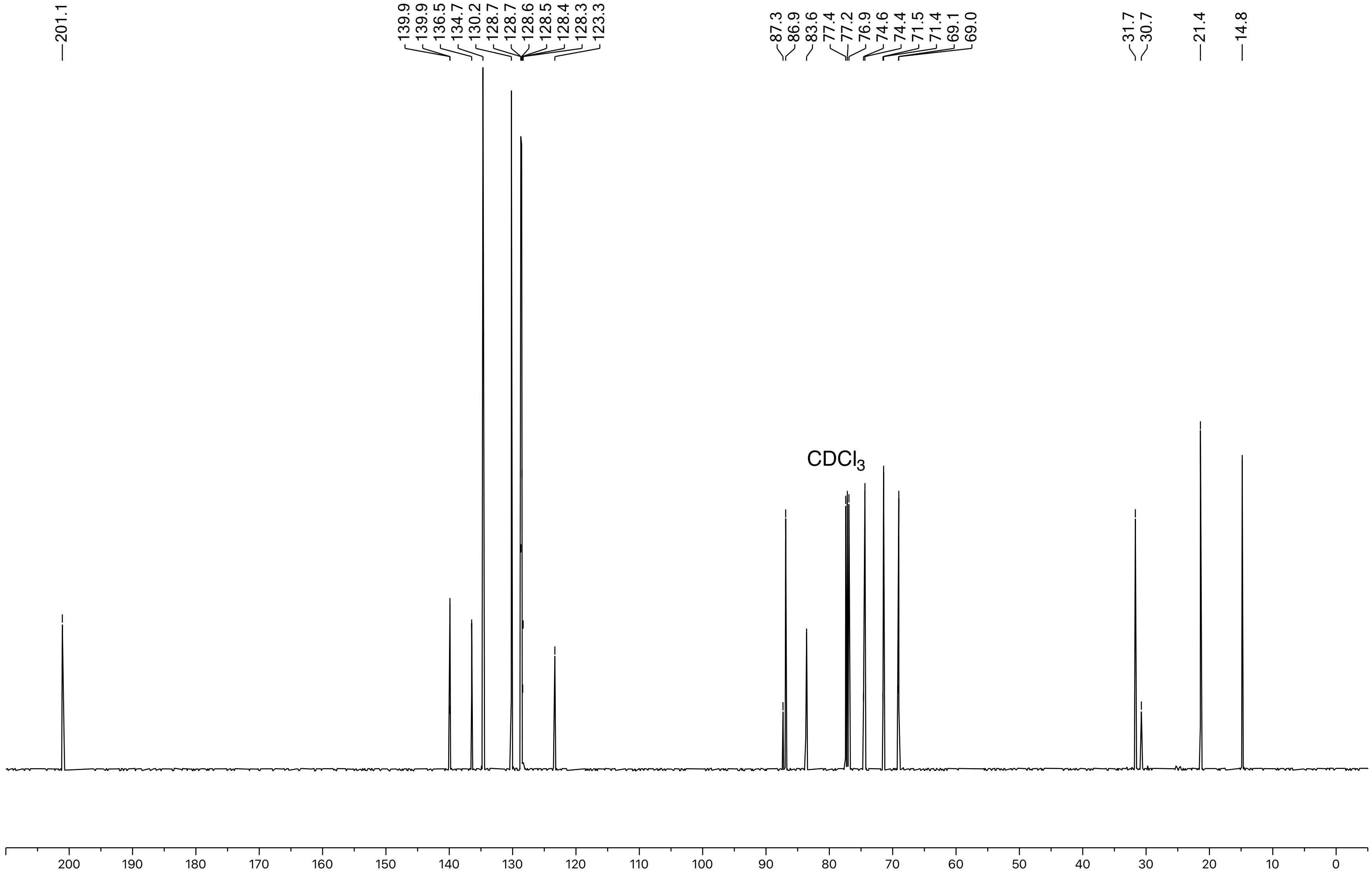

^1H NMR, 500 MHz, $CDCl_3$

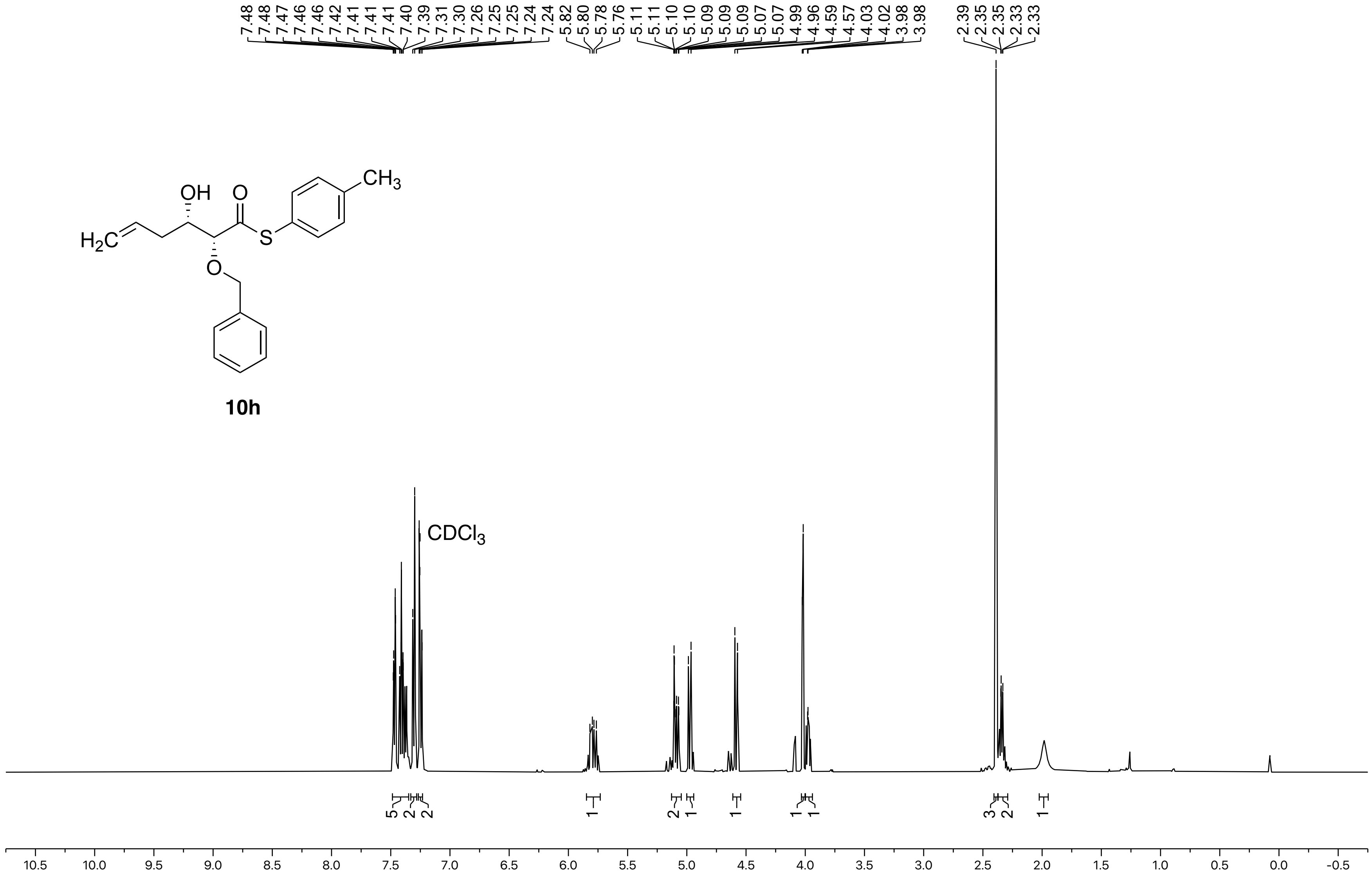

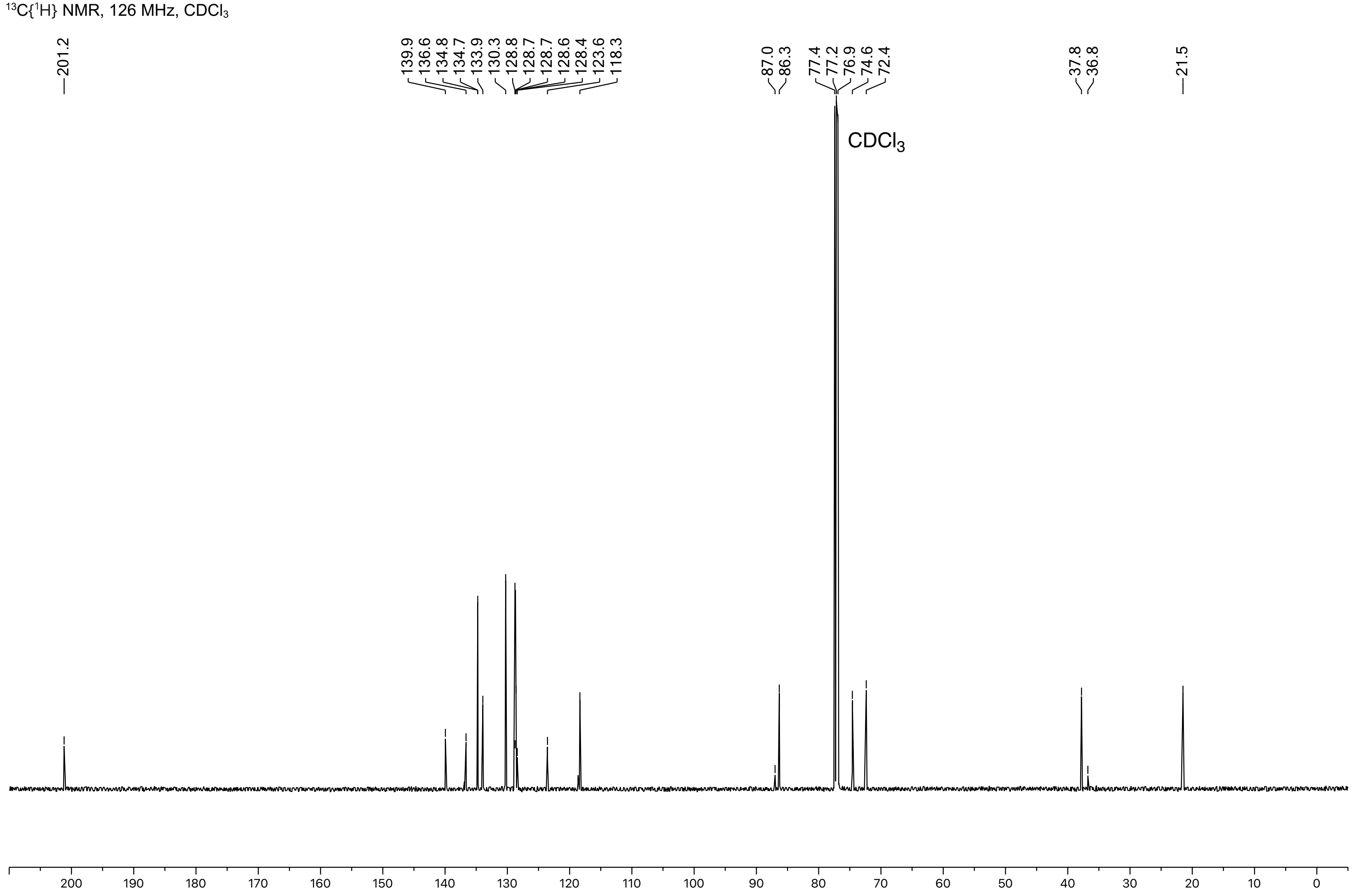
$^{13}C\{^1H\}$ NMR, 126 MHz, $CDCl_3$
201.2
139.9
136.6
134.8
134.7
133.9
130.3
128.8
128.7
128.7
128.6
128.4
123.6
118.3
87.0
86.3
77.4
77.2
76.9
74.6
72.4
37.8
36.8
21.5
$CDCl_3$
200 190 180 170 160 150 140 130 120 110 100 90 80 70 60 50 40 30 20 10 0

^{1}H NMR, 500 MHz, $CDCl_3$

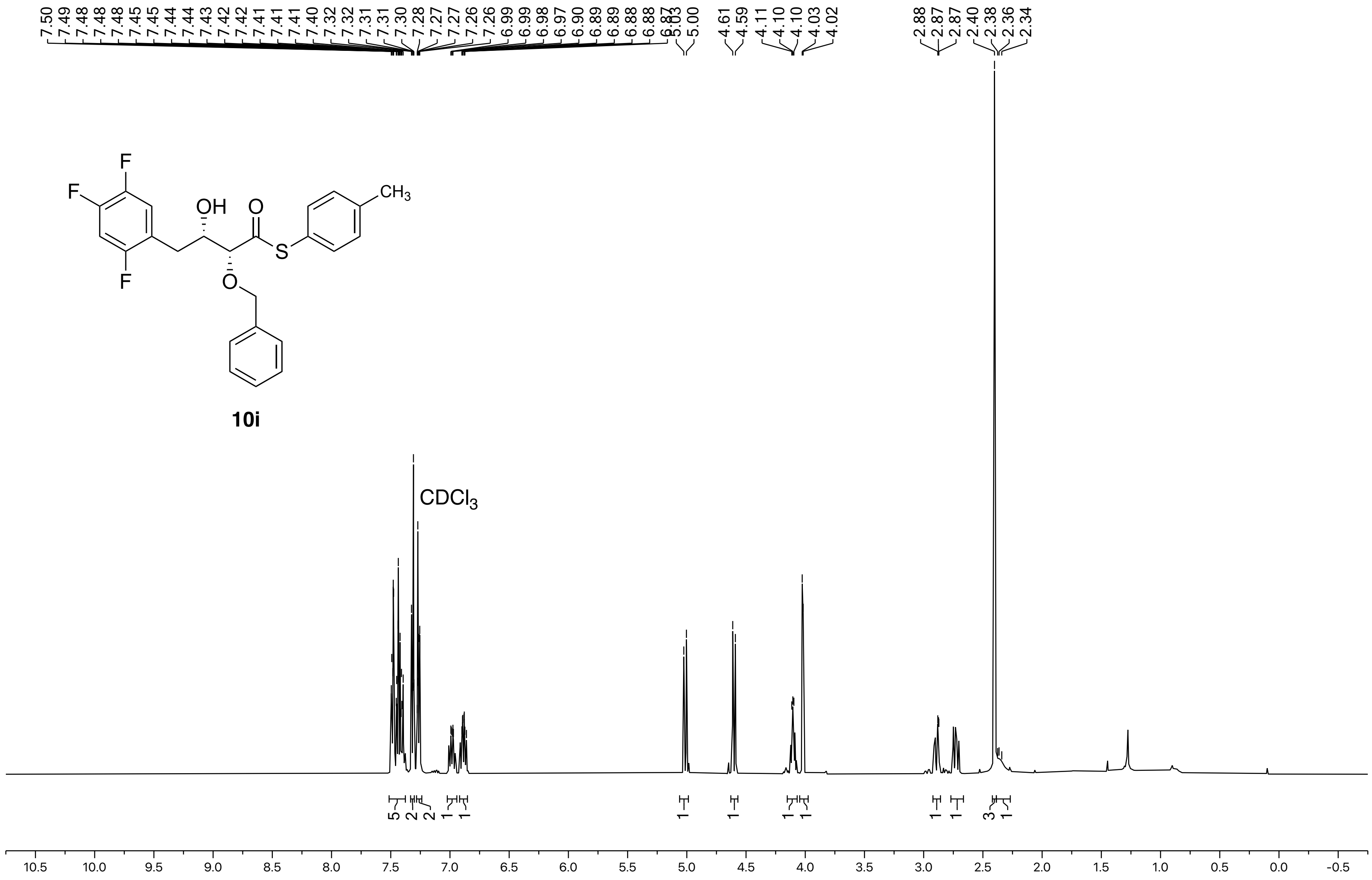

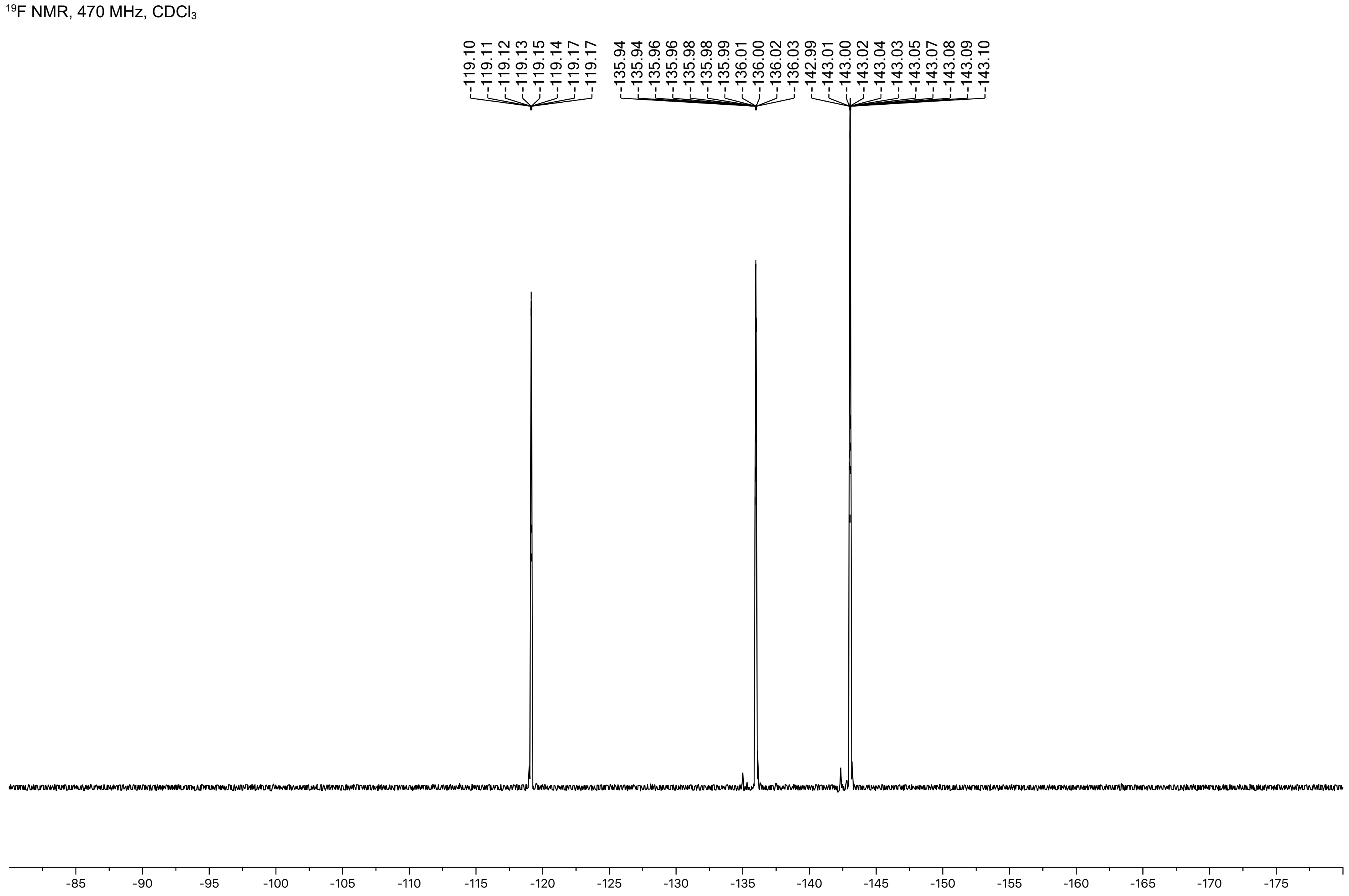

^{19}F NMR, 470 MHz, CDCl$_3$
-119.10
-119.11
-119.12
-119.13
-119.15
-119.14
-119.17
-119.17
-135.94
-135.94
-135.96
-135.96
-135.98
-135.98
-135.99
-136.01
-136.00
-136.02
-136.03
-142.99
-143.01
-143.00
-143.02
-143.04
-143.03
-143.05
-143.07
-143.08
-143.09
-143.10
-85 -90 -95 -100 -105 -110 -115 -120 -125 -130 -135 -140 -145 -150 -155 -160 -165 -170 -175

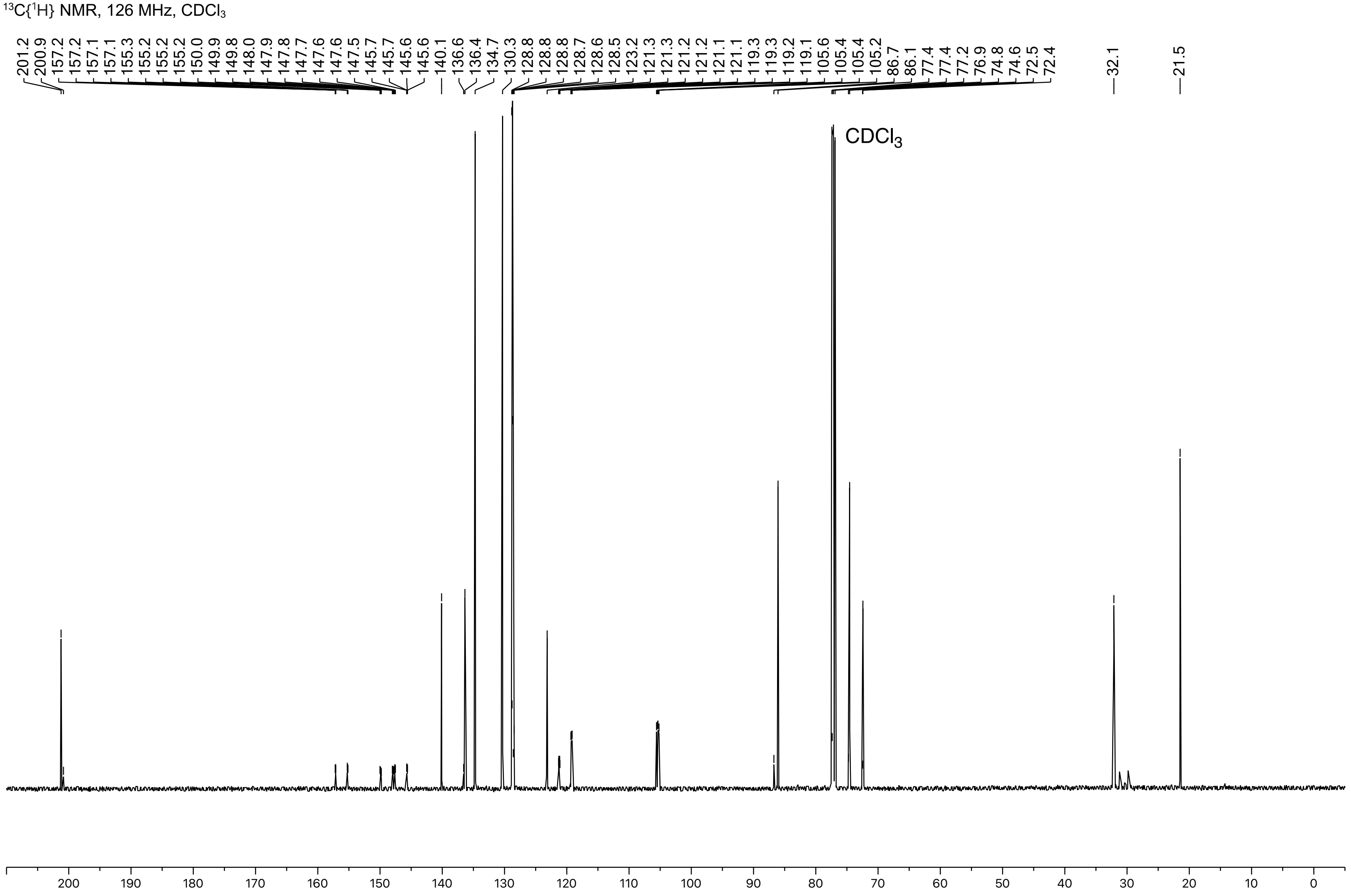

$^{13}C\{^{1}H\}$ NMR, 126 MHz, $CDCl_3$
201.2
200.9
157.2
157.2
157.1
157.1
155.3
155.2
155.2
155.2
150.0
149.9
149.8
148.0
147.9
147.8
147.7
147.6
147.6
147.5
145.7
145.7
145.6
145.6
140.1
136.6
136.4
134.7
130.3
128.8
128.8
128.8
128.7
128.6
128.5
123.2
121.3
121.3
121.2
121.2
121.1
121.1
119.3
119.3
119.2
119.1
105.6
105.4
105.4
105.2
86.7
86.1
77.4
77.4
77.2
76.9
74.8
74.6
72.5
72.4
32.1
21.5
$CDCl_3$
200
190
180
170
160
150
140
130
120
110
100
90
80
70
60
50
40
30
20
10
0

^{1}H NMR, 500 MHz, $CDCl_3$

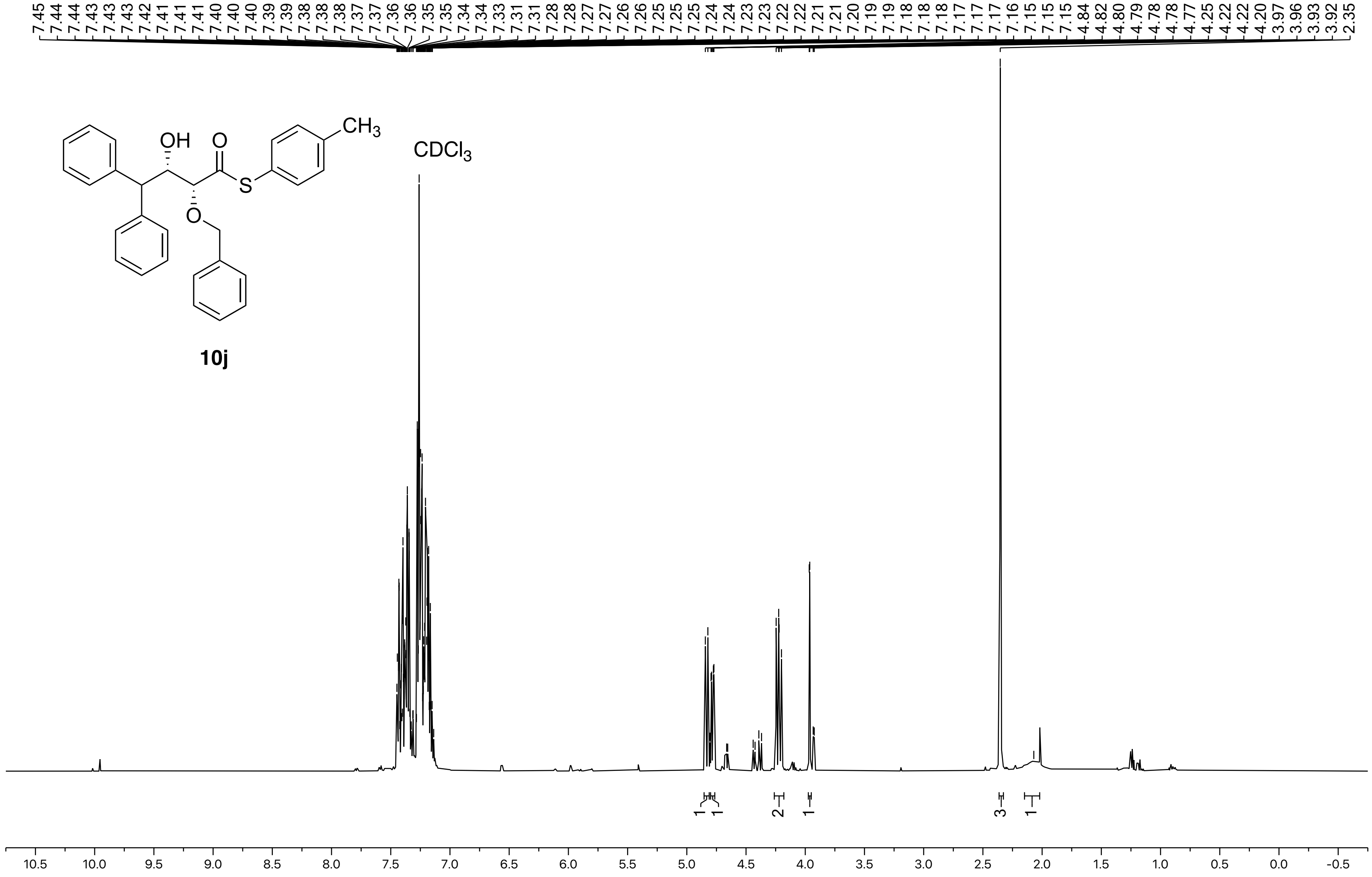

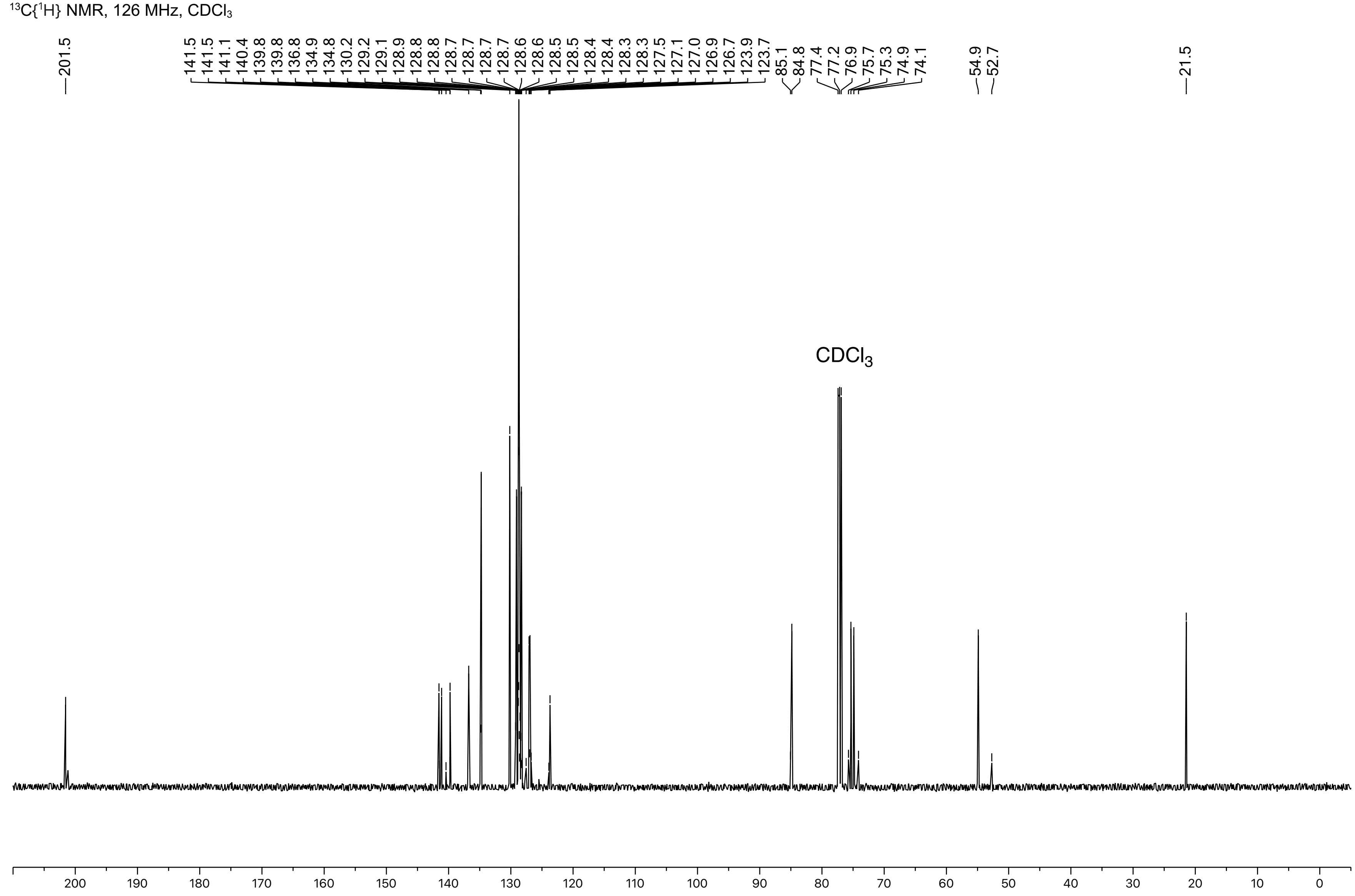

$^{13}C\{^{1}H\}$ NMR, 126 MHz, $CDCl_3$
201.5
141.5
141.5
141.1
140.4
139.8
139.8
136.8
134.9
134.8
130.2
129.2
129.1
128.9
128.8
128.8
128.7
128.7
128.7
128.7
128.6
128.6
128.5
128.5
128.4
128.4
128.3
128.3
127.5
127.1
127.0
126.9
126.7
123.9
123.7
85.1
84.8
77.4
77.2
76.9
75.7
75.3
74.9
74.1
54.9
52.7
21.5
$CDCl_3$
200
190
180
170
160
150
140
130
120
110
100
90
80
70
60
50
40
30
20
10
0

^{1}H NMR, 500 MHz, $CDCl_3$

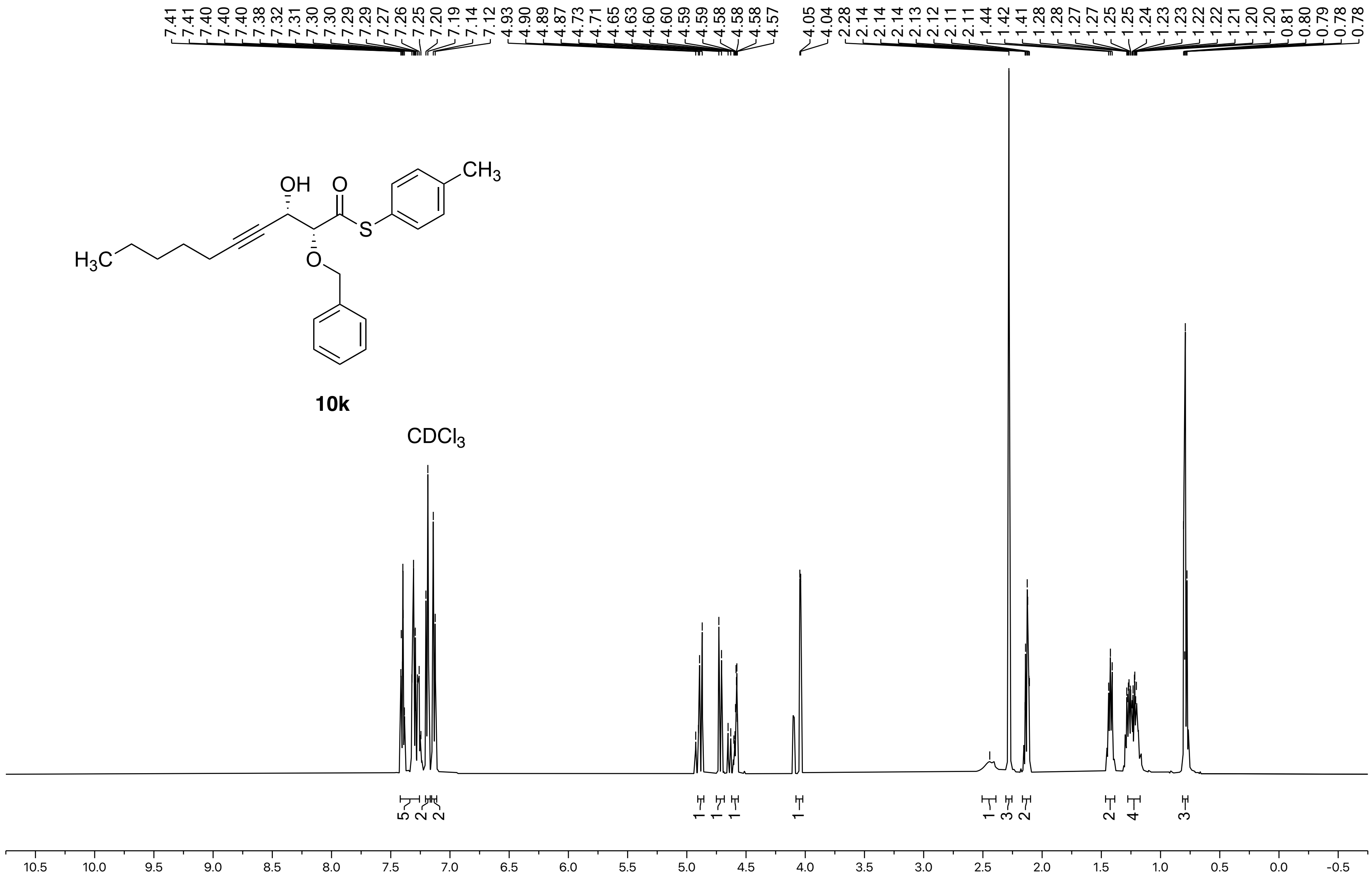

$^{13}C\{^{1}H\}$ NMR, 126 MHz, $CDCl_3$

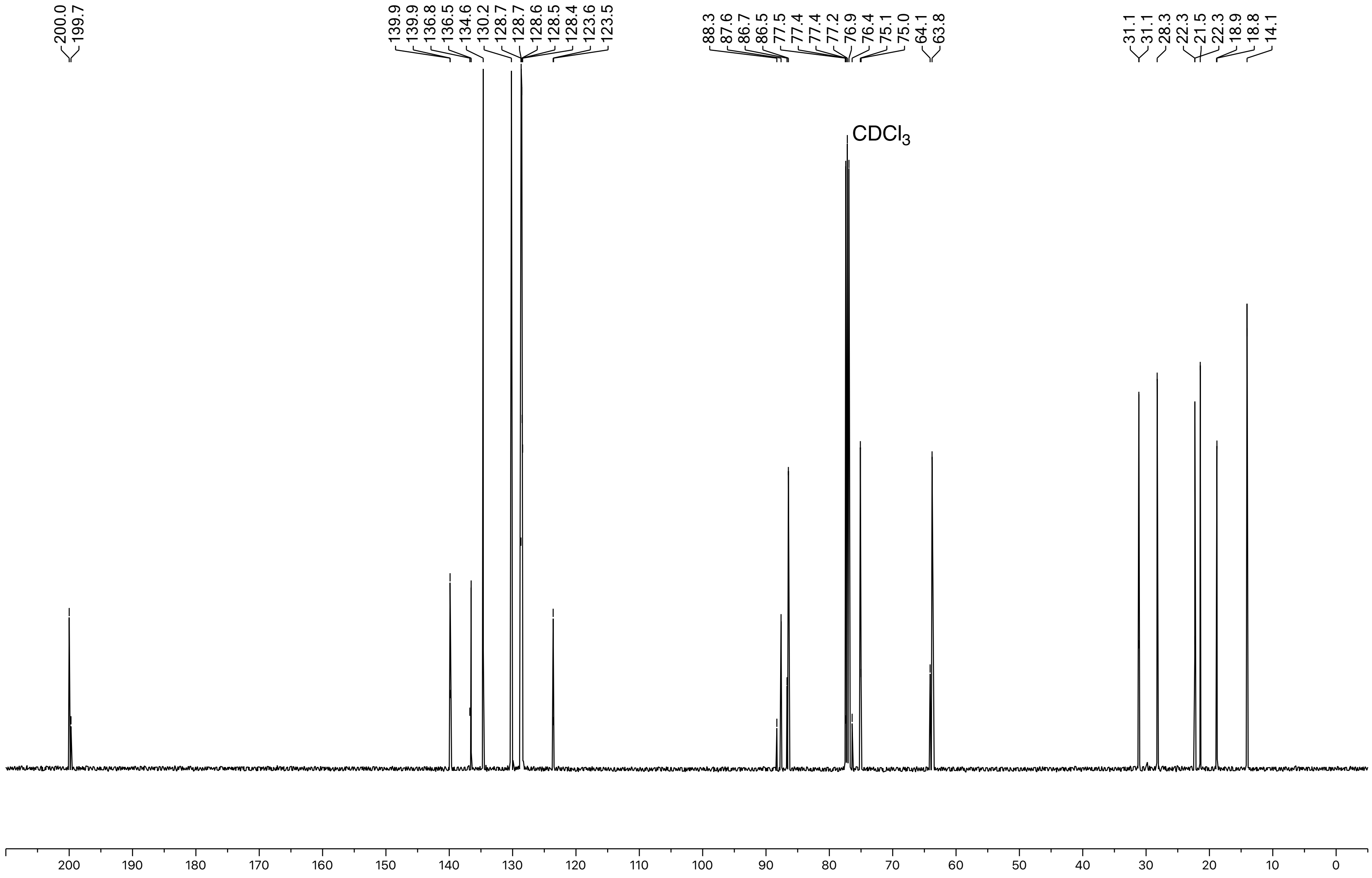

^{1}H NMR, 500 MHz, $CDCl_3$

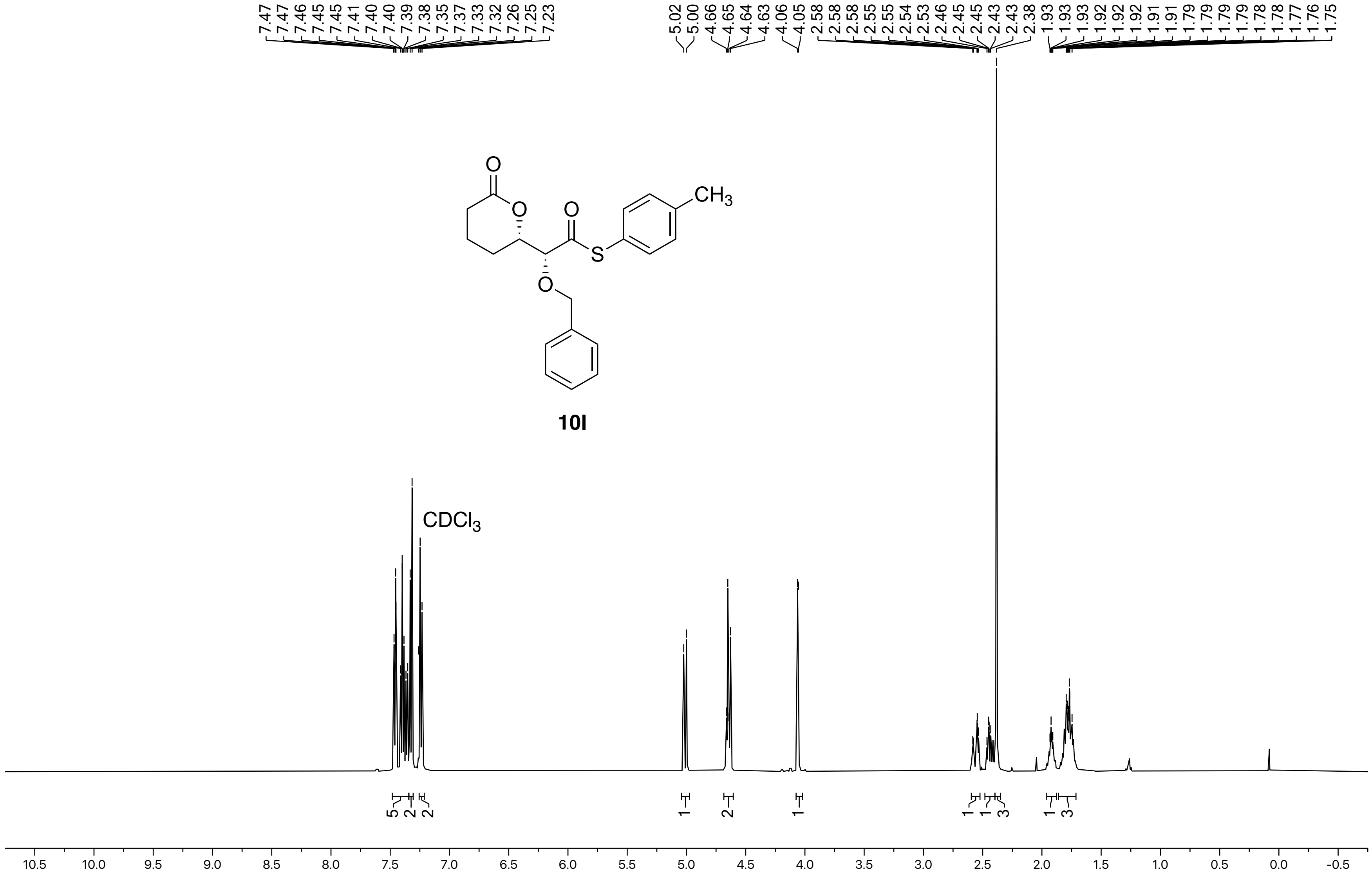

$^{13}C\{^{1}H\}$ NMR, 126 MHz, $CDCl_3$

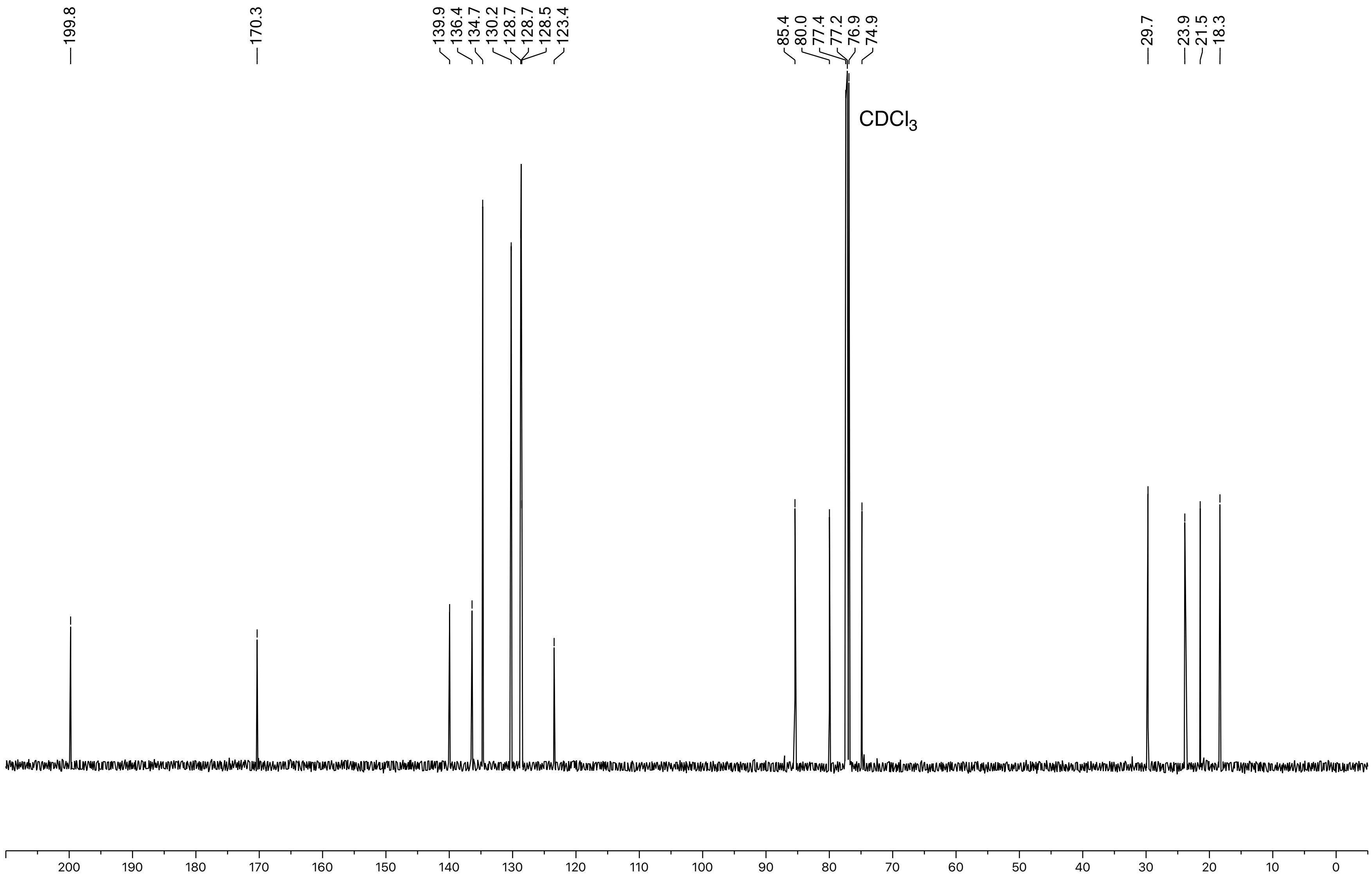

^{1}H NMR, 500 MHz, $CDCl_3$

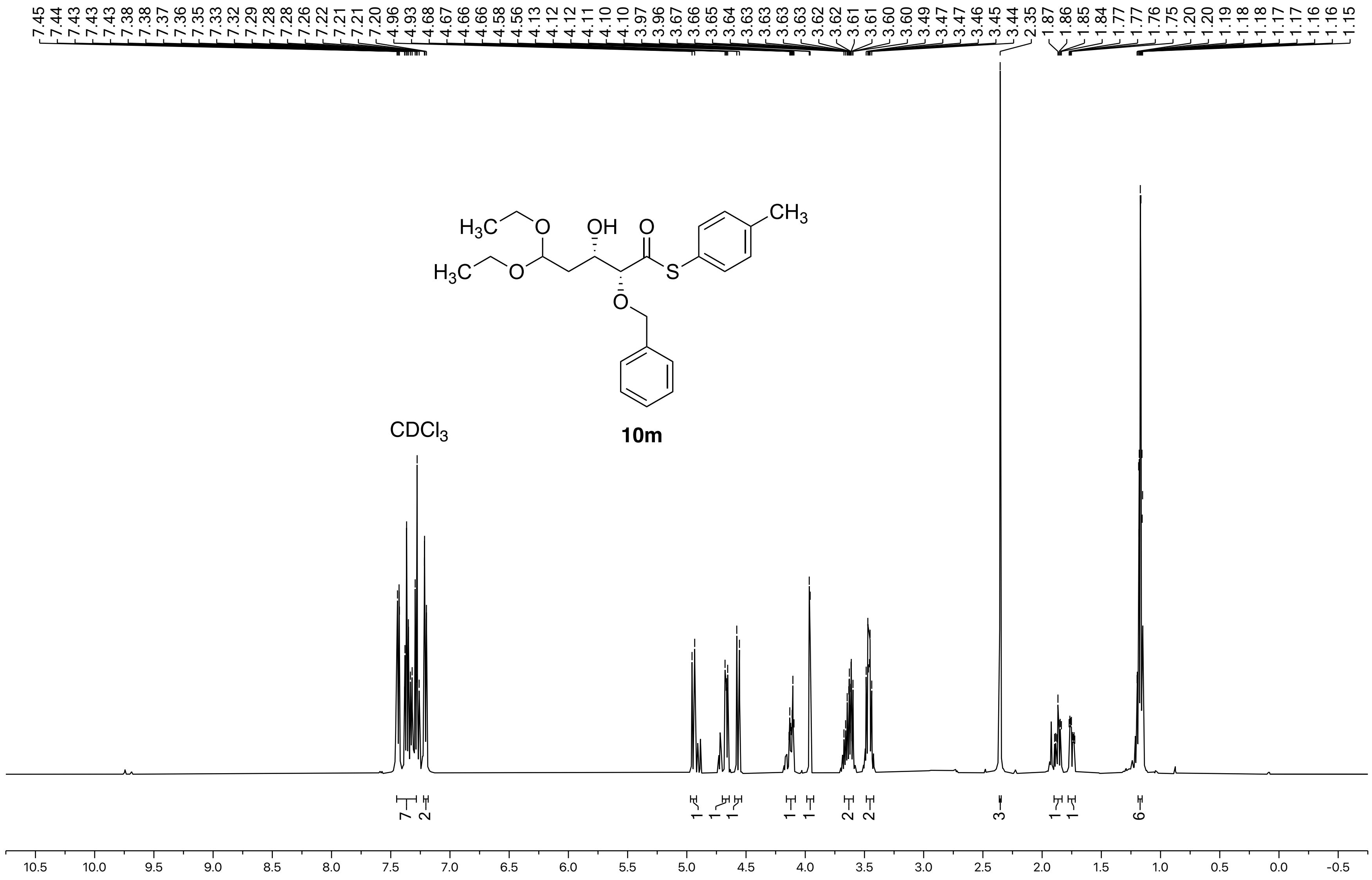

10m

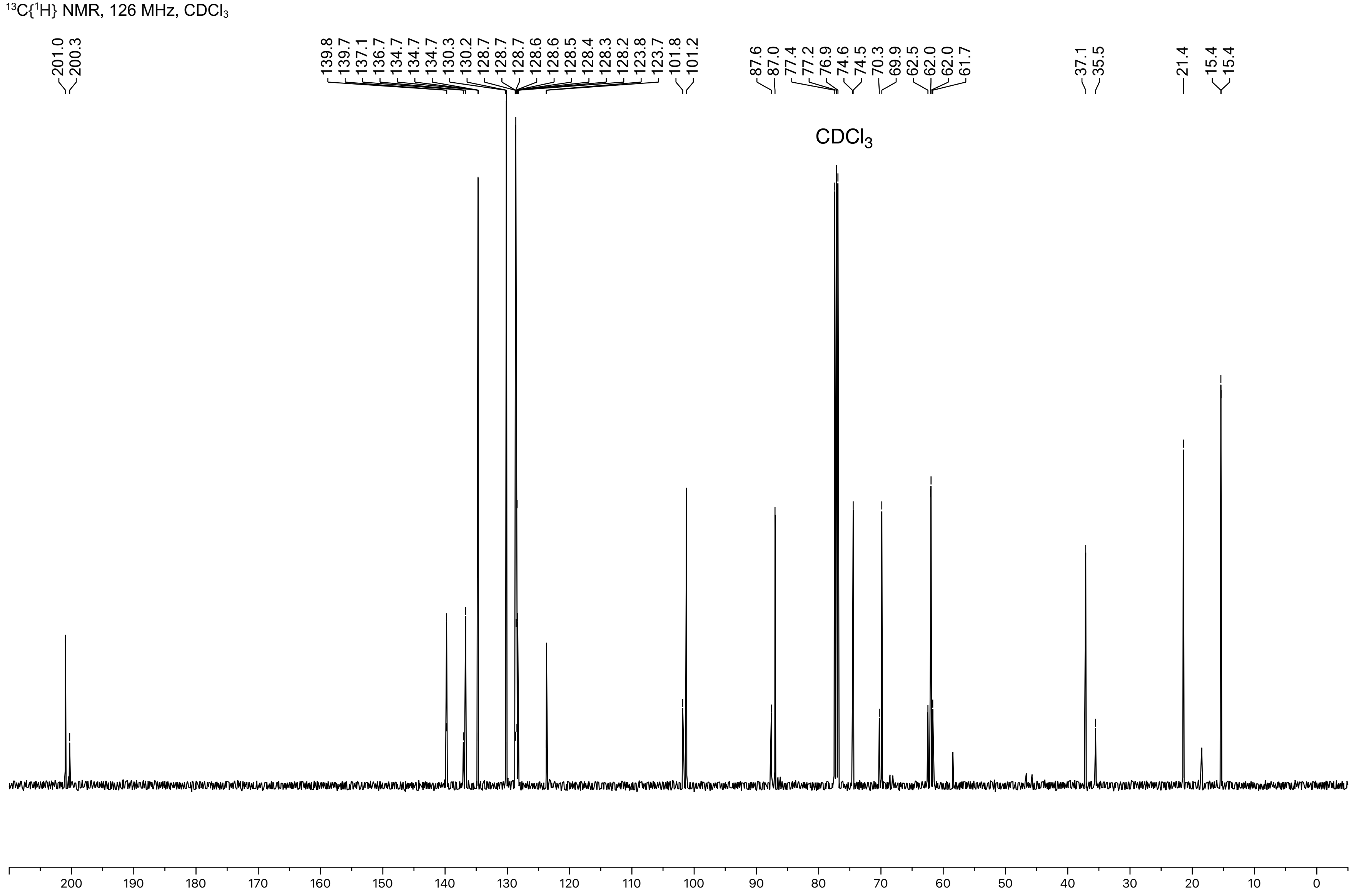

$^{13}C\{^1H\}$ NMR, 126 MHz, $CDCl_3$
201.0
200.3
139.8
139.7
137.1
136.7
134.7
134.7
134.7
130.3
130.2
128.7
128.7
128.7
128.6
128.6
128.5
128.4
128.3
128.2
123.8
123.7
101.8
101.2
87.6
87.0
77.4
77.2
76.9
74.6
74.5
70.3
69.9
62.5
62.0
62.0
61.7
37.1
35.5
21.4
15.4
15.4
$CDCl_3$
200
190
180
170
160
150
140
130
120
110
100
90
80
70
60
50
40
30
20
10
0

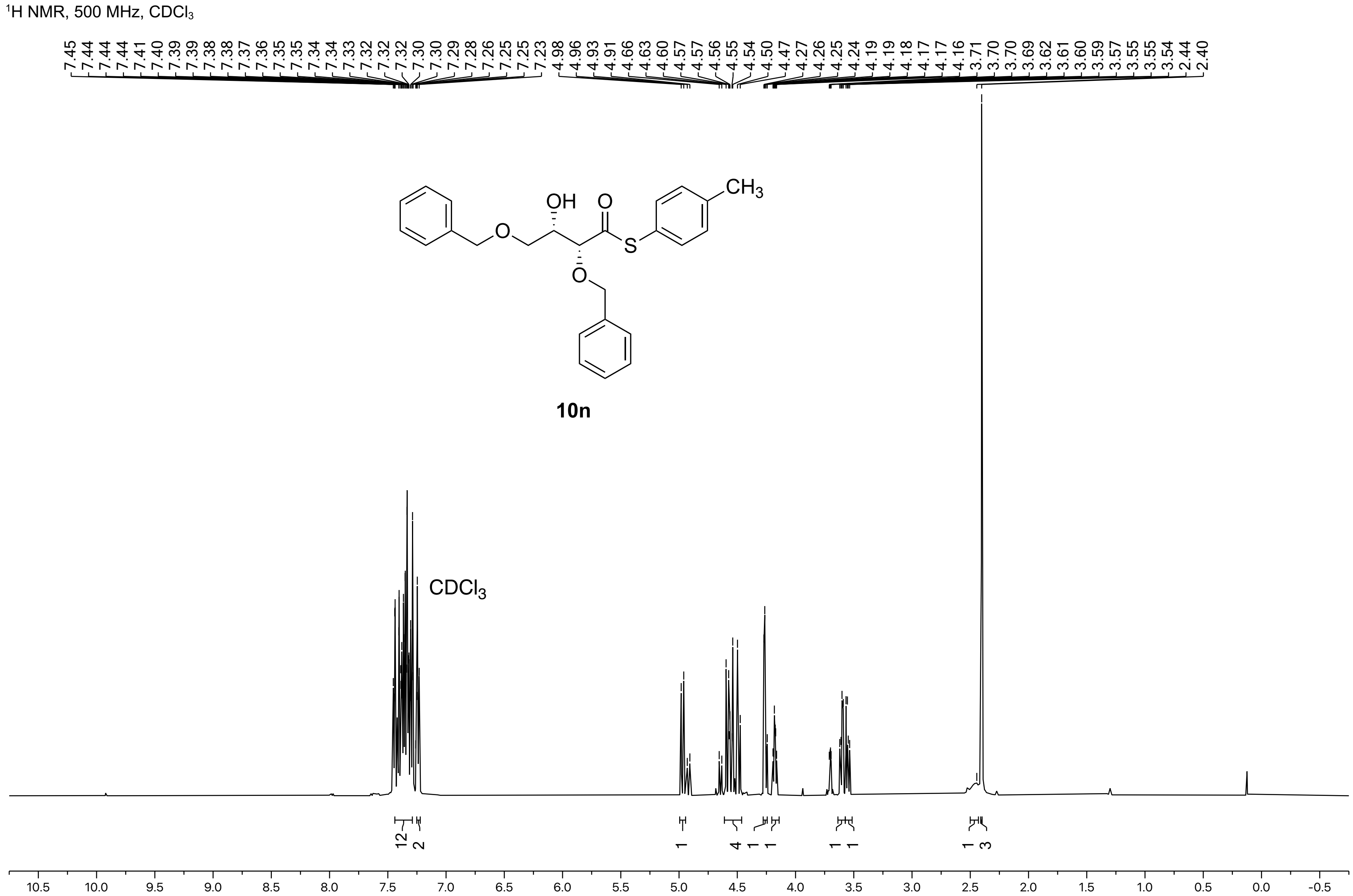

¹H NMR, 500 MHz, CDCl₃
7.45 7.44 7.44 7.44 7.41 7.40 7.39 7.39 7.38 7.38 7.37 7.36 7.35 7.35 7.34 7.34 7.33 7.32 7.32 7.32 7.30 7.30 7.29 7.28 7.26 7.25 7.25 7.23 4.98 4.96 4.93 4.91 4.66 4.63 4.60 4.57 4.57 4.56 4.55 4.55 4.54 4.50 4.47 4.27 4.26 4.25 4.24 4.19 4.19 4.18 4.17 4.17 4.16 3.71 3.70 3.70 3.69 3.62 3.61 3.60 3.59 3.57 3.55 3.55 3.54 2.44 2.40
OH
O
CH3
O
S
O
10n
CDCl3
12
2
1
4
1
1
1
1
1
3
10.5 10.0 9.5 9.0 8.5 8.0 7.5 7.0 6.5 6.0 5.5 5.0 4.5 4.0 3.5 3.0 2.5 2.0 1.5 1.0 0.5 0.0 -0.5

$^{13}C\{^1H\}$ NMR, 126 MHz, $CDCl_3$

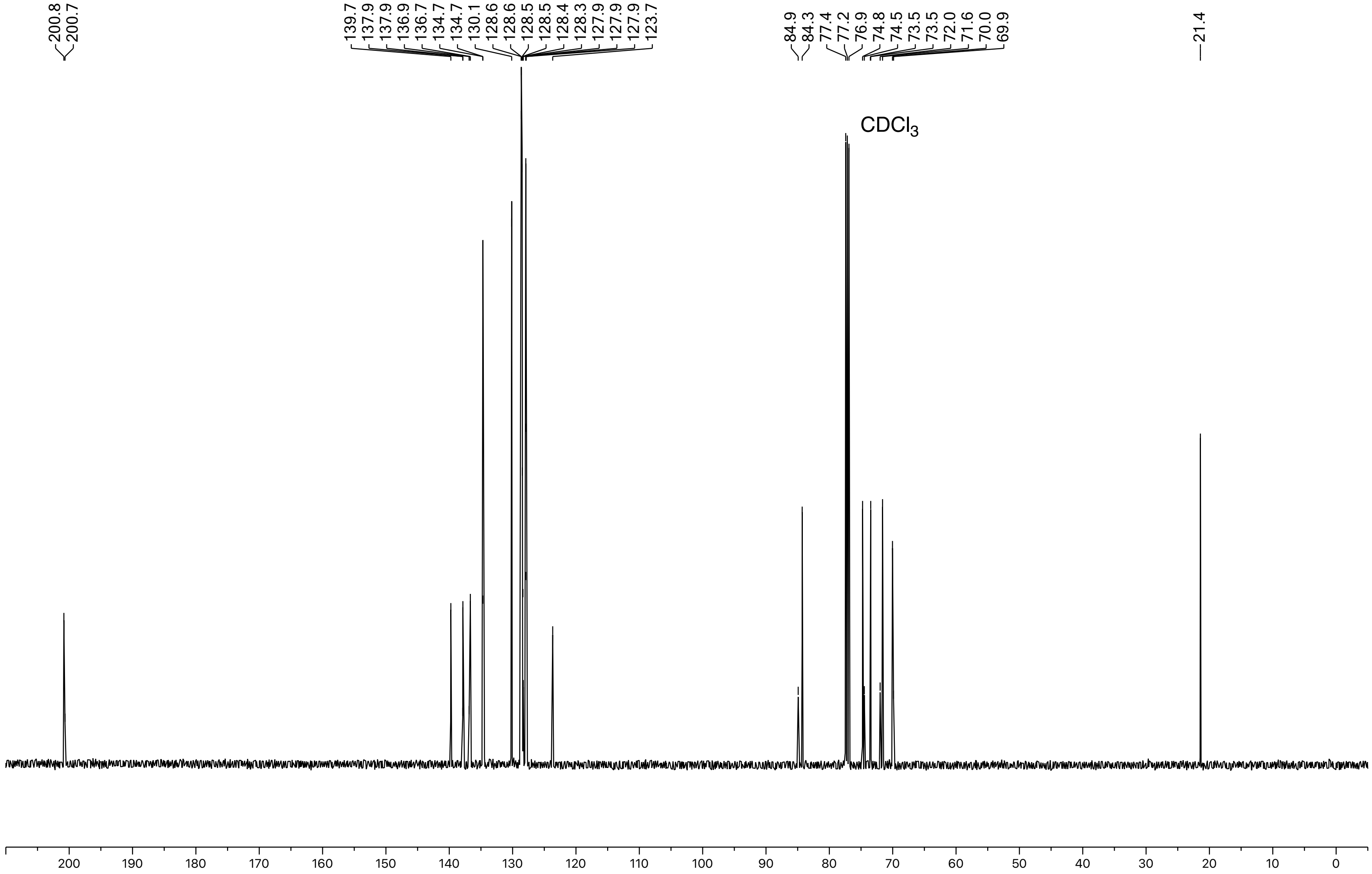

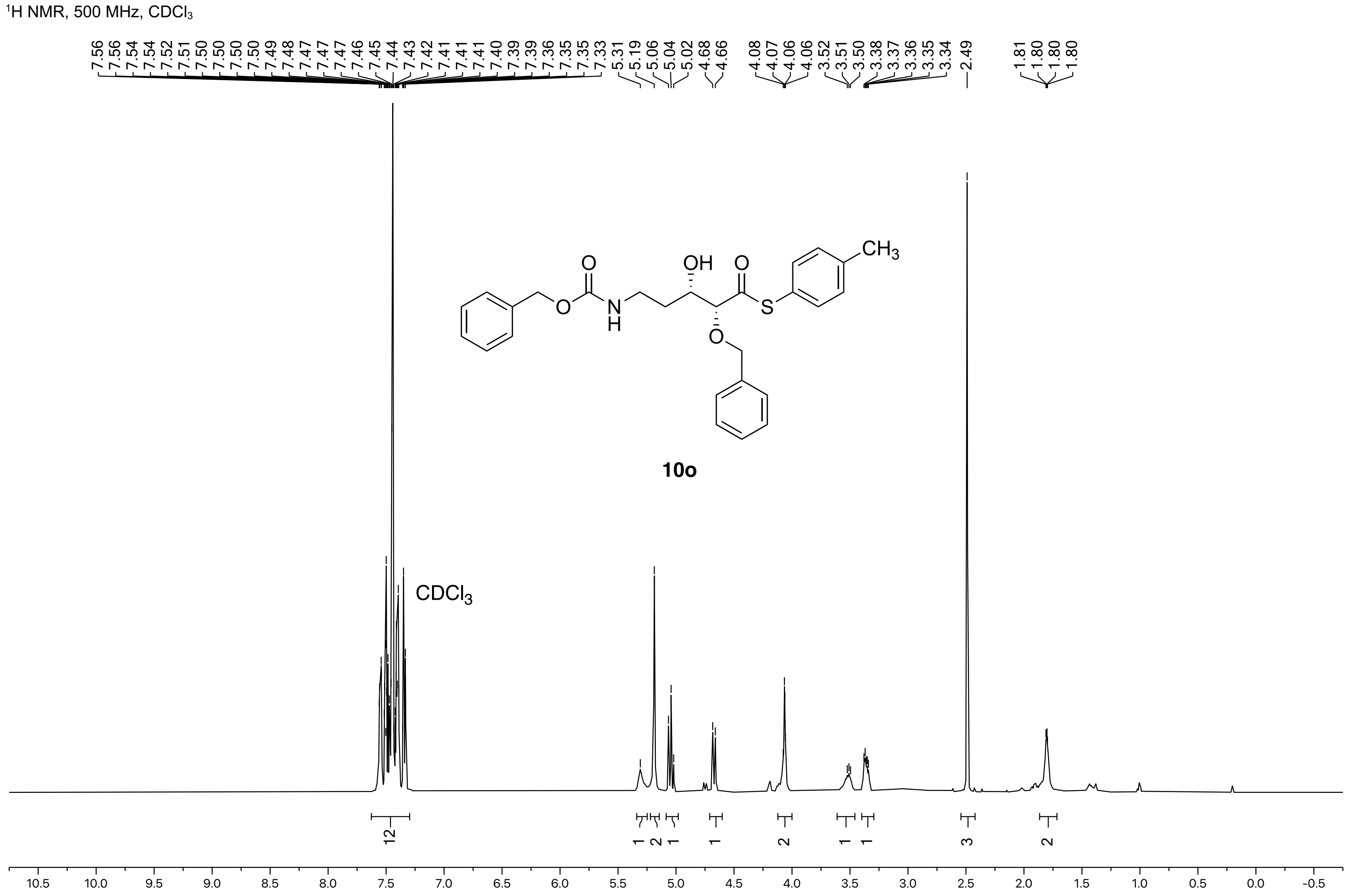

^{1}H NMR, 500 MHz, CDCl3
CDCl3
10o

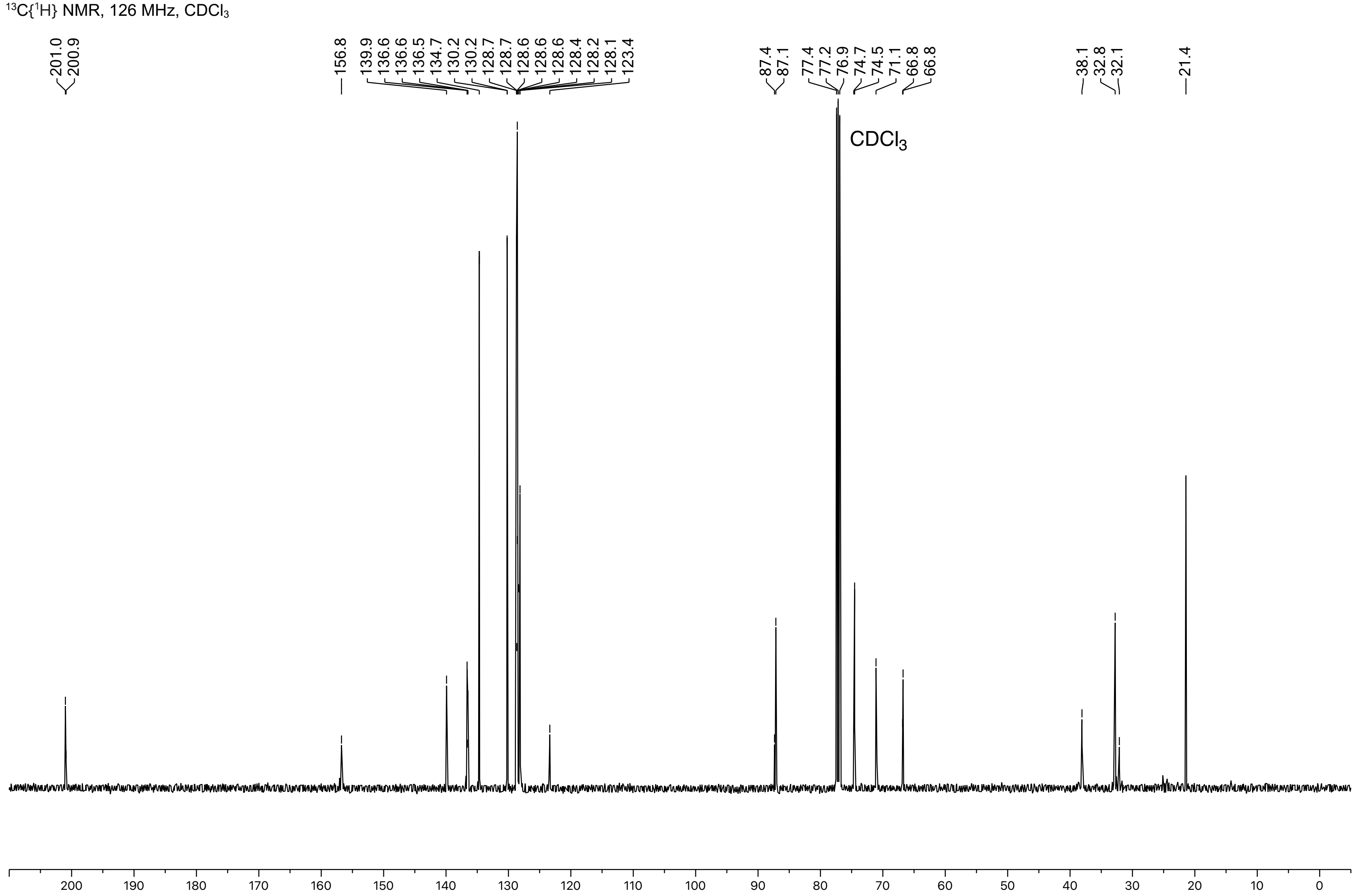
$^{13}C\{^1H\}$ NMR, 126 MHz, $CDCl_3$
201.0
200.9
156.8
139.9
136.6
136.6
136.5
134.7
130.2
130.2
128.7
128.7
128.6
128.6
128.6
128.4
128.2
128.1
123.4
87.4
87.1
77.4
77.2
76.9
74.7
74.5
71.1
66.8
66.8
38.1
32.8
32.1
21.4
$CDCl_3$
200 190 180 170 160 150 140 130 120 110 100 90 80 70 60 50 40 30 20 10 0

^1H NMR, 500 MHz, $CDCl_3$

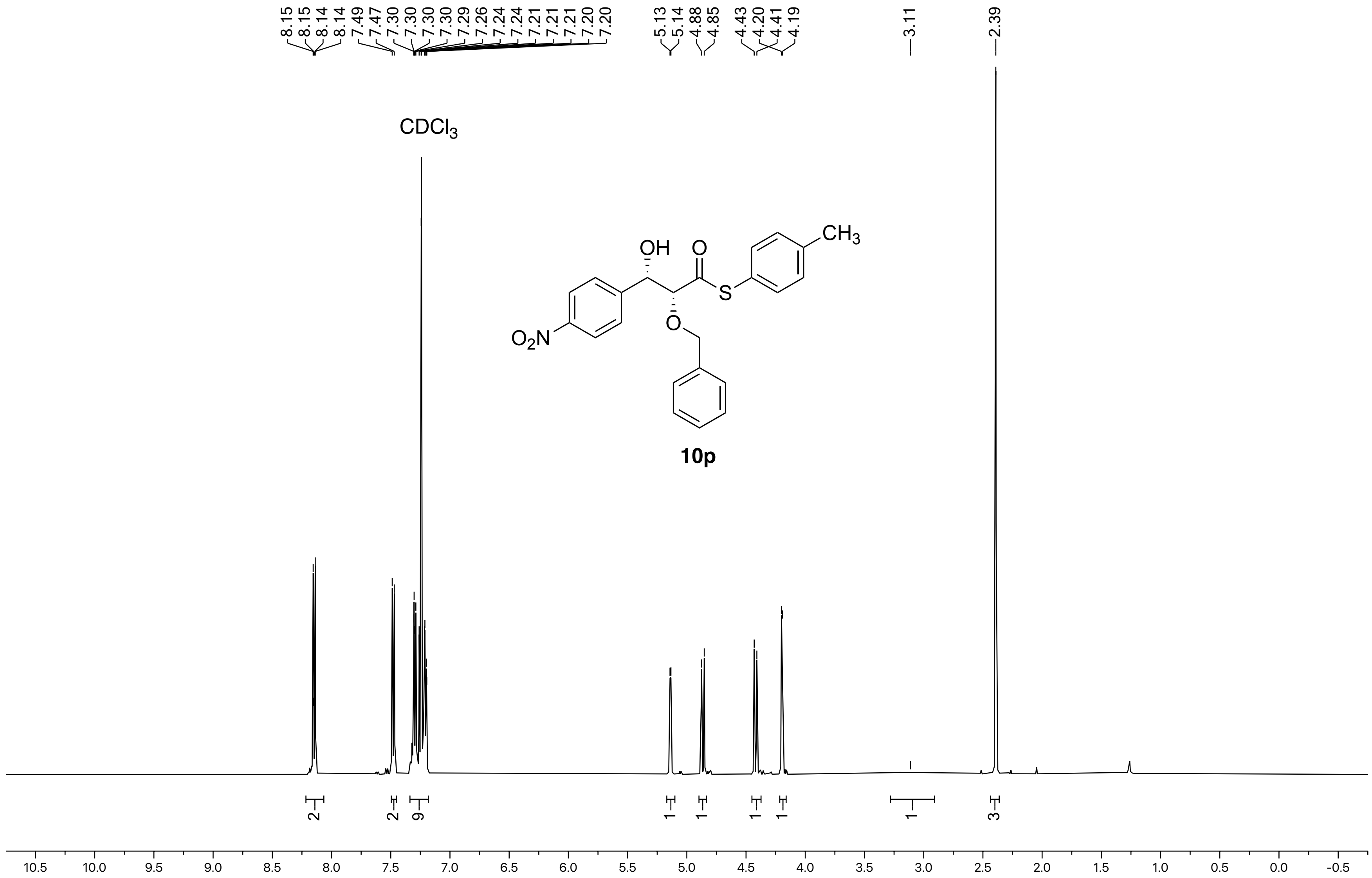

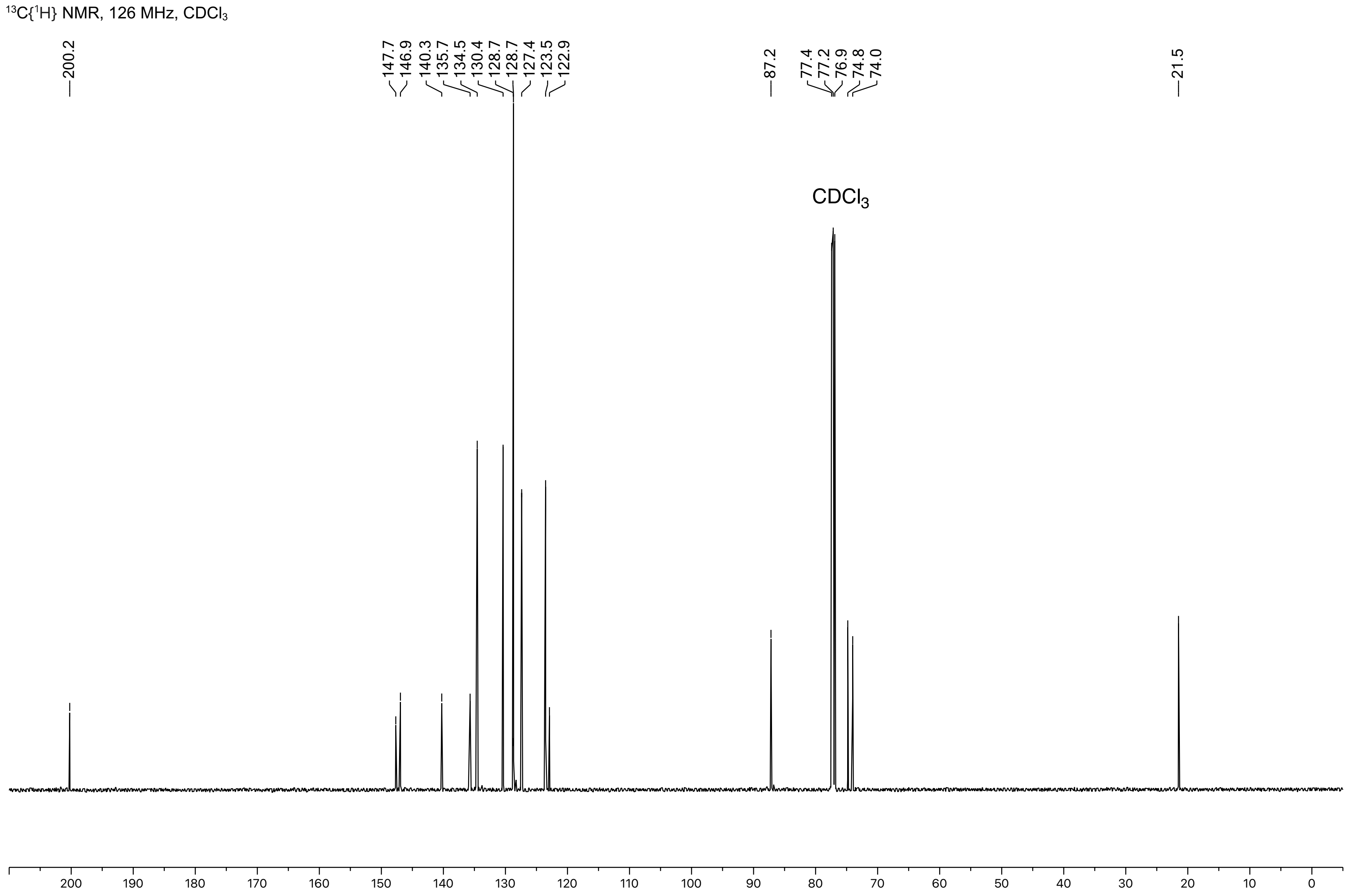

$^{13}C\{^1H\}$ NMR, 126 MHz, $CDCl_3$
200.2
147.7
146.9
140.3
135.7
134.5
130.4
128.7
128.7
127.4
123.5
122.9
87.2
77.4
77.2
76.9
74.8
74.0
21.5
$CDCl_3$
200 190 180 170 160 150 140 130 120 110 100 90 80 70 60 50 40 30 20 10 0

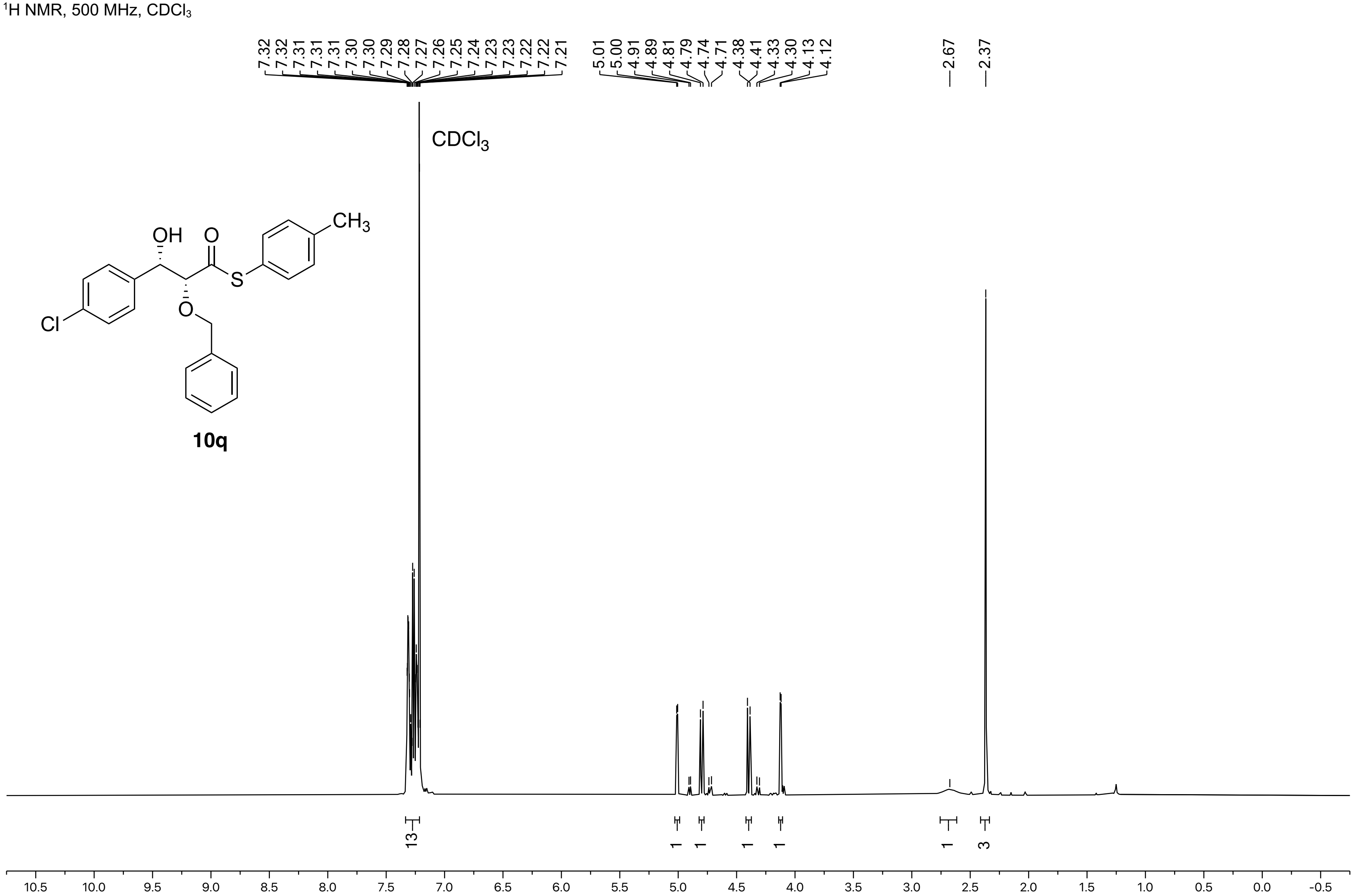

^{1}H NMR, 500 MHz, CDCl$_3$
7.32 7.32 7.31 7.31 7.31 7.30 7.29 7.28 7.27 7.26 7.25 7.24 7.23 7.23 7.22 7.22 7.21
5.01 5.00 4.91 4.89 4.81 4.79 4.74 4.71 4.38 4.41 4.33 4.30 4.13 4.12
2.67
2.37
CDCl$_3$
OH
O
S
CH$_3$
Cl
O
10q
13
1
1
1
1
1
3
10.5 10.0 9.5 9.0 8.5 8.0 7.5 7.0 6.5 6.0 5.5 5.0 4.5 4.0 3.5 3.0 2.5 2.0 1.5 1.0 0.5 0.0 -0.5

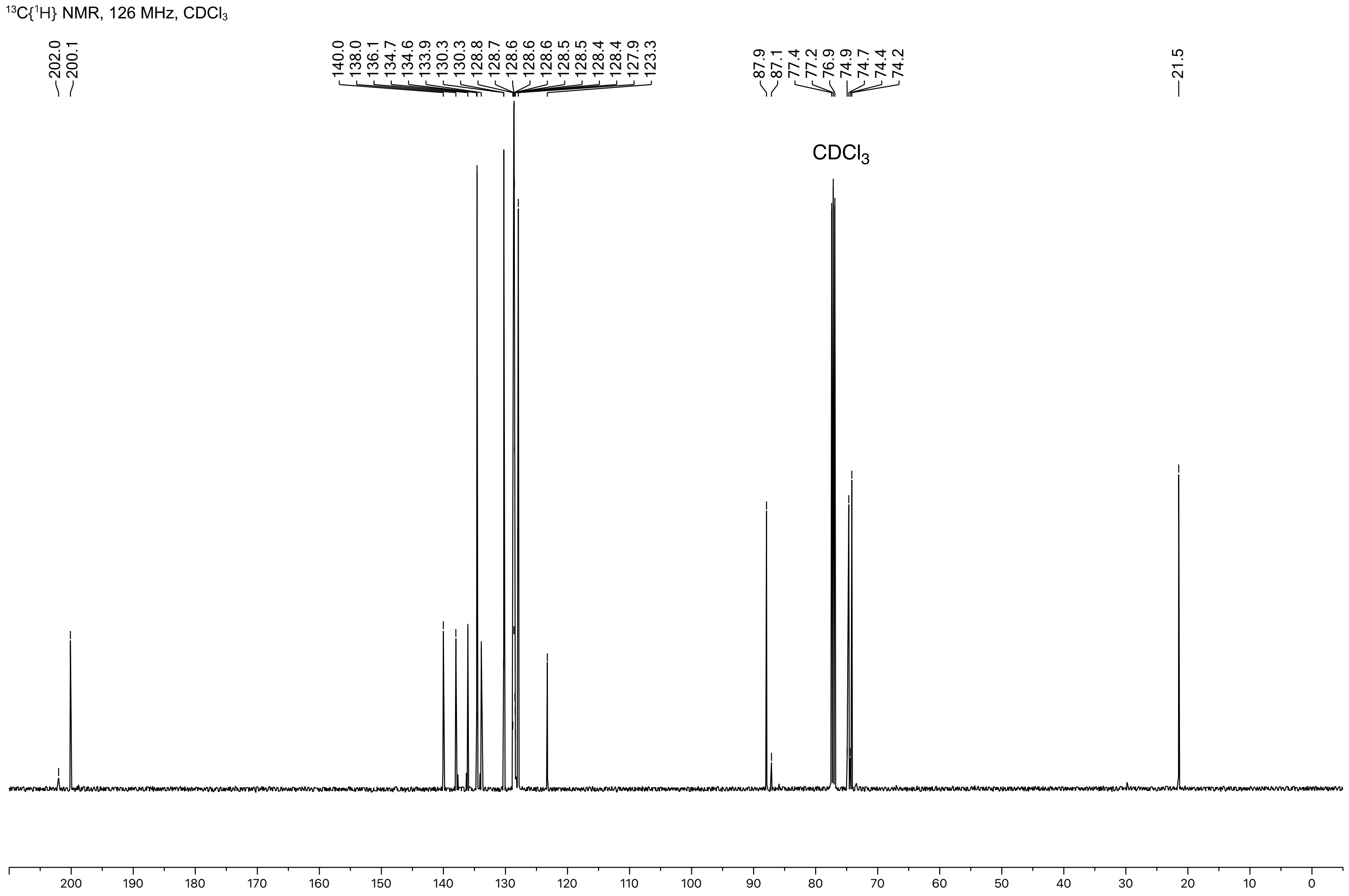
$^{13}C\{^1H\}$ NMR, 126 MHz, $CDCl_3$
202.0
200.1
140.0
138.0
136.1
134.7
134.6
133.9
130.3
130.3
128.8
128.7
128.6
128.6
128.6
128.5
128.5
128.4
128.4
127.9
123.3
87.9
87.1
77.4
77.2
76.9
74.9
74.7
74.4
74.2
21.5
$CDCl_3$
200
190
180
170
160
150
140
130
120
110
100
90
80
70
60
50
40
30
20
10
0

^1H NMR, 500 MHz, $CDCl_3$

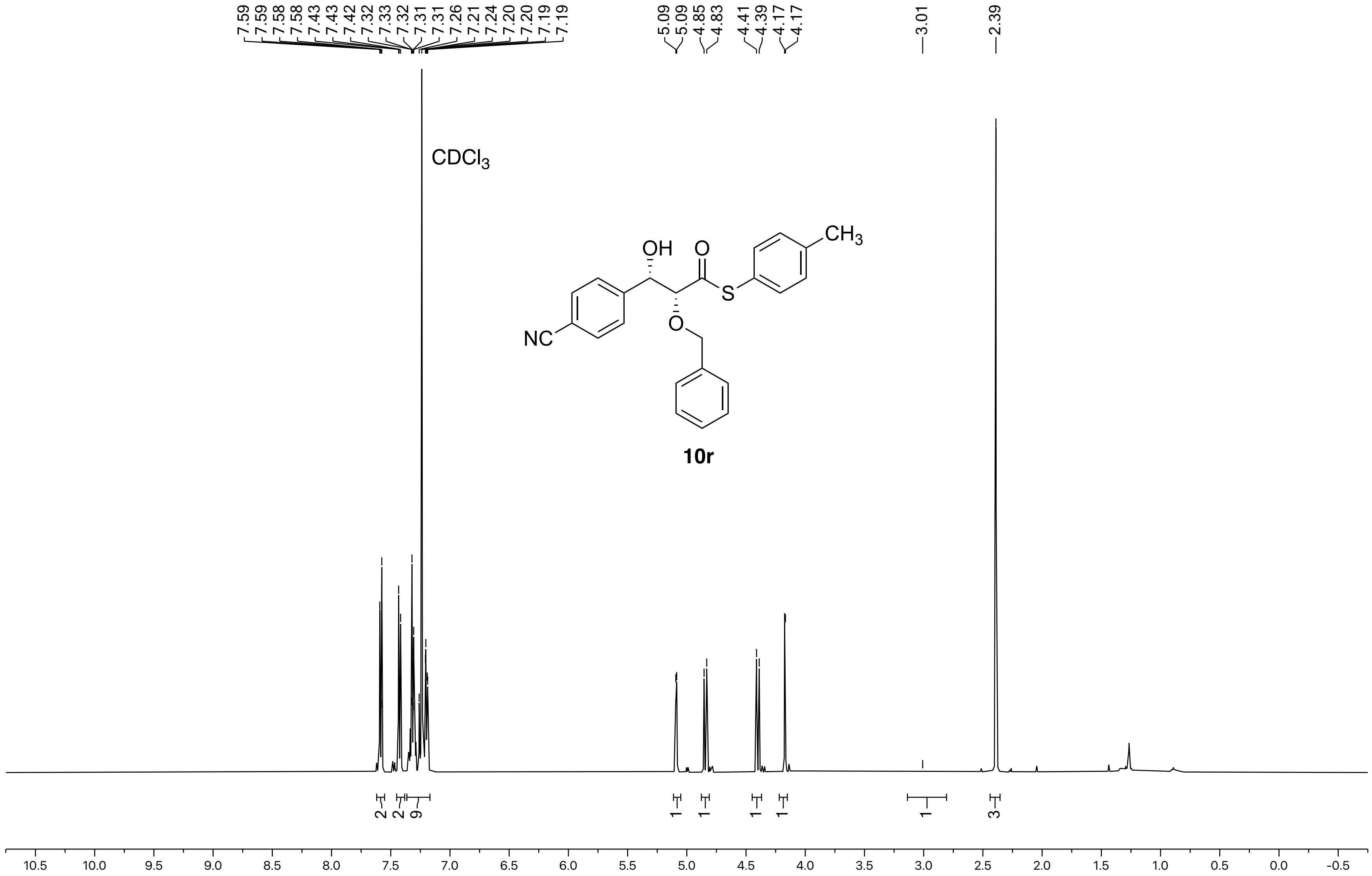

$^{13}C\{^{1}H\}$ NMR, 126 MHz, $CDCl_3$

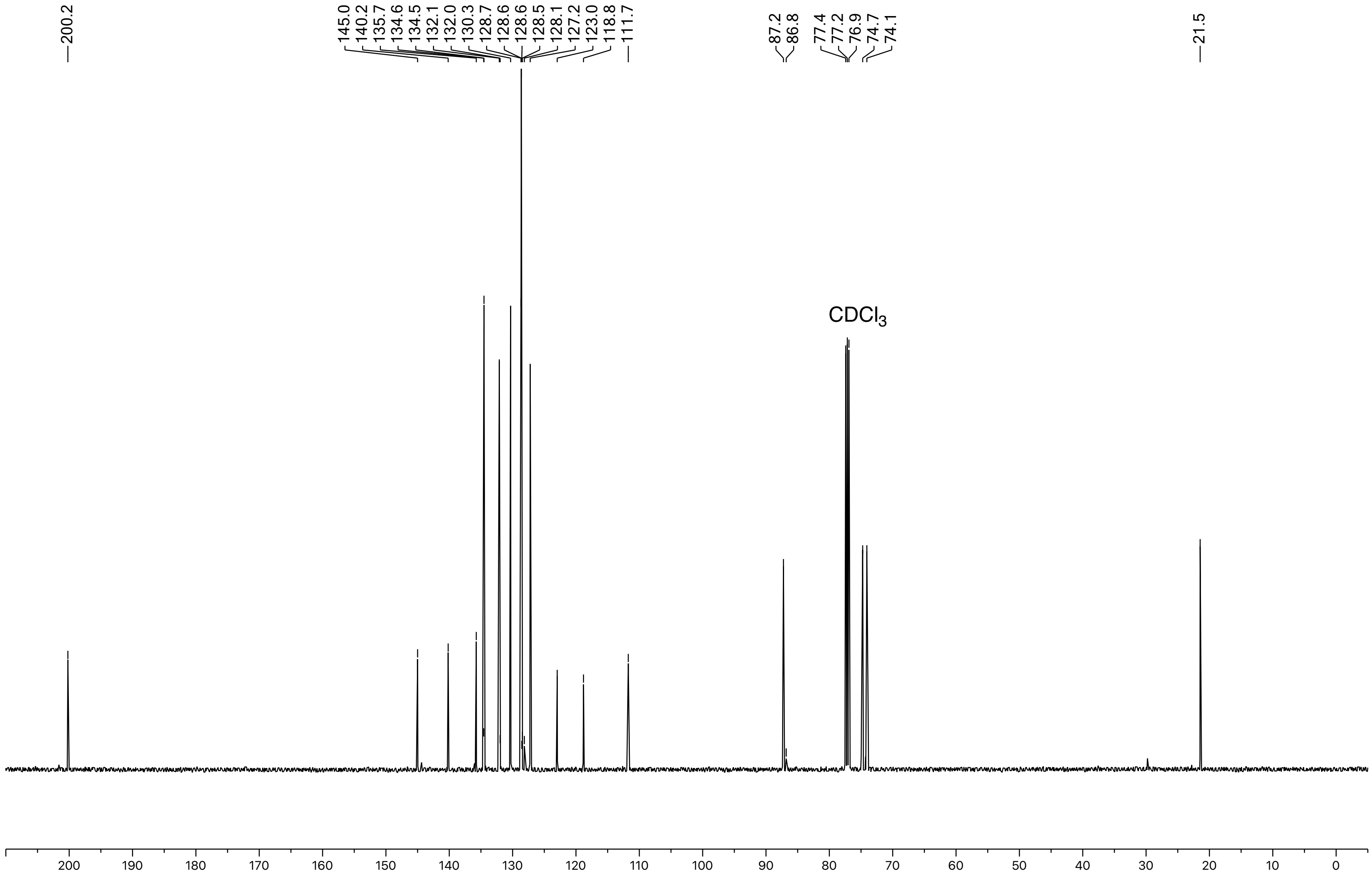

^1H NMR, 500 MHz, $CDCl_3$

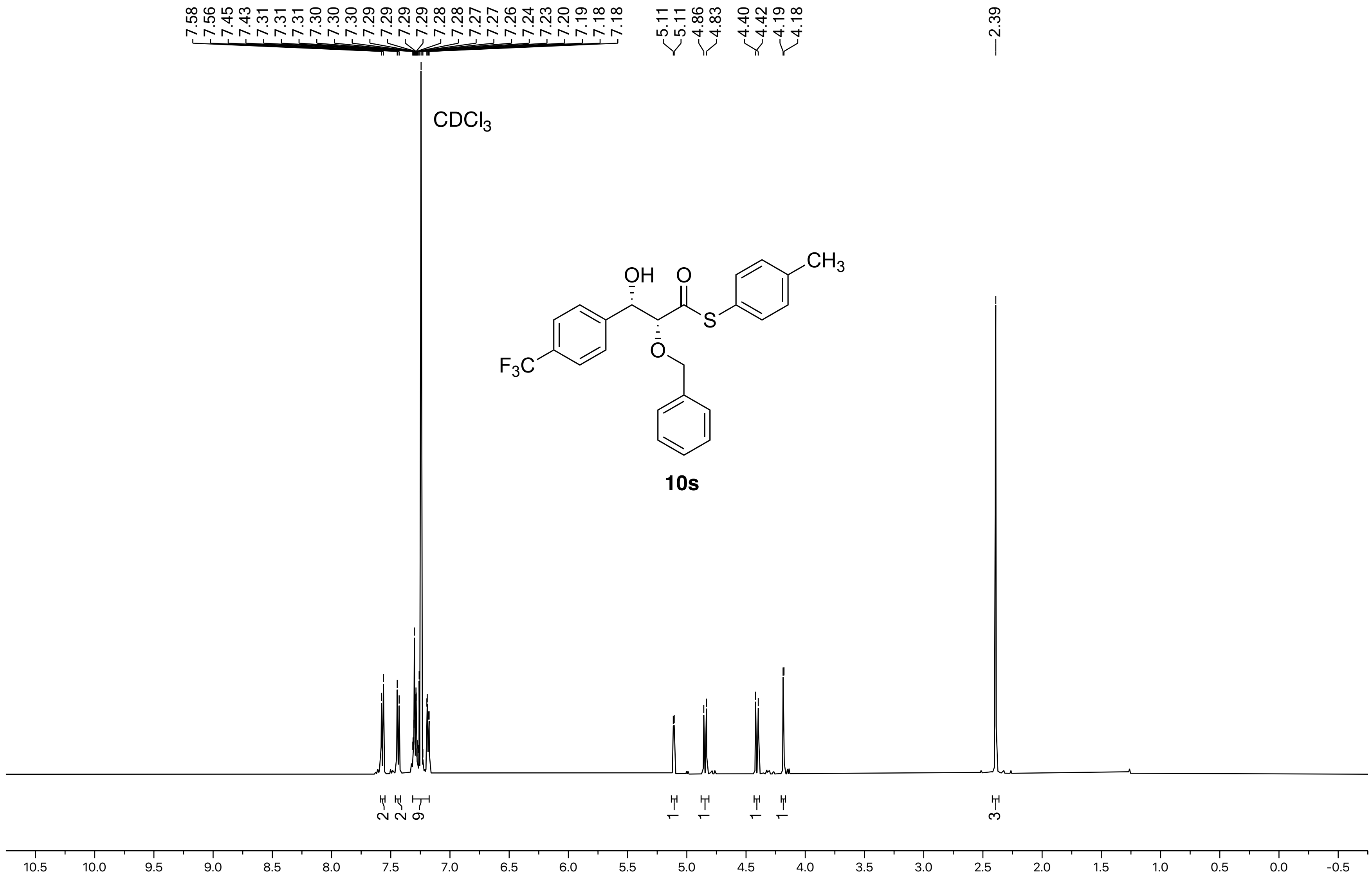

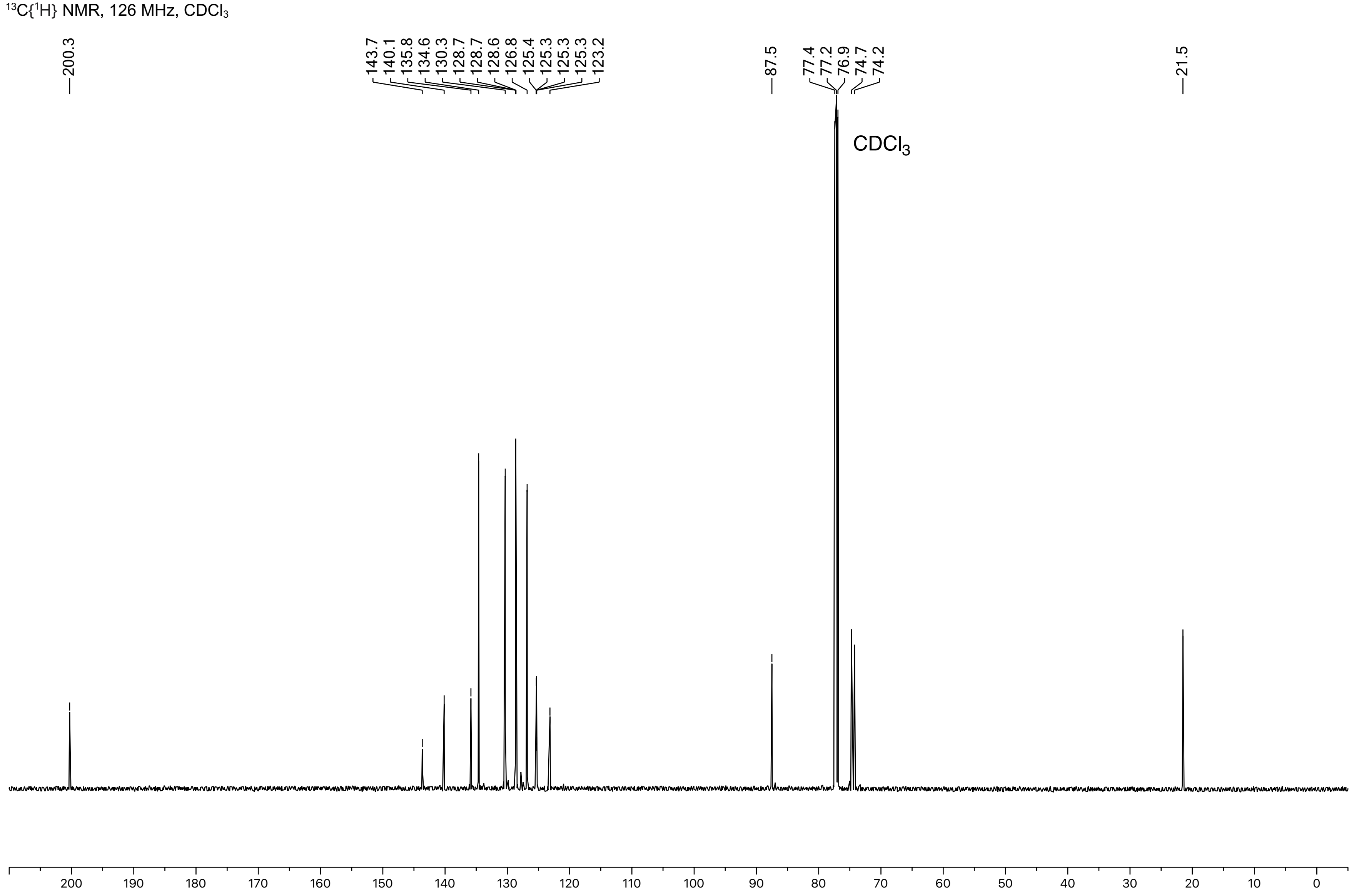

$^{13}C\{^{1}H\}$ NMR, 126 MHz, $CDCl_3$
200.3
143.7
140.1
135.8
134.6
130.3
128.7
128.7
128.6
126.8
125.4
125.3
125.3
125.3
123.2
87.5
77.4
77.2
76.9
74.7
74.2
21.5
$CDCl_3$
200 190 180 170 160 150 140 130 120 110 100 90 80 70 60 50 40 30 20 10 0

^{19}F NMR, 470 MHz, $CDCl_3$

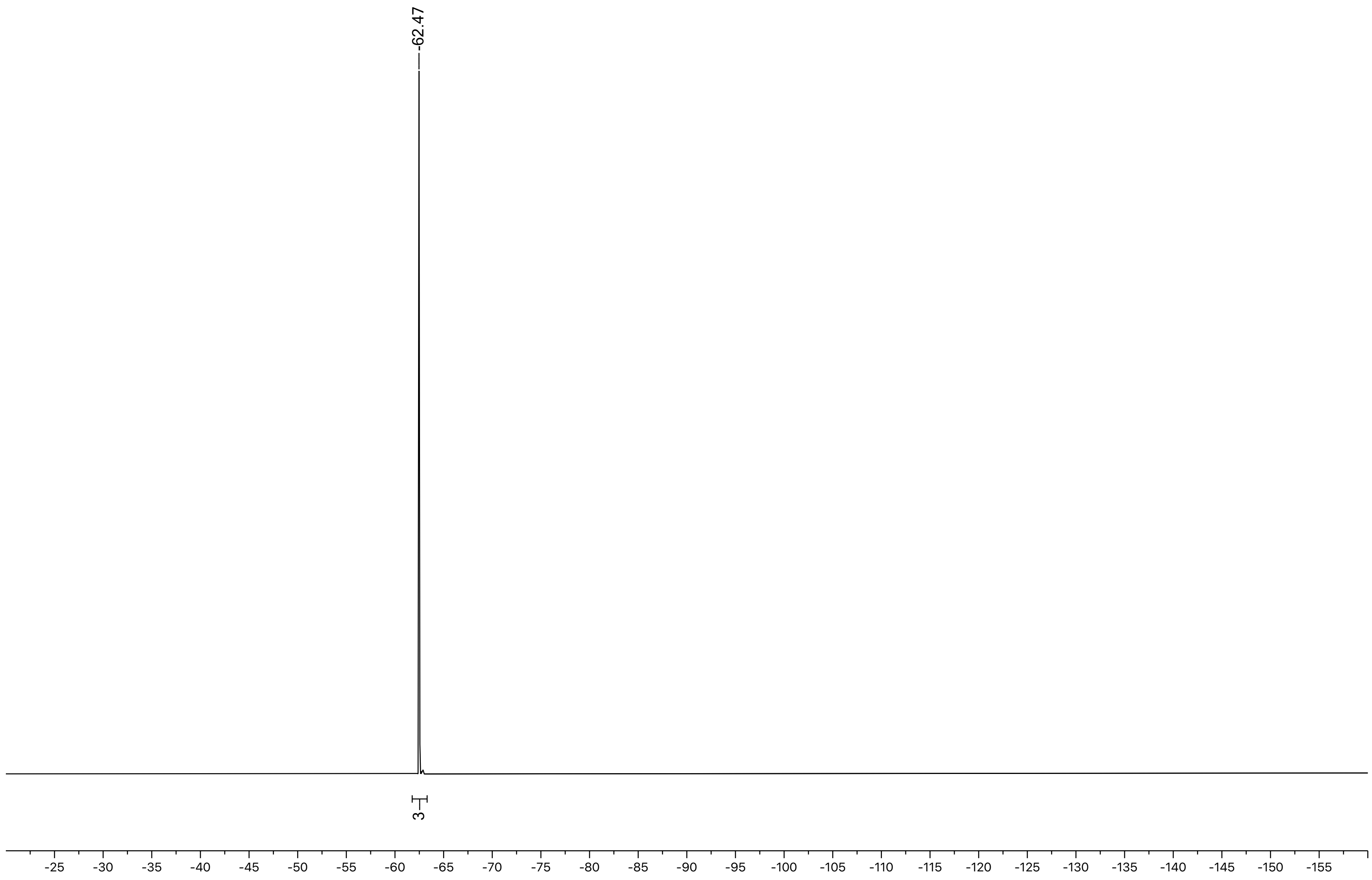

^{1}H NMR, 500 MHz, $CDCl_3$

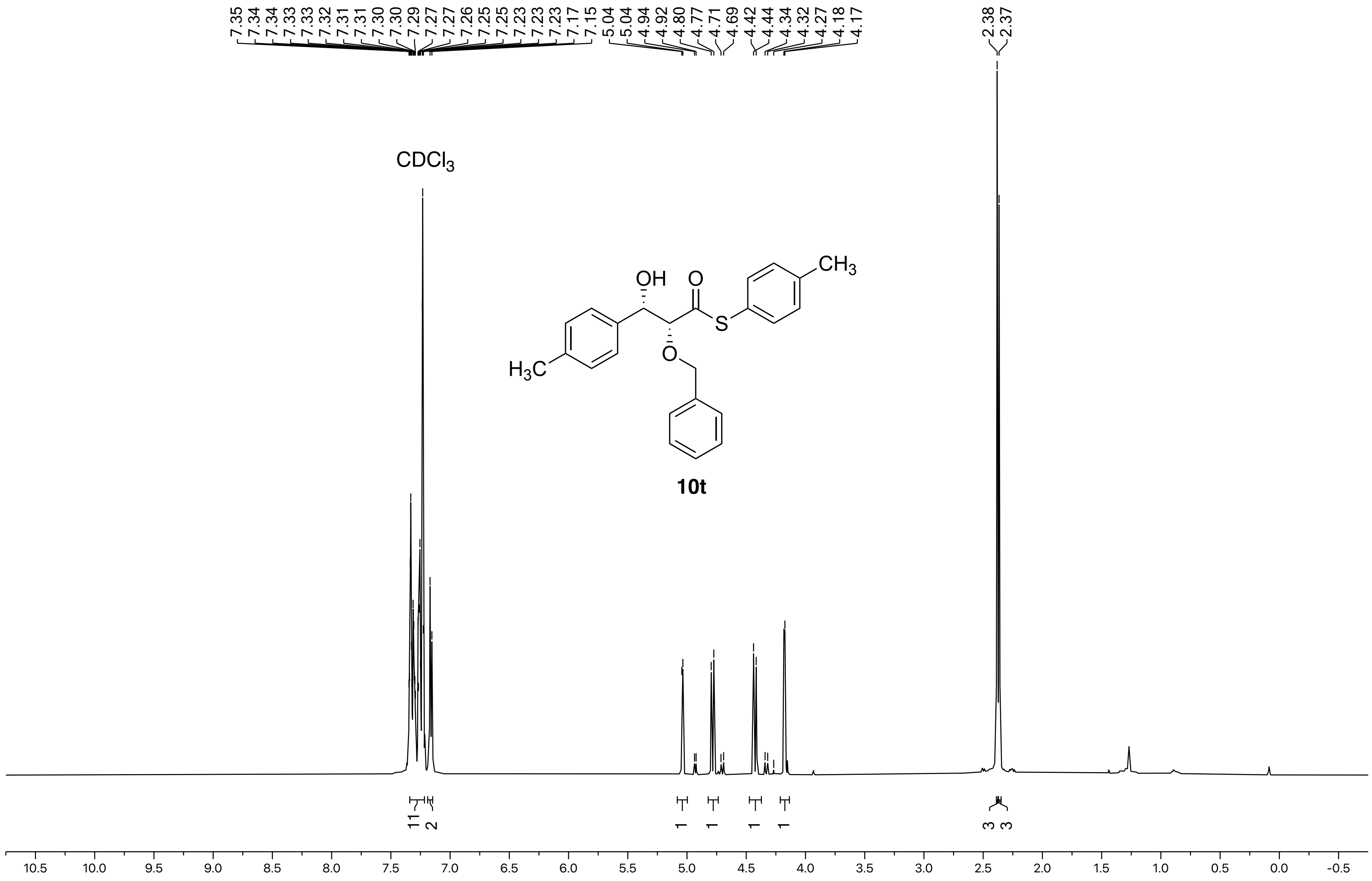

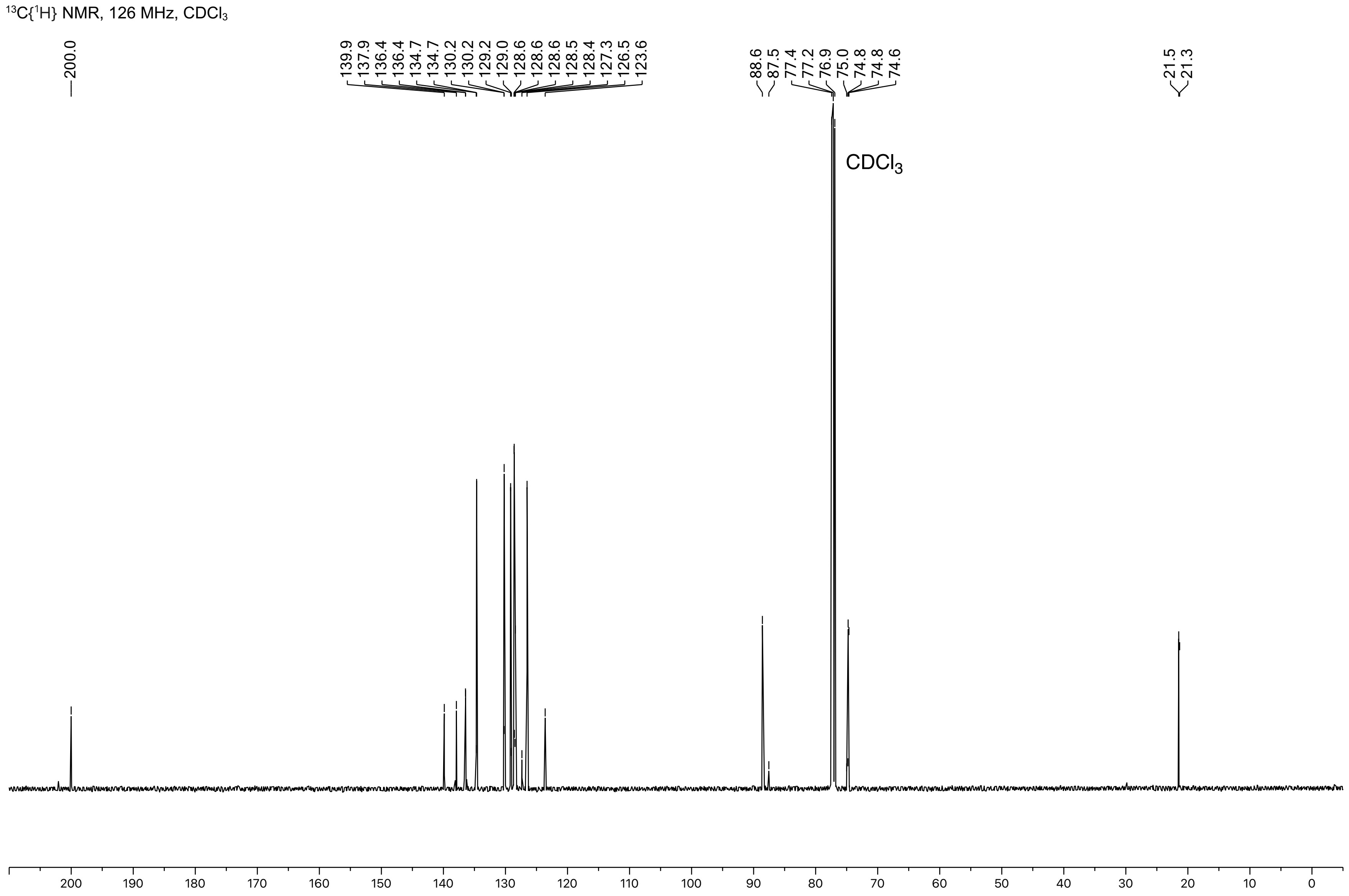

$^{13}C\{^1H\}$ NMR, 126 MHz, $CDCl_3$
200.0
139.9
137.9
136.4
136.4
134.7
130.2
130.2
129.2
129.0
128.6
128.6
128.6
128.5
128.4
127.3
126.5
123.6
88.6
87.5
77.4
77.2
76.9
75.0
74.8
74.8
74.6
21.5
21.3
$CDCl_3$
200
190
180
170
160
150
140
130
120
110
100
90
80
70
60
50
40
30
20
10
0

^{1}H NMR, 500 MHz, $CDCl_3$

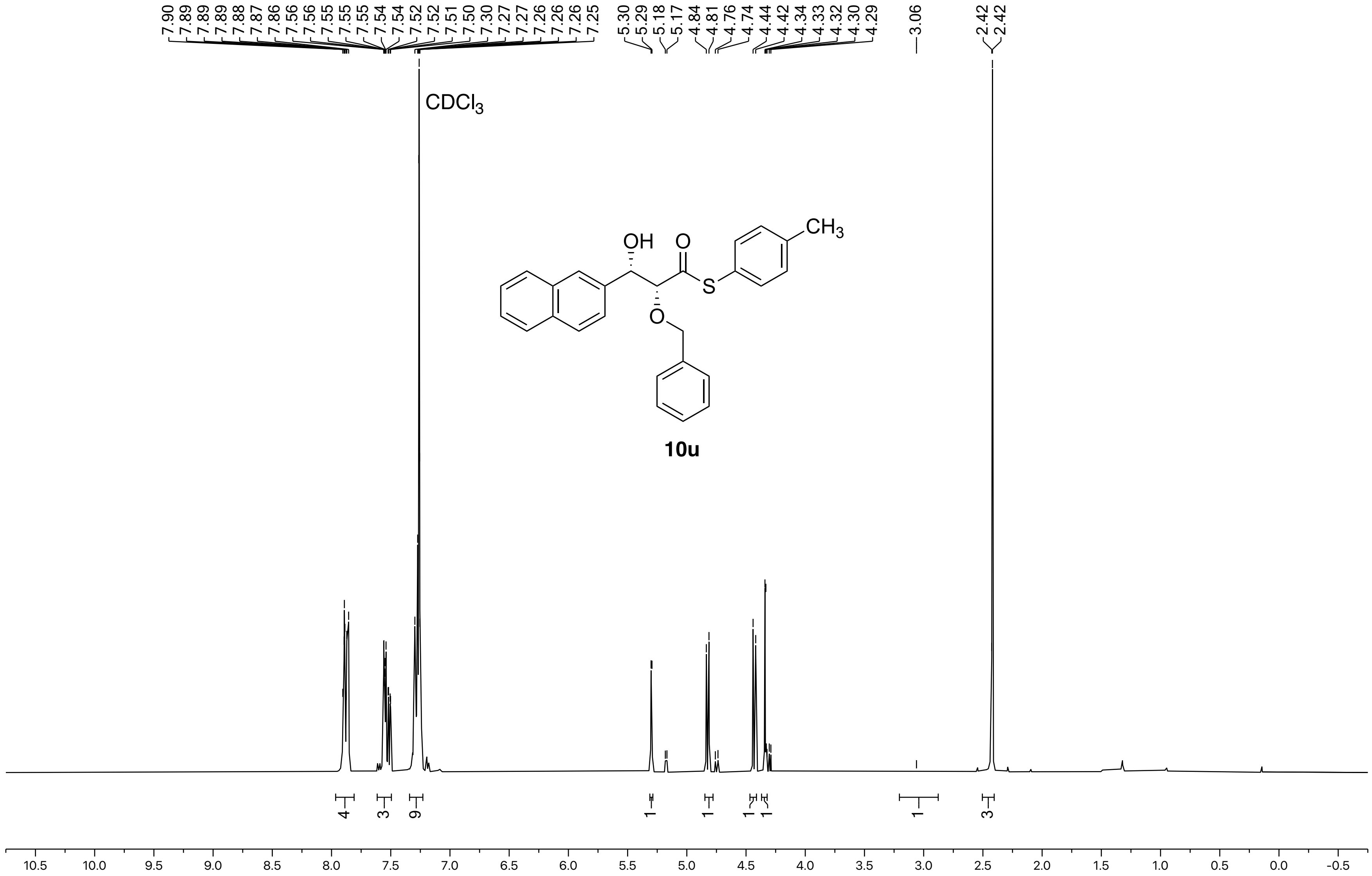

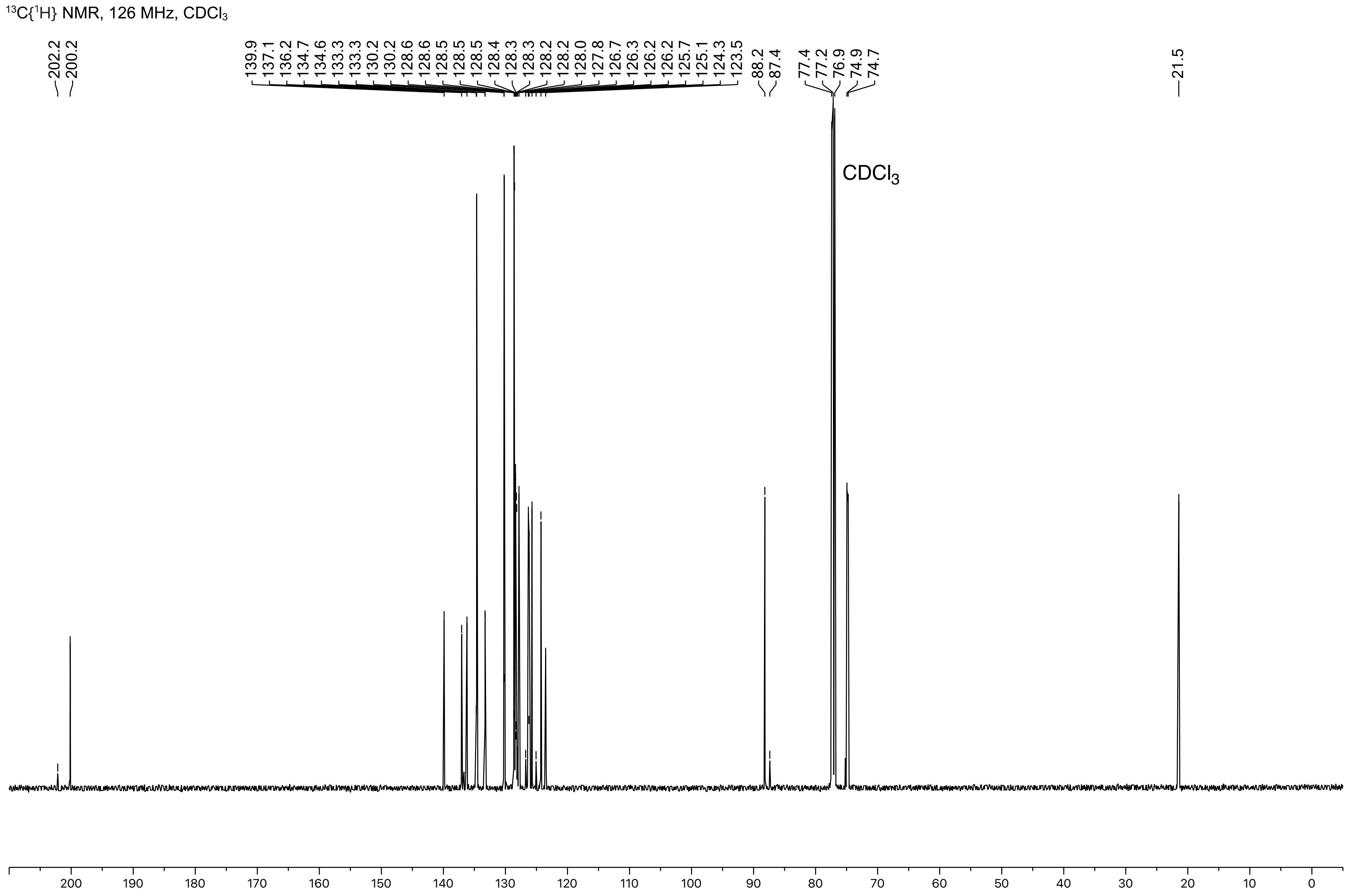
13C{1H} NMR, 126 MHz, CDCl3
202.2
200.2
139.9
137.1
136.2
134.7
134.6
133.3
133.3
130.2
130.2
128.6
128.6
128.5
128.5
128.5
128.4
128.3
128.3
128.3
128.2
128.2
128.0
127.8
126.7
126.3
126.2
126.2
125.7
125.1
124.3
123.5
88.2
87.4
77.4
77.2
76.9
74.9
74.7
21.5
CDCl3
200
190
180
170
160
150
140
130
120
110
100
90
80
70
60
50
40
30
20
10
0

^{1}H NMR, 500 MHz, $CDCl_3$

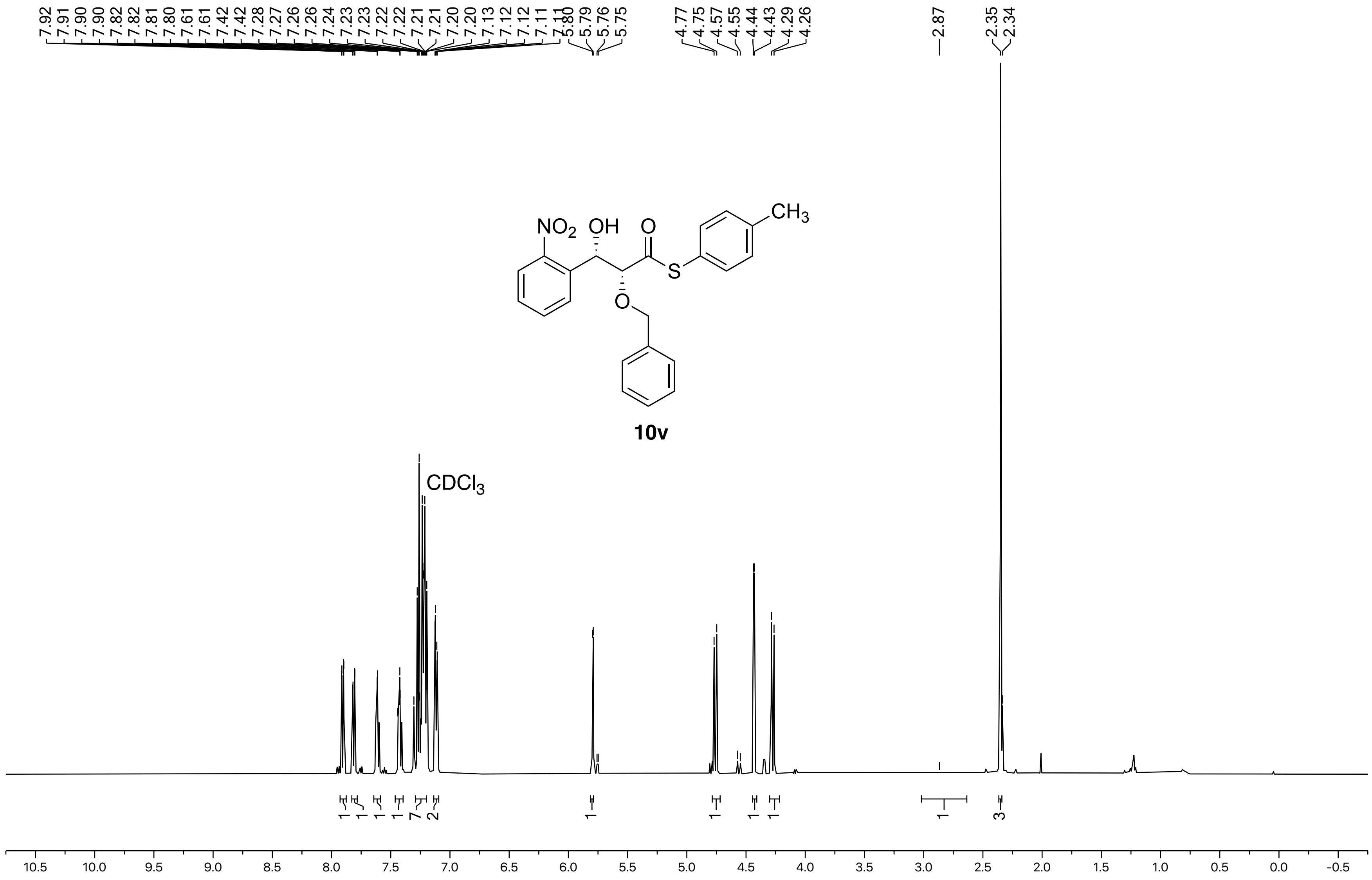

$^{13}C\{^1H\}$ NMR, 126 MHz, $CDCl_3$

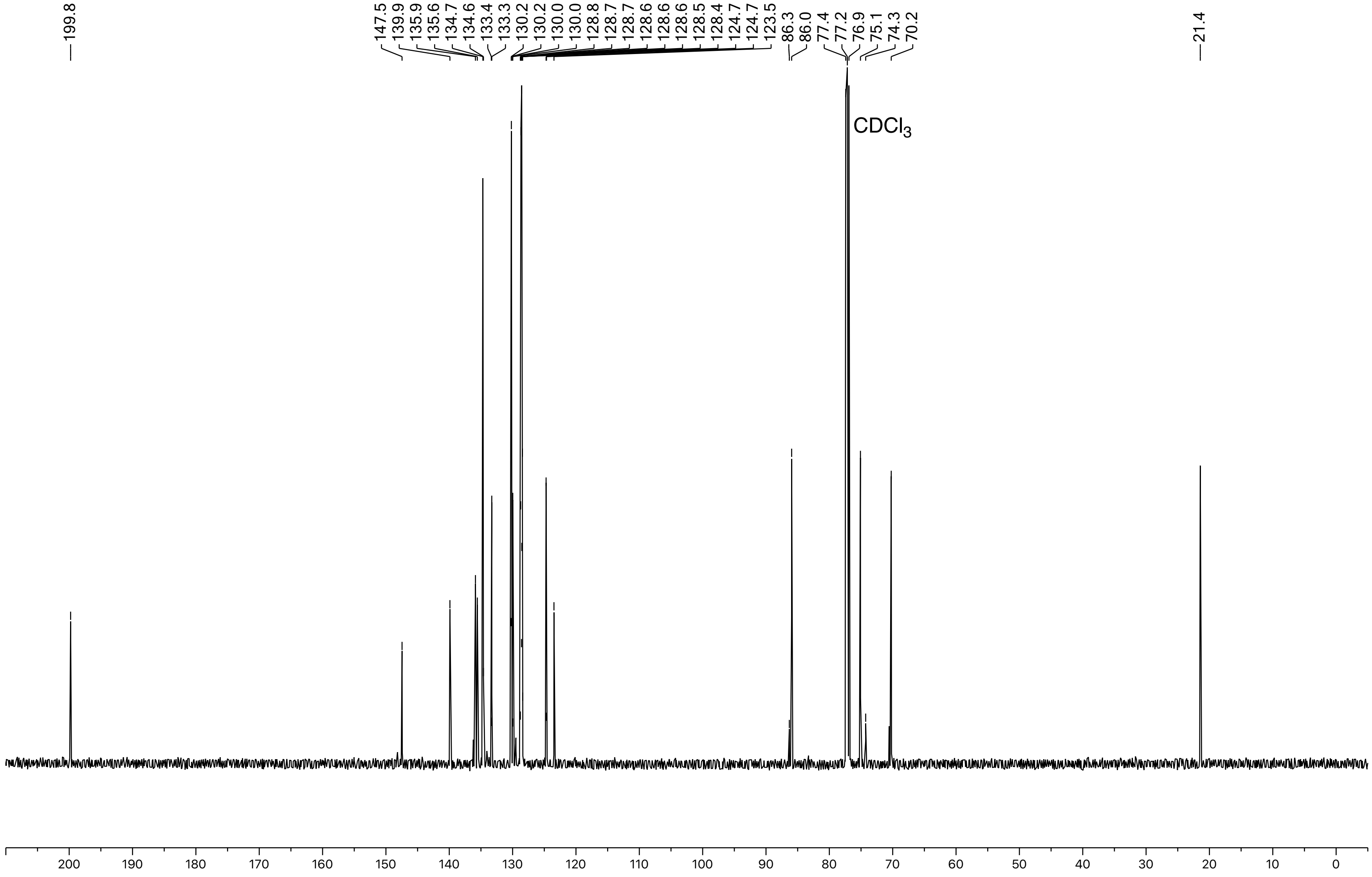

^{1}H NMR, 500 MHz, $CDCl_3$

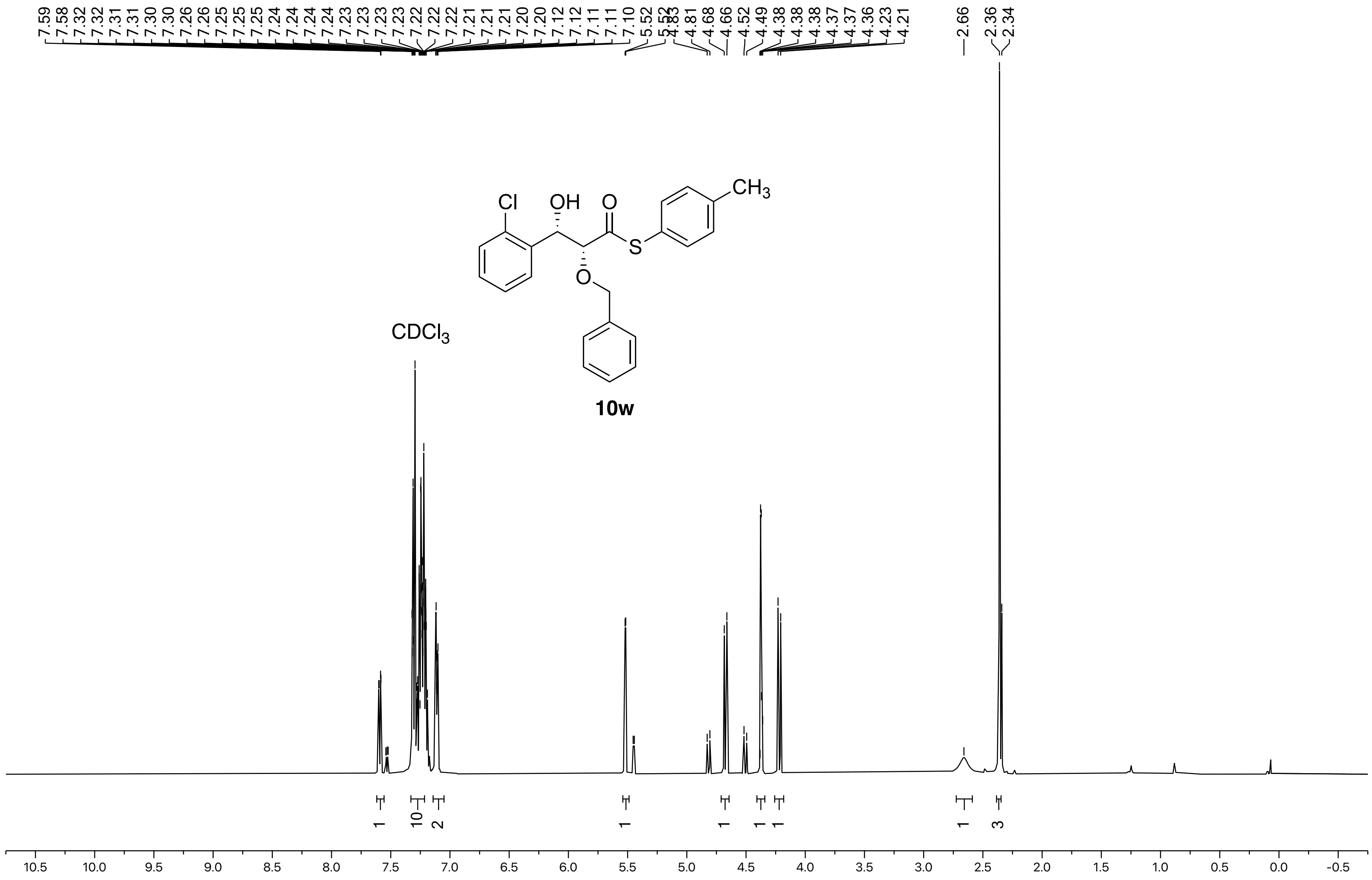

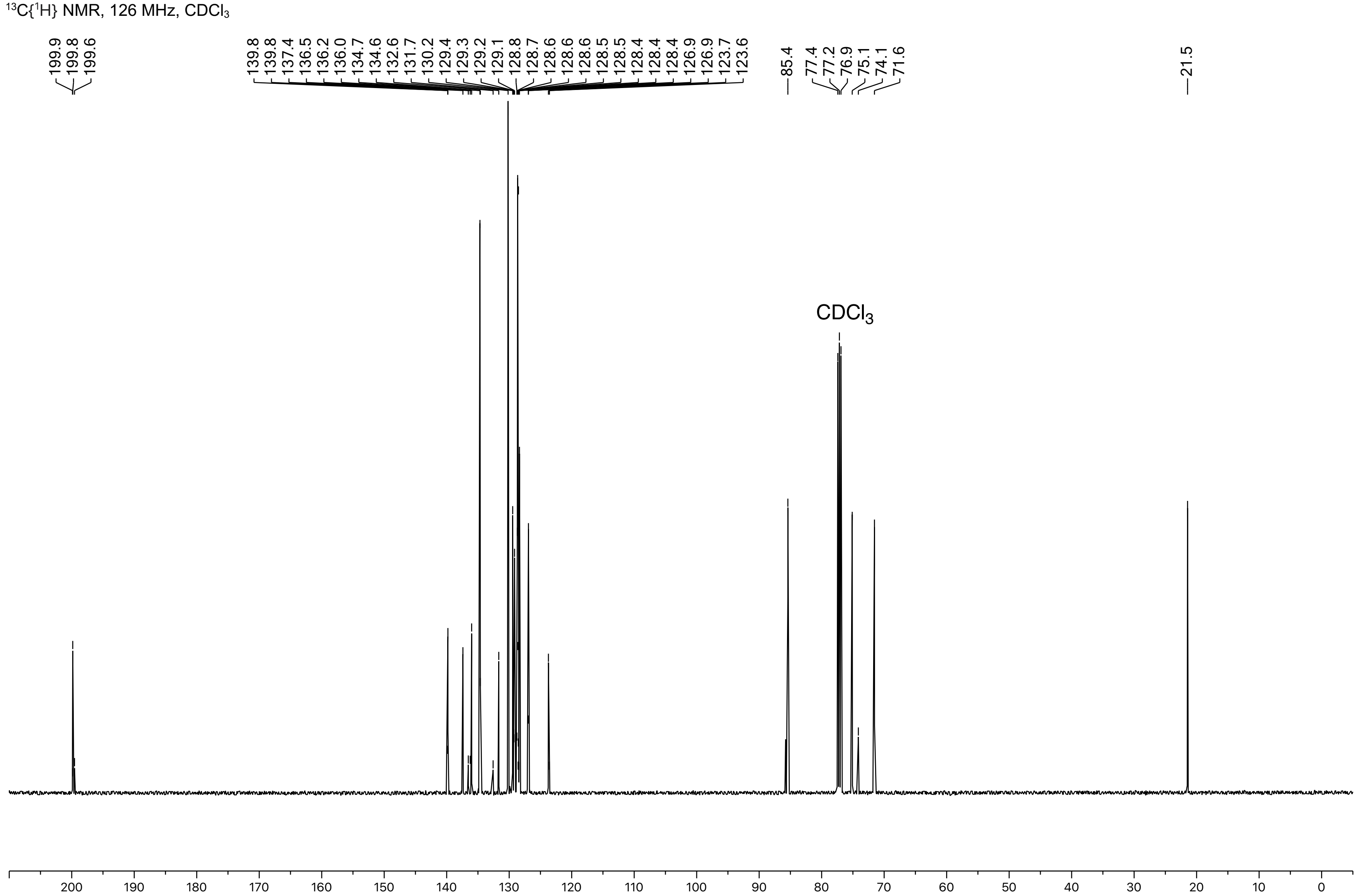

$^{13}C\{^1H\}$ NMR, 126 MHz, $CDCl_3$
199.9
199.8
199.6
139.8
139.8
137.4
136.5
136.2
136.0
134.7
134.6
132.6
131.7
130.2
129.4
129.3
129.2
129.1
128.8
128.7
128.6
128.6
128.6
128.5
128.5
128.4
128.4
128.4
126.9
126.9
123.7
123.6
85.4
77.4
77.2
76.9
75.1
74.1
71.6
21.5
$CDCl_3$
200 190 180 170 160 150 140 130 120 110 100 90 80 70 60 50 40 30 20 10 0

^{1}H NMR, 500 MHz, $CDCl_3$

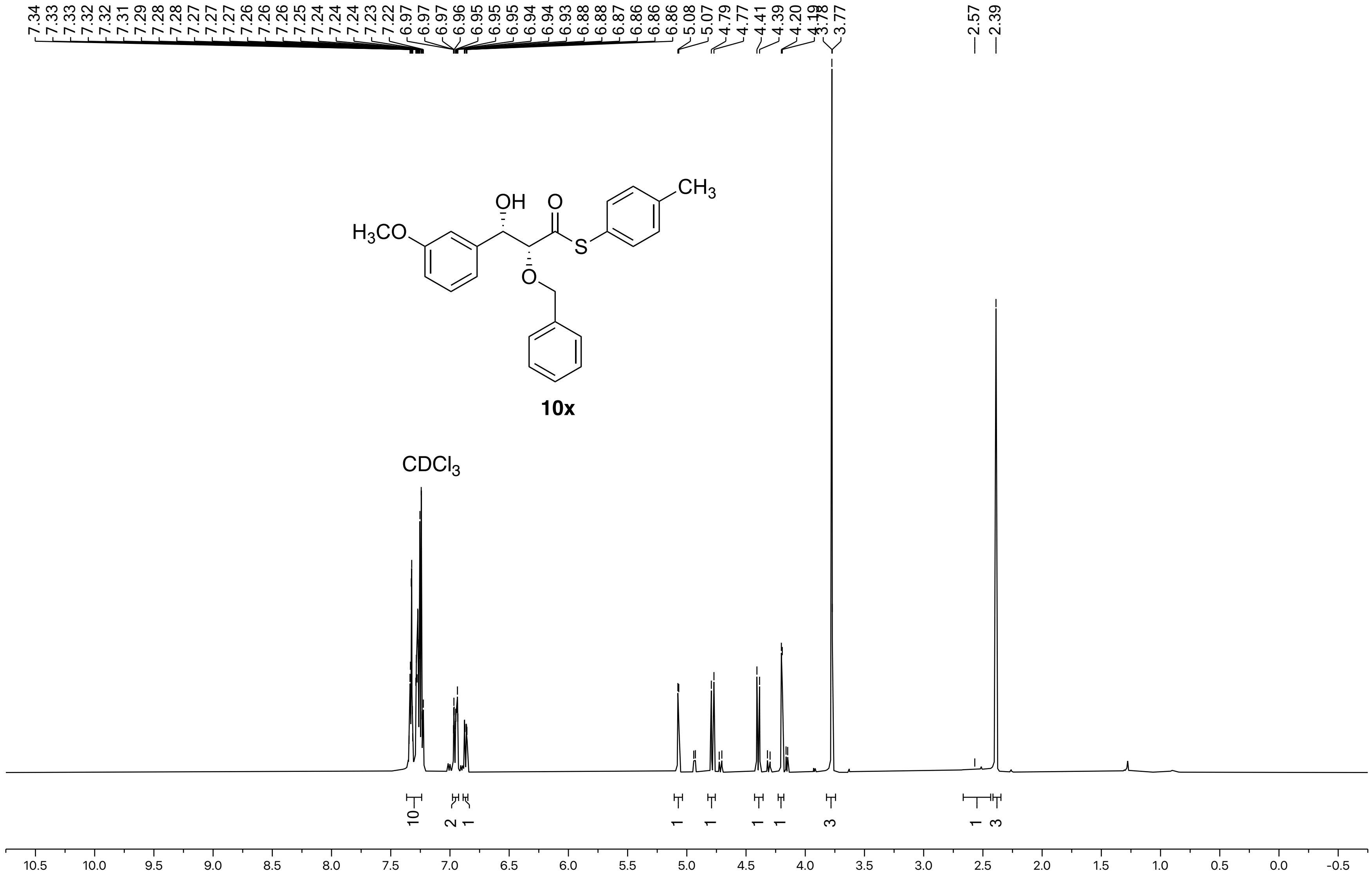

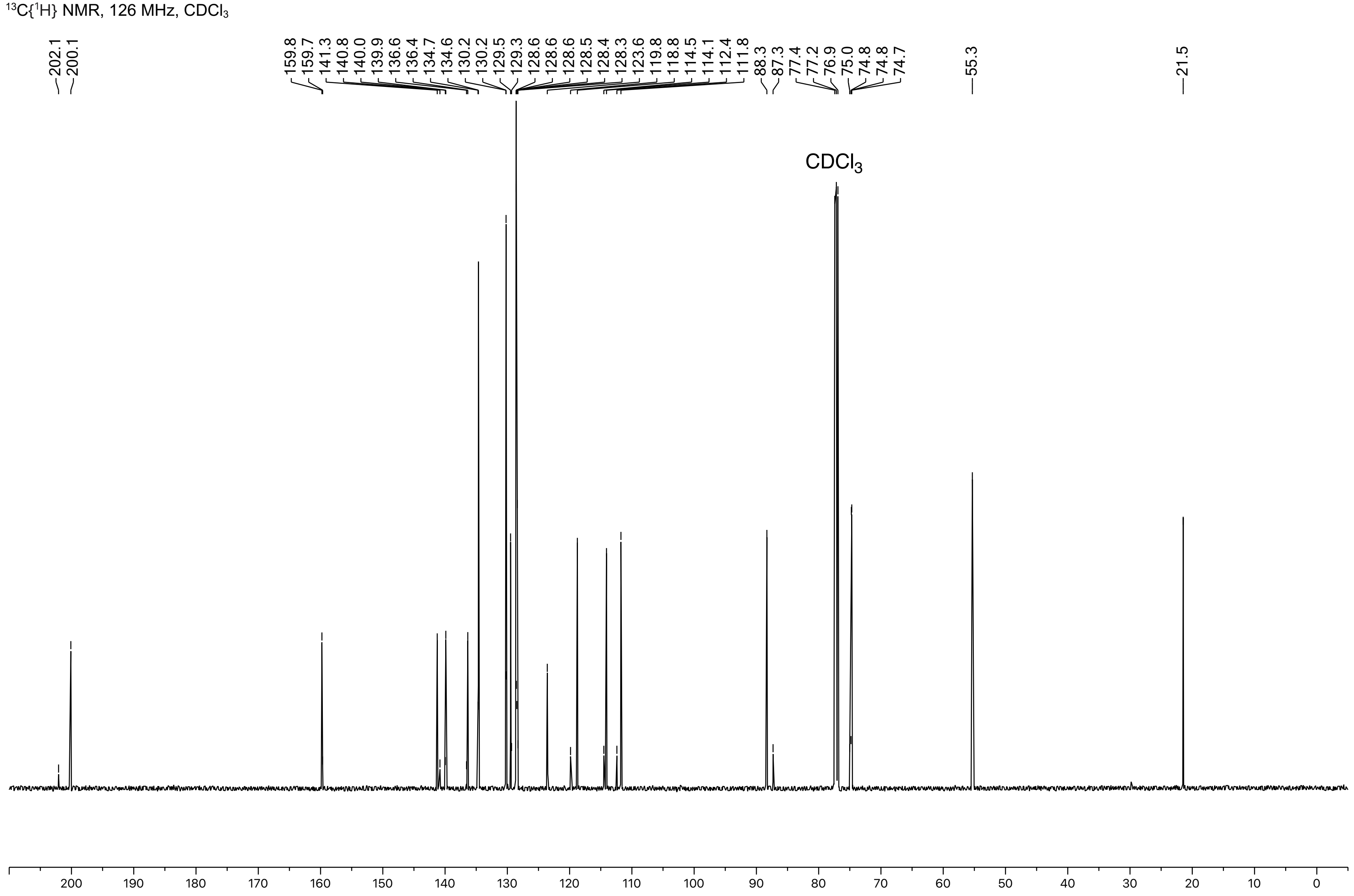

$^{13}C\{^1H\}$ NMR, 126 MHz, $CDCl_3$
202.1
200.1
159.8
159.7
141.3
140.8
140.0
139.9
136.6
136.4
134.7
134.6
130.2
130.2
129.5
129.3
128.6
128.6
128.6
128.5
128.4
128.3
123.6
119.8
118.8
114.5
114.1
112.4
111.8
88.3
87.3
77.4
77.2
76.9
75.0
74.8
74.8
74.7
55.3
21.5
$CDCl_3$
200
190
180
170
160
150
140
130
120
110
100
90
80
70
60
50
40
30
20
10
0

^{1}H NMR, 500 MHz, $CDCl_3$

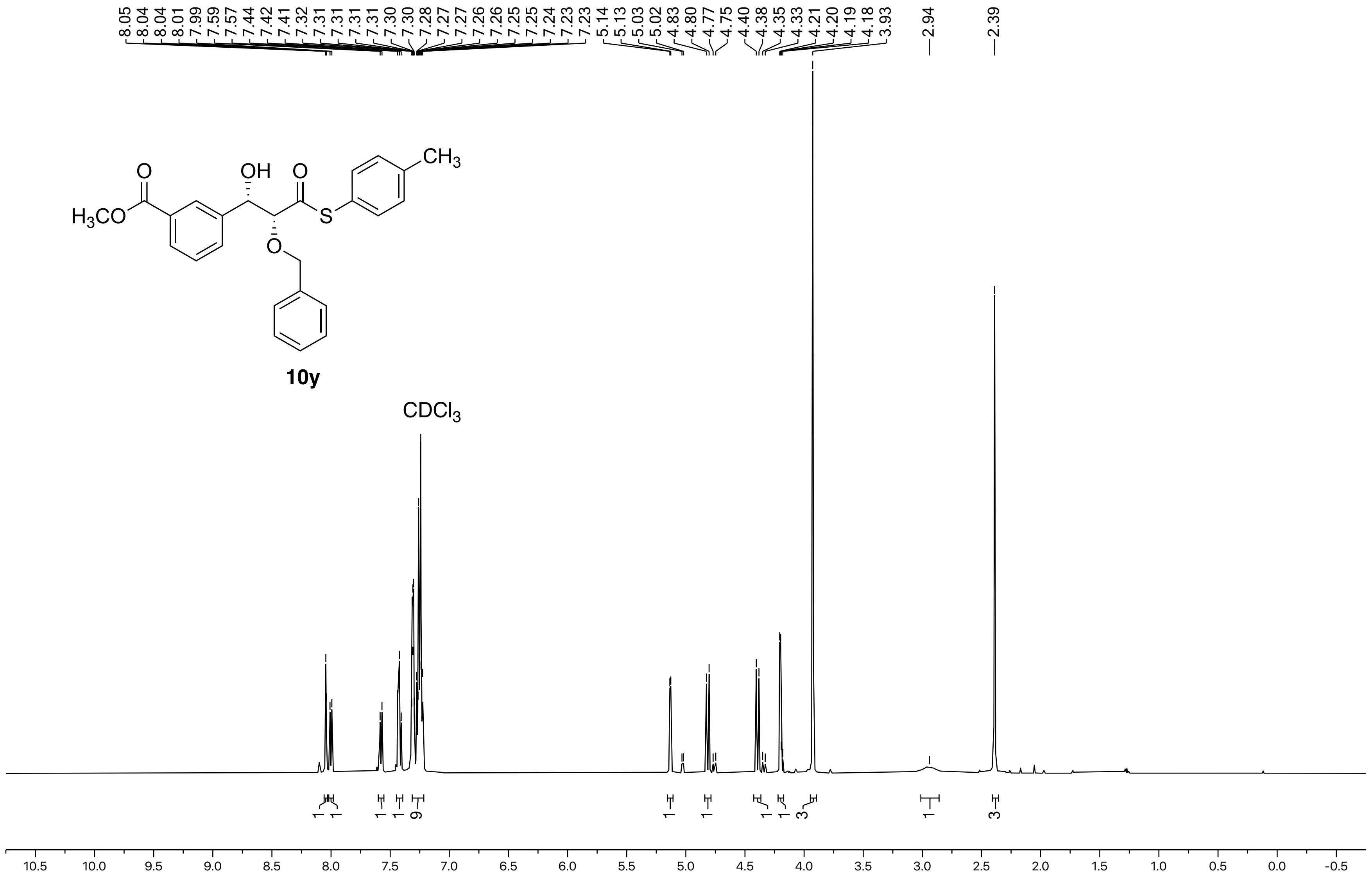

$^{13}C\{^1H\}$ NMR, 126 MHz, $CDCl_3$

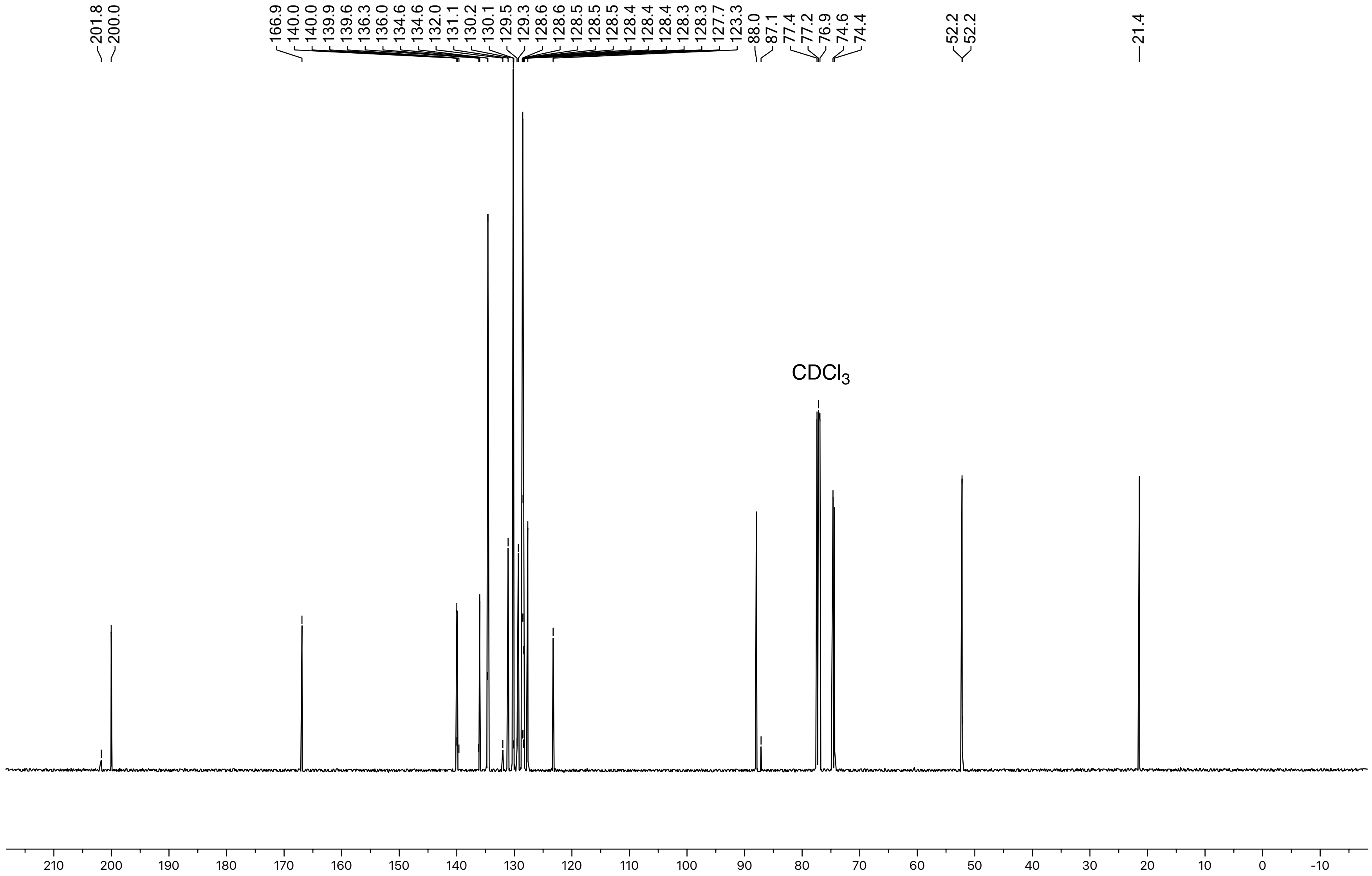

^{1}H NMR, 500 MHz, $CDCl_3$

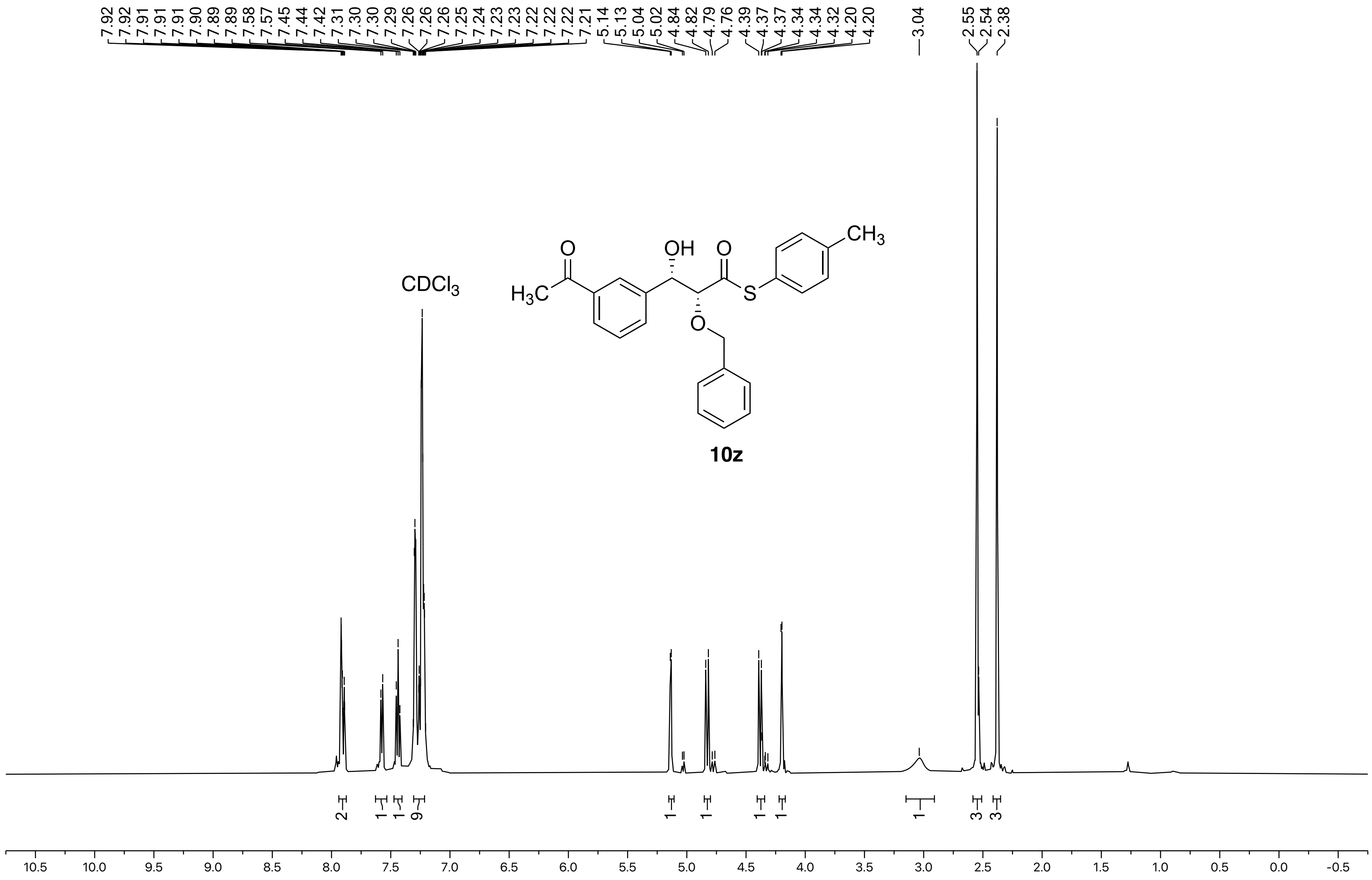

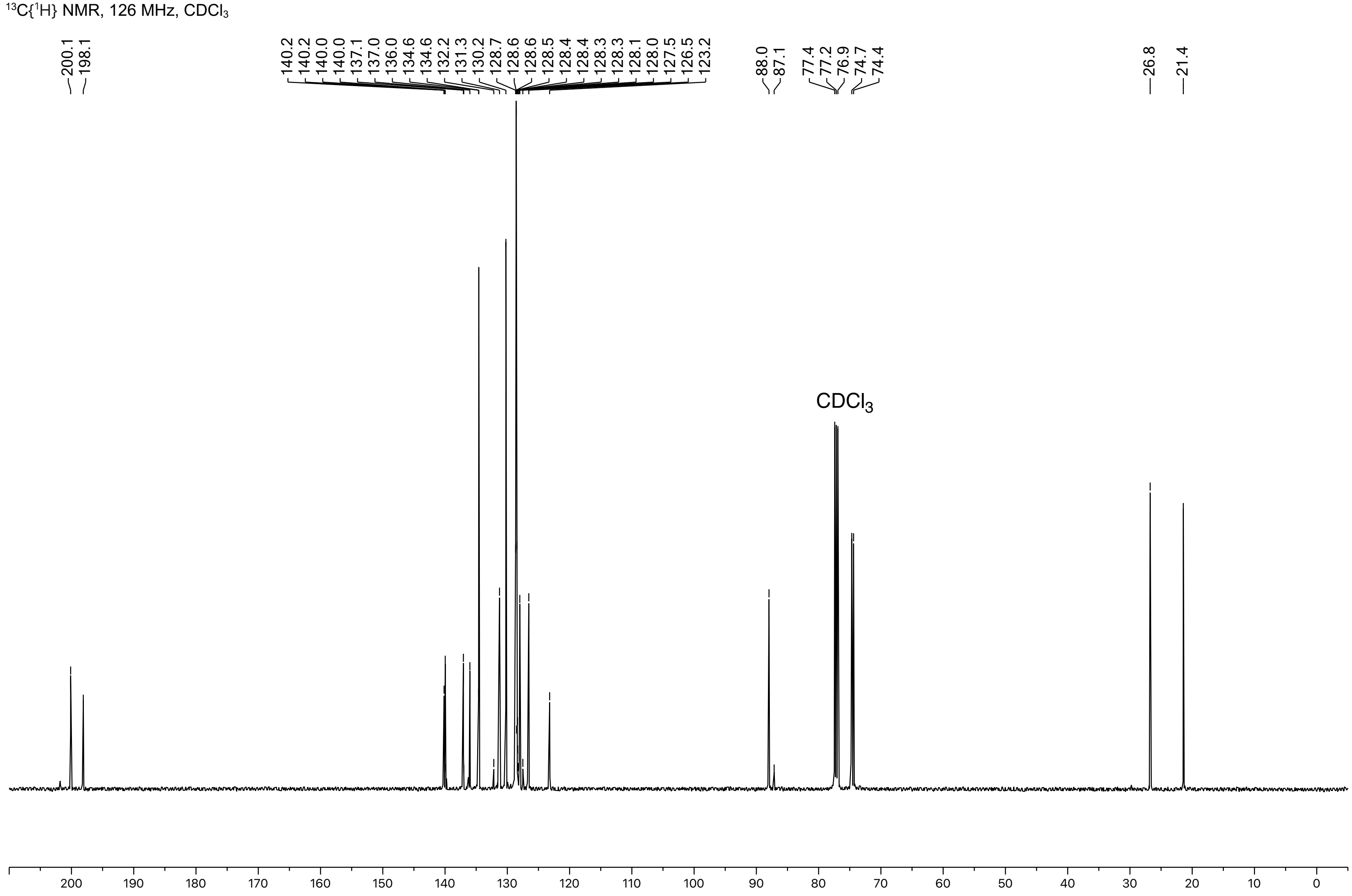
13C{1H} NMR, 126 MHz, CDCl3
200.1
198.1
140.2
140.2
140.0
140.0
137.1
137.0
136.0
134.6
134.6
132.2
131.3
130.2
128.7
128.6
128.6
128.5
128.4
128.4
128.3
128.3
128.1
128.0
127.5
126.5
123.2
88.0
87.1
77.4
77.2
76.9
74.7
74.4
26.8
21.4
CDCl3
200
190
180
170
160
150
140
130
120
110
100
90
80
70
60
50
40
30
20
10
0

^{1}H NMR, 500 MHz, $CDCl_3$

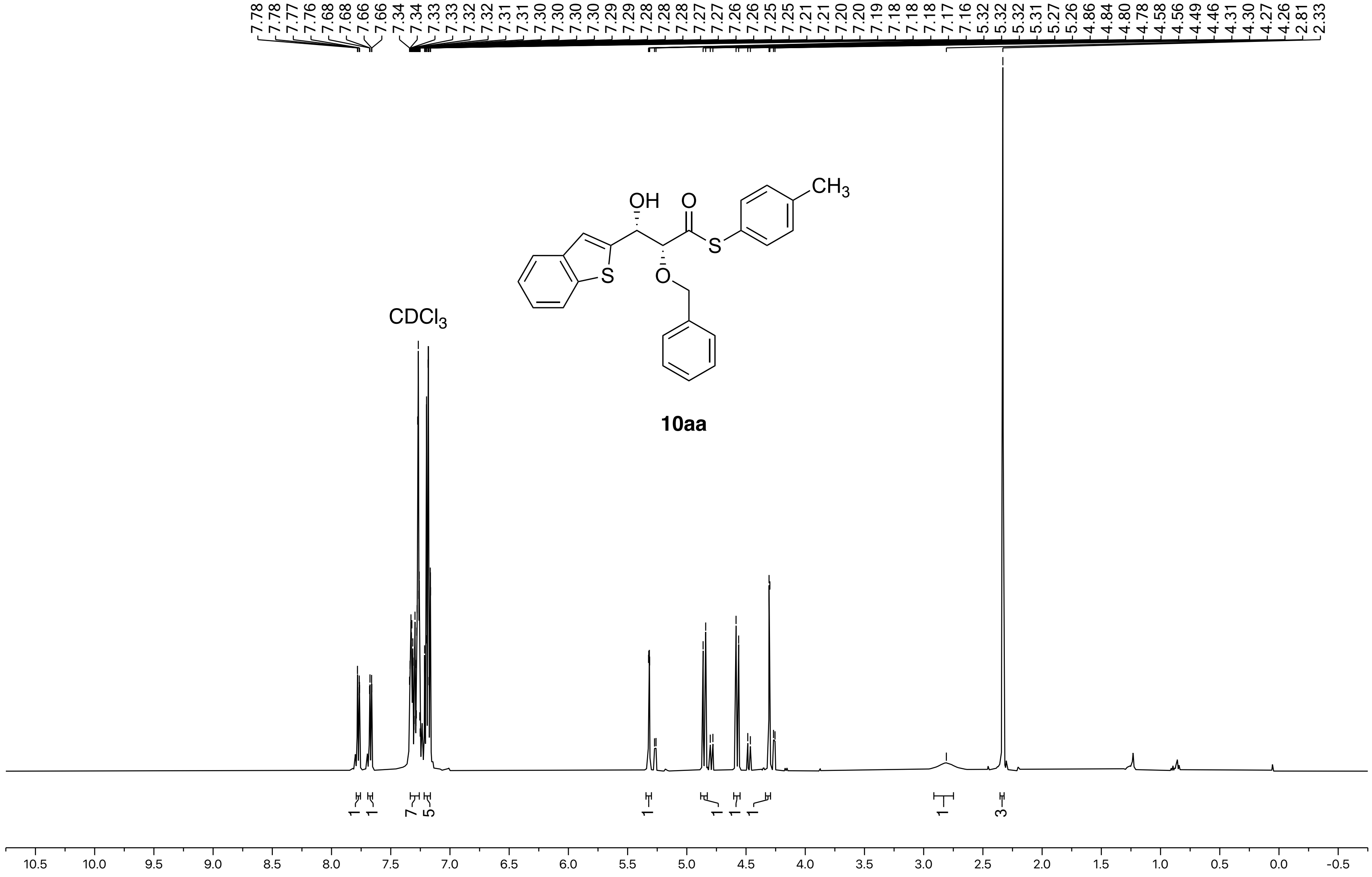

10aa

$^{13}C\{^{1}H\}$ NMR, 126 MHz, $CDCl_3$

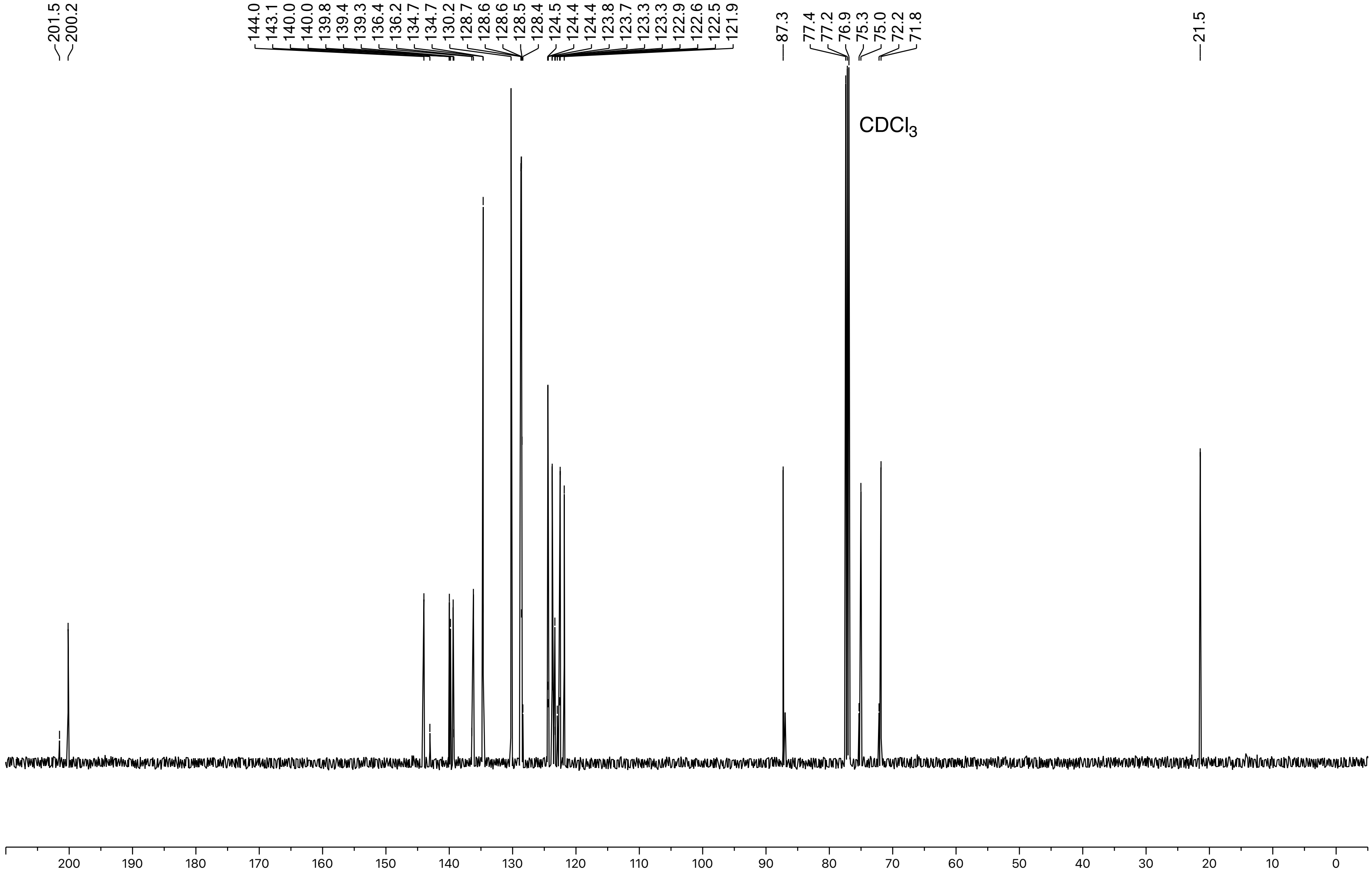

^{1}H NMR, 500 MHz, $CDCl_3$

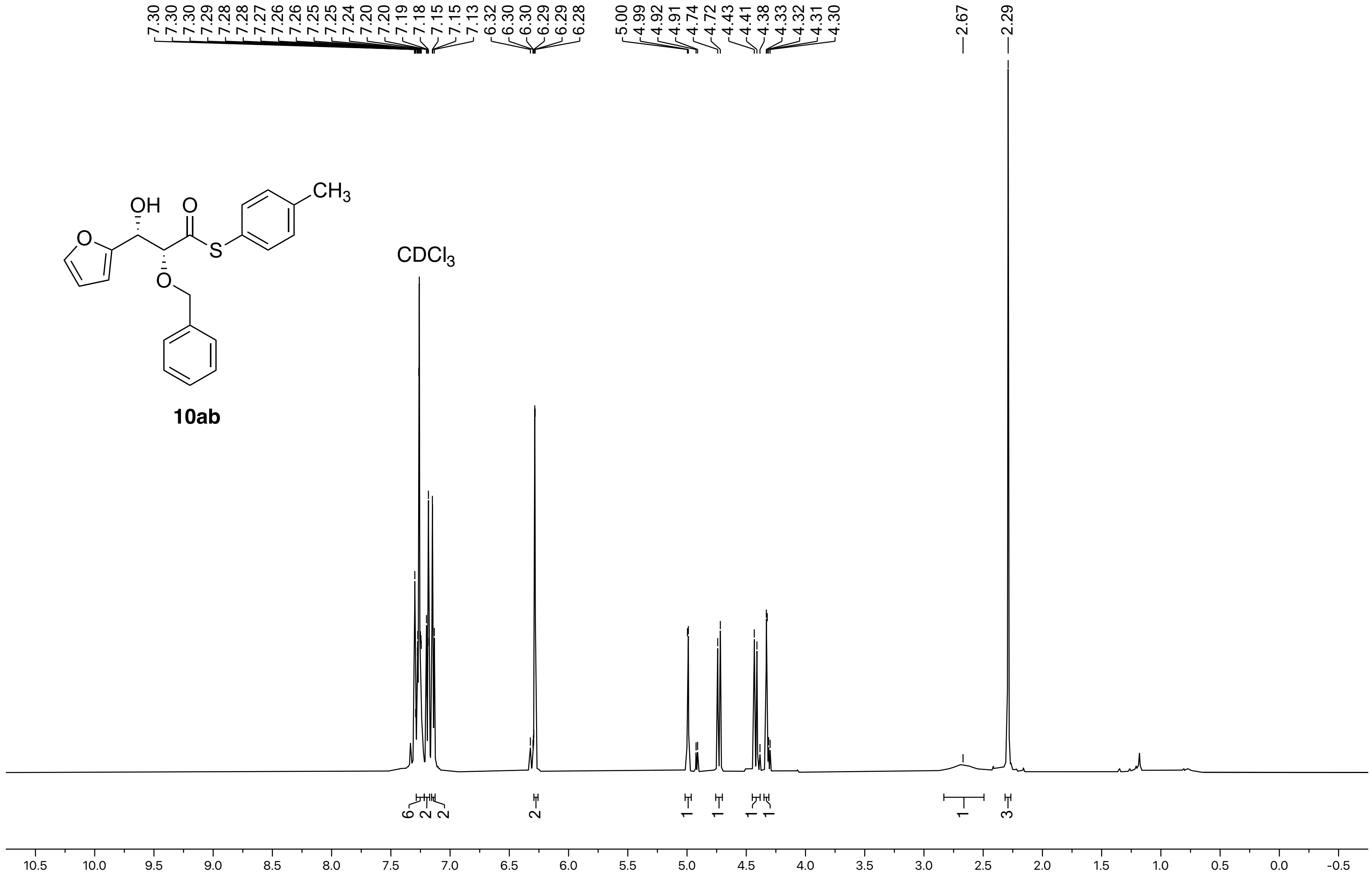

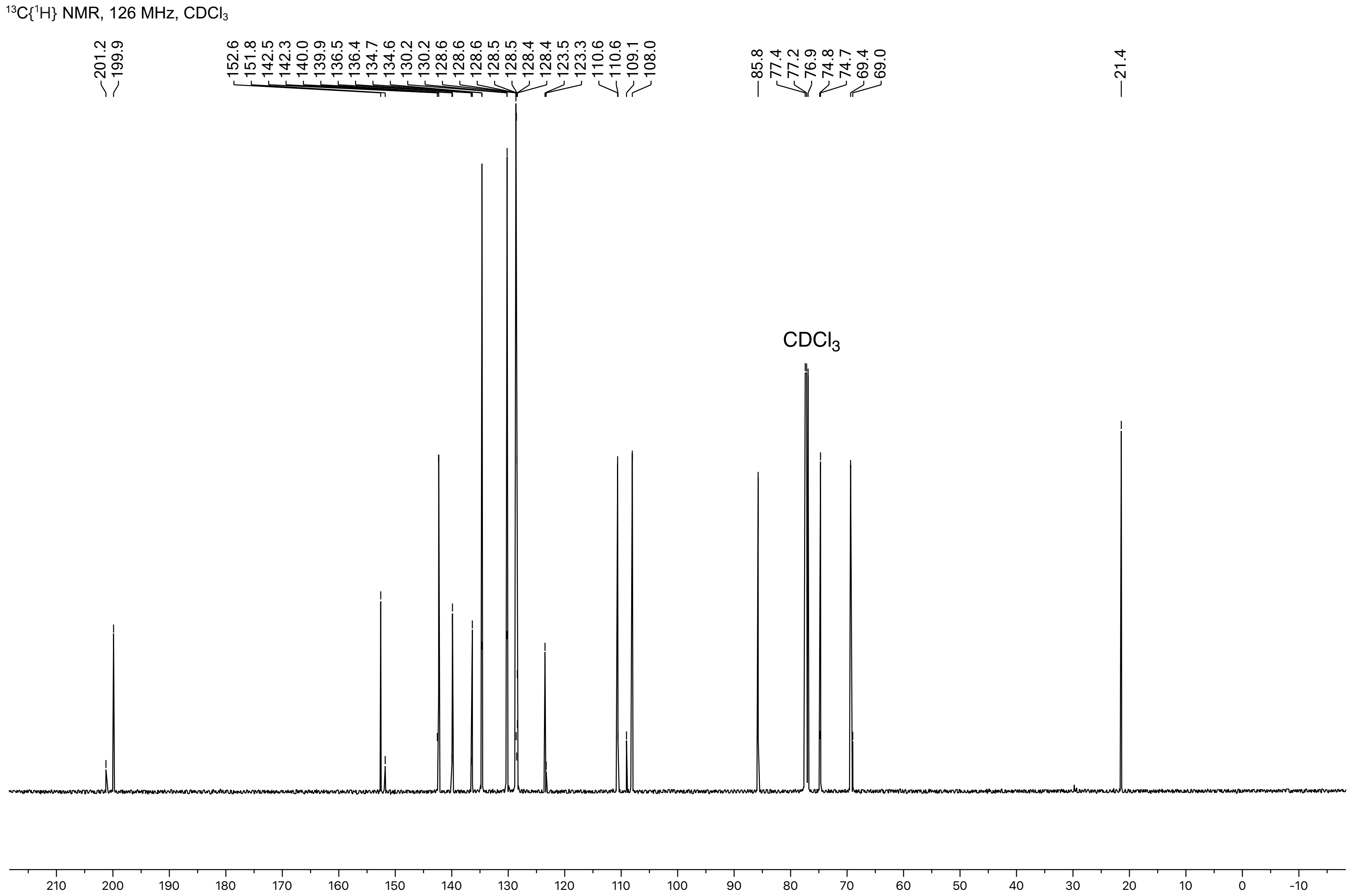

$^{13}C\{^1H\}$ NMR, 126 MHz, $CDCl_3$
201.2
199.9
152.6
151.8
142.5
142.3
140.0
139.9
136.5
136.4
134.7
134.6
130.2
130.2
128.6
128.6
128.6
128.5
128.5
128.4
128.4
123.5
123.3
110.6
110.6
109.1
108.0
85.8
77.4
77.2
76.9
74.8
74.7
69.4
69.0
21.4
$CDCl_3$
210 200 190 180 170 160 150 140 130 120 110 100 90 80 70 60 50 40 30 20 10 0 -10

^{1}H NMR, 500 MHz, $CDCl_3$

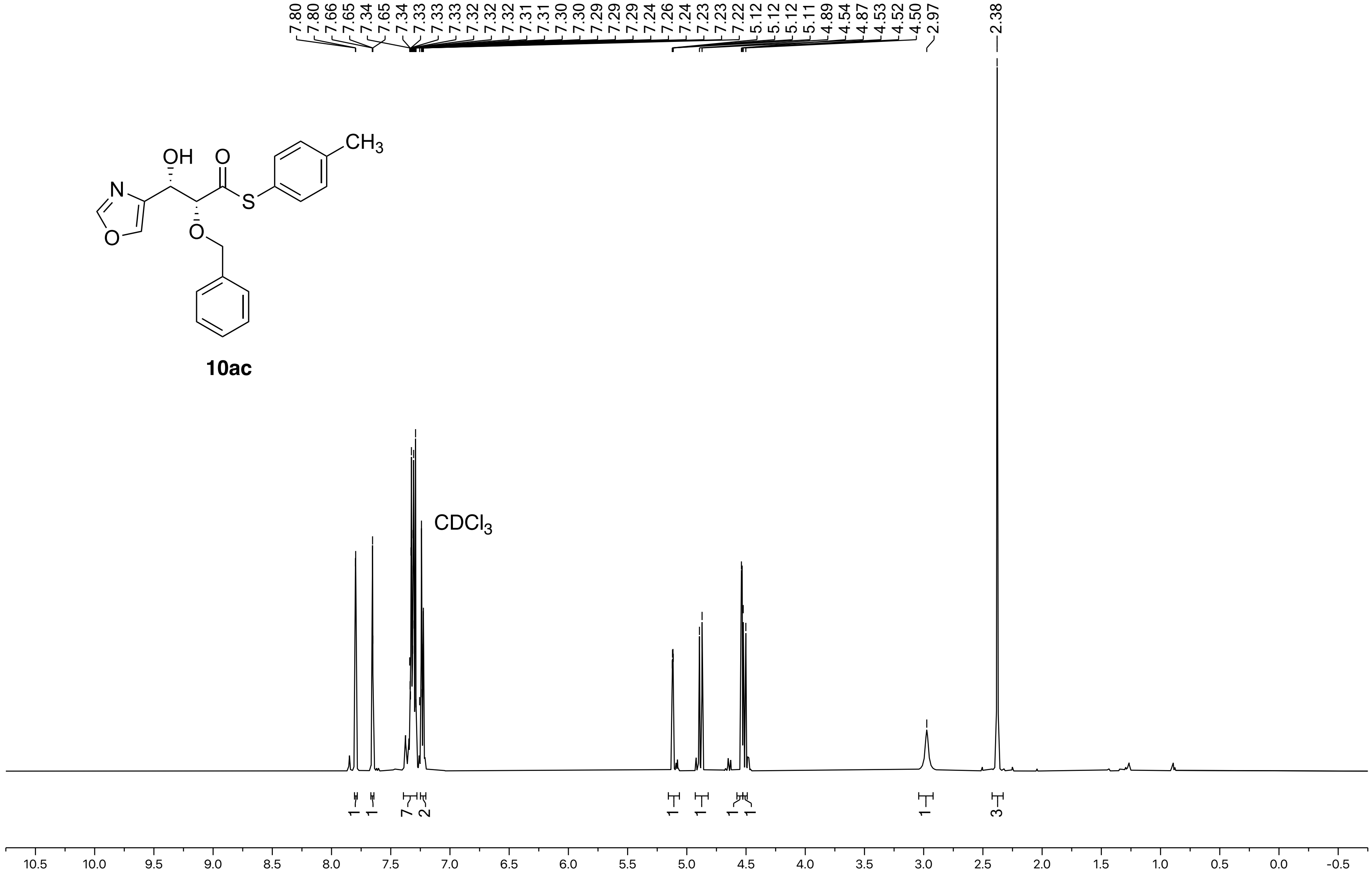

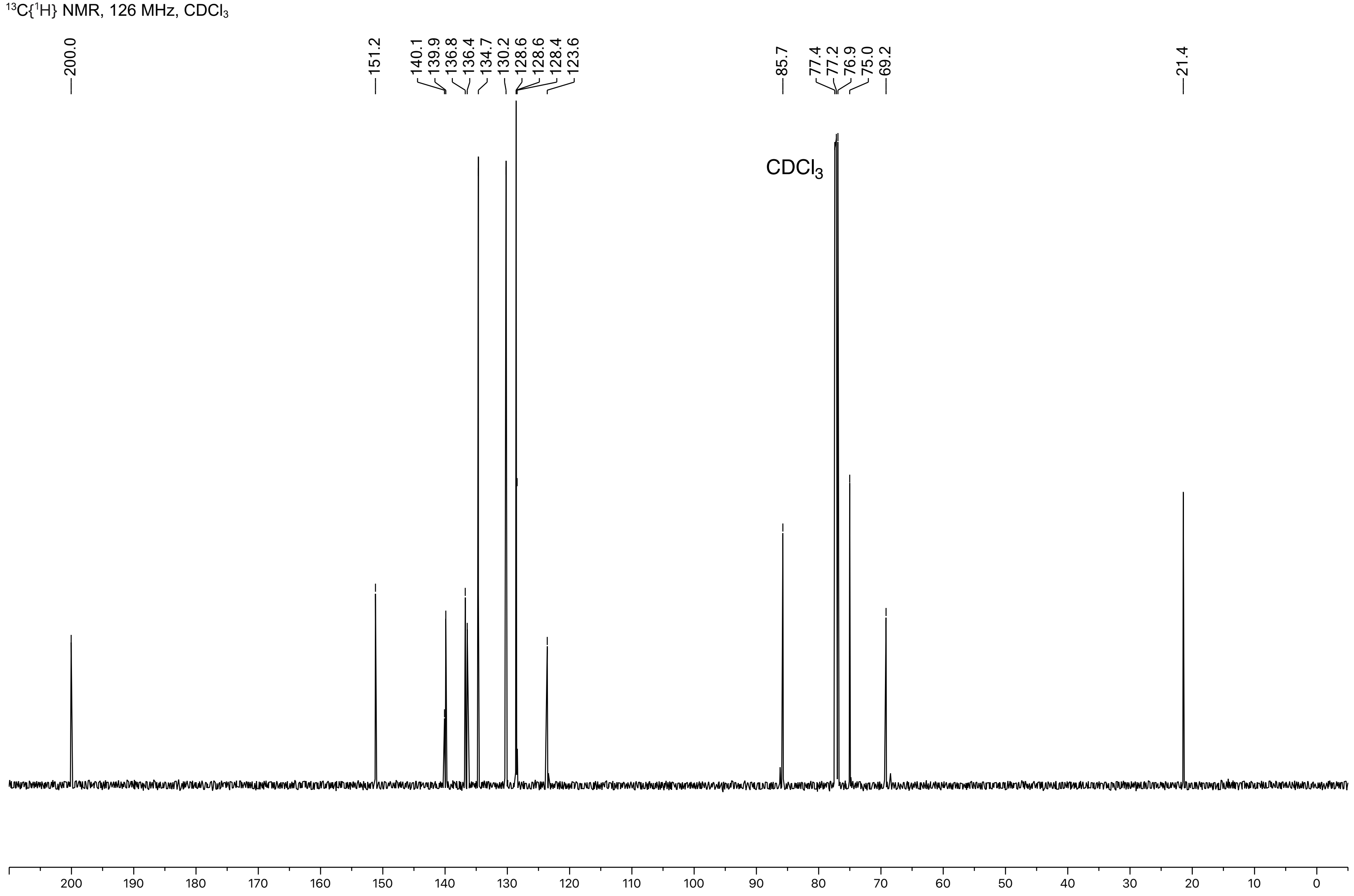
$^{13}C\{^1H\}$ NMR, 126 MHz, $CDCl_3$
200.0
151.2
140.1
139.9
136.8
136.4
134.7
130.2
128.6
128.6
128.4
123.6
85.7
77.4
77.2
76.9
75.0
69.2
21.4
$CDCl_3$
200
190
180
170
160
150
140
130
120
110
100
90
80
70
60
50
40
30
20
10
0

^{1}H NMR, 500 MHz, $CDCl_3$

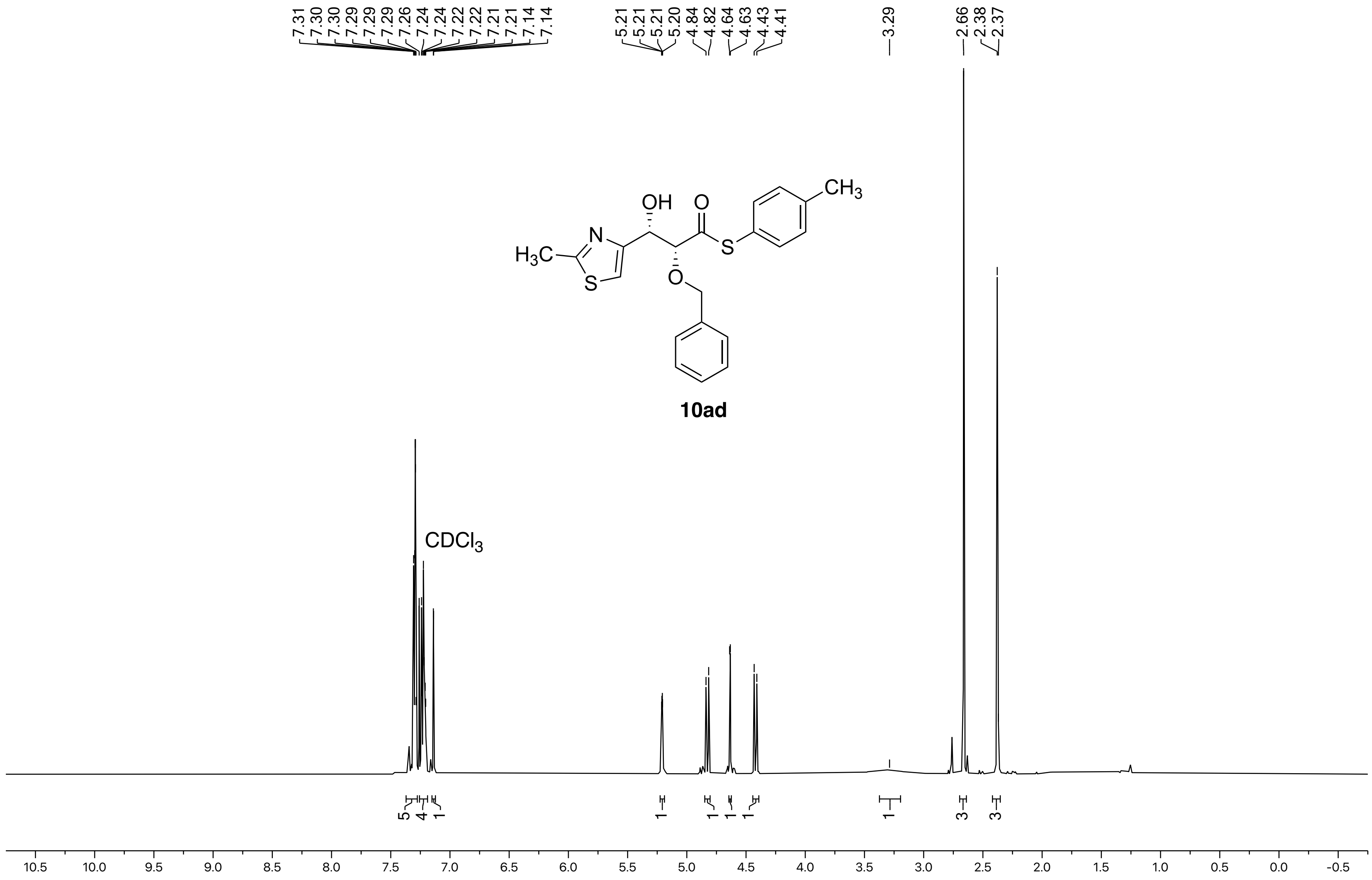

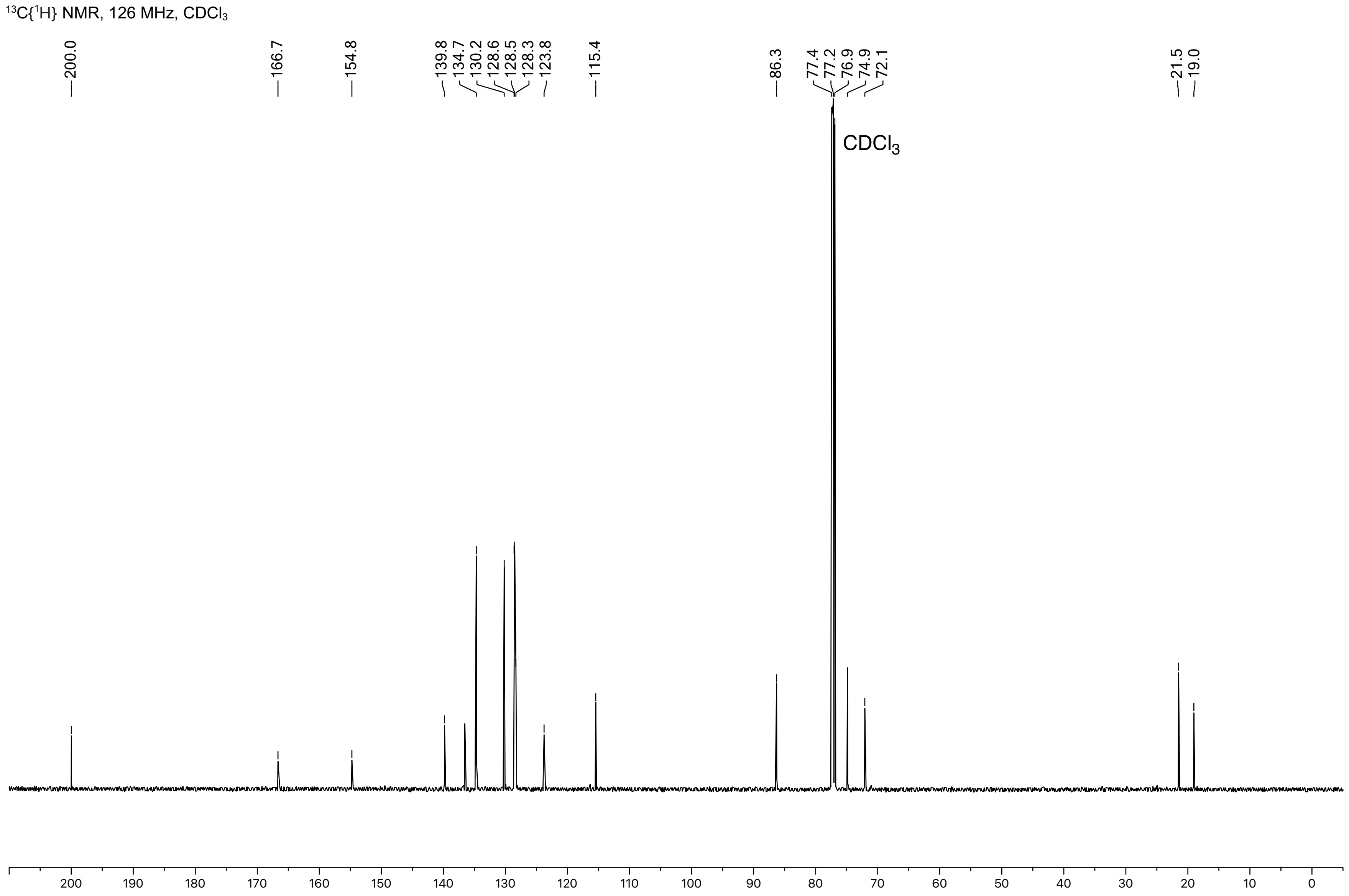

$^{13}C\{^1H\}$ NMR, 126 MHz, $CDCl_3$
200.0
166.7
154.8
139.8
134.7
130.2
128.6
128.5
128.3
123.8
115.4
86.3
77.4
77.2
76.9
74.9
72.1
21.5
19.0
$CDCl_3$
200 190 180 170 160 150 140 130 120 110 100 90 80 70 60 50 40 30 20 10 0

^1H NMR, 500 MHz, $CDCl_3$

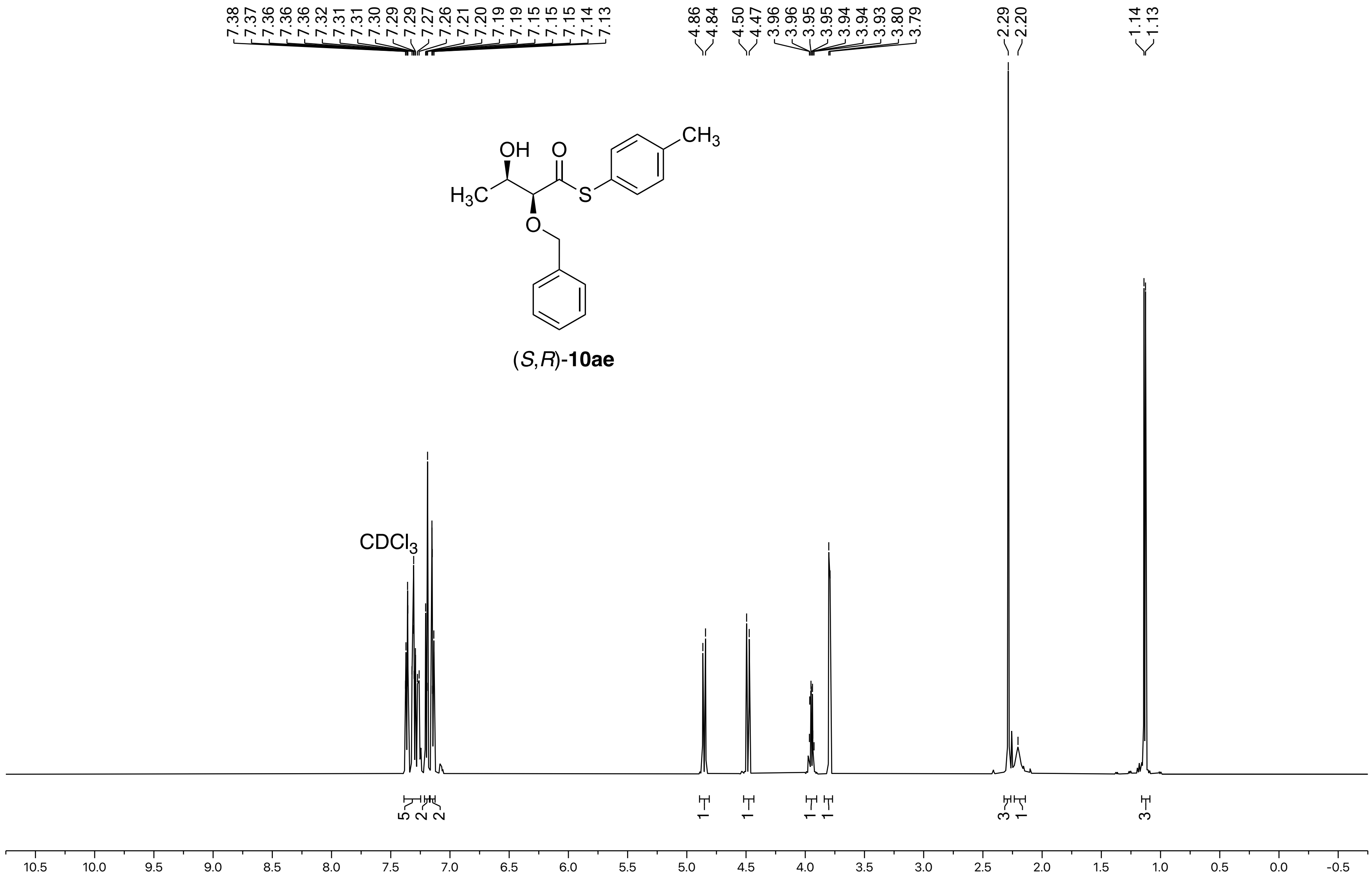

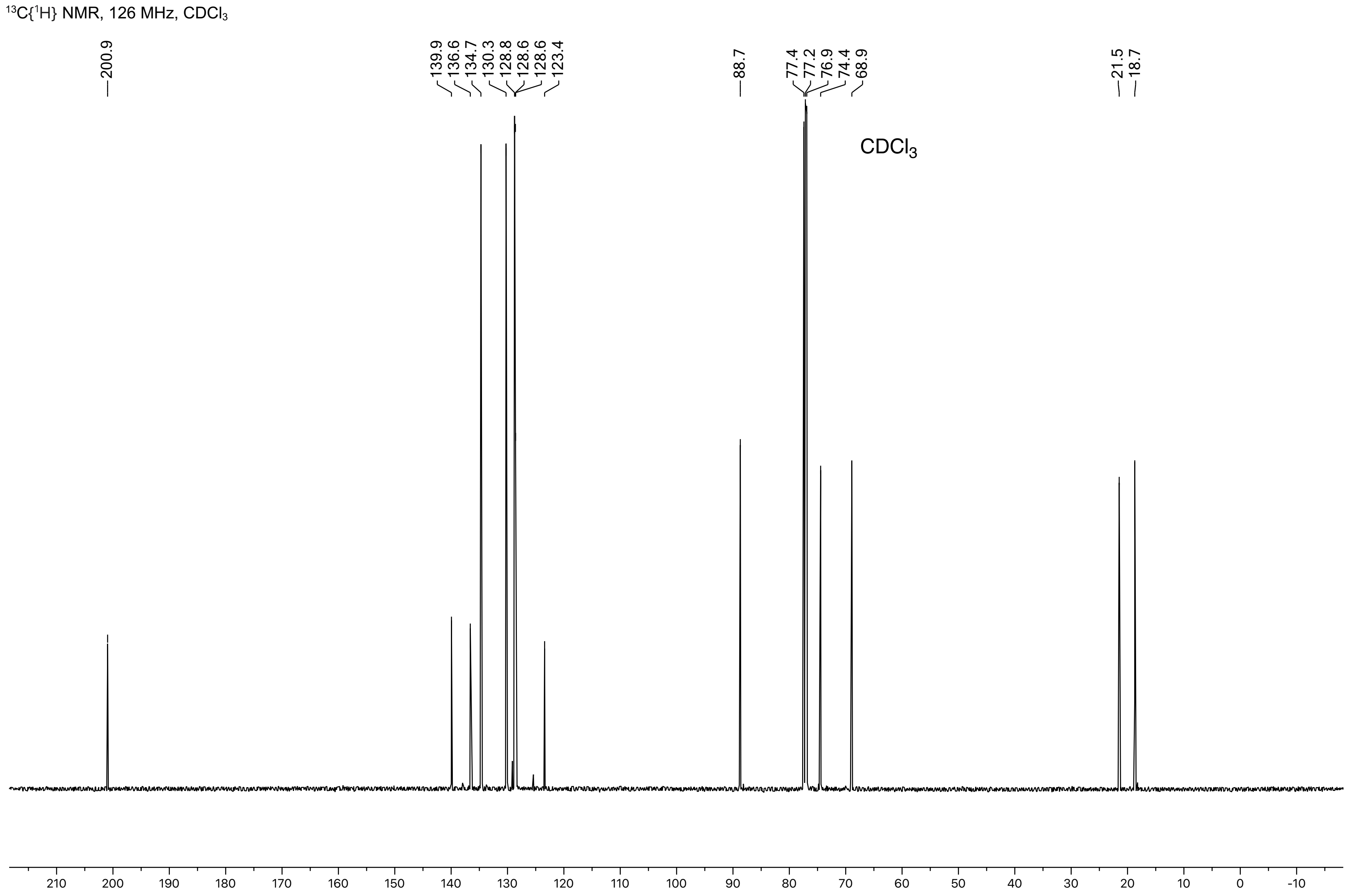

$^{13}C\{^1H\}$ NMR, 126 MHz, $CDCl_3$
200.9
139.9
136.6
134.7
130.3
128.8
128.6
128.6
123.4
88.7
77.4
77.2
76.9
74.4
68.9
21.5
18.7
$CDCl_3$
210
200
190
180
170
160
150
140
130
120
110
100
90
80
70
60
50
40
30
20
10
0
-10

^{1}H NMR, 500 MHz, $CDCl_3$

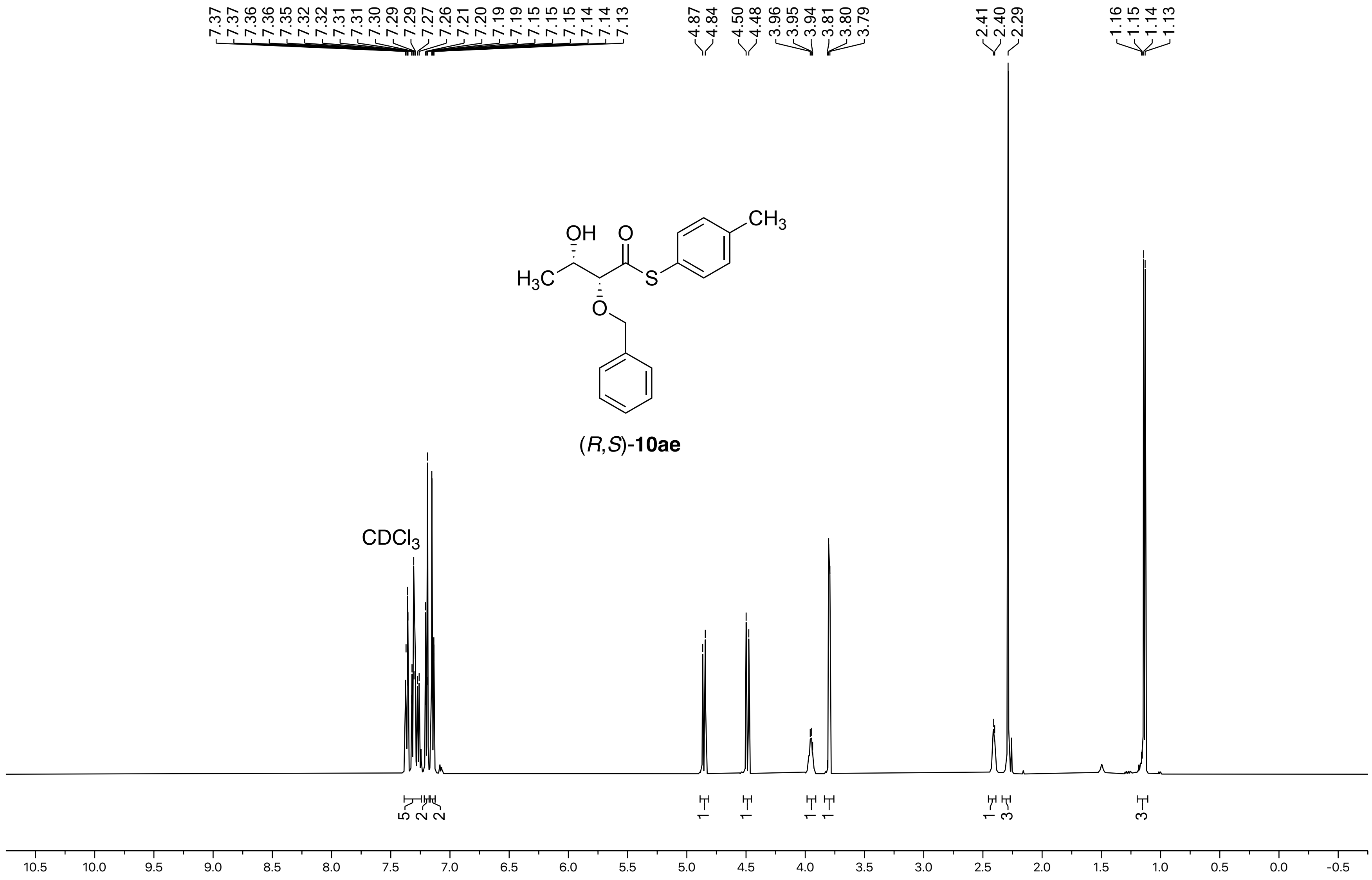

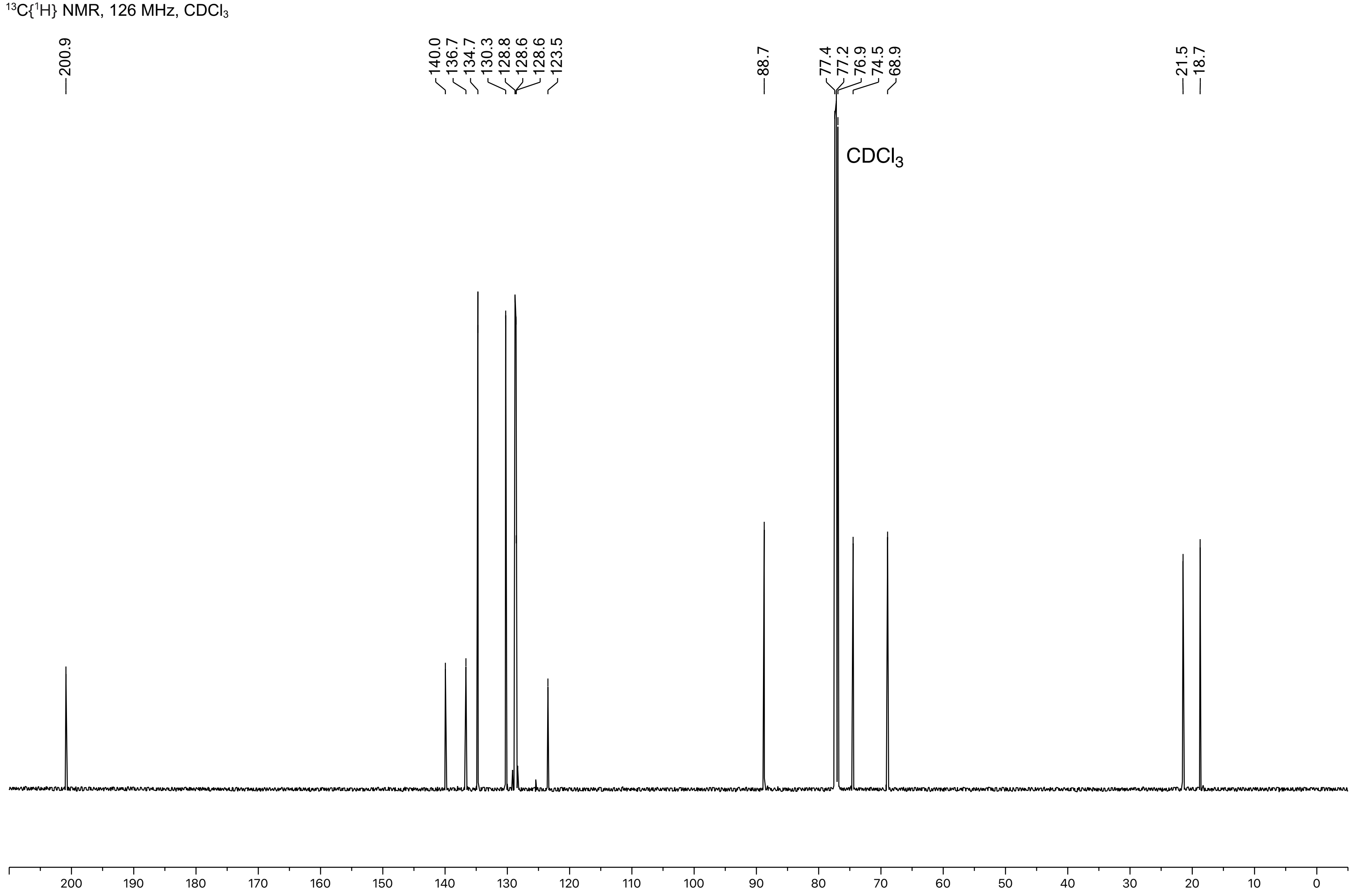
$^{13}C\{^{1}H\}$ NMR, 126 MHz, $CDCl_3$
200.9
140.0
136.7
134.7
130.3
128.8
128.6
128.6
123.5
88.7
77.4
77.2
76.9
74.5
68.9
21.5
18.7
$CDCl_3$
200 190 180 170 160 150 140 130 120 110 100 90 80 70 60 50 40 30 20 10 0

^1H NMR, 500 MHz, $CDCl_3$

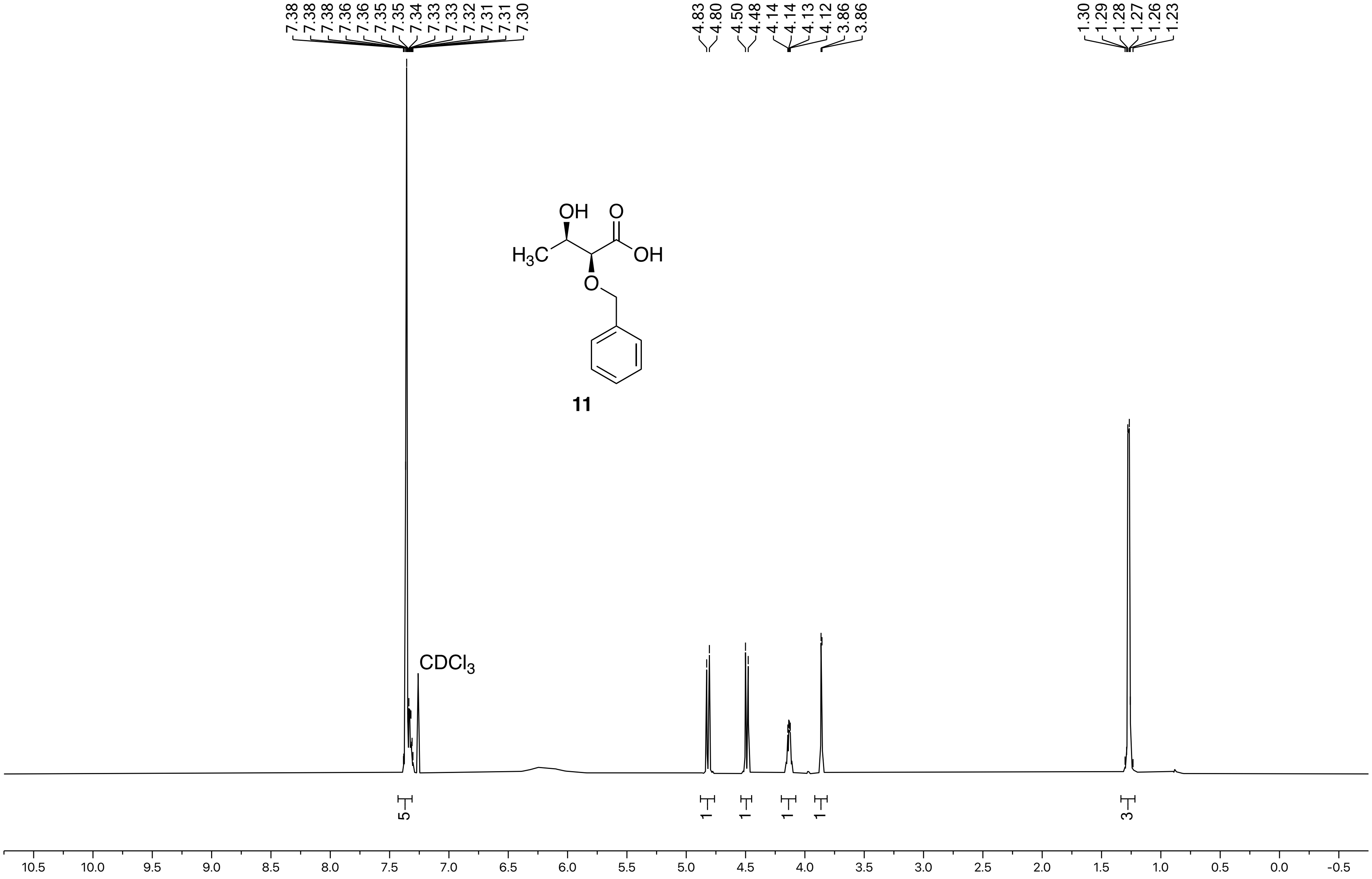

11

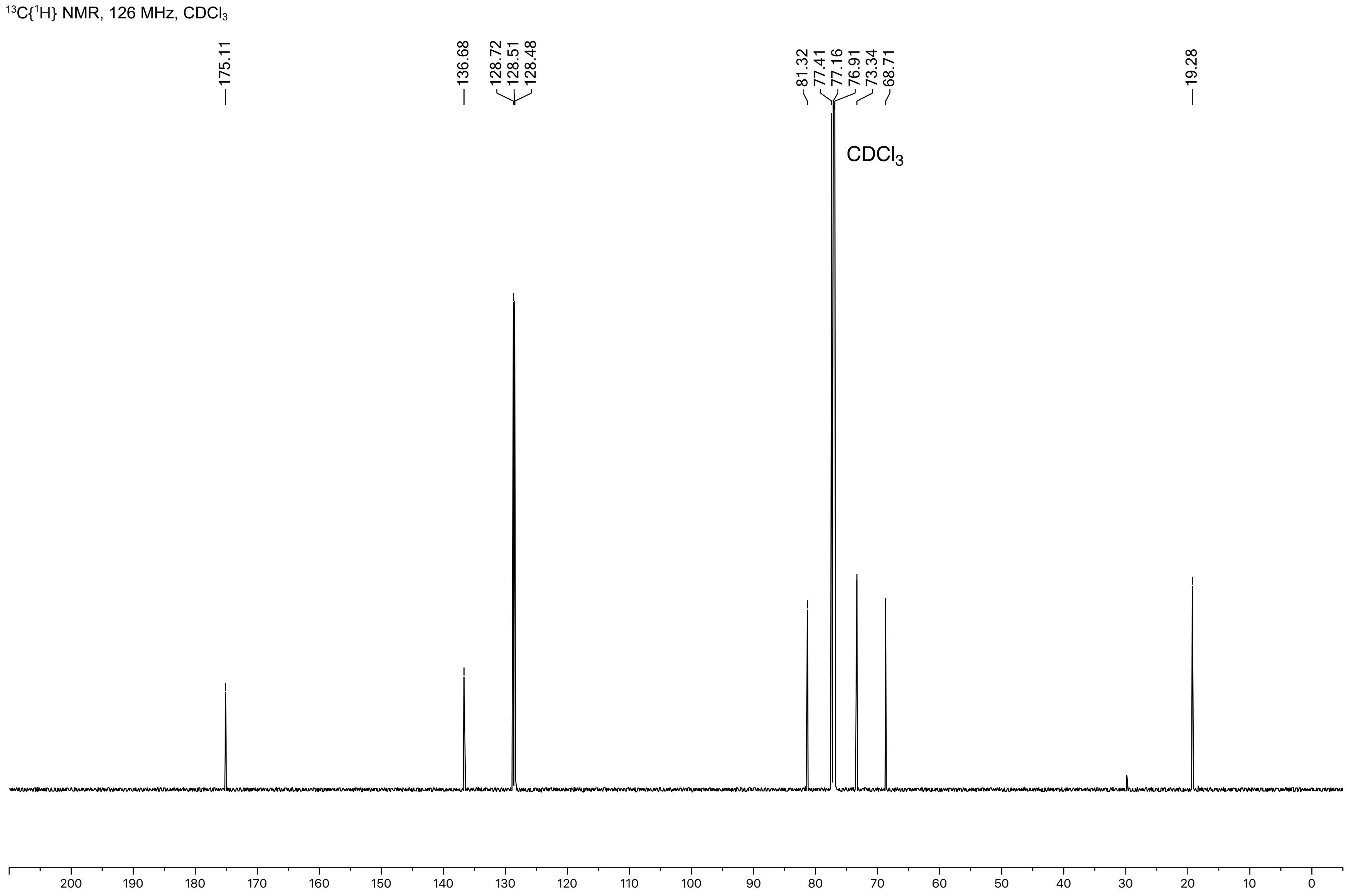

$^{13}C\{^1H\}$ NMR, 126 MHz, $CDCl_3$
175.11
136.68
128.72
128.51
128.48
81.32
77.41
77.16
76.91
73.34
68.71
19.28
$CDCl_3$
200 190 180 170 160 150 140 130 120 110 100 90 80 70 60 50 40 30 20 10 0

^{1}H NMR, 500 MHz, $CDCl_3$

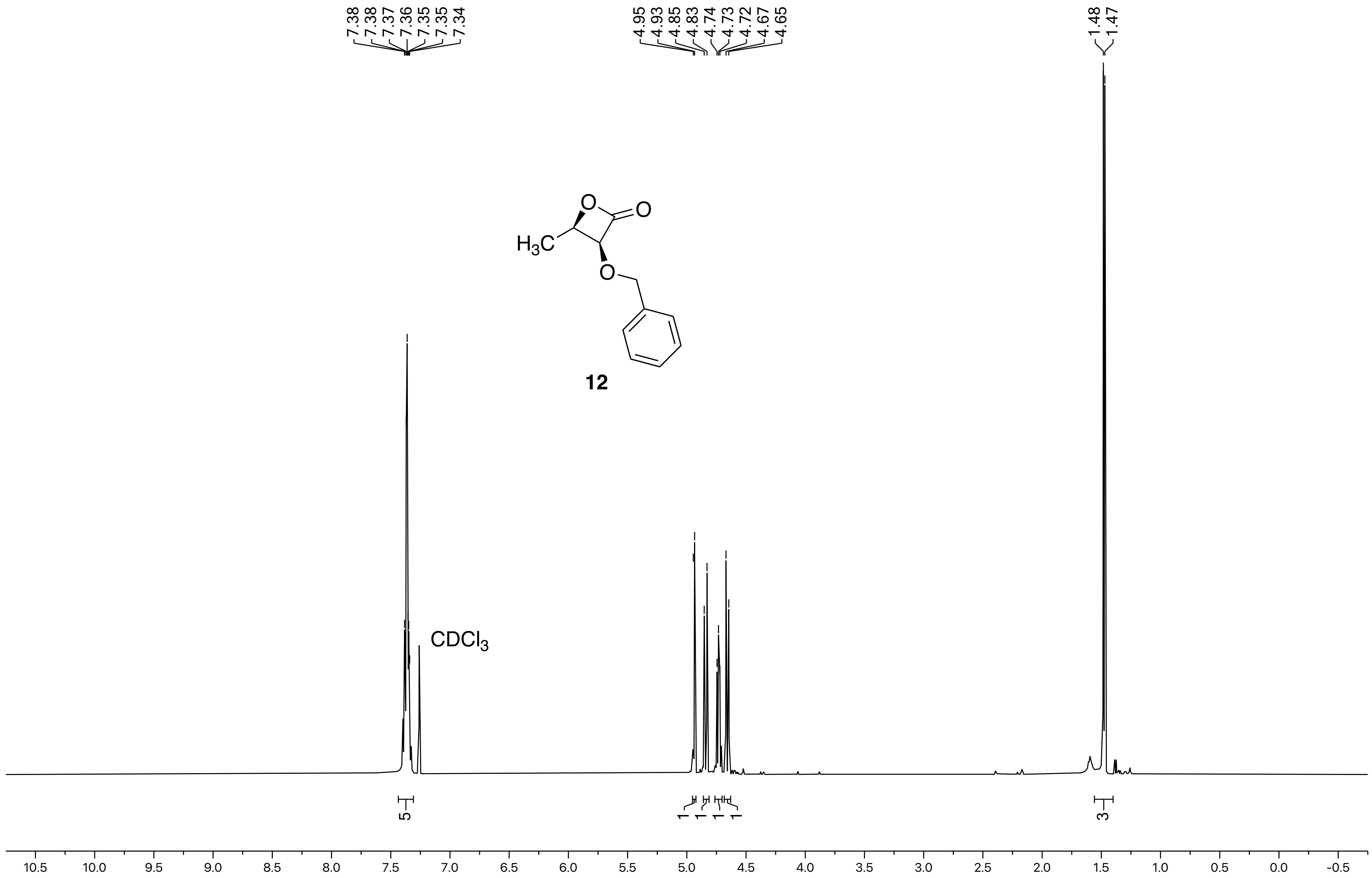

12

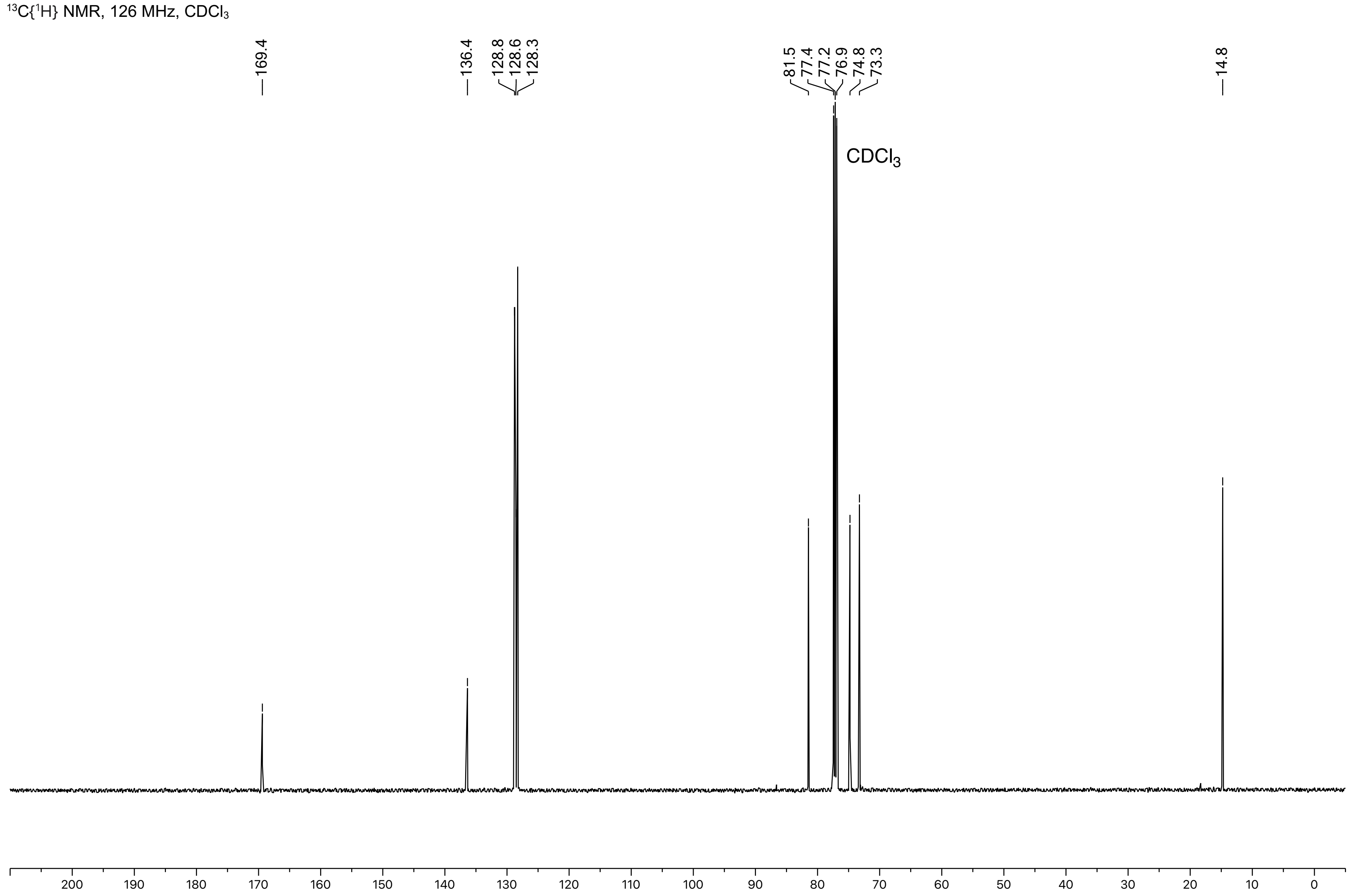

$^{13}C\{^1H\}$ NMR, 126 MHz, $CDCl_3$
169.4
136.4
128.8
128.6
128.3
81.5
77.4
77.2
76.9
74.8
73.3
14.8
$CDCl_3$
200 190 180 170 160 150 140 130 120 110 100 90 80 70 60 50 40 30 20 10 0

^{1}H NMR, 500 MHz, $CDCl_3$

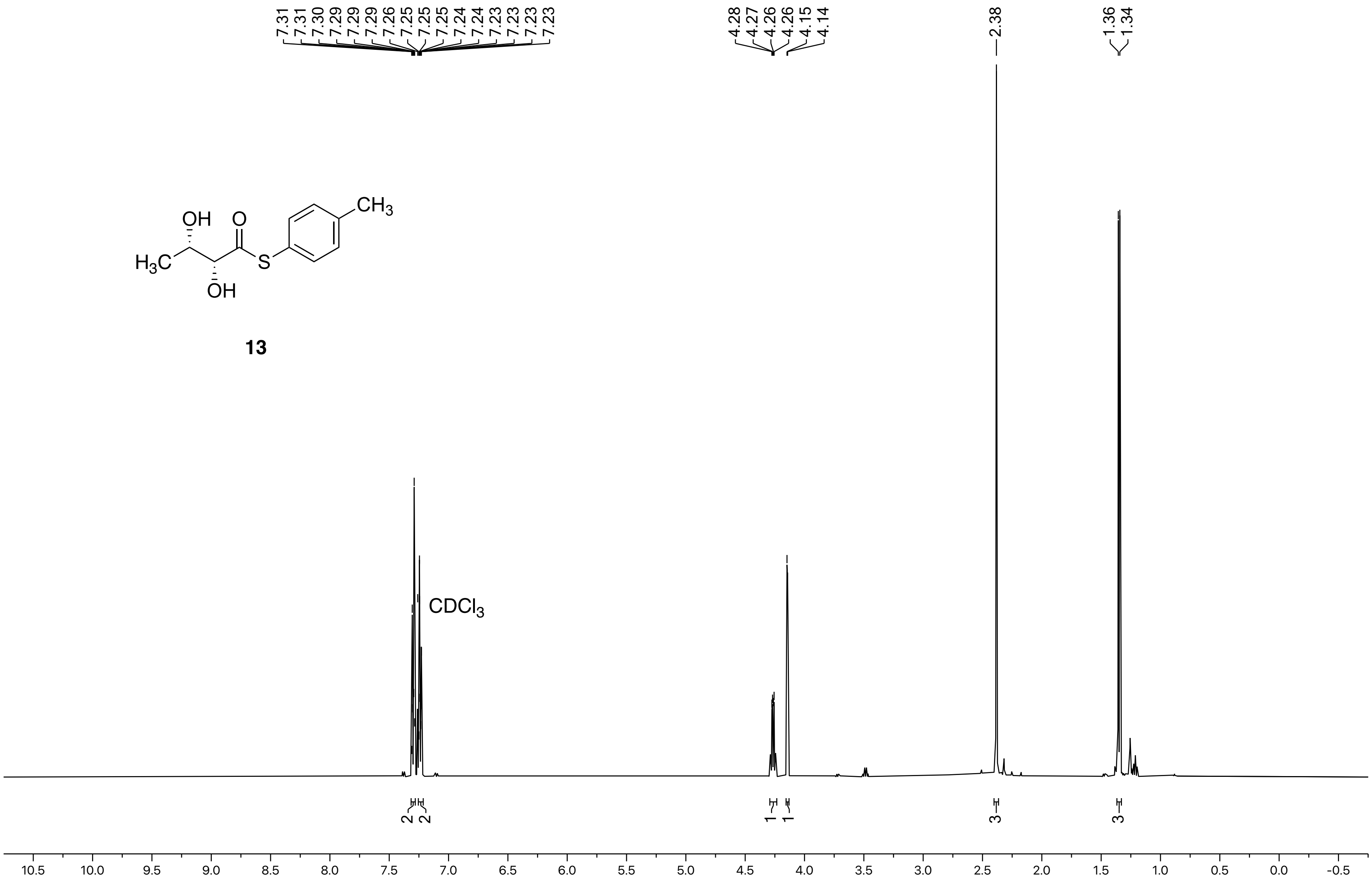

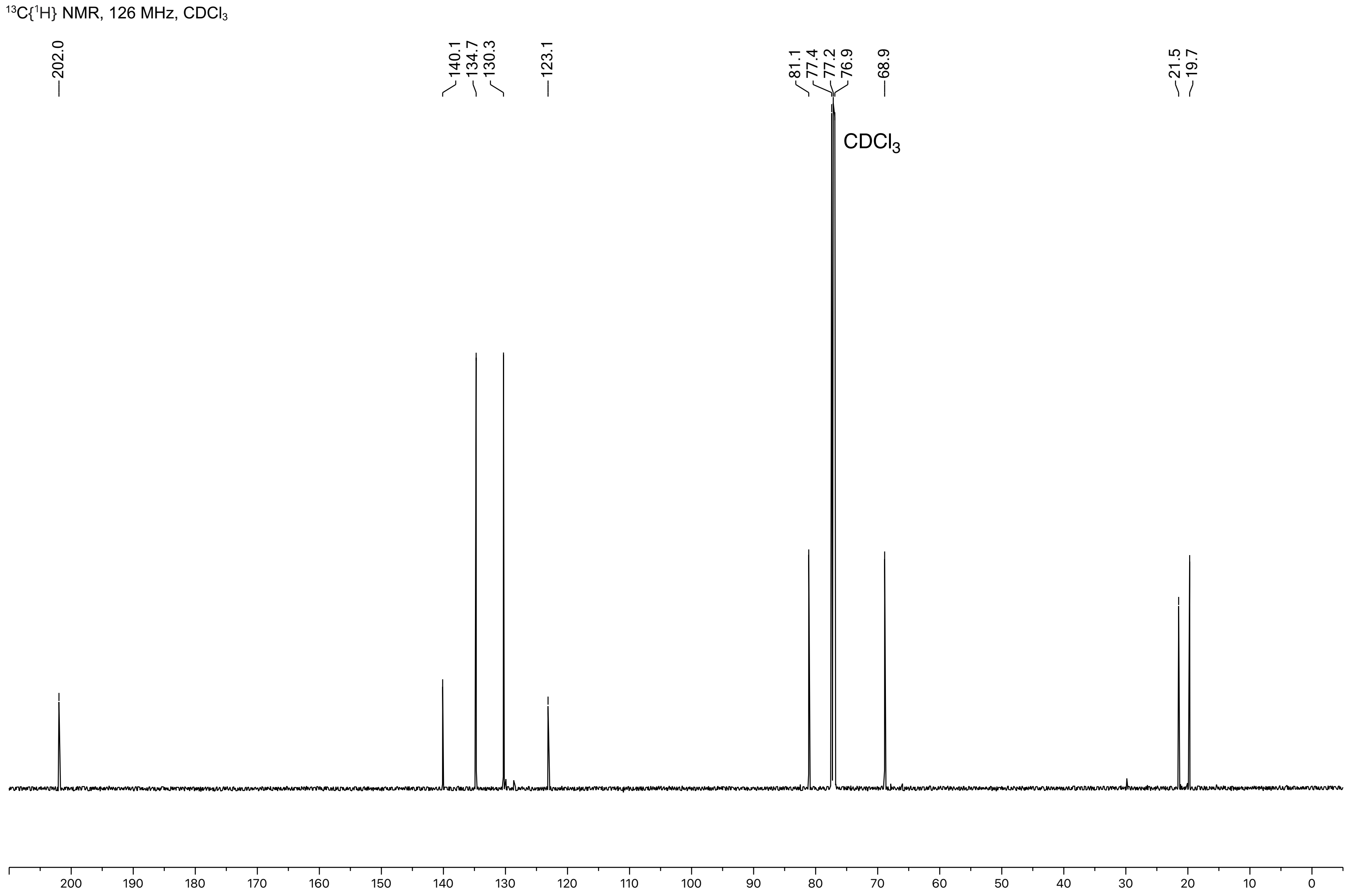

$^{13}C\{^1H\}$ NMR, 126 MHz, $CDCl_3$
202.0
140.1
134.7
130.3
123.1
81.1
77.4
77.2
76.9
68.9
21.5
19.7
$CDCl_3$
200 190 180 170 160 150 140 130 120 110 100 90 80 70 60 50 40 30 20 10 0

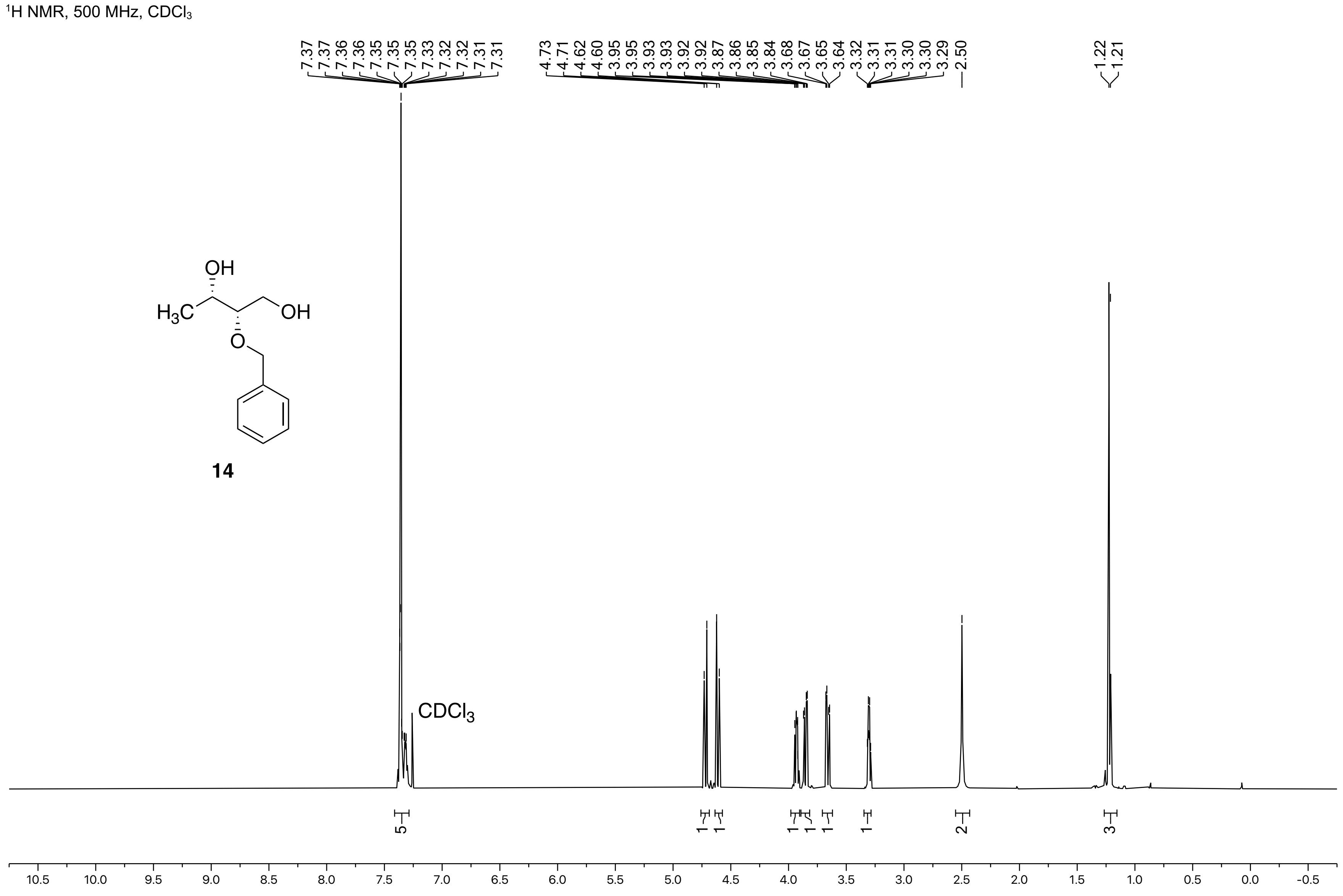

1H NMR, 500 MHz, CDCl3
7.37
7.37
7.36
7.36
7.35
7.35
7.35
7.33
7.32
7.32
7.31
7.31
4.73
4.71
4.62
4.60
3.95
3.95
3.93
3.93
3.92
3.92
3.87
3.86
3.85
3.84
3.68
3.67
3.65
3.64
3.32
3.31
3.31
3.30
3.30
3.29
2.50
1.22
1.21
OH
H3C
OH
O
14
CDCl3
5
1
1
1
1
1
1
2
3
10.5
10.0
9.5
9.0
8.5
8.0
7.5
7.0
6.5
6.0
5.5
5.0
4.5
4.0
3.5
3.0
2.5
2.0
1.5
1.0
0.5
0.0
-0.5

$^{13}C\{^1H\}$ NMR, 126 MHz, $CDCl_3$

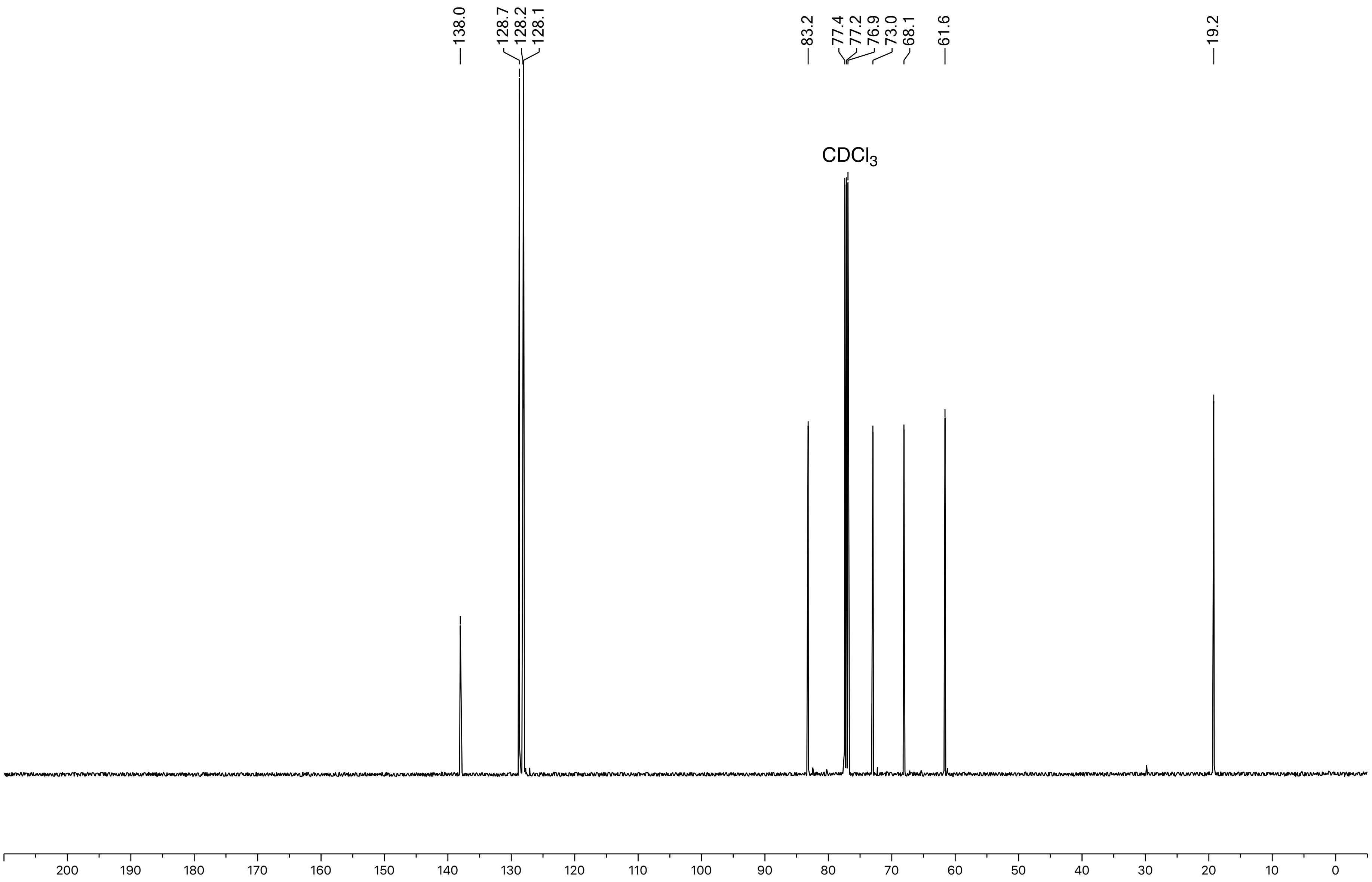

^{1}H NMR, 500 MHz, $CDCl_3$

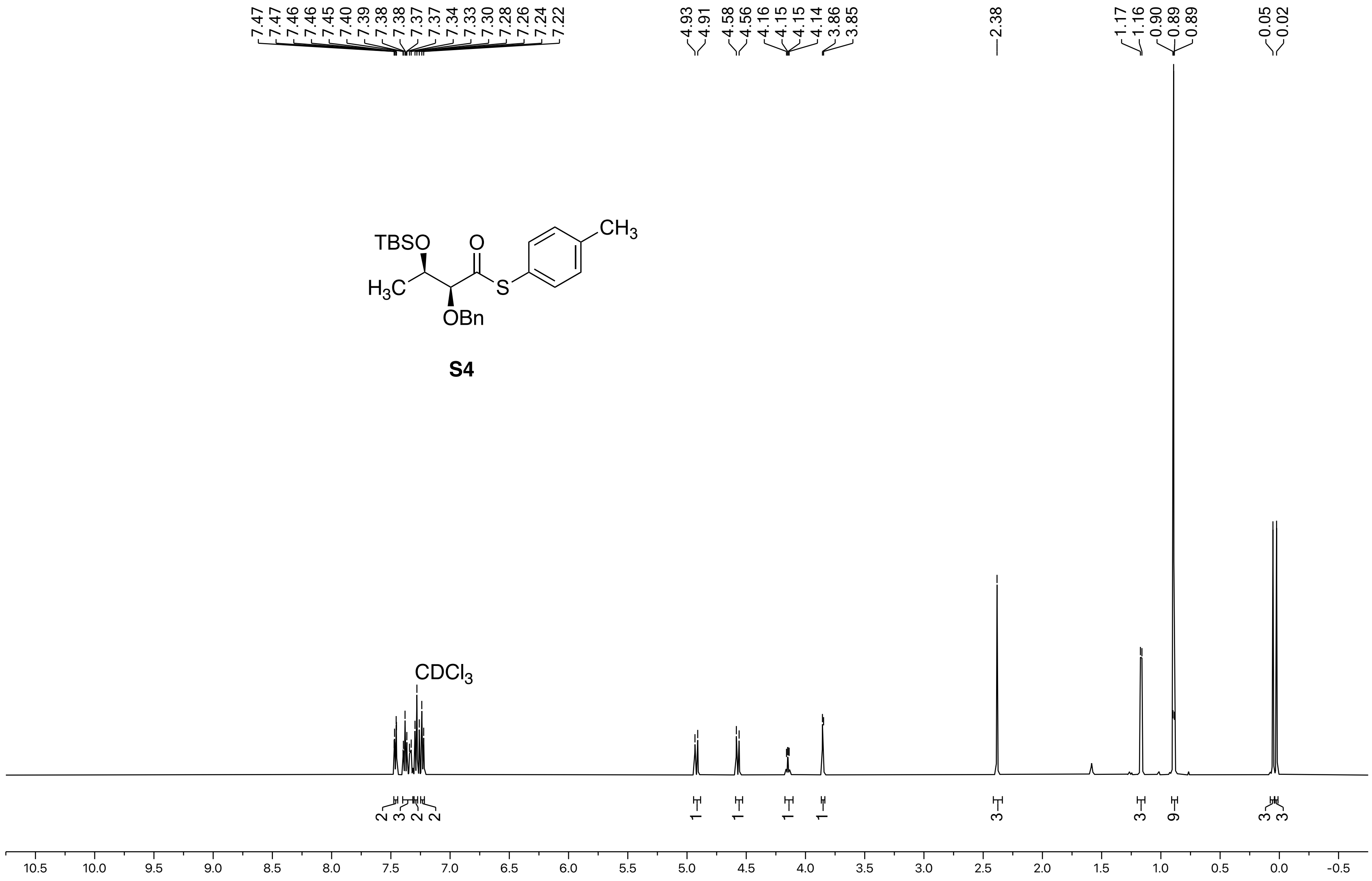

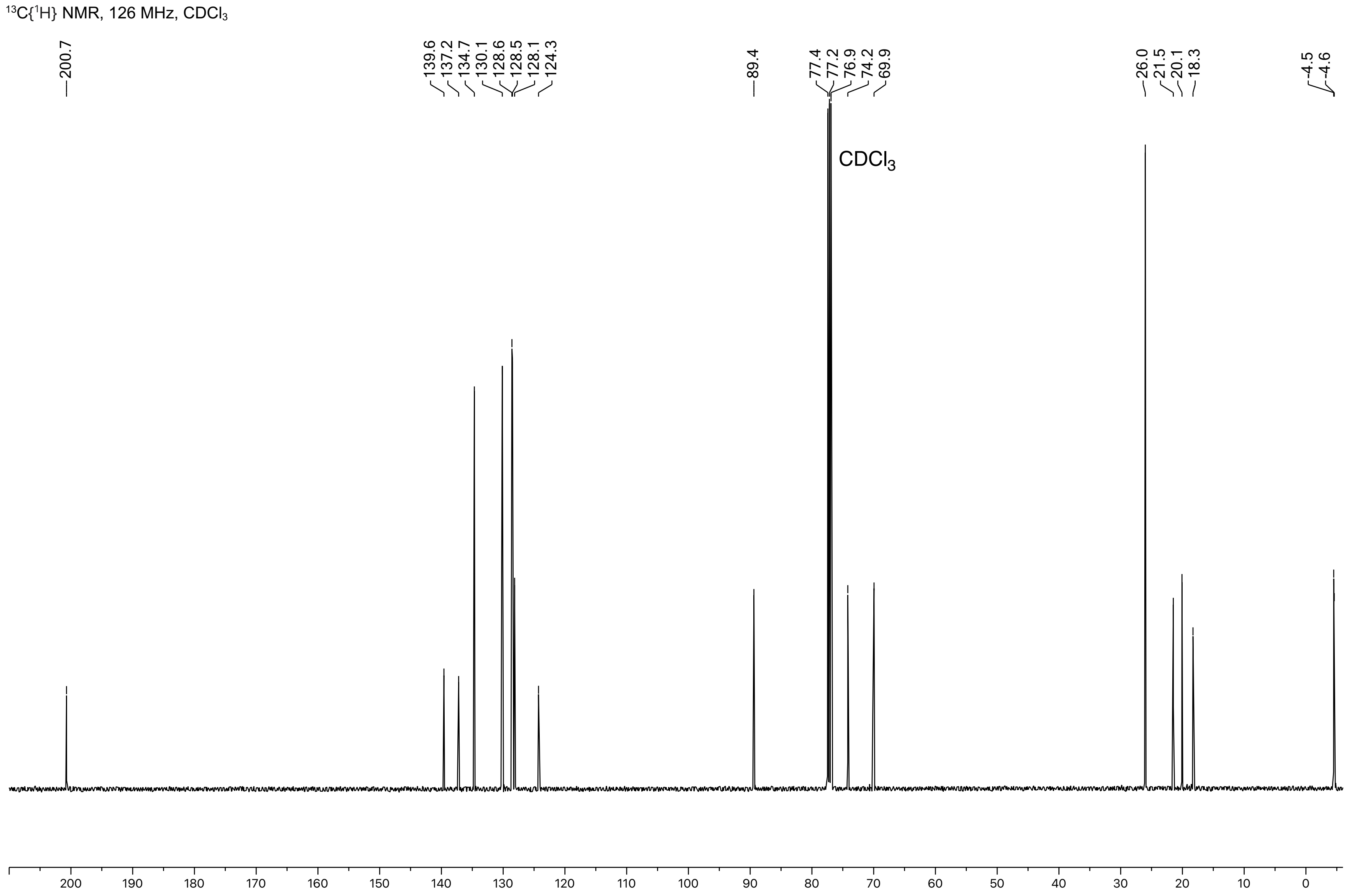

$^{13}C\{^1H\}$ NMR, 126 MHz, $CDCl_3$
200.7
139.6
137.2
134.7
130.1
128.6
128.5
128.1
124.3
89.4
77.4
77.2
76.9
74.2
69.9
26.0
21.5
20.1
18.3
-4.5
-4.6
$CDCl_3$
200
190
180
170
160
150
140
130
120
110
100
90
80
70
60
50
40
30
20
10
0

^{1}H NMR, 500 MHz, $CDCl_3$

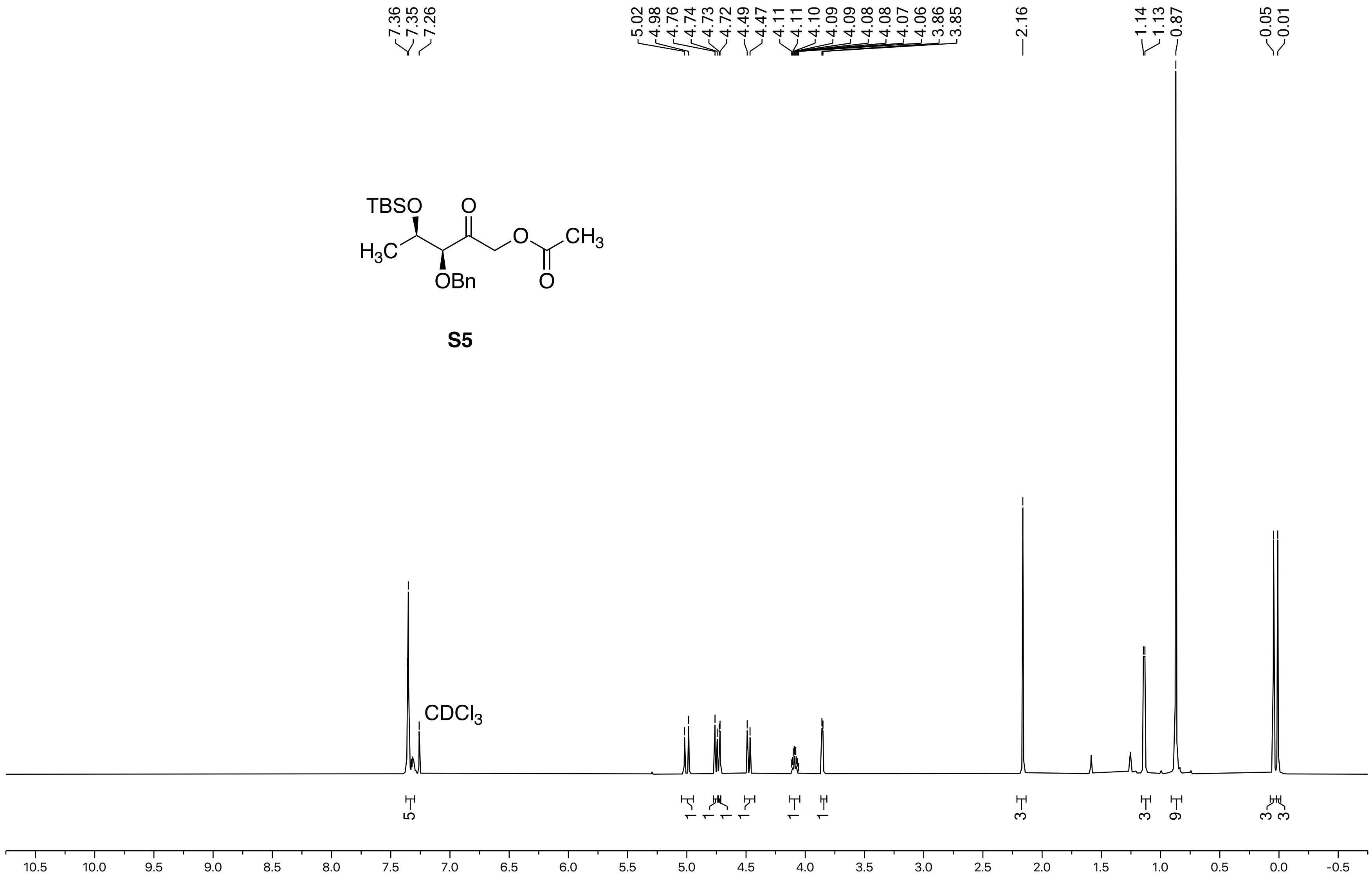

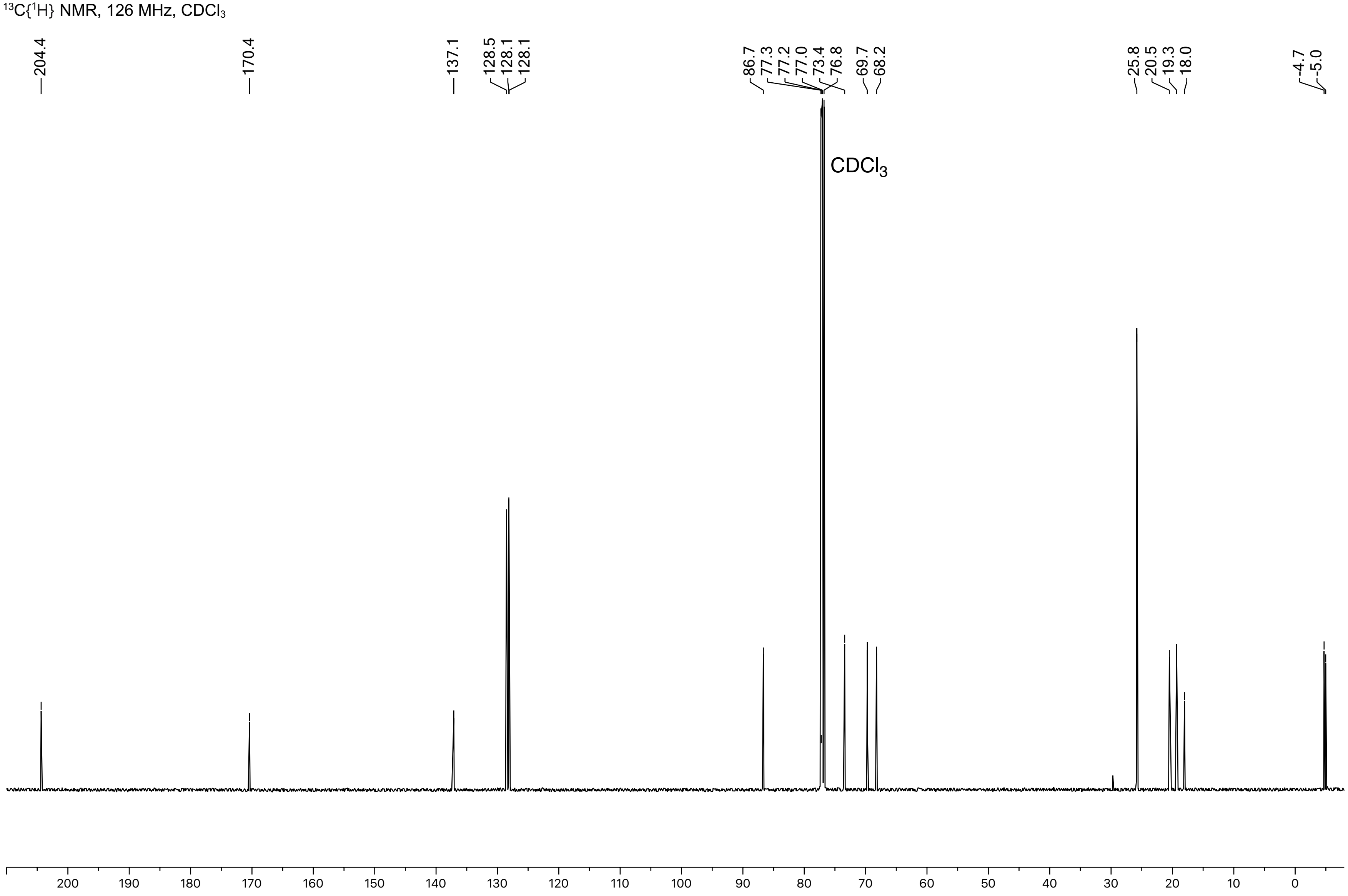

$^{13}C\{^1H\}$ NMR, 126 MHz, $CDCl_3$
204.4
170.4
137.1
128.5
128.1
128.1
86.7
77.3
77.2
77.0
73.4
76.8
69.7
68.2
25.8
20.5
19.3
18.0
-4.7
-5.0
$CDCl_3$
200
190
180
170
160
150
140
130
120
110
100
90
80
70
60
50
40
30
20
10
0

^{1}H NMR, 500 MHz, $CDCl_3$

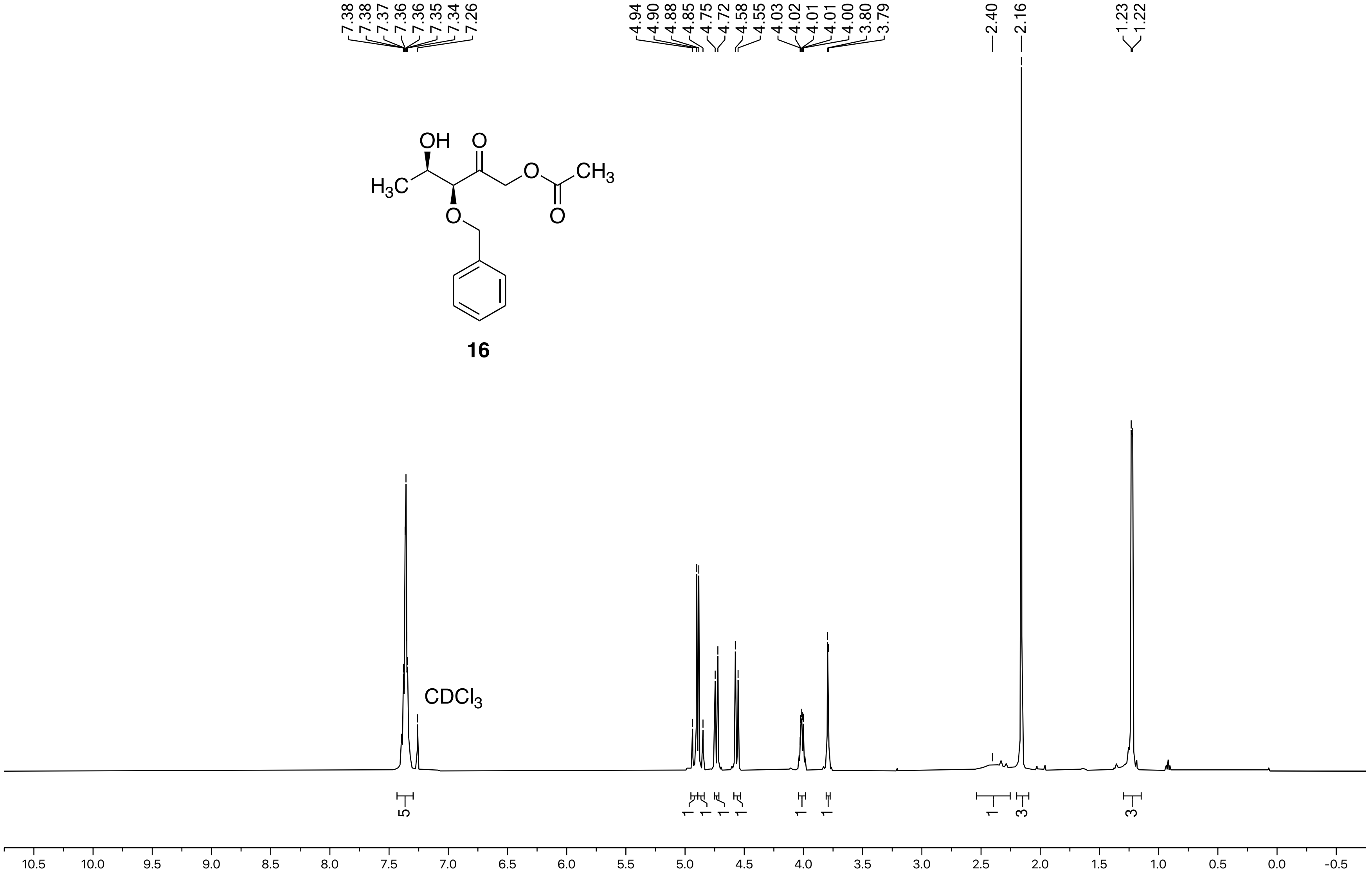

16

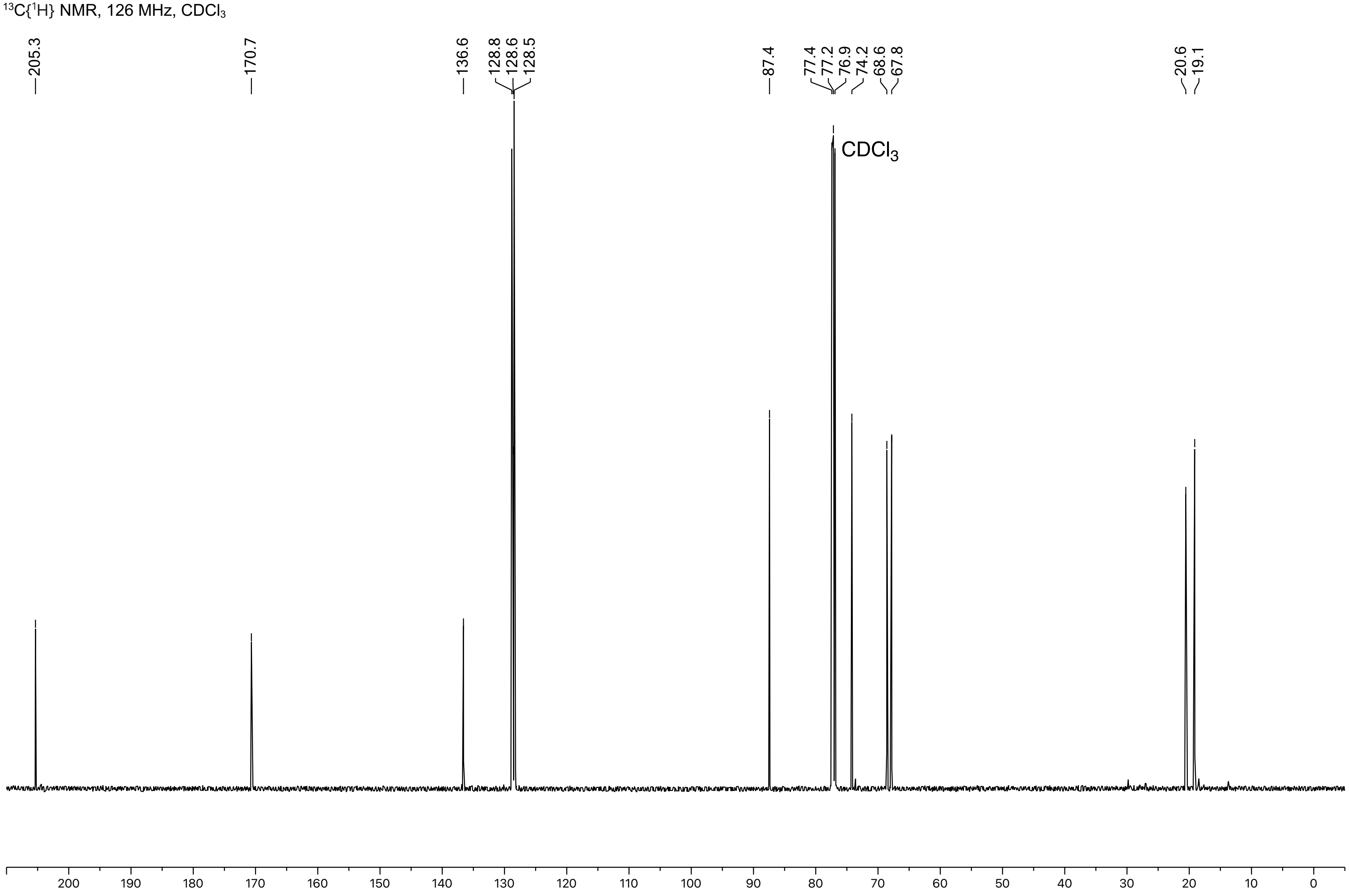

$^{13}C\{^1H\}$ NMR, 126 MHz, CDCl$_3$
205.3
170.7
136.6
128.8
128.6
128.5
87.4
77.4
77.2
76.9
74.2
68.6
67.8
20.6
19.1
CDCl$_3$
200
190
180
170
160
150
140
130
120
110
100
90
80
70
60
50
40
30
20
10
0

^{1}H NMR, 500 MHz, CDCl$_3$

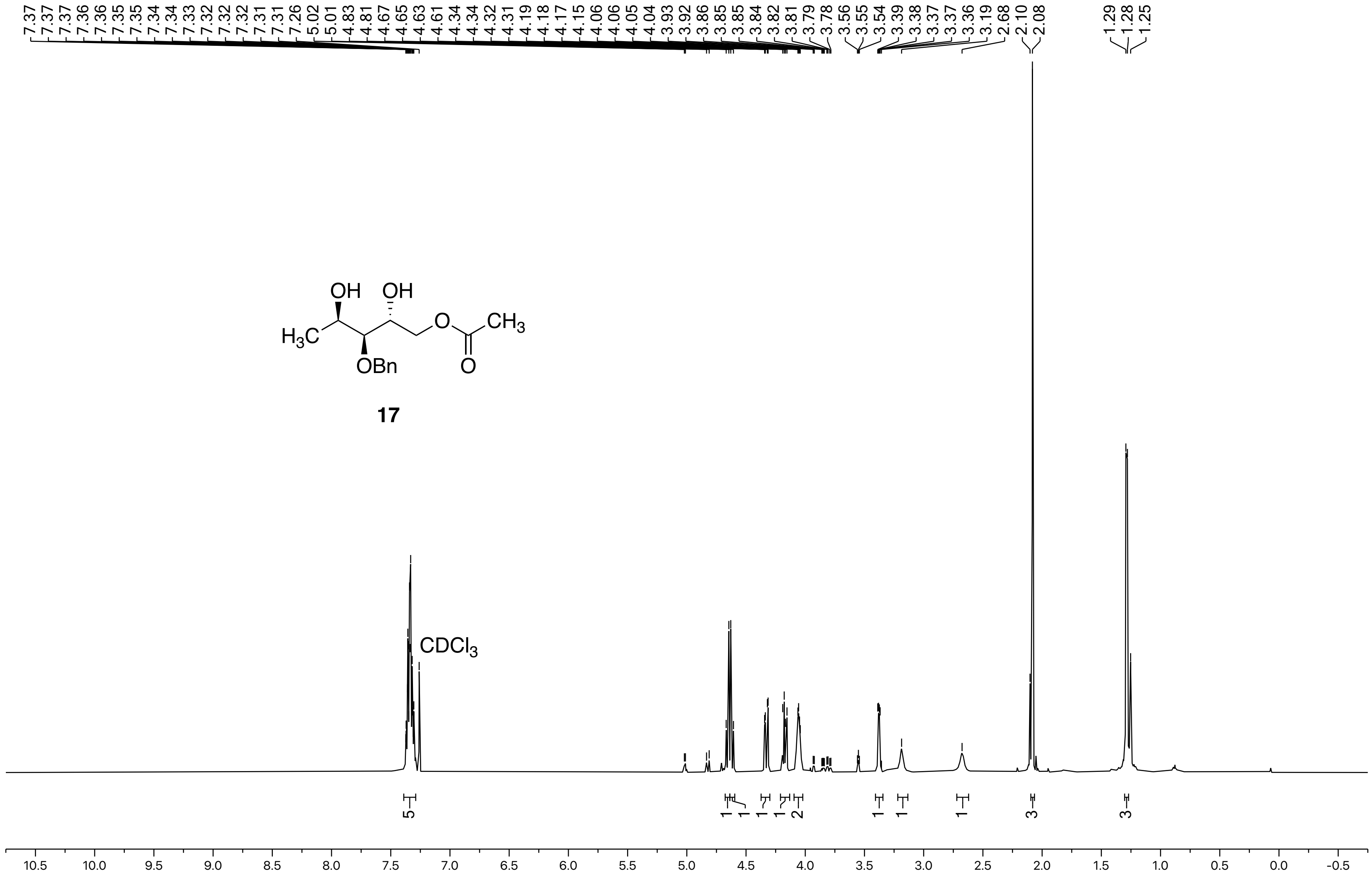

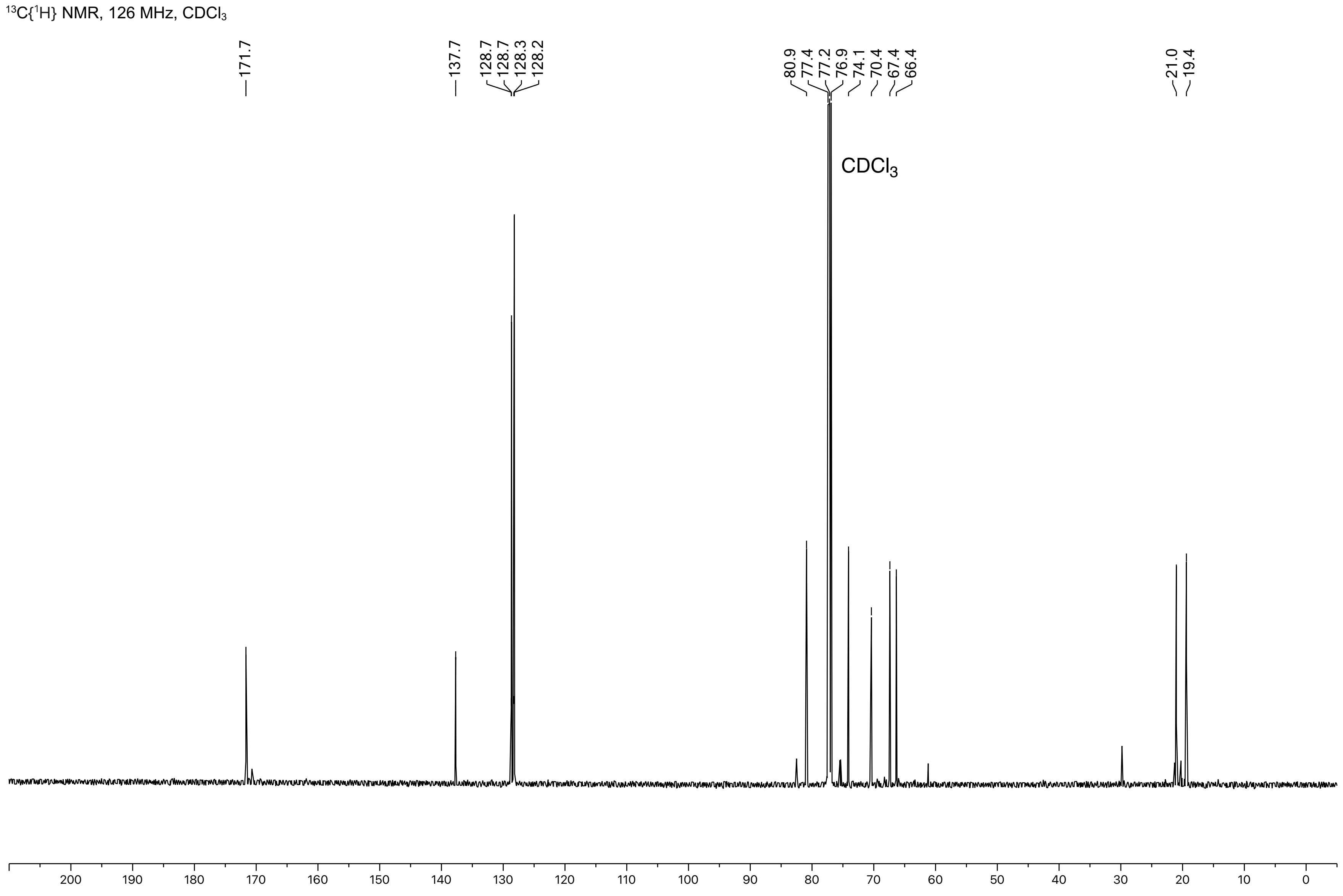

$^{13}C\{^1H\}$ NMR, 126 MHz, $CDCl_3$
171.7
137.7
128.7
128.7
128.3
128.2
80.9
77.4
77.2
76.9
74.1
70.4
67.4
66.4
21.0
19.4
$CDCl_3$
200 190 180 170 160 150 140 130 120 110 100 90 80 70 60 50 40 30 20 10 0

^{1}H NMR, 500 MHz, $CDCl_3$

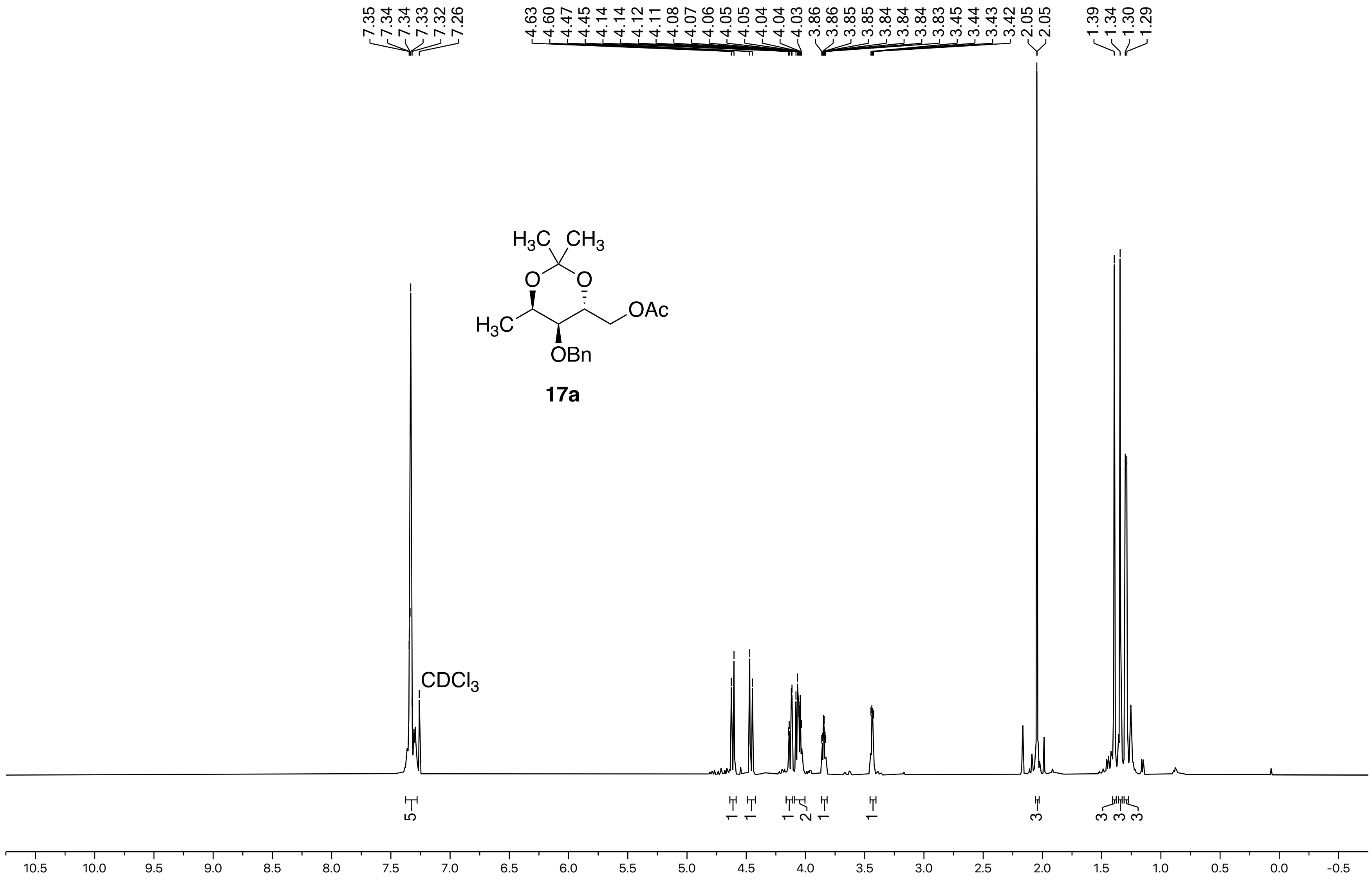

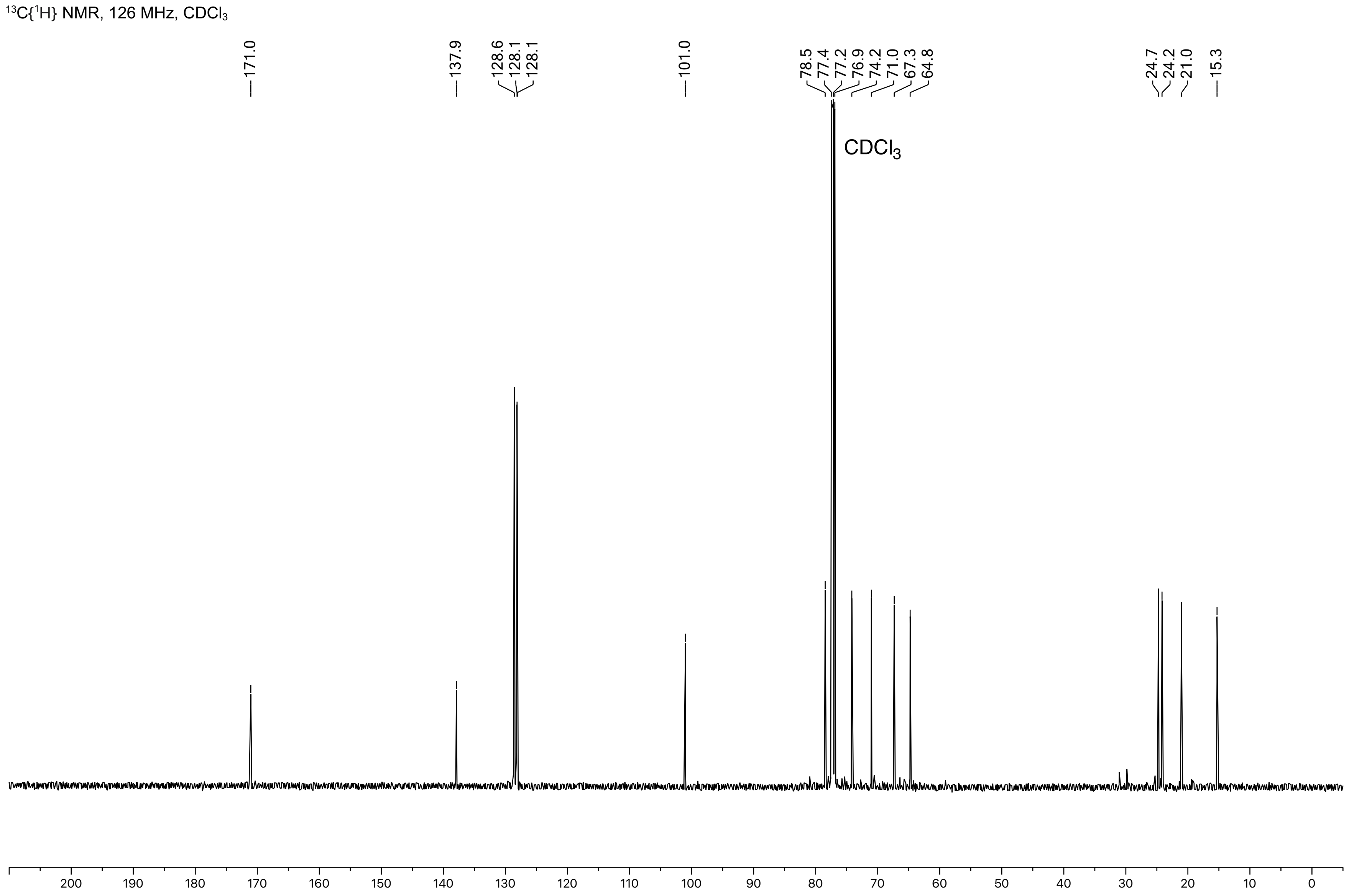

$^{13}C\{^{1}H\}$ NMR, 126 MHz, $CDCl_3$
171.0
137.9
128.6
128.1
128.1
101.0
78.5
77.4
77.2
76.9
74.2
71.0
67.3
64.8
24.7
24.2
21.0
15.3
$CDCl_3$
200 190 180 170 160 150 140 130 120 110 100 90 80 70 60 50 40 30 20 10 0

^1H NMR, 500 MHz, $CDCl_3$

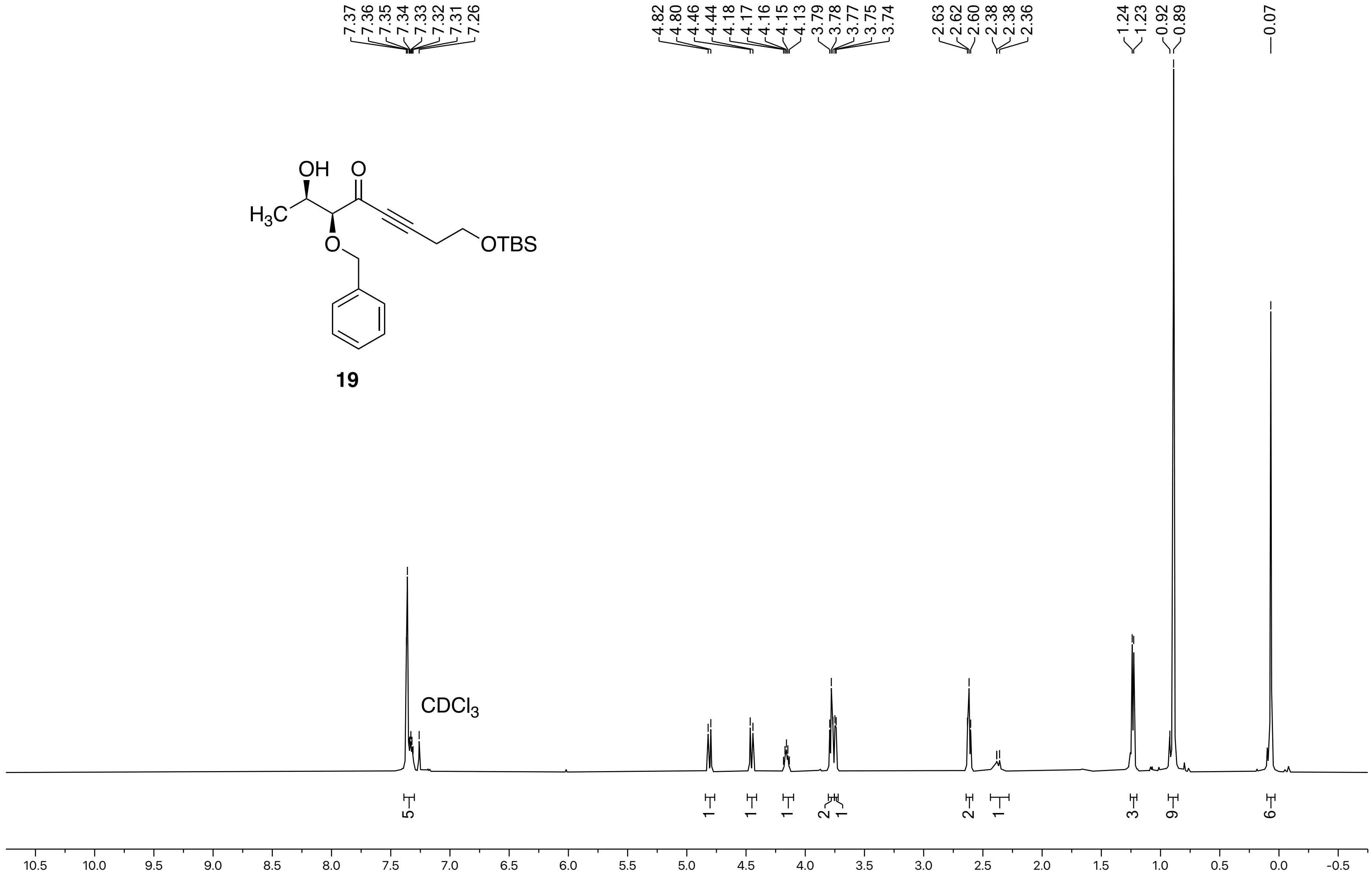

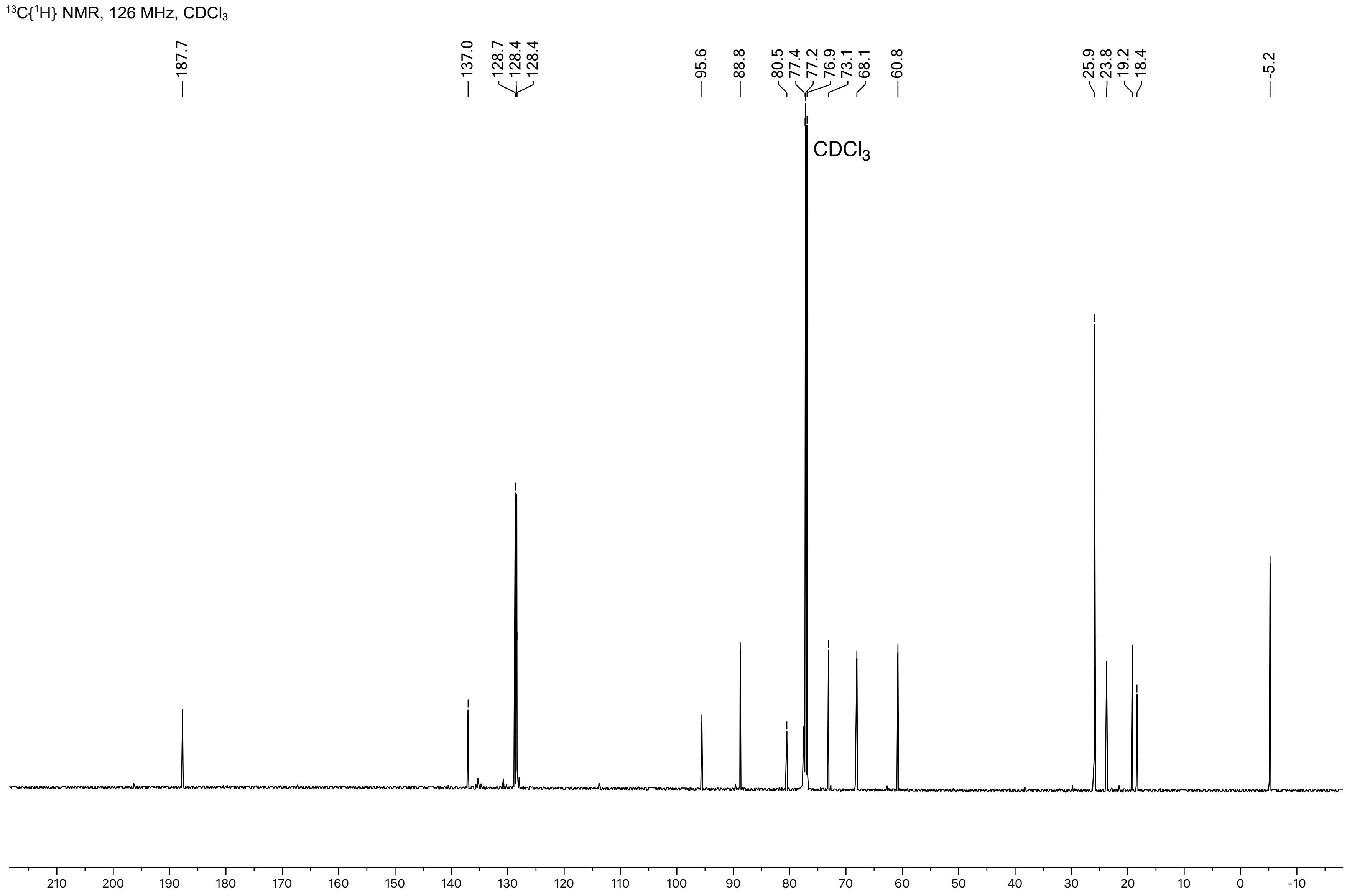

$^{13}C\{^1H\}$ NMR, 126 MHz, $CDCl_3$
187.7
137.0
128.7
128.4
128.4
95.6
88.8
80.5
77.4
77.2
76.9
73.1
68.1
60.8
25.9
23.8
19.2
18.4
-5.2
$CDCl_3$
210
200
190
180
170
160
150
140
130
120
110
100
90
80
70
60
50
40
30
20
10
0
-10

^{1}H NMR, 500 MHz, $CDCl_3$

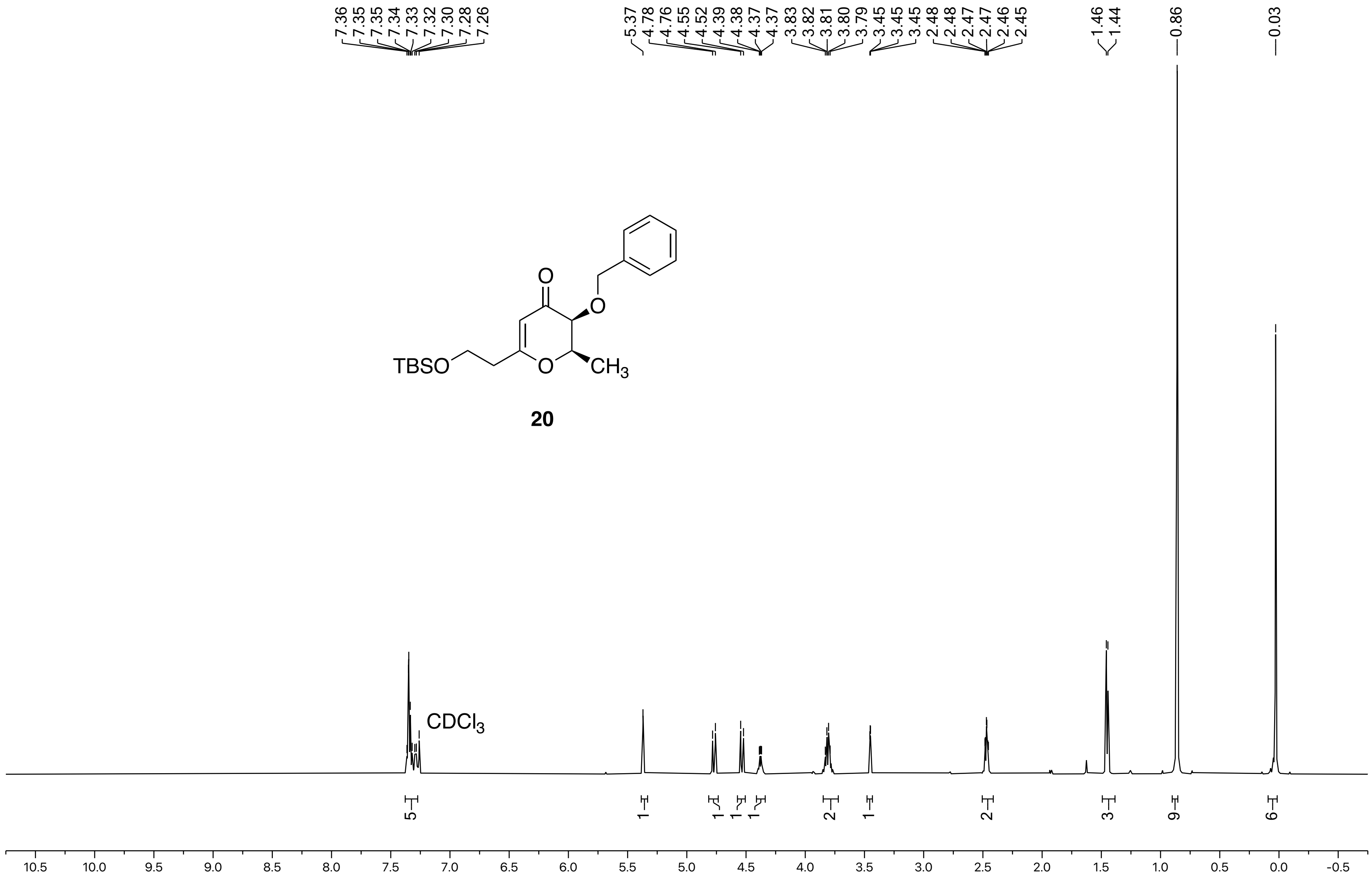

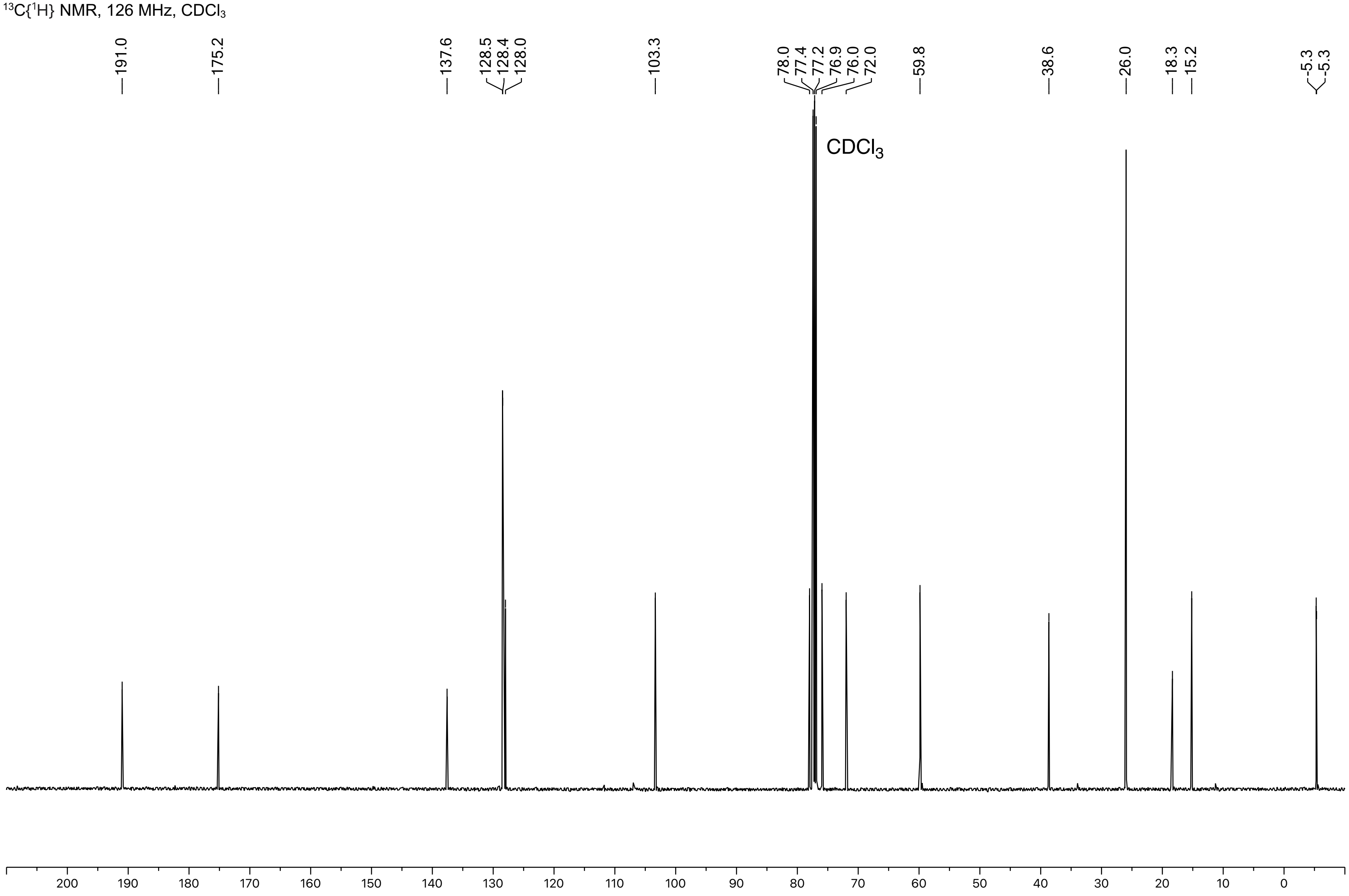
$^{13}C\{^{1}H\}$ NMR, 126 MHz, $CDCl_3$
191.0
175.2
137.6
128.5
128.4
128.0
103.3
78.0
77.4
77.2
76.9
76.0
72.0
59.8
38.6
26.0
18.3
15.2
-5.3
-5.3
$CDCl_3$
200
190
180
170
160
150
140
130
120
110
100
90
80
70
60
50
40
30
20
10
0

^{1}H NMR, 500 MHz, CDCl$_3$

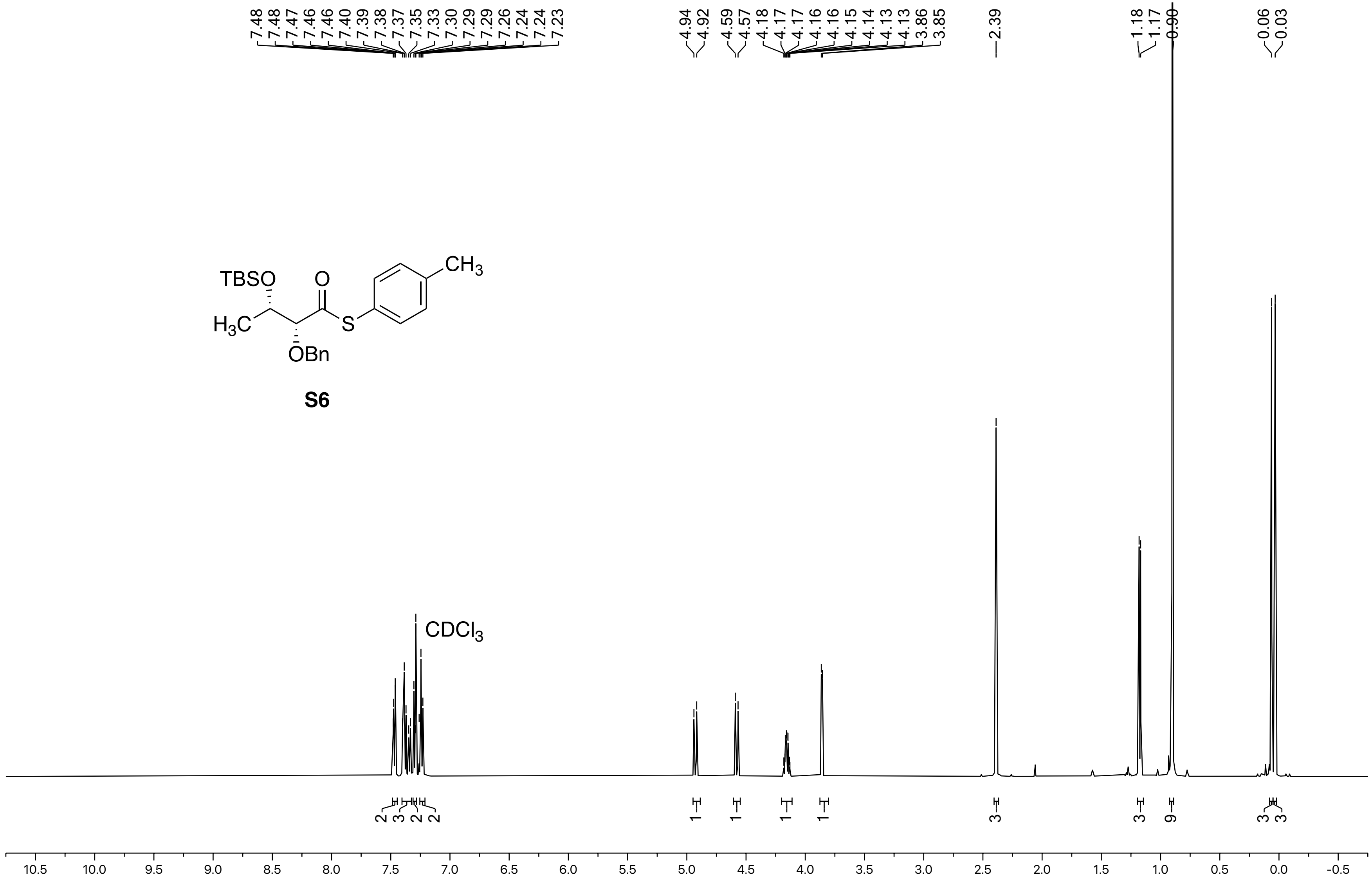

S6

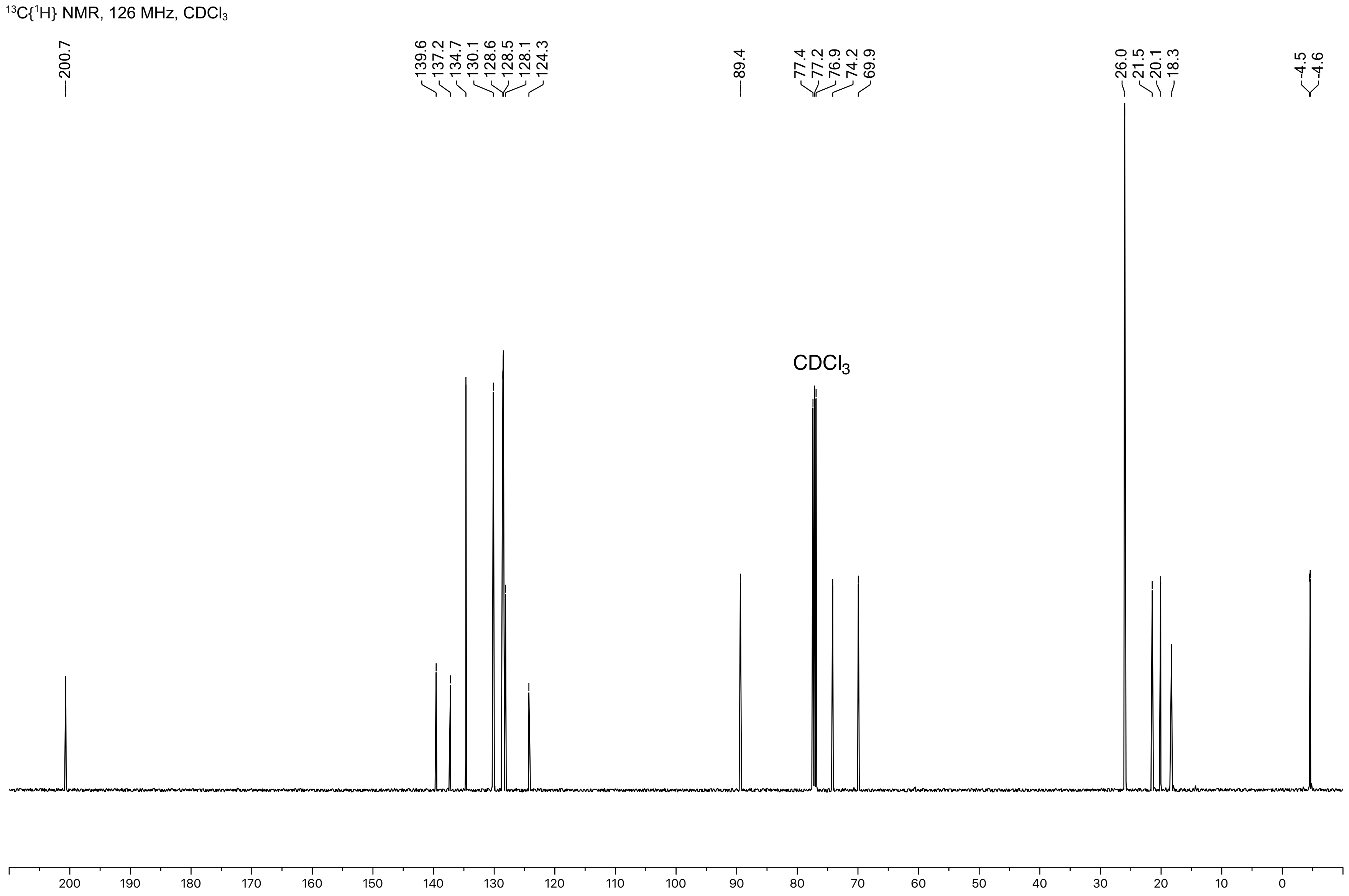

$^{13}C\{^1H\}$ NMR, 126 MHz, $CDCl_3$
200.7
139.6
137.2
134.7
130.1
128.6
128.5
128.1
124.3
89.4
77.4
77.2
76.9
74.2
69.9
26.0
21.5
20.1
18.3
-4.5
-4.6
$CDCl_3$
200 190 180 170 160 150 140 130 120 110 100 90 80 70 60 50 40 30 20 10 0

^{1}H NMR, 500 MHz, $CDCl_3$

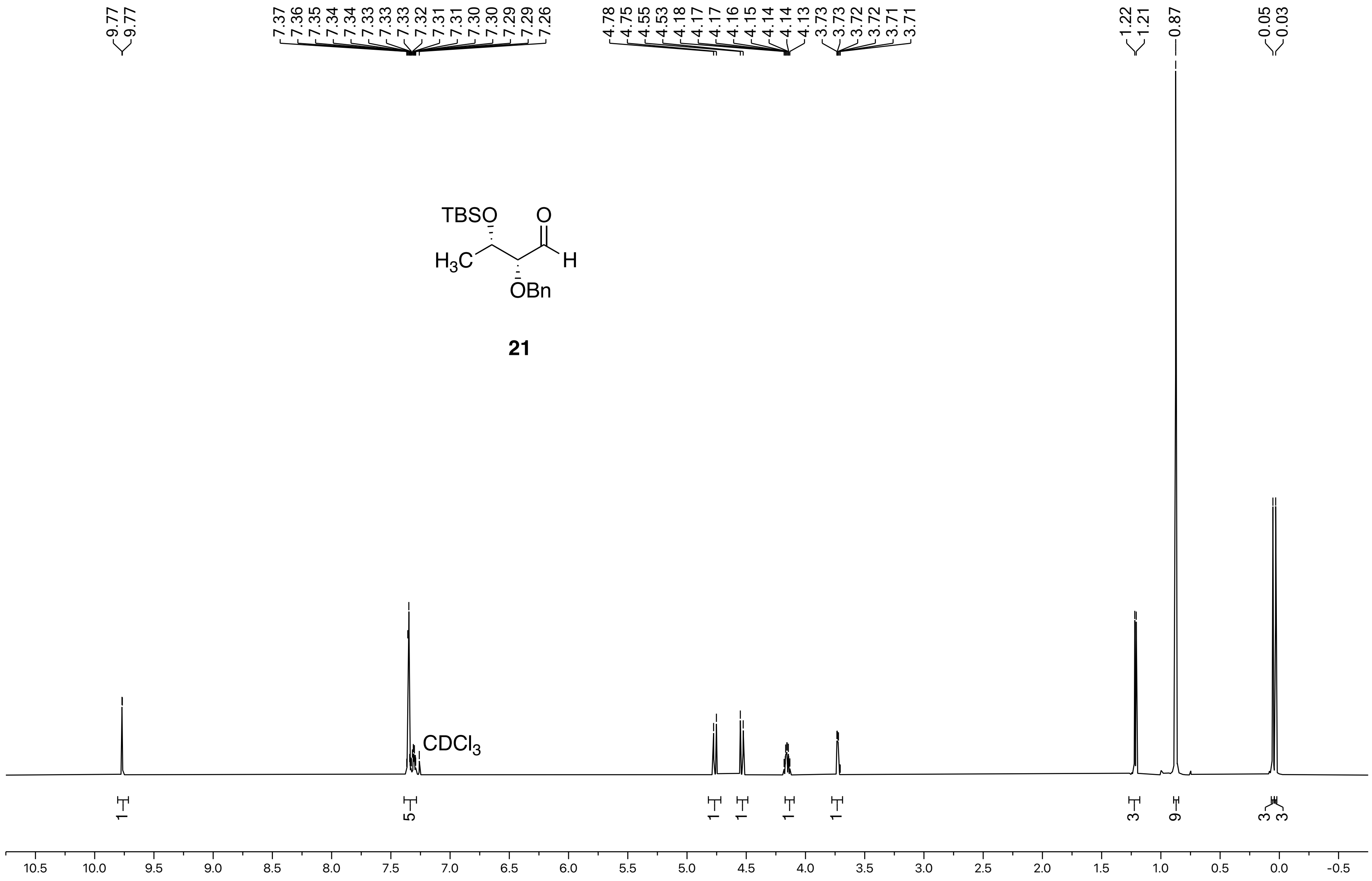

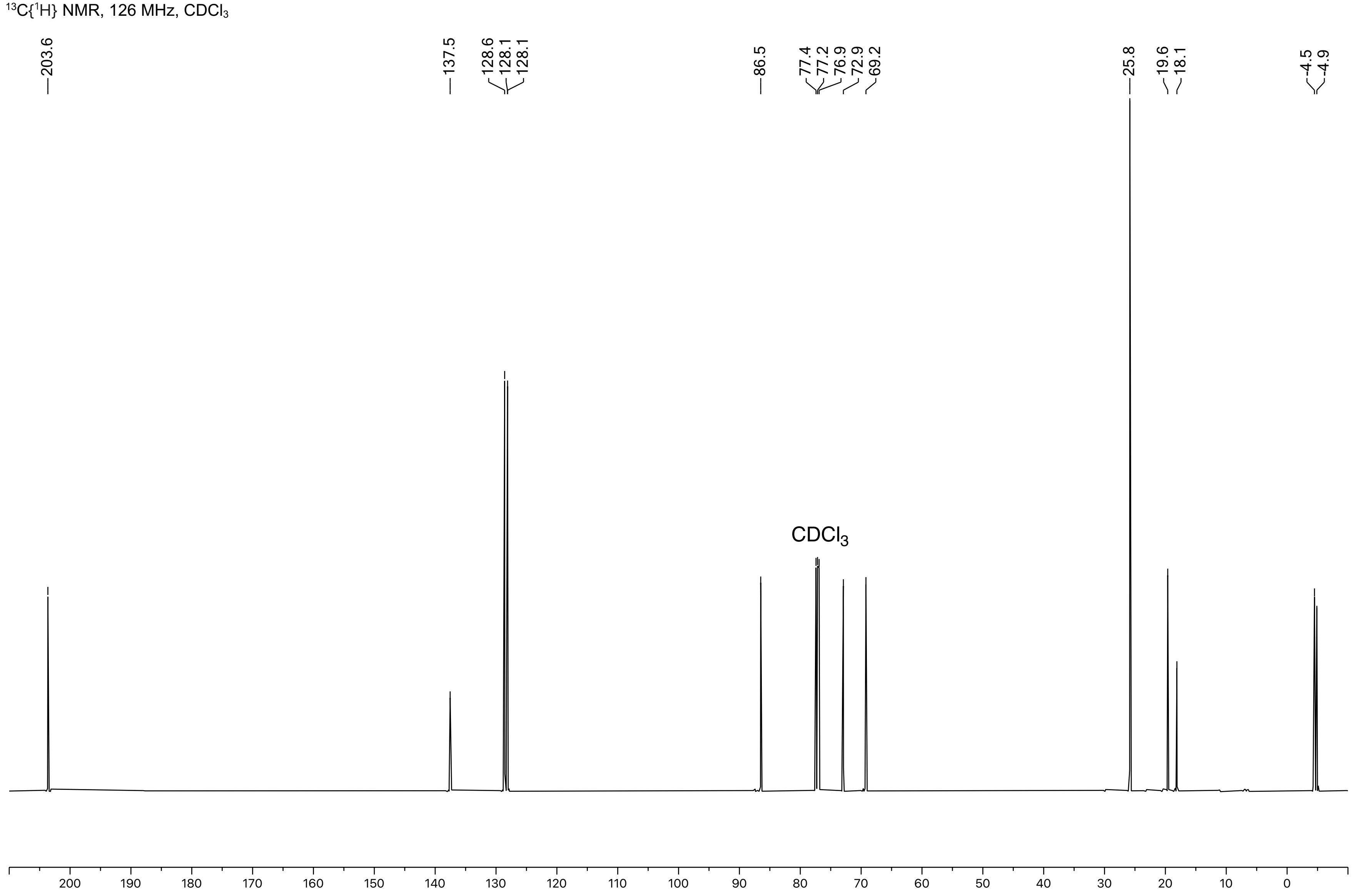

13C{1H} NMR, 126 MHz, CDCl3
203.6
137.5
128.6
128.1
128.1
86.5
77.4
77.2
76.9
72.9
69.2
25.8
19.6
18.1
-4.5
-4.9
CDCl3
200
190
180
170
160
150
140
130
120
110
100
90
80
70
60
50
40
30
20
10
0

^{1}H NMR, 500 MHz, $CDCl_3$

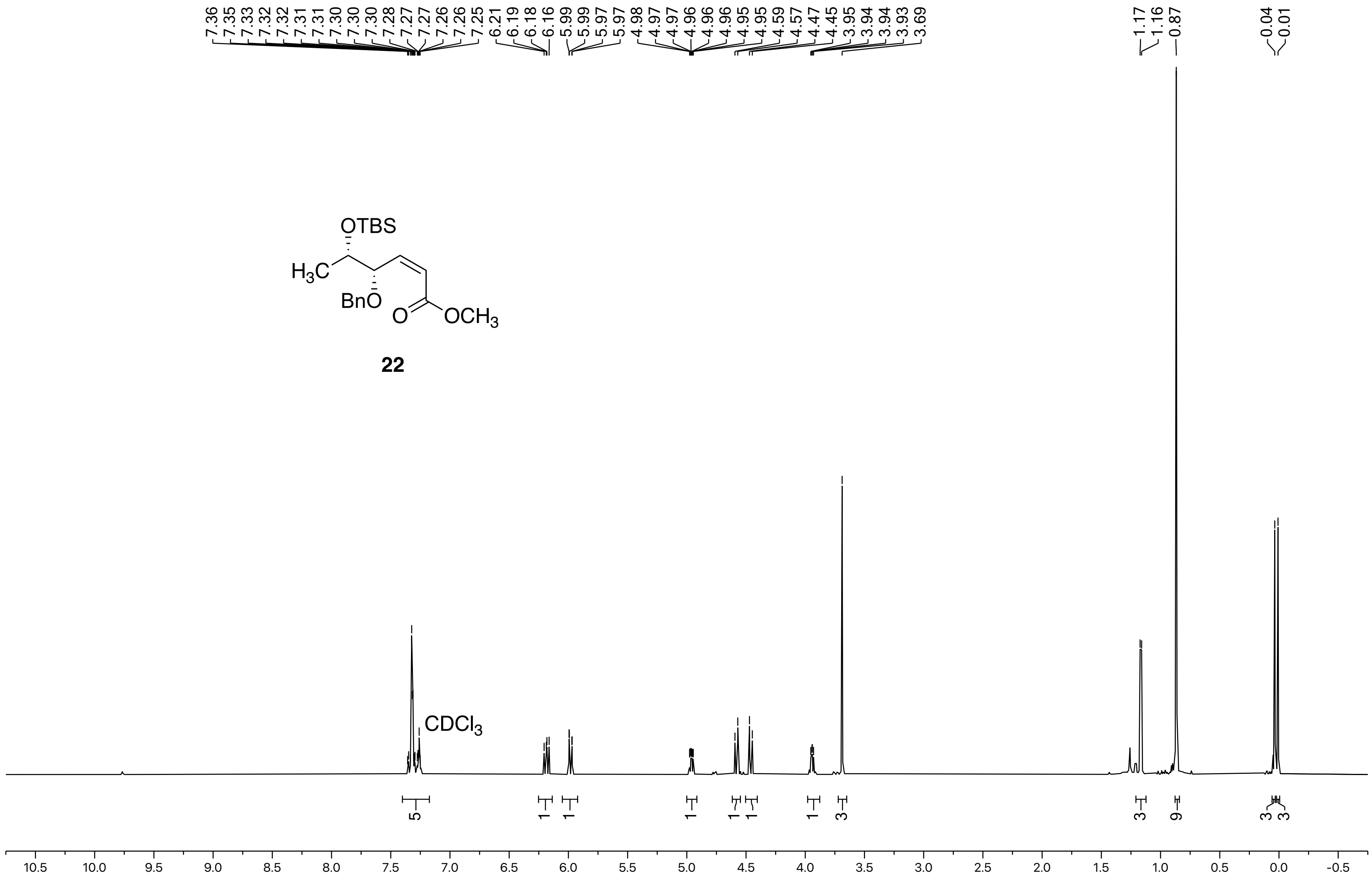

$^{13}C\{^{1}H\}$ NMR, 126 MHz, $CDCl_3$

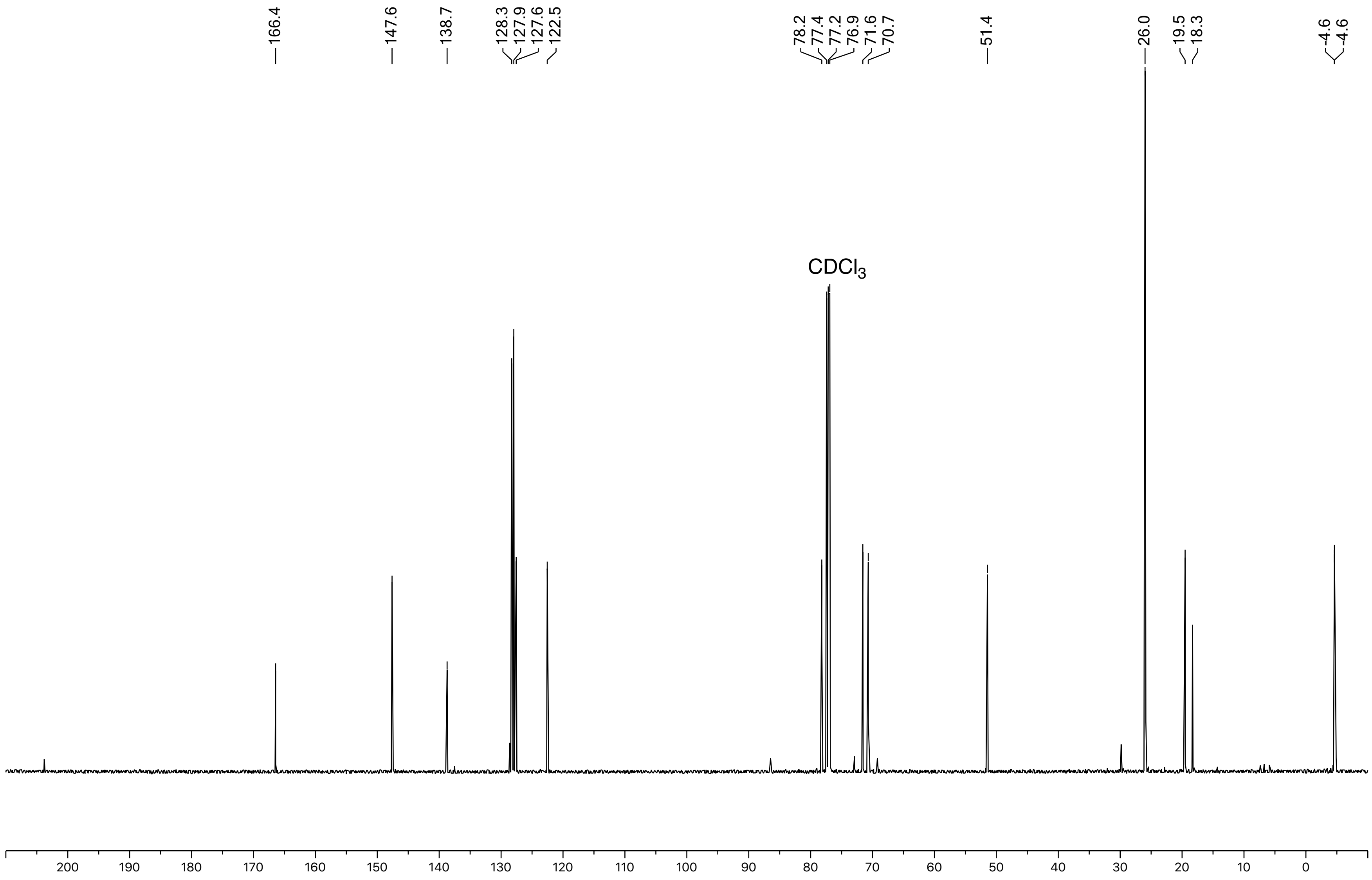

^{1}H NMR, 500 MHz, $CDCl_3$

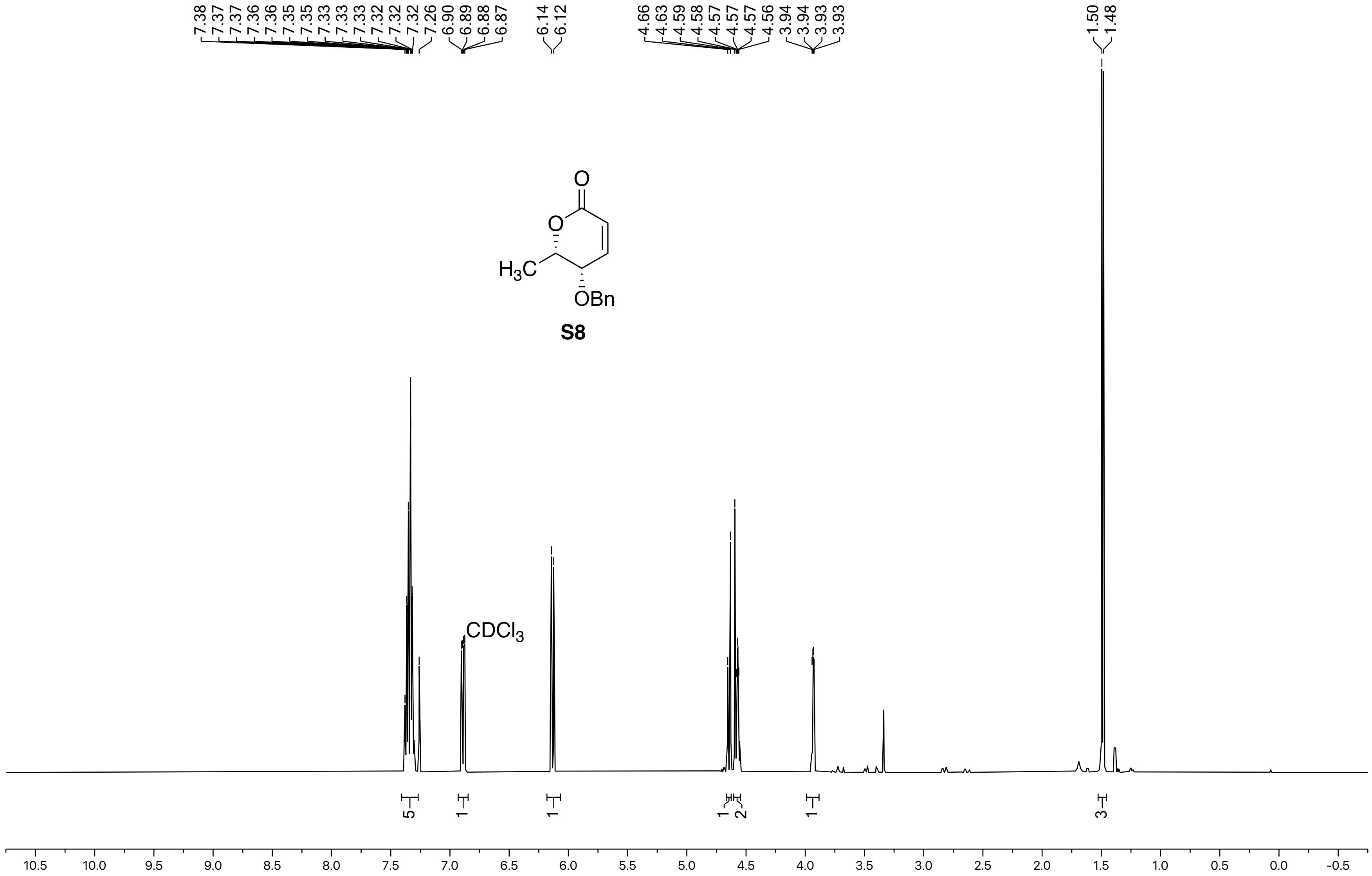

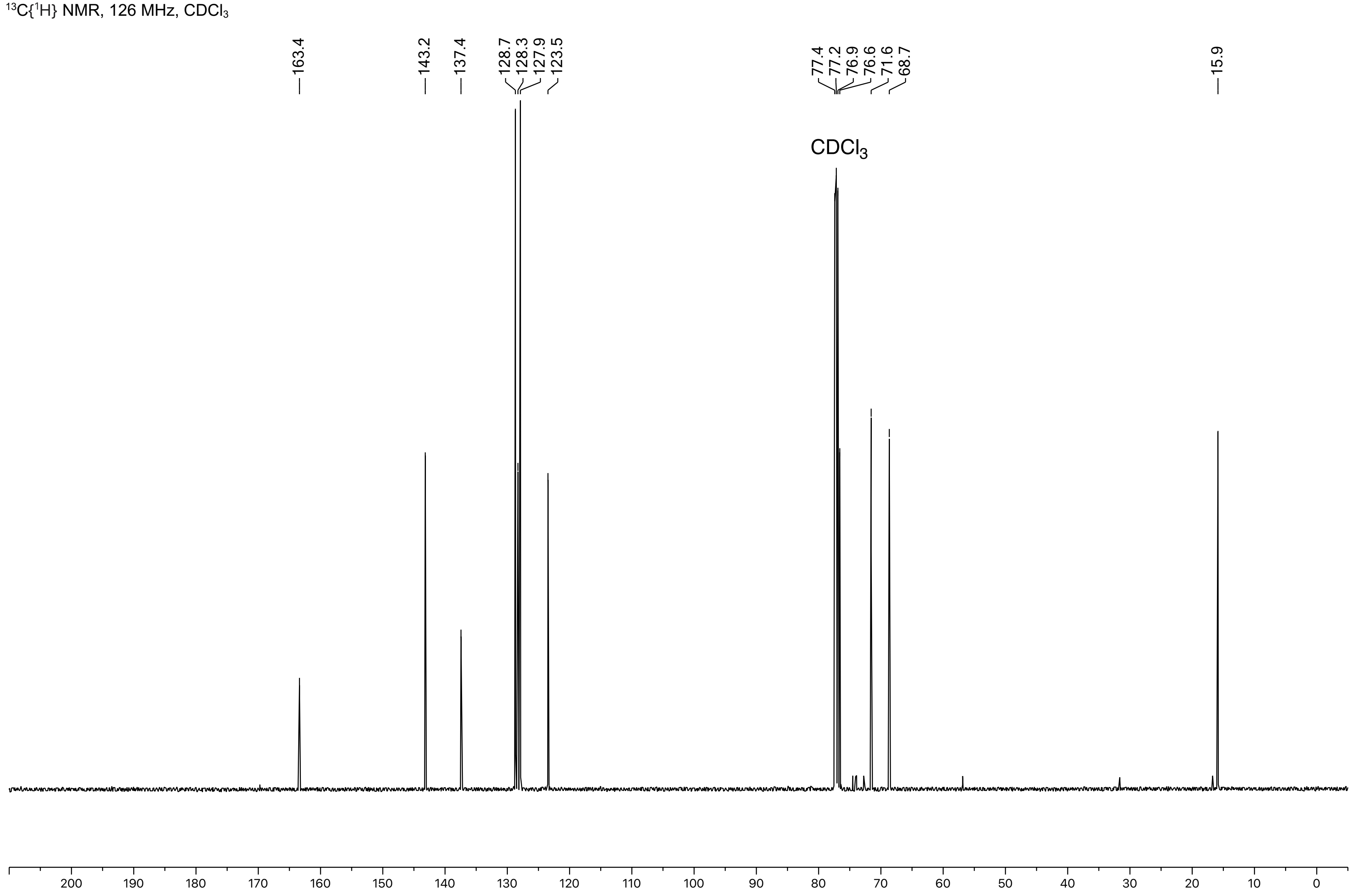

$^{13}C\{^{1}H\}$ NMR, 126 MHz, $CDCl_3$
163.4
143.2
137.4
128.7
128.3
127.9
123.5
77.4
77.2
76.9
76.6
71.6
68.7
15.9
$CDCl_3$
200
190
180
170
160
150
140
130
120
110
100
90
80
70
60
50
40
30
20
10
0

^1H NMR, 500 MHz, $CDCl_3$

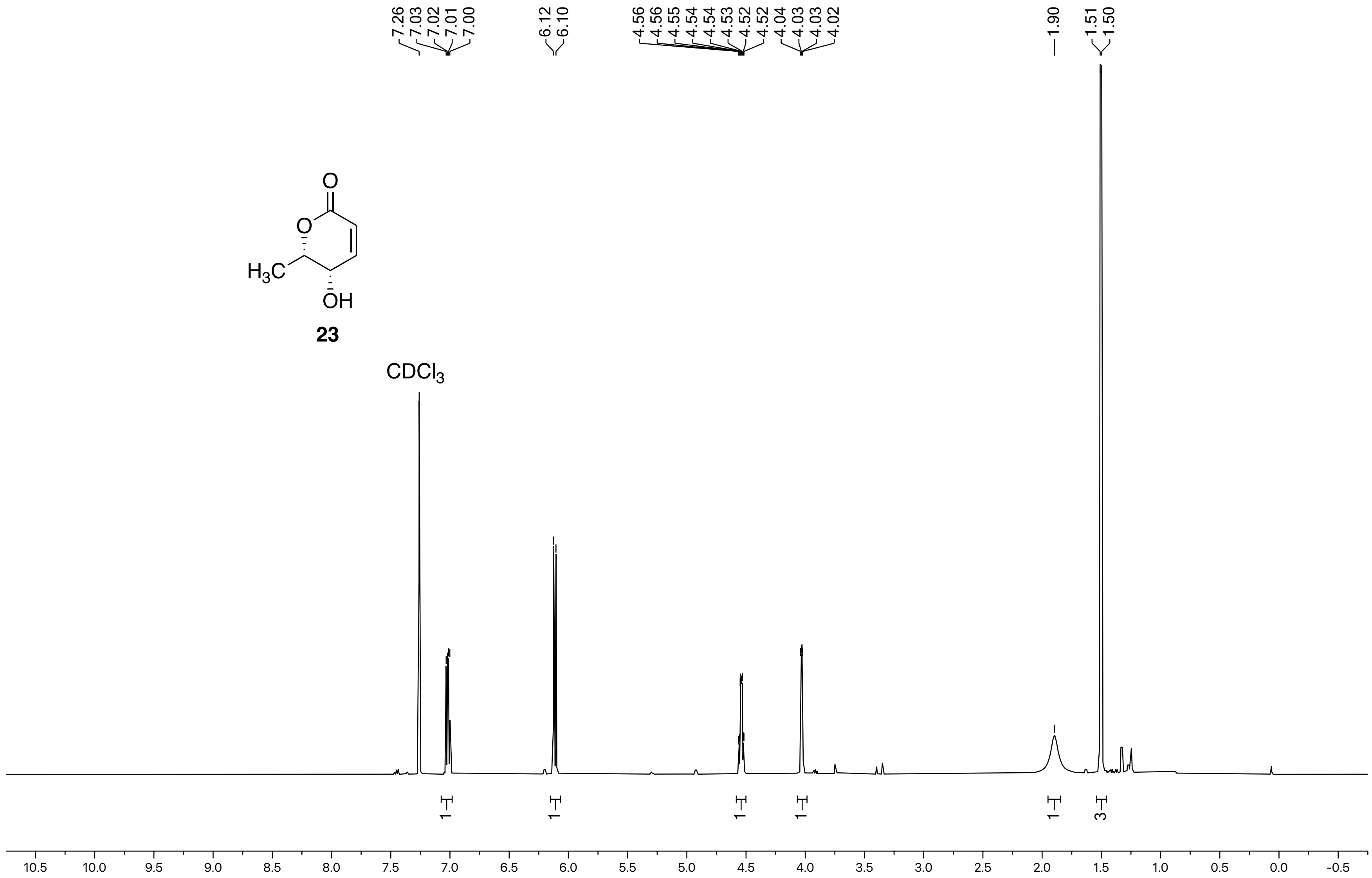

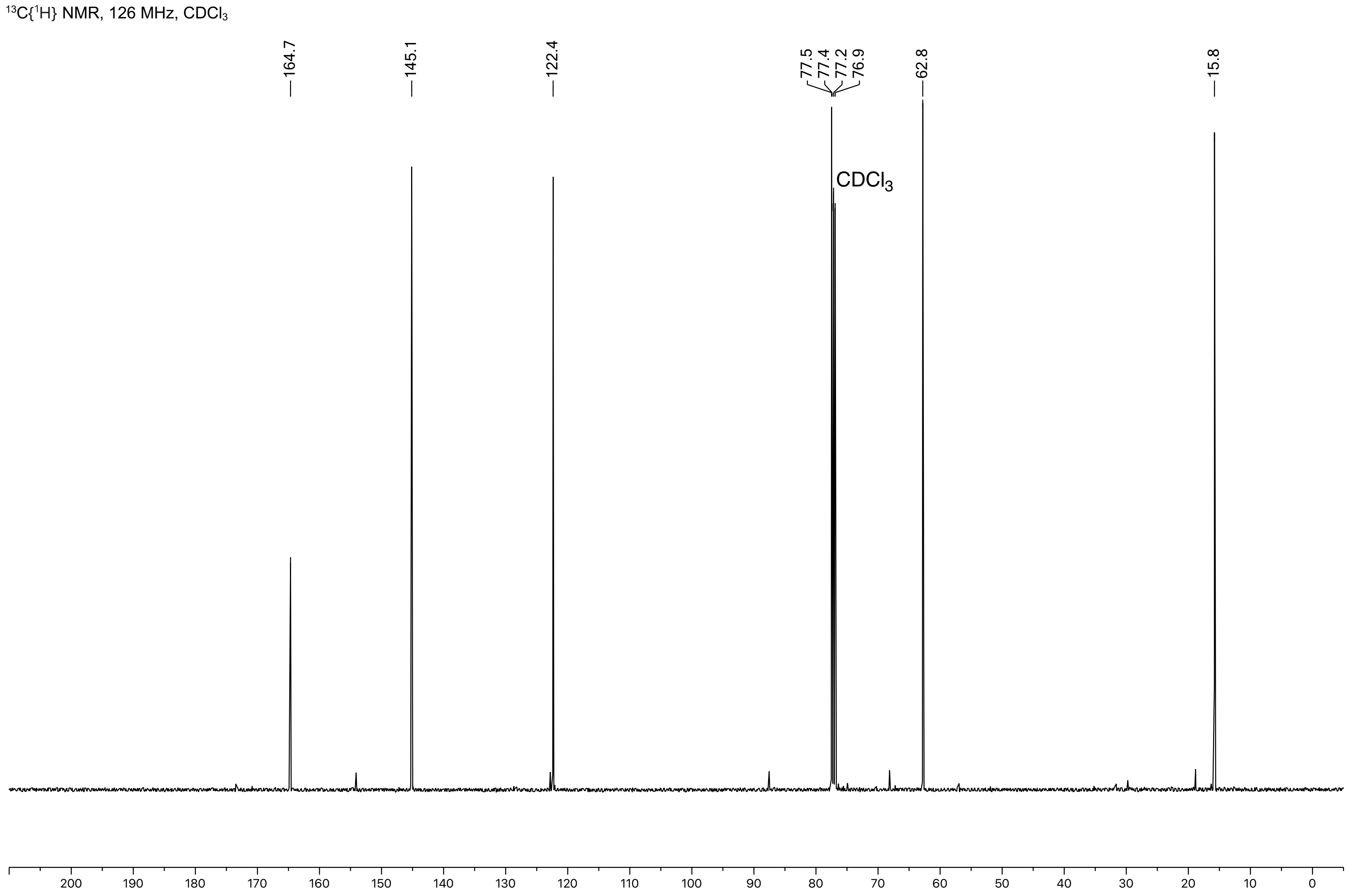

$^{13}C\{^1H\}$ NMR, 126 MHz, $CDCl_3$
164.7
145.1
122.4
77.5
77.4
77.2
76.9
62.8
15.8
$CDCl_3$
200
190
180
170
160
150
140
130
120
110
100
90
80
70
60
50
40
30
20
10
0

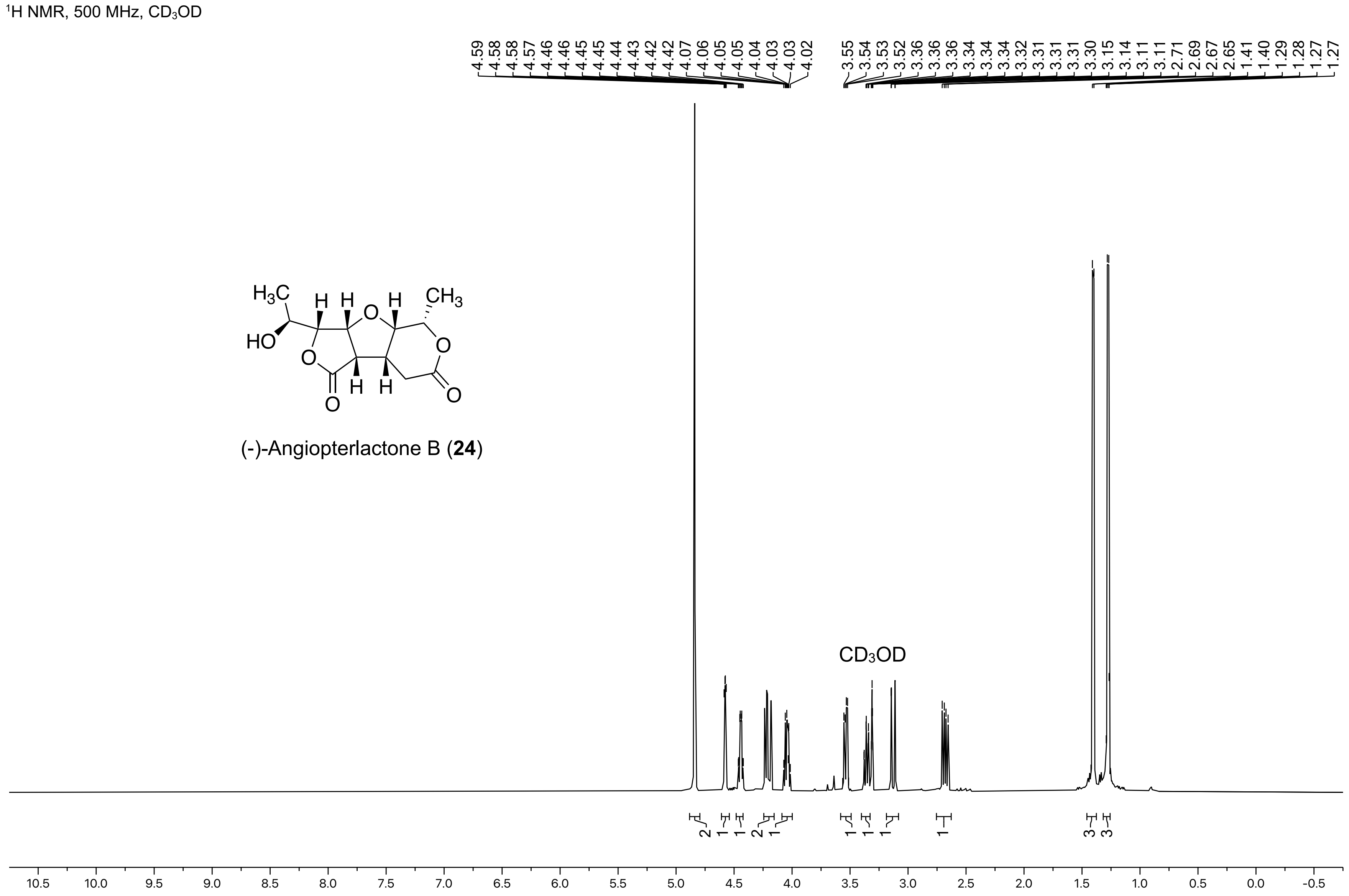

^{1}H NMR, 500 MHz, CD_3OD
4.59 4.58 4.58 4.57 4.46 4.46 4.45 4.45 4.44 4.43 4.42 4.42 4.07 4.06 4.05 4.05 4.04 4.03 4.03 4.02
3.55 3.54 3.53 3.52 3.36 3.36 3.36 3.34 3.34 3.34 3.32 3.31 3.31 3.31 3.30 3.15 3.14 3.11 3.11 2.71 2.69 2.67 2.65 1.41 1.40 1.29 1.28 1.27 1.27
H_3C
HO
CH_3
(-)-Angiopterlactone B (**24**)
CD_3OD
2 1 1 2 1 1 1 1 1 3 3
10.5 10.0 9.5 9.0 8.5 8.0 7.5 7.0 6.5 6.0 5.5 5.0 4.5 4.0 3.5 3.0 2.5 2.0 1.5 1.0 0.5 0.0 -0.5

$^{13}C\{^{1}H\}$ NMR, 126 MHz, CD_3OD

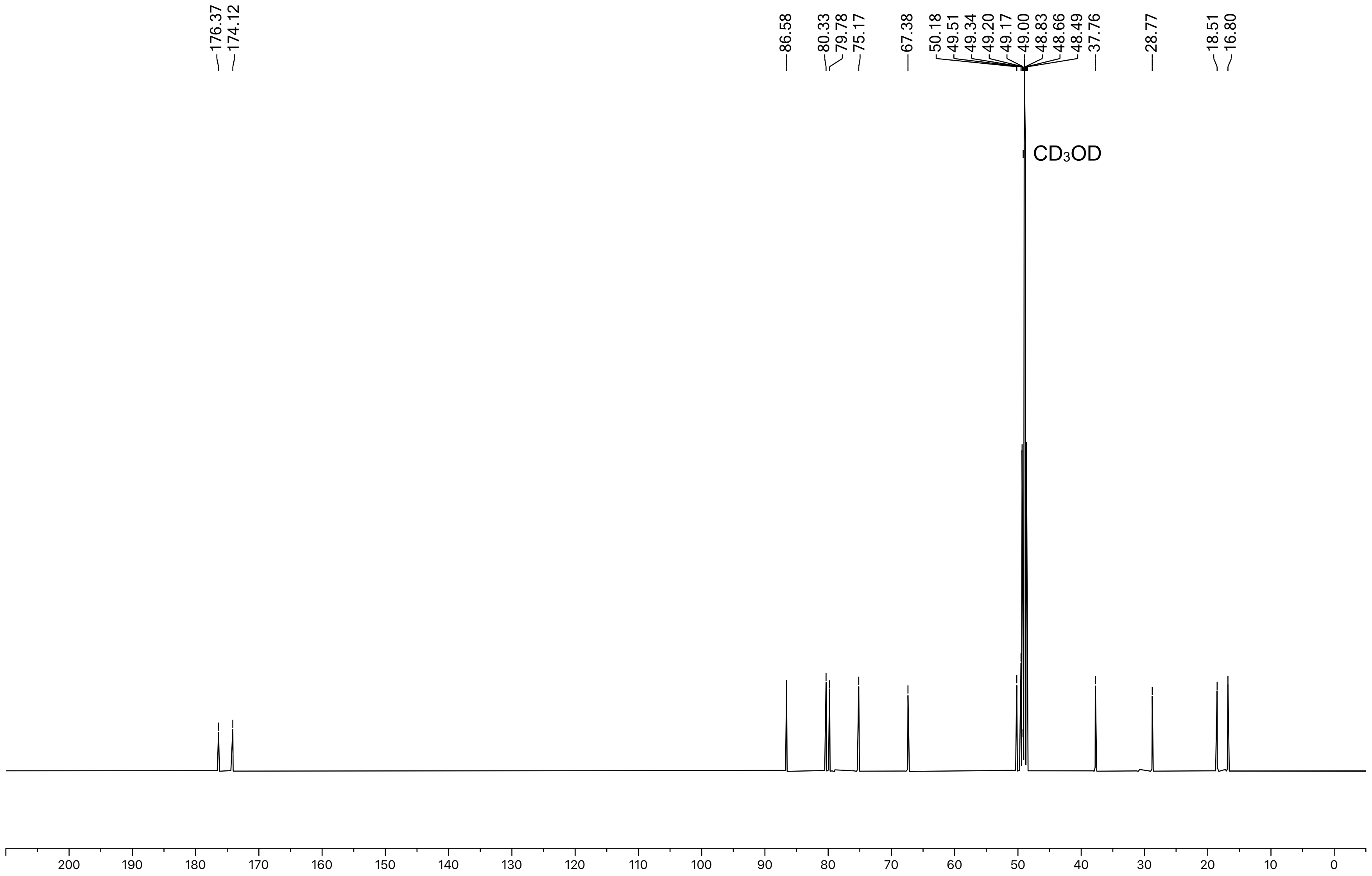

^{1}H NMR, 500 MHz, $CDCl_3$

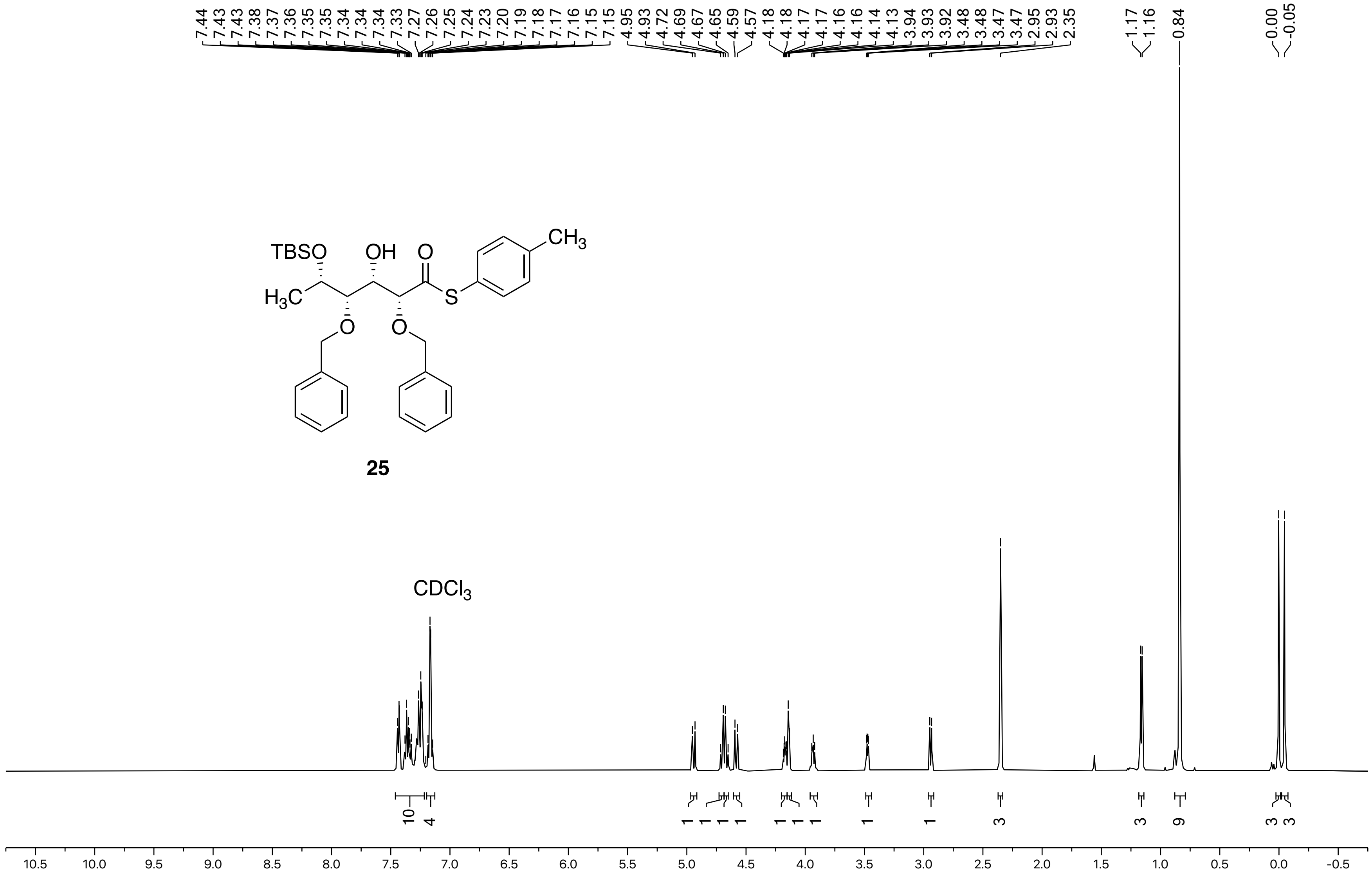

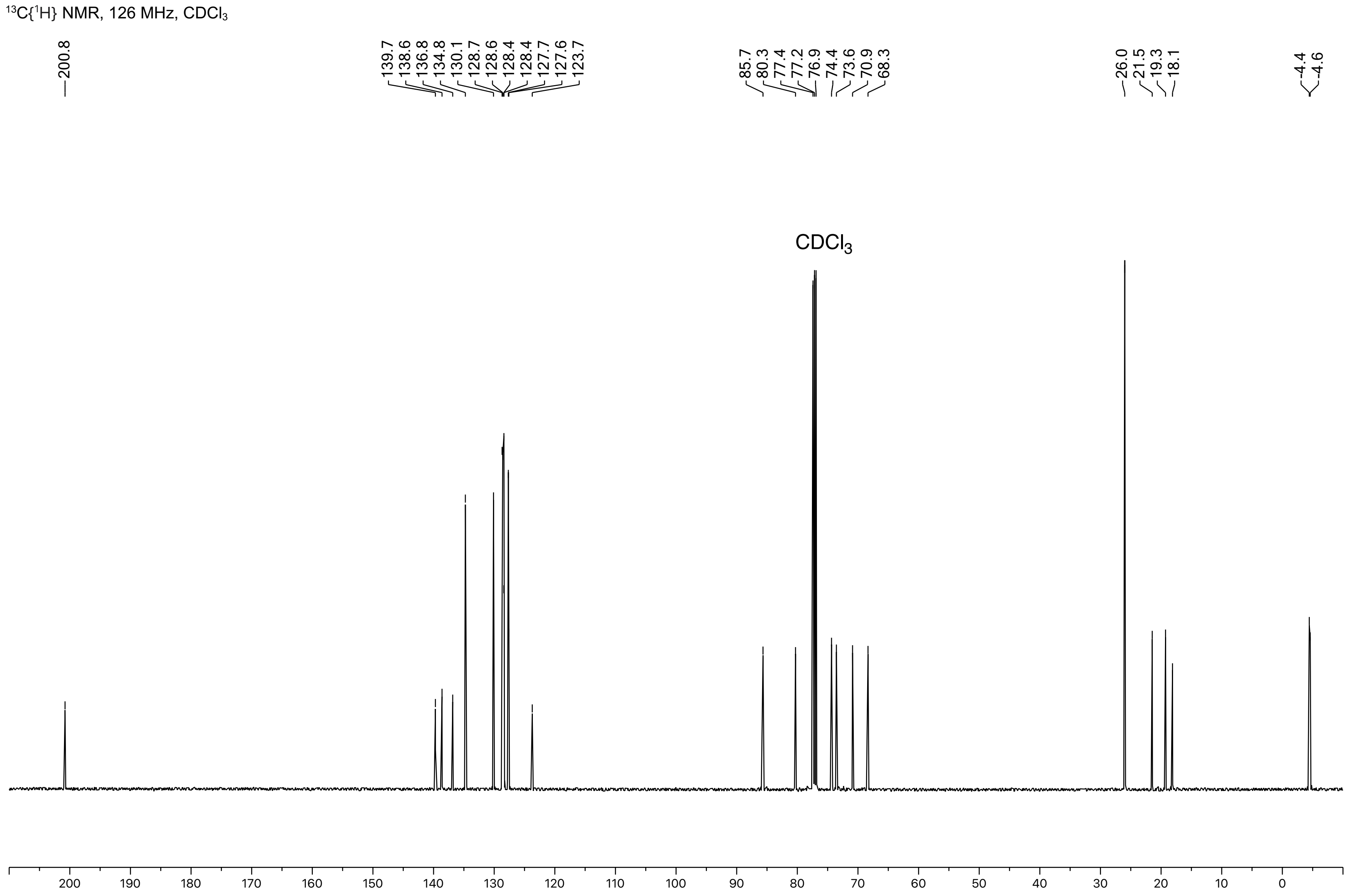

$^{13}C\{^1H\}$ NMR, 126 MHz, $CDCl_3$
200.8
139.7
138.6
136.8
134.8
130.1
128.7
128.6
128.4
128.4
127.7
127.6
123.7
85.7
80.3
77.4
77.2
76.9
74.4
73.6
70.9
68.3
26.0
21.5
19.3
18.1
-4.4
-4.6
$CDCl_3$
200
190
180
170
160
150
140
130
120
110
100
90
80
70
60
50
40
30
20
10
0

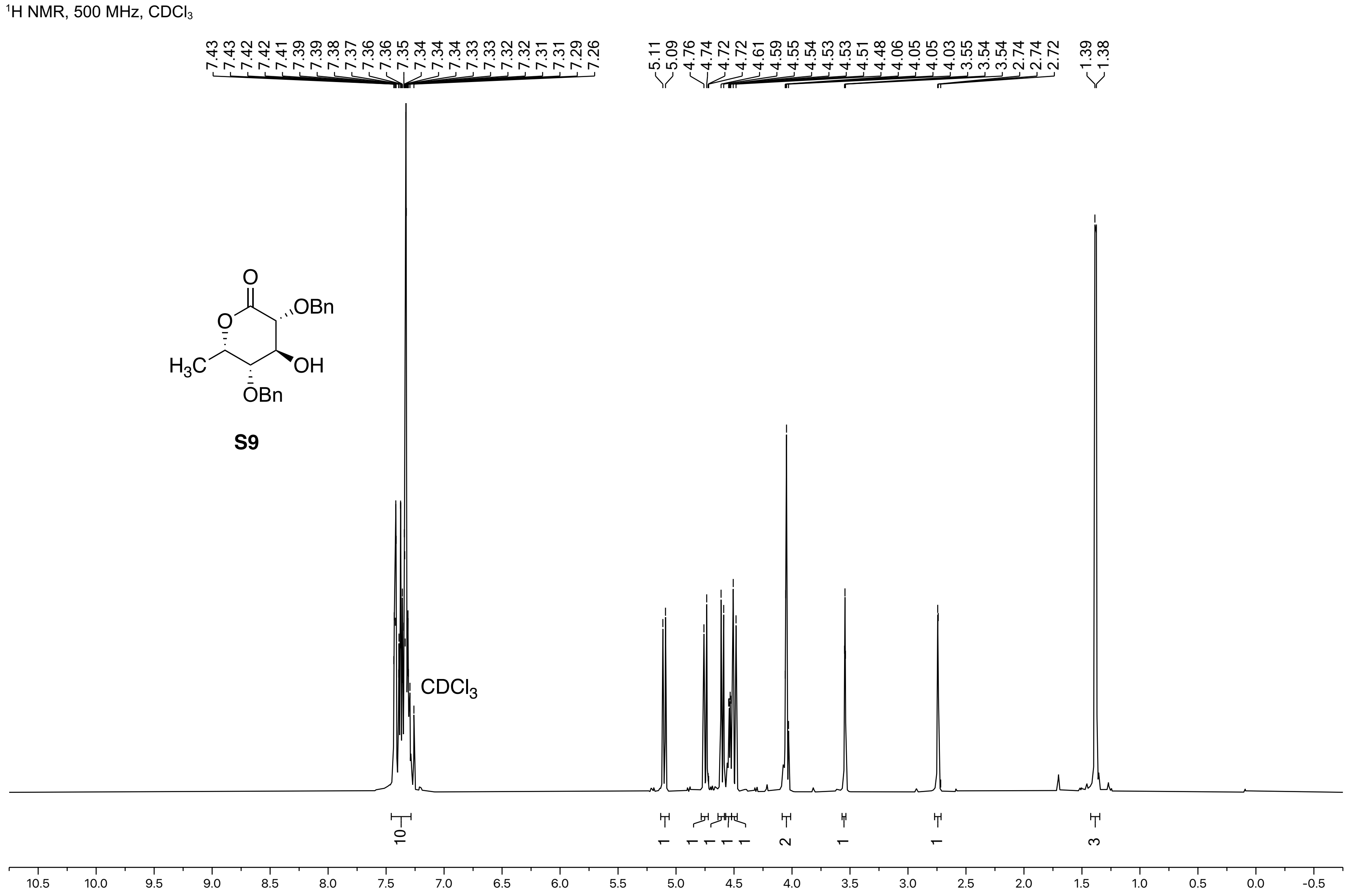
1H NMR, 500 MHz, CDCl3
S9
CDCl3

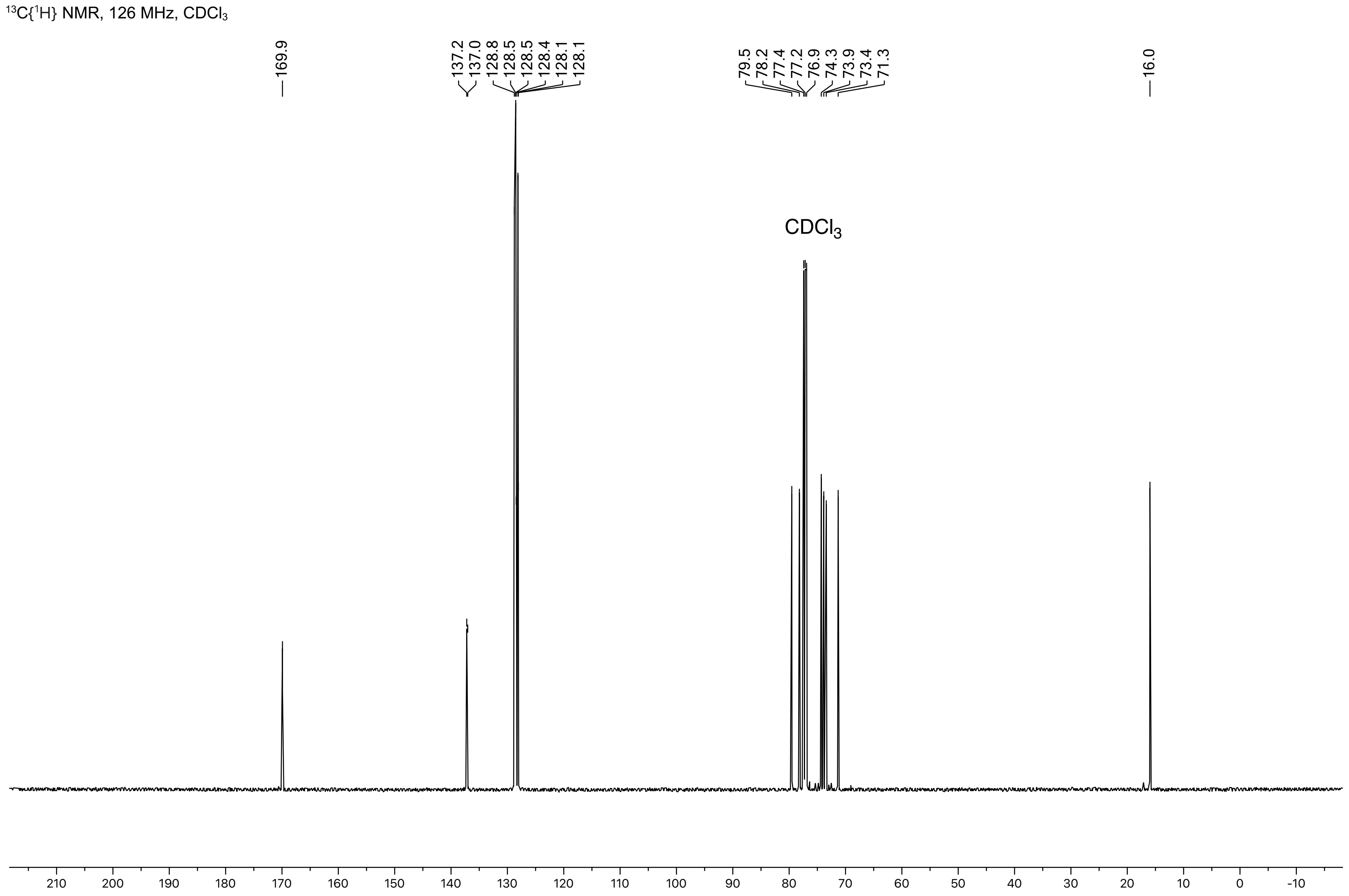
13C{1H} NMR, 126 MHz, CDCl3
169.9
137.2
137.0
128.8
128.5
128.5
128.4
128.1
128.1
79.5
78.2
77.4
77.2
76.9
74.3
73.9
73.4
71.3
16.0
CDCl3
210
200
190
180
170
160
150
140
130
120
110
100
90
80
70
60
50
40
30
20
10
0
-10

^1H NMR, 500 MHz, $CDCl_3$

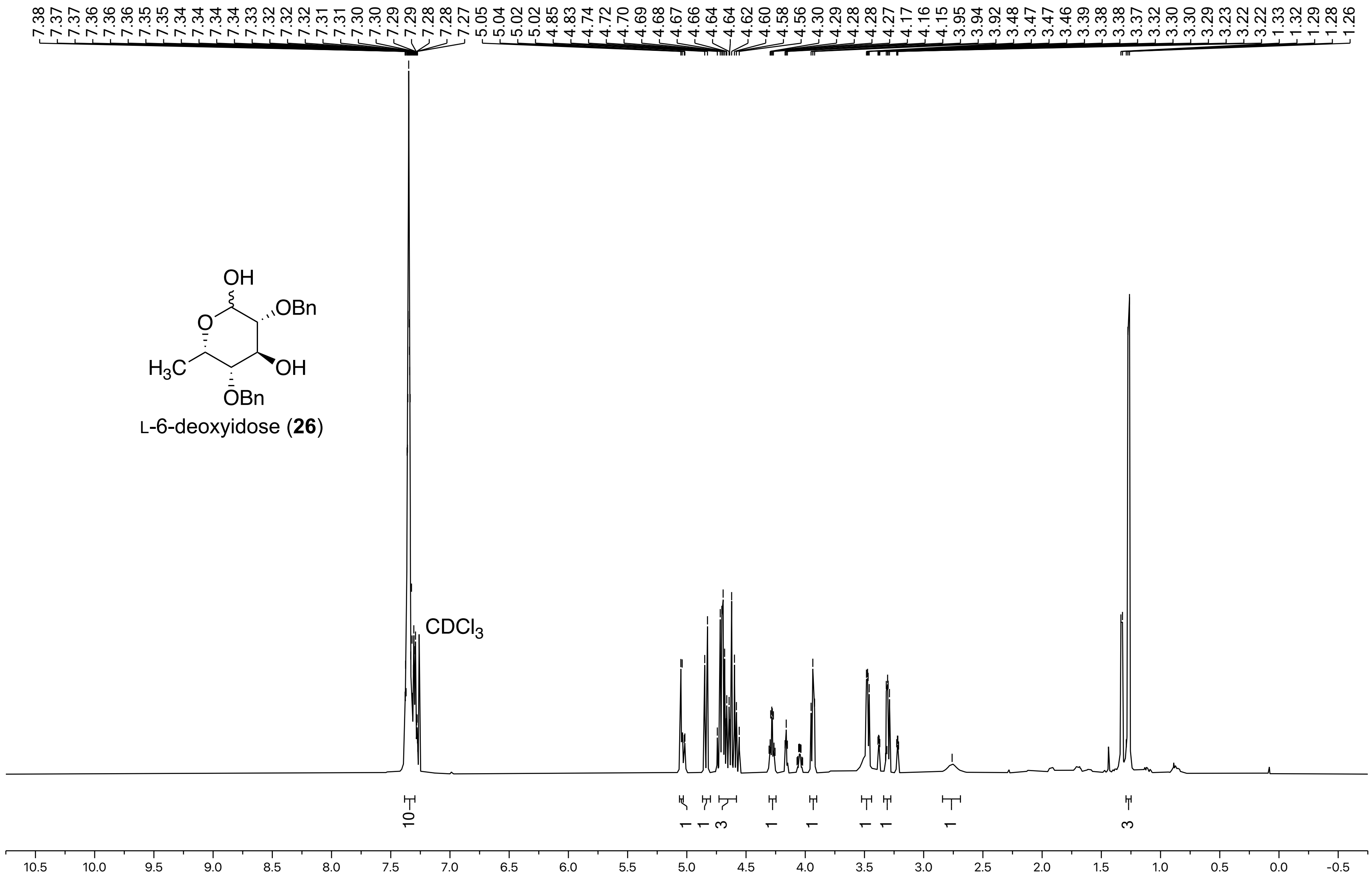

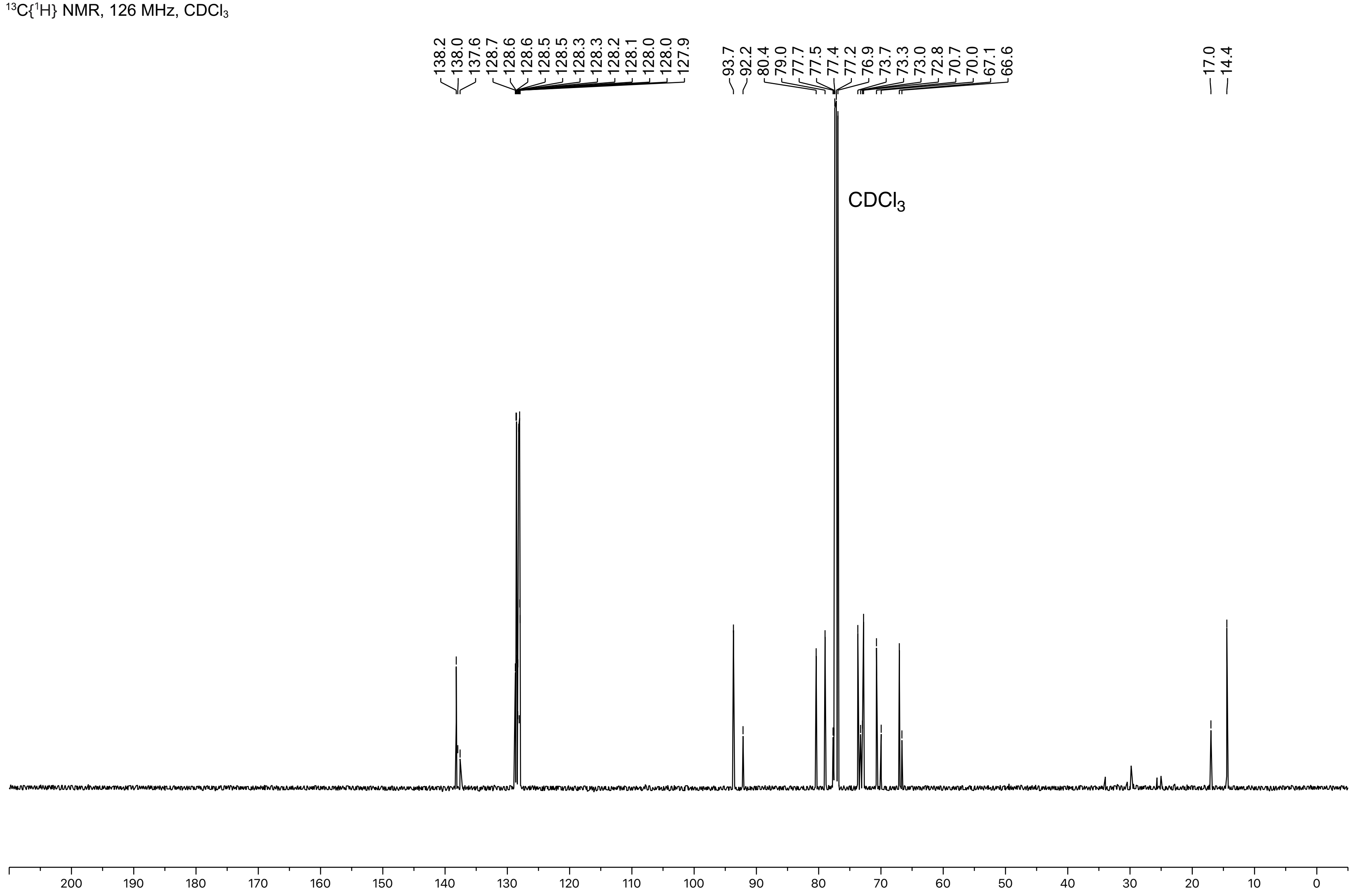

$^{13}C\{^1H\}$ NMR, 126 MHz, $CDCl_3$
138.2
138.0
137.6
128.7
128.6
128.6
128.5
128.5
128.3
128.3
128.2
128.1
128.0
128.0
127.9
93.7
92.2
80.4
79.0
77.7
77.5
77.4
77.2
76.9
73.7
73.3
73.0
72.8
70.7
70.0
67.1
66.6
17.0
14.4
$CDCl_3$
200
190
180
170
160
150
140
130
120
110
100
90
80
70
60
50
40
30
20
10
0

Catalog of X-ray data

a) Crystal data of (2*R*,3*S*)-**10a**

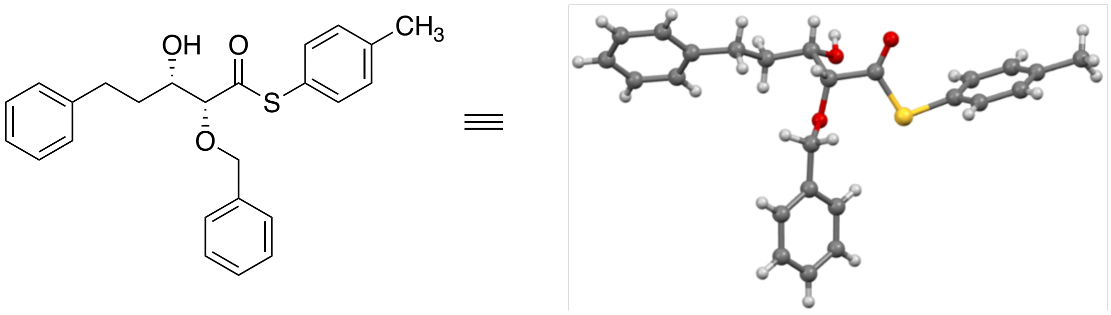

Molecular structure of **10a** (ellipsoid contours of probability levels are 50%).

Identification code	(2*R*,3*S*)-**10a**
Empirical formula	$C_{25}H_{26}O_3S$
Formula weight	406.549
Temperature/K	296.15
Crystal system	monoclinic
Space group	$P2_1$
a/Å	14.0020(8)
b/Å	5.5591(3)
c/Å	15.2697(9)
α/°	90
β/°	114.666(2)
γ/°	90
Volume/Å^3	1080.12(11)
Z	2
ρ_{calc}g/cm^3	1.250
μ/mm^{-1}	0.173
F(000)	432.5
Crystal size/mm^3	0.3 × 0.25 × 0.2
Radiation	Mo Kα (λ = 0.71073)
2Θ range for data collection/°	3.32 to 61.08
Index ranges	$-19 \le h \le 19$, $-7 \le k \le 7$, $-21 \le l \le 21$
Reflections collected	26864
Independent reflections	6425 [R_{int} = 0.0485, R_{sigma} = 0.0405]
Data/restraints/parameters	6425/1/264
Goodness-of-fit on F^2	1.046
Final R indexes [I>=2σ (I)]	R_1 = 0.0591, wR_2 = 0.1667
Final R indexes [all data]	R_1 = 0.0696, wR_2 = 0.1822
Largest diff. peak/hole / e Å^{-3}	0.27/-0.37
Flack parameter	0.05(3)
CCDC:	2371527

b) Crystal data of (2*R*,3*S*)-**10e**

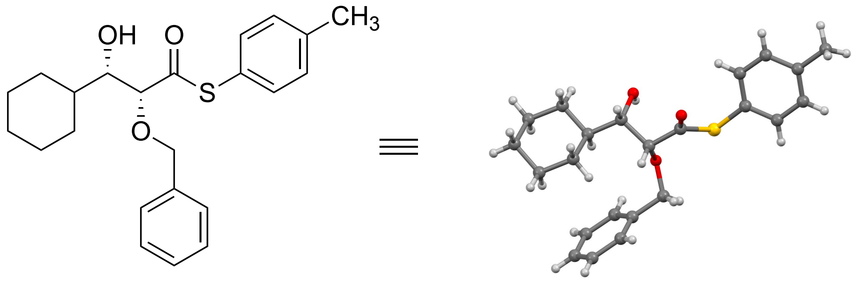

Molecular structure of **10e** (ellipsoid contours of probability levels are 50%).

Identification code	(2*R*,3*S*)-**10e**
Empirical formula	$C_{23}H_{28}O_3S$
Formula weight	384.51
Temperature/K	180
Crystal system	triclinic
Space group	P-1
a/Å	8.5428(3)
b/Å	9.6819(3)
c/Å	14.3851(5)
α/°	77.9320(10)
β/°	74.1800(10)
γ/°	65.4830(10)
Volume/Å^3	1035.25(6)
Z	2
ρ_{calc}g/cm^3	1.234
μ/mm^{-1}	0.176
F(000)	412.0
Crystal size/mm^3	0.3 × 0.1 × 0.1
Radiation	MoKα (λ = 0.71073)
2Θ range for data collection/°	2.96 to 81.8
Index ranges	-14 ≤ h ≤ 14, -17 ≤ k ≤ 17, -26 ≤ l ≤ 24
Reflections collected	73016
Independent reflections	11776 [R_{int} = 0.0314, R_{sigma} = 0.0317]
Data/restraints/parameters	11776/0/246
Goodness-of-fit on F^2	1.063
Final R indexes [I>=2σ (I)]	R_1 = 0.0548, wR_2 = 0.1329
Final R indexes [all data]	R_1 = 0.0868, wR_2 = 0.1462
Largest diff. peak/hole / e Å^{-3}	0.52/-0.42
CCDC:	2367925

c) Crystal data of (2*R*,3*S*)-**10p**

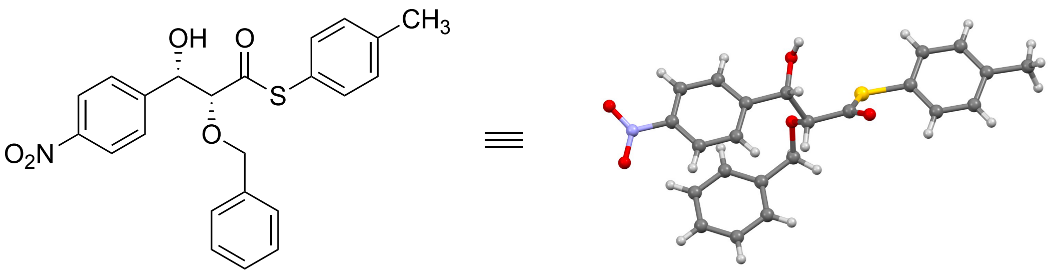

Molecular structure of **10p** (ellipsoid contours of probability levels are 50%).

Identification code	(2*R*,3*S*)-**10p**
Empirical formula	$C_{23}H_{21}NO_5S$
Formula weight	423.47
Temperature/K	200
Crystal system	monoclinic
Space group	$P2_1$
a/Å	11.616(3)
b/Å	7.0317(14)
c/Å	13.144(3)
α/°	90
β/°	104.545(7)
γ/°	90
Volume/Å^3	1039.3(4)
Z	2
ρ_{calc}g/cm^3	1.353
μ/mm^{-1}	0.191
F(000)	444.0
Crystal size/mm^3	0.6 × 0.15 × 0.15
Radiation	MoKα (λ = 0.71073)
2Θ range for data collection/°	3.202 to 71.584
Index ranges	-17 ≤ h ≤ 18, -10 ≤ k ≤ 11, -20 ≤ l ≤ 21
Reflections collected	42720
Independent reflections	7903 [R_{int} = 0.0275, R_{sigma} = 0.0206]
Data/restraints/parameters	7903/1/273
Goodness-of-fit on F^2	1.016
Final R indexes [I>=2σ (I)]	R_1 = 0.0323, wR_2 = 0.0899
Final R indexes [all data]	R_1 = 0.0350, wR_2 = 0.0917
Largest diff. peak/hole / e Å^{-3}	0.40/-0.17
Flack parameter	0.061(11)
CCDC:	2367935

d) Crystal data of (2*R*,3*S*)-**10r**

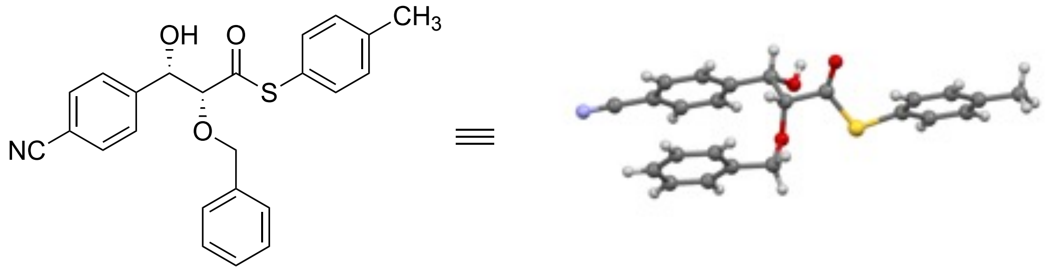

Molecular structure of **10r** (ellipsoid contours of probability levels are 50%).

Identification code	(2*R*,3*S*)-**10r**
Empirical formula	$C_{24}H_{21}NO_3S$
Formula weight	403.48
Temperature/K	296.15
Crystal system	monoclinic
Space group	$P2_1$
a/Å	11.3396(5)
b/Å	7.1759(3)
c/Å	13.0278(6)
α/°	90
β/°	101.464(2)
γ/°	90
Volume/Å^3	1038.95(8)
Z	2
ρ_{calc}g/cm^3	1.290
μ/mm^{-1}	0.181
F(000)	424.0
Crystal size/mm^3	0.4 × 0.15 × 0.14
Radiation	MoKα (λ = 0.71073)
2Θ range for data collection/°	4.354 to 71.402
Index ranges	-18 ≤ h ≤ 18, -11 ≤ k ≤ 11, -21 ≤ l ≤ 21
Reflections collected	89313
Independent reflections	9560 [R_{int} = 0.0293, R_{sigma} = 0.0151]
Data/restraints/parameters	9560/1/264
Goodness-of-fit on F^2	1.061
Final R indexes [I>=2σ (I)]	R_1 = 0.0342, wR_2 = 0.0887
Final R indexes [all data]	R_1 = 0.0368, wR_2 = 0.0906
Largest diff. peak/hole / e Å^{-3}	0.39/-0.20
Flack parameter	0.064(8)
CCDC:	2367938

e) Crystal data of (2*R*,3*S*)-**10ad**

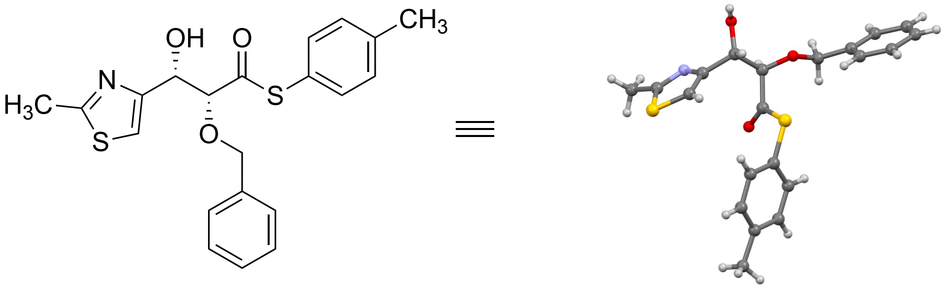

Molecular structure of **10ad** (ellipsoid contours of probability levels are 50%).

Identification code	(2*R*,3*S*)-**10ad**
Empirical formula	$C_{21}H_{21}NO_3S_2$
Formula weight	399.51
Temperature/K	180
Crystal system	monoclinic
Space group	$P2_1$
a/Å	11.0534(4)
b/Å	5.6238(2)
c/Å	16.2023(6)
α/°	90
β/°	91.515(2)
γ/°	90
Volume/Å^3	1006.82(6)
Z	2
ρ_{calc}g/cm^3	1.318
μ/mm^{-1}	0.285
F(000)	420.0
Crystal size/mm^3	0.6 × 0.15 × 0.14
Radiation	MoKα (λ = 0.71073)
2Θ range for data collection/°	4.408 to 72.63
Index ranges	-18 ≤ h ≤ 17, -9 ≤ k ≤ 9, -26 ≤ l ≤ 26
Reflections collected	33400
Independent reflections	9717 [R_{int} = 0.0211, R_{sigma} = 0.0192]
Data/restraints/parameters	9717/1/247
Goodness-of-fit on F^2	1.063
Final R indexes [I>=2σ (I)]	R_1 = 0.0284, wR_2 = 0.0806
Final R indexes [all data]	R_1 = 0.0309, wR_2 = 0.0823
Largest diff. peak/hole / e Å^{-3}	0.39/-0.20
Flack parameter	0.068(9)
CCDC:	2367937

Bibliography

[1] Rahman, Md. A.; Cellnik, T.; Ahuja, B. B.; Li, L.; Healy, A. R. A Catalytic Enantioselective Stereodivergent Aldol Reaction. *Sci. Adv.* **2023**, *9* (11), eadg8776. https://doi.org/10.1126/sciadv.adg8776.

[2] Podunavac, M.; Mailyan, A. K.; Jackson, J. J.; Lovy, A.; Farias, P.; Huerta, H.; Molgó, J.; Cardenas, C. C.; Zakarian, A. Scalable Total *Synthesis*, IP3R Inhibitory Activity of Desmethylxestospongin B, and Effect on Mitochondrial Function and Cancer Cell Survival. *Angew. Chem. Int. Ed.* **2021**, *60* (20), 11278–11282. https://doi.org/10.1002/anie.202102259.

[3] Kobayashi, H.; Eickhoff, J. A.; Zakarian, A. *Synthesis of* 2-Aminoazoles from Thioesters via α-Heterosubstituted Ketones by Copper-Mediated Cross-Coupling. *J. Org. Chem.* **2015**, *80* (20), 9989–9999. https://doi.org/10.1021/acs.joc.5b01558.

[4] Vugts, D. J.; Veum, L.; al-Mafraji, K.; Lemmens, R.; Schmitz, R. F.; de Kanter, F. J. J.; Groen, M. B.; Hanefeld, U.; Orru, R. V. A. A Mild Chemo-Enzymatic Oxidation–Hydrocyanation Protocol. *Eur. J. Org. Chem.* **2006**, *7*, 1672–1677. https://doi.org/10.1002/ejoc.200500905.

[5] Trost, B. M.; Seganish, W. M.; Chung, C. K.; Amans, D. Total *Synthesis of* Laulimalide: *Synthesis of* the Northern and Southern Fragments. *Chem. Eur. J.* **2012**, *18*, 2948–2960. https://doi.org/10.1002/chem.201102898.

[6] Geall, A. J.; Blagbrough, I. S. Homologation of Polyamines in the Rapid *Synthesis of* Lipospermine Conjugates and Related Lipoplexes. *Tetrahedron* **2000**, *56*, 2449–2460. https://doi.org/10.1016/S0040-4020(99)01082-0.

[7] Evans, D. A.; Chapman, K. T.; Carreira, E. M. Directed Reduction of β-Hydroxy Ketones Employing Tetramethylammonium Triacetoxyborohydride. *J. Am. Chem. Soc.* **1988**, 110 (11), 3560–3578. https://doi.org/10.1021/ja00219a035.

[8] Rychnovsky, S. D.; Rogers, B.; Yang, G. Analysis of two carbon-^{13}NMR correlations for determining the stereochemistry of 1,3-diol acetonides. *J. Org. Chem*. **1993**, *58*, 3511– 3515. https://doi.org/10.1021/jo00065a011.

[9] Rychnovsky, S. D.; Skalitzky, D. J. Stereochemistry of alternating polyol chains: ^{13}C NMR analysis of 1,3-diol acetonides. *Tetrahedron Lett.* **1990**, *31*, 945– 948. https://doi.org/10.1016/S0040-4039(00)94399-5.

[10] Thomson, M. I.; Nichol, G. S.; Lawrence, A. L. Total *Synthesis of* (−)-Angiopterlactone B. *Org. Lett.* **2017**, *19* (9), 2199–2201. https://doi.org/10.1021/acs.orglett.7b00929.

[11] Davies, S. G.; Nicholson, R. L.; Smith, A. D. A SuperQuat glycolate aldol approach to the asymmetric *Synthesis of* hexose monosaccharides. *Org. Biomol. Chem*. **2005**, *3*, 348–359. https://doi.org/10.1039/B415943H.
